# Photochemical Flow Synthesis of Trisubstituted Oxazoles Enabled by High-Power UV–B LED Modules

**DOI:** 10.1021/acs.orglett.5c03241

**Published:** 2025-09-04

**Authors:** Ruairi Crawford, Rémy Broersma, Frouke van den Berg, Marcus Baumann

**Affiliations:** † School of Chemistry, 8797 University College Dublin, Dublin 4, Ireland; ‡ 42359 Signify Research, High Tech Campus 7, 5656 AE Eindhoven, The Netherlands

## Abstract

The use of a newly developed UV-B LED module for the continuous flow synthesis of a variety of trisubstituted oxazole products from readily available isoxazoles is reported. Crucially, this work demonstrates for the first time that these UV-LED modules (up to 40 W electrical power) are effective replacements of classical medium-pressure Hg-lamps by providing monochromatic light (centered at 308 nm) which facilitates the highlighted photochemical process. The desired oxazole products are afforded in high yields, and the applicability toward synthesizing analogs of the anti-inflammatory drug oxaprozin at multigram scale is reported. The availability of such high-power UV-LED modules not only circumvents the use of inefficient metal vapor lamps but also highlights new opportunities for light-driven processes in general.

## Graphical abstract

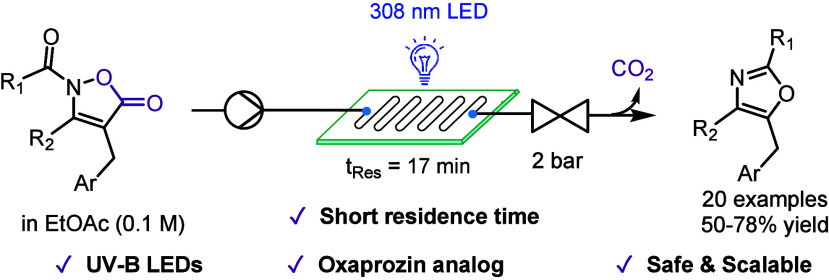

The use of ultraviolet (UV) light has a long tradition of triggering a large variety of photochemical reactions including photocycloadditions, rearrangements, and other unique transformations that harness the excited state of organic molecules. While the direct photochemical excitation of starting materials offers a facile route to a plethora of products unattainable through complementary thermal reactions, a major challenge concerns the ability to achieve this at high levels of selectivity. Therefore, substantial amounts of side products often accompany the formation of the desired photoproduct when employing this strategy. Metal vapor lamps (e.g., Hg-lamps) are commonly used when light with wavelengths of 200–400 nm is required; however, these light sources emit not only light across the entire UV–vis spectrum but also generate significant amounts of heat requiring active cooling mechanisms to be in place.^1,2^

The huge popularity of photochemical reactions over the past two decades^3^ can be explained by the availability of modern LEDs emitting near-monochromatic light in the visible and UV-A region. When coupled with suitable photocatalysts the use of LEDs enables the selective activation of a variety of bonds in densely functionalized substrates thus covering a large chemical space of structurally complex molecular entities.^4–6^ Concurrent with the advent of high-power LEDs continuous flow techniques based on miniaturized set-ups have been exploited to overcome limitations of photochemical reactions such as their scalability which is governed by the Beer–Lambert law.^7,8^ As highlighted in the literature, the use of continuous flow photochemistry provides a simple solution to achieve the expedited synthesis of target molecules in a scalable manner which facilitates diverse applications in both academic and industrial settings.^9–12^ Moreover, continuous processing provides for spatiotemporal resolution of the reaction mixture, which often translates into higher yields by minimizing overirradiation and thus product decomposition.

One major limitation in this field is the lack of LEDs emitting monochromatic light with high optical power at wavelengths below 365 nm. As a consequence, many studies describing the use of UV-light in batch^13^ as well as flow-based^14,15^ photoreactions resort to the use of medium-pressure Hg-lamps that are energy inefficient and result in the formation of various side products. To address this issue and overcome this bottleneck, we set out to develop a set of high-intensity LED modules emitting in the UV-B and UV-C region. Here we disclose our findings when exploiting such UV-LEDs for the photochemical flow synthesis of trisubstituted oxazoles from isoxazolones (Scheme 1, bottom).

**Scheme 1:**
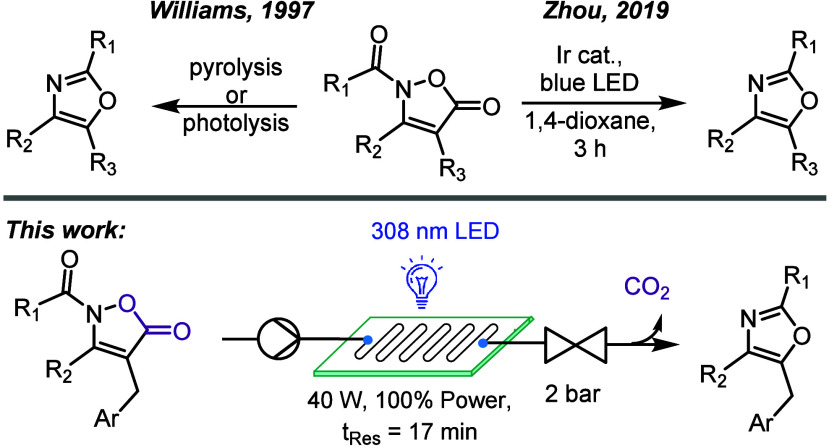
Literature Synthesis of Oxazoles from Isoxazolones

Oxazoles represent a class of biologically relevant heterocycles that can be prepared via a variety of well-established routes.^16–21^ Within the literature several studies report access to di/trisubstituted oxazoles from 2-acylisoxazol-5-ones (Scheme 1, top).^22–24^ For instance, flash vacuum pyrolysis and photolysis both have been described; however, these approaches require specialized equipment, accompanied by high operational costs and safety concerns. An interesting alternative was recently reported by Zhou which involves the photochemical transformation of *N*-acylated isoxazolones via photoredox catalysis (Scheme 1).^24^ This approach involves the photochemical decarboxylation of the substrate, followed by cyclization of a putative radical intermediate affording the heteroaromatic target. Inspired by this approach, our own efforts commenced by evaluating the conversion of isoxazolone (**1a**) into the desired oxazole product (**2a**) using different light sources as highlighted in Table 1. This study employed a standard flow approach whereby solutions of **1** (100 mM EtOAc) were directed with a peristaltic pump through either a glass chip (2 mL volume) or a tubular reactor coil (10 mL volume, PFA tubing 1/16’ i.d., housed inside the UV-150 unit of a Vapourtec flow module) and a subsequent back-pressure regulator (set to 2 bar) to facilitate the steady release of the CO_2_ byproduct.

**Table 1:**
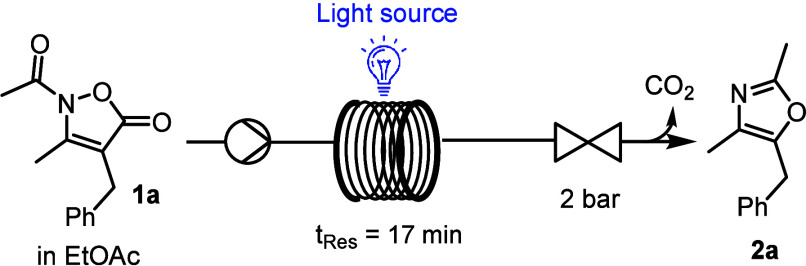
Initial Screening of Various UV-Light Sources^a^

Entry	Light Source	Yield of **2a** (%)
1	Hg-lamp (107 W)	61 (14)
2	365 nm (LED, 70 W)	ND
2	330 nm (LED, 40 W)	ND
3	308 nm (LED, 40 W)	71
4	280 nm (LED, 40 W)	37 (50)
5	265 nm (LED, 40 W)	5 (70)

a Reactions were performed at 0.35 mmol scale in EtOAc at 100 mM. Reported yields are ^1^H NMR yields of compound **2a** (% of remaining starting material in brackets) using trichloroethylene as internal standard. ND – not detected.

As shown in Table 1, the desired oxazole formation was realized in a residence time of 17 min when using a medium-pressure Hg-lamp (107 W input power) in combination with the Vapourtec flow reactor system; however, active cooling using a stream of compressed air and a low-pass filter blocking wavelengths above 400 nm were necessary to obtain **2** in an acceptable yield of 61% which was accompanied by several unidentified side products (entry 1). Importantly, when using a high-power LED emitting at 365 nm (70 W input power, t_Res_ = 17 min) and the same flow setup, no conversion to the desired oxazole product was observed (entry 2). This result clearly shows that wavelengths below 365 nm are critical for the desired transformation. Next, we trialed a set of newly developed high-power LED spot modules from Signify emitting monochromatic light in the UV-B and UV-C regions (entries 3–6). The photochemistry spot modules have a small rectangular-shaped light emitting window with an array of UV LEDs and are air-cooled. The flow setup was modified using a glass chip instead of the tubular reactor coil but maintaining the same residence time as well as the use of the backpressure regulator to control the release of CO_2_.

Employing a 330 nm LED gave no conversion to product **2a** showing that there was no overlap between the absorbance of **1a** and the emitted LED light (see (SI) for details). In contrast, utilizing an LED with a wavelength of 308 nm gave the best result for the formation of **2a** (71% yield, entry 3). Attempts with LEDs emitting at 280 and 265 nm (entries 4–5) gave a lower conversion to **2a** with considerable amounts of unreacted **1a** remaining. This can be attributed to the ability of the borosilicate glass absorbing at 288 nm.^25^ Also, George and co-workers reported the UV-cut off for borosilicate glass to be 280 nm^15^ which ultimately resulted in poor photon penetration into the reactor zone for entries 4 and 5.

As the setup was homemade (see for details) we were able to study the preferred distance of the LEDs from the glass-chip reactor (Table 2). The short path length provided by flow photochemistry is a major contributor for reduced reaction times compared to batch mode.^4^ Overall, a distance of 20 mm clearly showed the best performance (entry 1) and ultimately enabled effective irradiation of the reaction solution. Furthermore, increasing the distance to 30 and 40 mm resulted in starting material remaining and thus unsatisfactory conversion to **2a** (entries 2–3) which is caused by a reduced average photon density due to the increased distance. Moreover, this indicates that longer reaction times would be required for full conversion.

**Table 2:** Distance of Light Source from Chip-Reactor^a^

Entry	Distance (mm)	Yield of **2a** (%)
1	20	71
2	30	63 (17)
3	40	61 (24)

a Reactions were performed at 0.35 mmol scale in EtOAc at 100 mM. Reported yields are ^1^H NMR yields of compound **2a** (% of remaining starting material in brackets) using trichloroethylene as internal standard.

Looking closely at the report from Zhou in 2019,^24^ a significant drop in the yield was observed for oxazoles bearing a methyl group as the R_1_ substituent (yields 36–41%). Taking this into account, we used this opportunity to establish whether our approach would overcome this limitation (Scheme 2). Our initial studies varied the alkyl chain length (**2a**–**d**) achieving superior yields between 61% and 67%. Moreover, strained rings, as found in products **2e** and **2f**, can be utilized without ring opening occurring. Incorporating a 6-membered ring (**2g**) was also feasible alongside introducing an oxygen atom into the cyclohexyl ring gaining access to a pyran derivative (**2h**). We then investigated the compatibility of the transformation for prefunctionalized molecules containing an ester (**2i**) and a ketone (**2j**) which due to its success provides sites for further functionalization. Incorporating an adamantane system was also achieved, indicating that bulky cage-like structures are tolerated at the acyl portion, giving the desired product **2k** in a high yield of 78%.

**Scheme 2:**
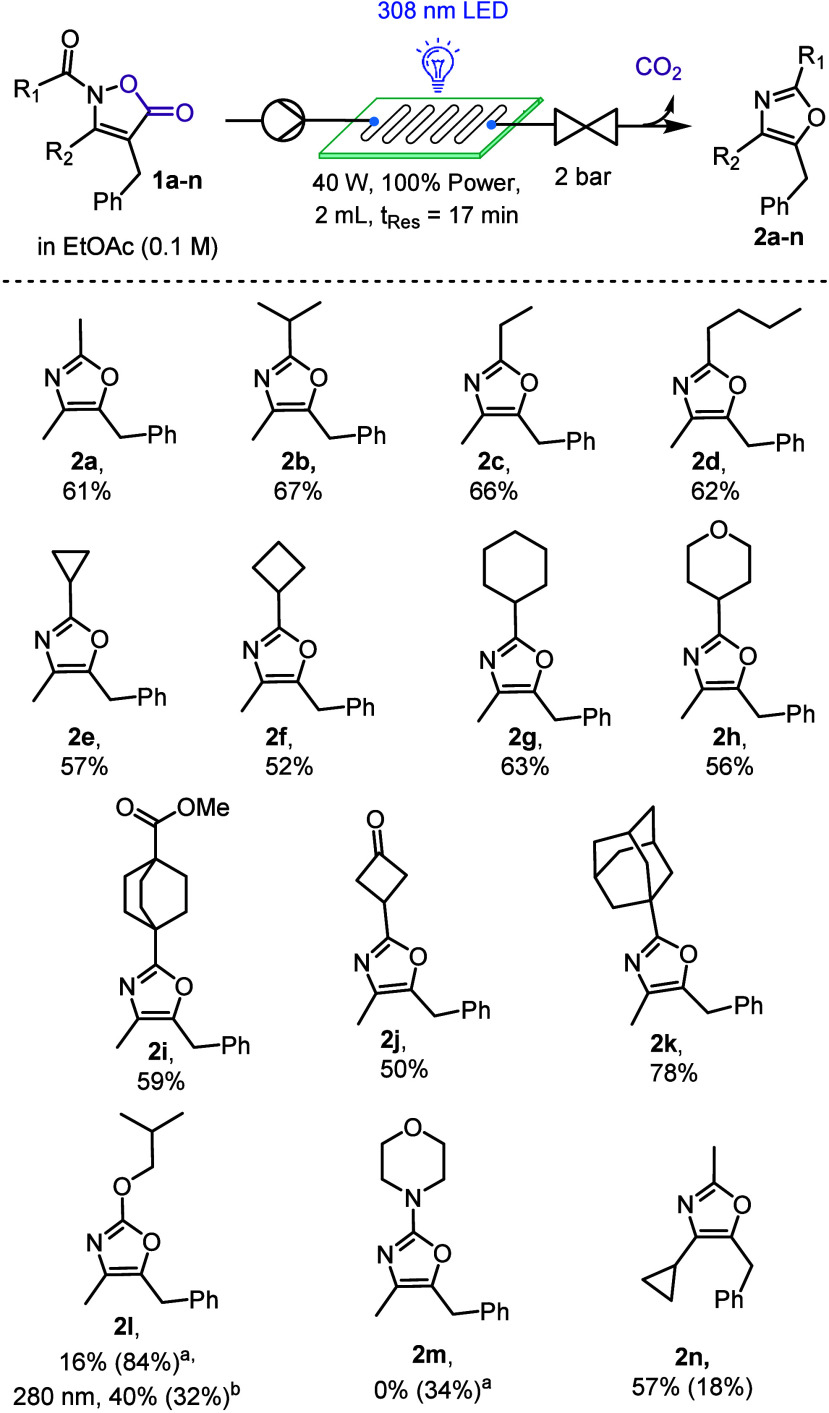
Reported Yields Are Isolated Yields (% Starting Material in Brackets)

a
^1^H NMR yield using trichloroethylene as internal standard.
b Residence time utilized was 8.5 min with ^1^H NMR yield reported.

Accessing the 2-alkoxy derivative **2l** was possible albeit at a reduced yield of 16% which was accompanied by a significant amount of starting material (84%). This result can be explained by the reduced absorbance of the corresponding substrate at 308 nm (see for details). Changing the LED light source to 280 nm and utilizing PFA tubing enabled an improved outcome, affording product **2l** in 40% yield with 32% of substrate remaining (t_Res_ 8.5 min). Analogously, the synthesis of morpholine derivative **2m** was attempted; however, in this case only unreacted substrate and photodecomposition were observed. Lastly, the introduction of a cyclopropyl ring in the 3-position of the oxazole was effective, delivering product **2n** in good yield with only small amounts of substrate remaining.

We then switched our attention to the aryl portion of the oxazole scaffold and the compatibility of the transformation with reactive functionalities and heterocyclic rings (Scheme 3). To our delight, phenol containing product **2o** could be synthesized in 59% yield, with the hydroxyl group not requiring prior protection. Typically, using UV-B and UV-C irradiation cleavage of aryl bromides is a common occurrence due to the high energy of the photons utilized.^26^ However, we found that bromide **2p** can be synthesized effectively using our approach, thus providing a useful handle for further functionalization. Interestingly, the nitrogen atom of indole containing systems needed to be protected with a Boc group which also mitigated any solubility issues encountered otherwise (i.e., **2q** vs **2r**). Lastly, thiophene product **2s** was also accessible, demonstrating that electron-rich heterocycles are tolerated.

**Scheme 3:**
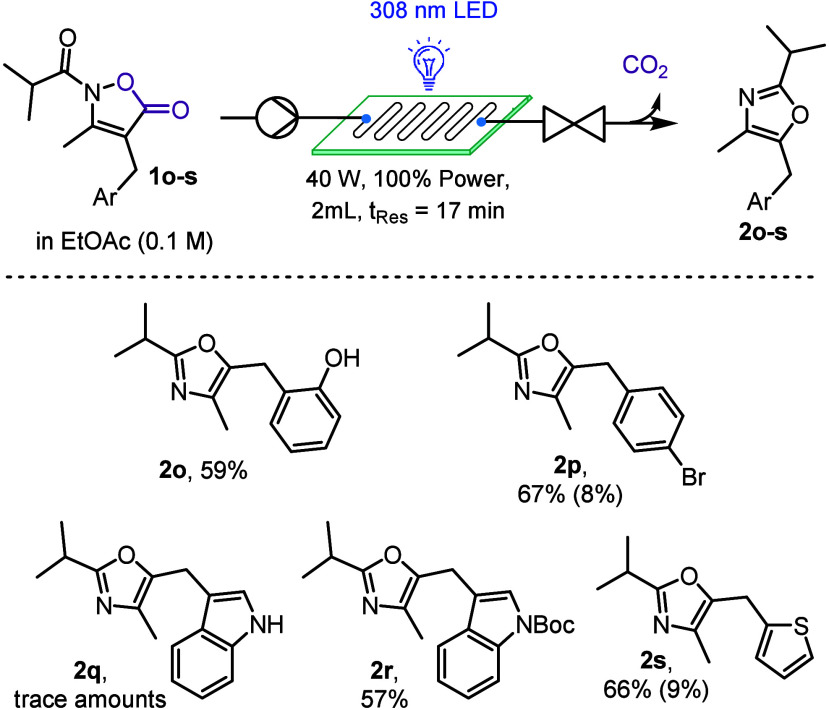
Oxazole Products with Variations at the 5-Position^a^

a Reported yields are isolated yields (% starting material in brackets).

Considering the wide range of pharmacological properties of oxazoles^17^ we next wished to apply our method to synthesizing an oxazole-based drug currently on the market. Oxaprozin, a nonsteroidal anti-inflammatory drug, emerged as an attractive target due to the trisubstituted nature of its oxazole core.^27^ Starting from succinic anhydride the required substrate **1t** was easily synthesized in 4 steps (Scheme 4, see for details). Flow chemistry is well-known for its inherent scalability to access gram quantities without need of significant reoptimization.^4^ As scaling flow reactions are primarily a function of time, we utilized the glass-chip reactor for a total of 13 h processing 2.3 g of **1t** under the standard conditions and achieving the synthesis of **2t** in a yield of 55%. Furthermore, **2t** was subjected to hydrolysis ultimately forming oxaprozin analog **3a** in 99% yield thus providing a new access to congeners of this important API which may stimulate further efforts making this drug and its analogs.

**Scheme 4:**
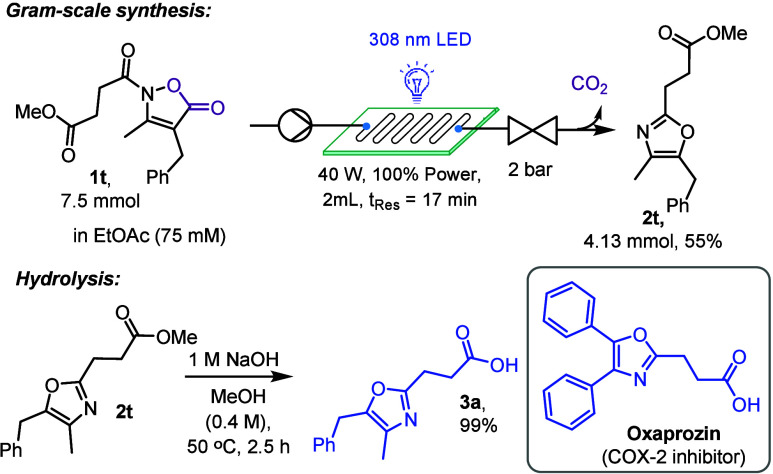
Synthesis of Oxaprozin Analog **3a**

Next, to gain insight into the formation of the oxazole products, we applied the standard conditions for the synthesis of **2a** in the presence of TEMPO (2,2,6,6-tetramethylpiperdin-1-yl)­oxyl, 1 equiv). Analyzing the resulting crude product sample by high-resolution mass spectrometry indicated that an adduct with TEMPO had formed (Scheme 5). Therefore, we propose that irradiation of **1a** leads to homolytic cleavage of the N–O bond generating both an amidyl radical and a carboxyl radical (**int1**). Nitrogen centered radicals have been reported by cleaving weak N–O and N–N bonds.^28^ Consequently, β-scission results in the decarboxylation event^29^ leading to the formation of **int2** which undergoes subsequent cyclization forming oxazole **2a**.

**Scheme 5:**
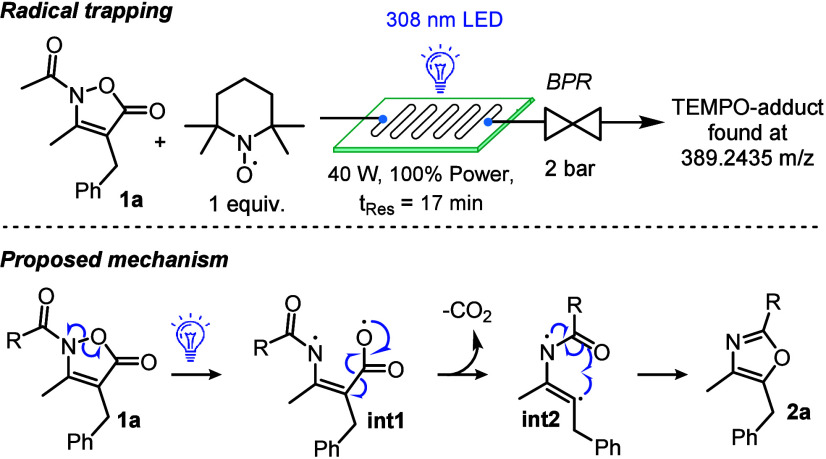
Radical Trapping Experiment and Proposed Mechanism for the Formation of **2a**

Having demonstrated the value of these newly developed UV-LED modules for the successful and scalable synthesis of a variety of oxazole products, we wished to evaluate whether further improvements to this flow approach can be made to provide further gains regarding productivity and photon efficiency. To this end, we placed an aluminum-based specular UV reflective sheet (provided by Signify) behind the glass reactor to reflect photons that did not initially trigger a photochemical reaction. If successful, this approach should increase the overall photon efficiency, leading to improved yield and impurity profiles at shorter residence time. Pleasingly, using this reflective sheet (ca. 0.5 cm distance from glass reactor) showed a significant enhancement of the reaction especially for short reaction times of 8.5 and 12.75 min (Table 3). This increase in performance therefore provides for higher productivity, which is attractive in view of additional reaction scale-up efforts while reducing the extent of side reactions.

**Table 3:**
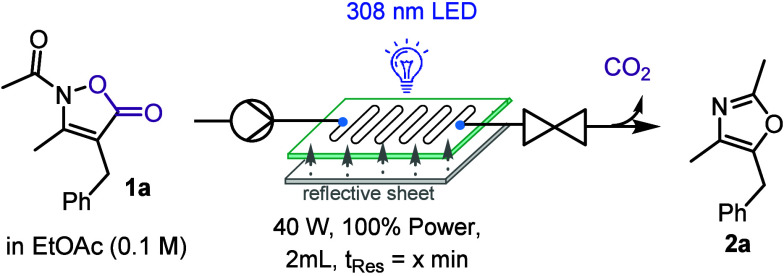
Effect of Reflective Material for the Synthesis of **2a** ^a^

t_res_ (min)	Yield of **2a** with reflective material	Yield of **2a** without reflective material
4.25	27 (62)	25 (61)
8.5	54 (32)	36 (38)
12.75	62 (15)	53 (15)
17	61 (4)	63 (4)

a Reactions were performed at 0.35 mmol scale in EtOAc at 100 mM. Reported yields are ^1^H NMR yields of compound **2a** (% starting material in brackets) using 1,3,5-trimethoxybenzene as internal standard.

In conclusion, we report on the exploitation of novel UV-B LED modules (308 nm) for the efficient and scalable generation of a variety of trisubstituted drug-like oxazoles. This process is triggered by the irradiation of isoxazolones which undergo a radical decarboxylation process forming the oxazole products in a short reaction time (8.5–17 min). The scalability of this continuous flow process was demonstrated by operating the reaction for a total of 13 h, accessing 1 g of an oxaprozin derivative, thus demonstrating the synthetic value of this route. Various alkyl and (hetero)­aryl appendages can be incorporated into the oxazole products, overcoming prior literature limitations to access these medicinally important entities. Overall, this method presents an attractive reagent-free route yielding diverse oxazoles by taking advantage of novel monochromatic UV-B LED modules that effectively replace classical yet inefficient metal vapor lamps.

## Supplementary Material





## Data Availability

The data underlying this study are available in the published article and its .

## Notes

Synthetic procedures, continuous flow setup, compound characterization and copies of NMR spectra (PDF)
FAIR data, including the primary NMR FID files, for compounds **S1**–**S14**, **1a**–**1s**, **2a**–**2t** and **3a** (ZIP)
