# Supplementary material for: Photochemical Flow Synthesis of Trisubstituted Oxazoles Enabled by High-Power UV–B LED Modules
Source: Org Lett. 2025 Sep 4;27(36):10152–6. doi: 10.1021/acs.orglett.5c03241 (PMC12442076; doi:10.1021/acs.orglett.5c03241)
Supplement: Supplementary file 2 [file ol5c03241_si_002.pdf]

Photochemical Flow Synthesis of Trisubstituted Oxazoles Enabled by High-Power UV-B LED Modules

Ruairi Crawford,[a] Rémy Broersma,[b] Frouke van den Berg[b] and Marcus Baumann[a,*]

Email: marcus.baumann@ucd.ie

[a] School of Chemistry, University College Dublin, Dublin 4, Ireland

[b] Signify Research, High Tech Campus 7, 5656 AE Eindhoven, The Netherlands

Contents

General Materials and Methods 3

Picture of Set-up 4

Reflectivity of Aluminum Sheet 5

Image of Reflective Material Set-up 5

UV-Vis Absorption 6

Experimental Procedures 8

General Procedure A – Synthesis of Isoxazol-5(4*H*)-ones (S1-6) 8

Procedure for Boc protection of Isoxazol-5(4*H*)-one (S7) 8

General Procedure B – Synthesis of Isoxazol-5(2*H*)-ones (S8-14) 9

Procedure for Synthesizing Acyl Chlorides 9

General Procedure C – Synthesis of Acetylated Isoxazol-5(2*H*)-ones (1a-t) 9

General Procedure D – Synthesis of Oxazoles (2a-s) 10

Gram Scale Procedure for Synthesis of Oxazole (2t) 10

Hydrolysis of Methyl Ester (3a) 11

Control Study 11

Characterization isoxazol-5(4*H*)-one (S1-S7) 12

Characterization isoxazol-5(2*H*)-one (S8-14) 14

Characterization Acetylated isoxazol-5(2H)-one (1a-t) 17

Characterization of Oxazoles (2a-3a) 24

References 32

Copy of NMR Spectra 33

General Materials and Methods

Substrates, reagents, and solvents were used as purchased without further purification. ^{1}H-NMR spectra were recorded at 25 ˚C using Varian VNMRS 400, 500 and Agilent DD2 500 MHz spectrometers. Deuterated solvents acquired from Sigma-Aldrich and Apollo Scientific were used as supplied. Spectra recorded in ppm using the chosen solvent peak as a reference ($CHCl_3$ δ_H = 7.26 ppm) or DMSO-d_6 (δ_H = 2.50 ppm) Data for ^{1}H-NMR are reported as follows: chemical shift (δ/ppm) (multiplicity, coupling constant (Hz), integration). ^{13}C[^{1}H] NMR spectra were recorded on the same instruments (100 and 125 MHz) and are reported relative to $CHCl_3$ (77.16 ppm) or DMSO-d_6 (39.5 ppm). Multiplicities are reported as follows: s = singlet, d = doublet, t = triplet, q = quartet, m = multiplicity (C, CH, CH_2, CH_3). Structural assignments were made with additional information from gHSQC, experiments.

IR spectra were obtained by use of a Platinum spectrometer (near, ATR sampling, Bruker, Billercia, MA, USA) with intensities of the characteristic signals as reported as weak (w, <20% of tallest signal), medium (m, 21-70% of tallest signal) or strong (s, >71% of tallest signal).

High-resolution mass spectrometry was performed using the indicated techniques on a micromass LCT orthogonal time of flight mass spectrometer and quadropole time-of-flight mass spectrometer with leucine-enkephalin (Tyr-Gly-Phe-Leu) as internal lock mass.

Melting points were recorded on Stuart SMP10 melting point apparatus.

Flow reactions were performed using a Vapourtec E-Series UV-150 photoflow reactor, containing a high-power LED (365 nm) or medium pressure mercury lamp with a reactor coil of 10 mL volume (FEP tubing). LED spot modules from Signify LEDs (330, 308 and 280 nm) were utilized.

TLC was performed on Merck pre-coated Silica gel 60 F254 aluminum plates with realization by UV irradiation at 254nm, $KMnO_4$. Flash chromatography was performed using Macherey-Nagel silica gel 60 M, with a particle range of 0.04 - 0.063 mm.

The photochemistry spot modules used in this study were provided by Signify (https://www.signify.com/global/innovation/photochemistry) and further details can be found via this weblink: specification sheet. The relevant product codes are PS-28x20-R-265, PS-28x20-R-280, PS-28x20-R-308 and PS-28x20-R-330.

The glass chip was purchased from Uniqsis: Uniqsis Products and Expertise - Asynt

Picture of Set-up

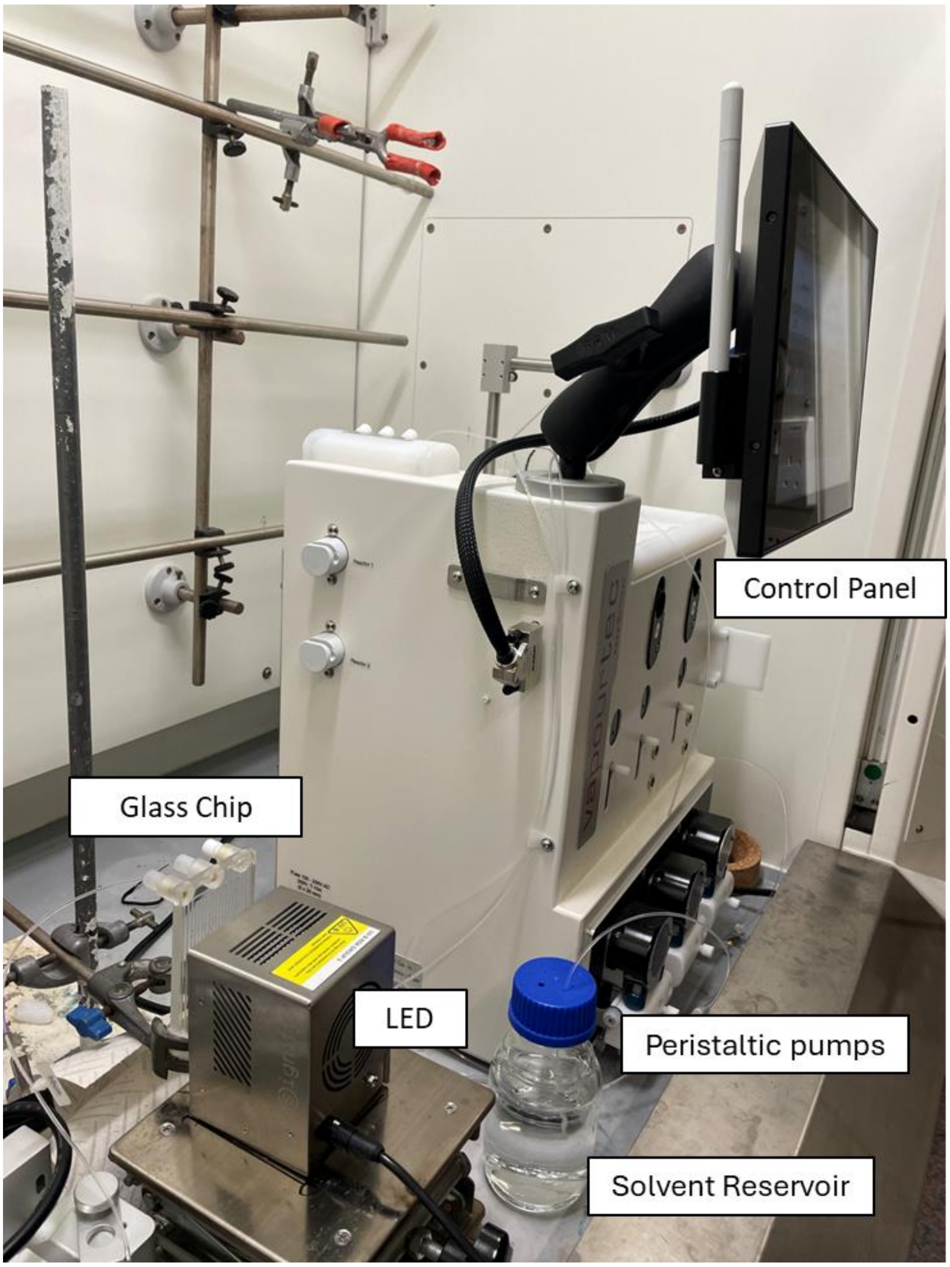

For all reactions the distance of the LED module to chip reactor was measured to be 20 mm.

Reflectivity of Aluminum Sheet

Aluminum sheet thickness: 0.3 mm

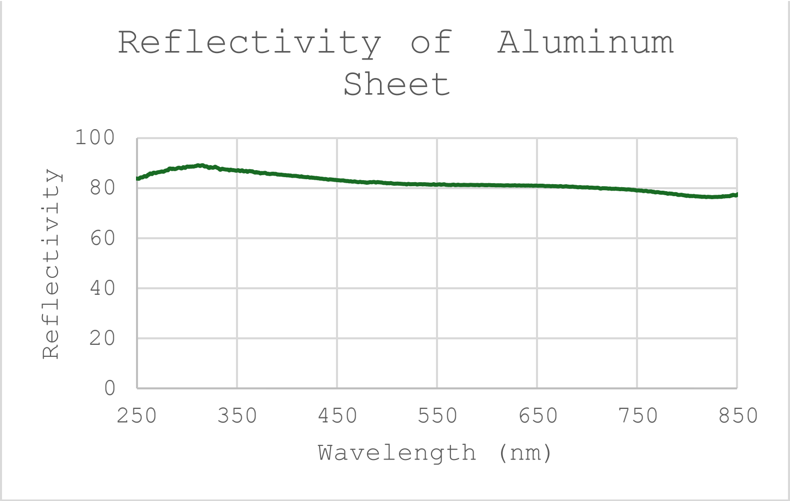

Image of Reflective Material Set-up [The reflective material was clamped (0.5 cm) behind the glass plate]

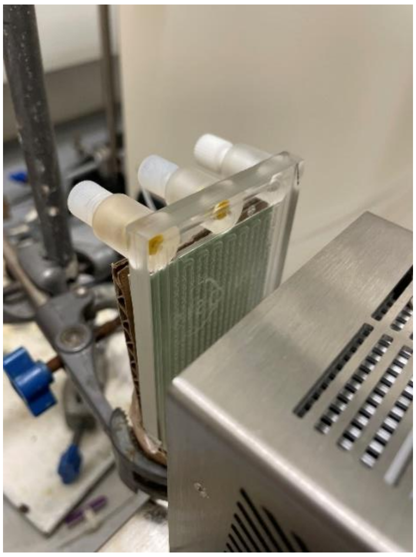

Effect of dimming on LED modules used:

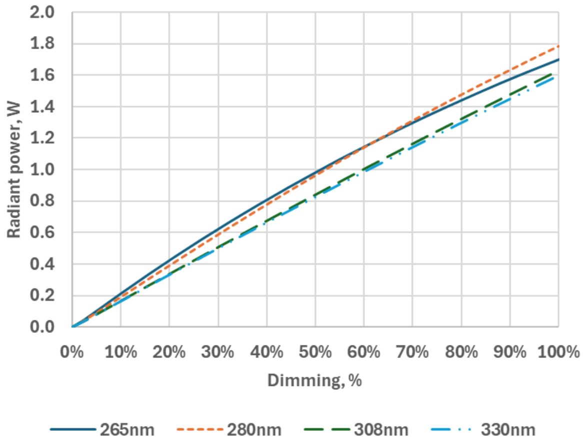

UV-Vis Absorption

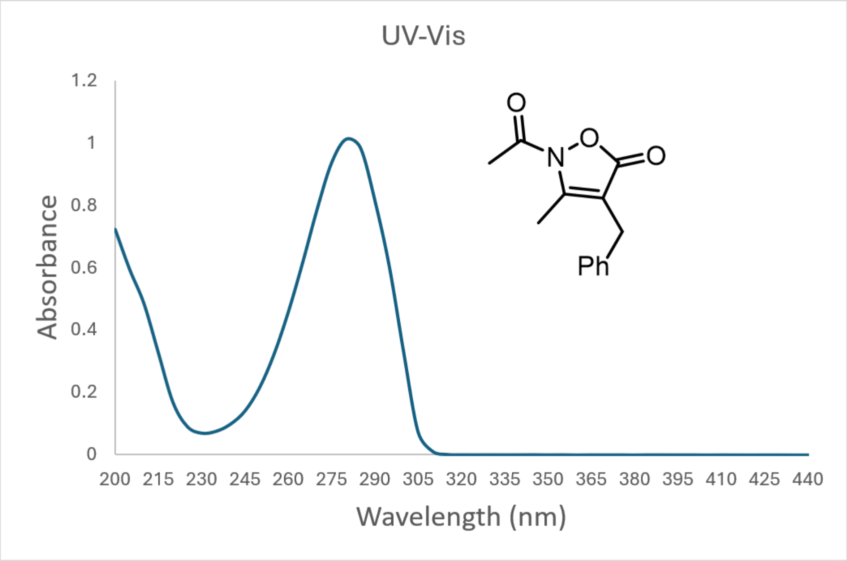

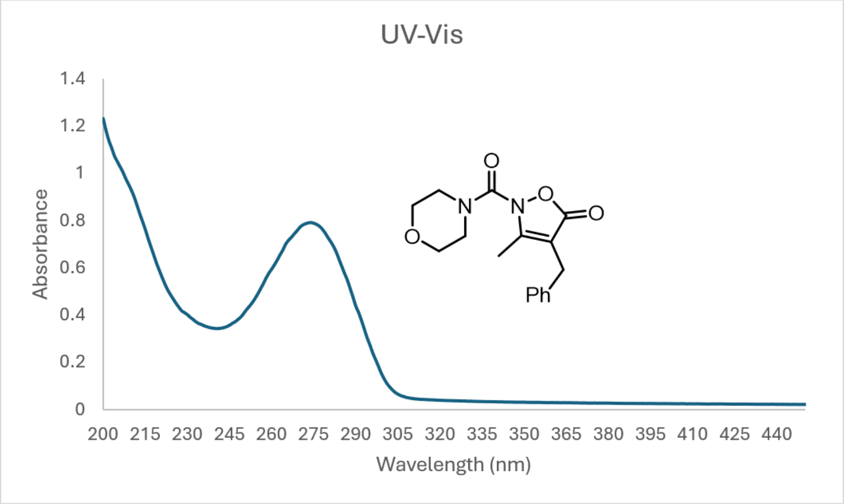

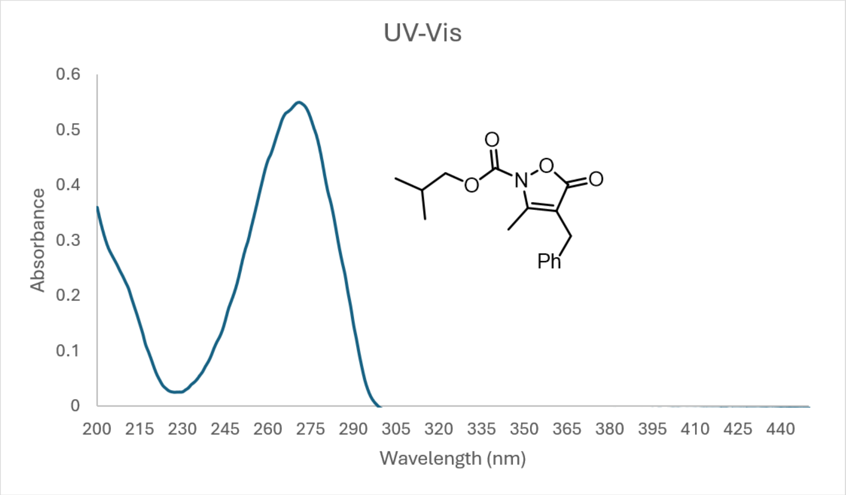

UV Emission of LED Light Sources Studied:

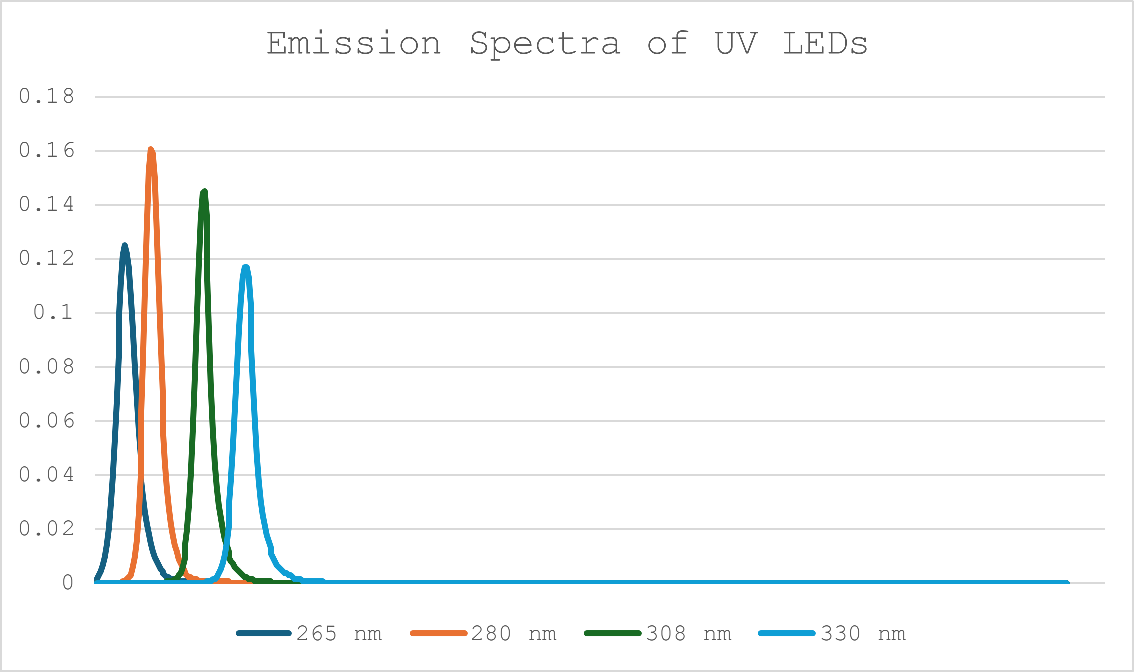

Comparative Emission Spectrum of a Medium-Pressure Hg-Lamp:

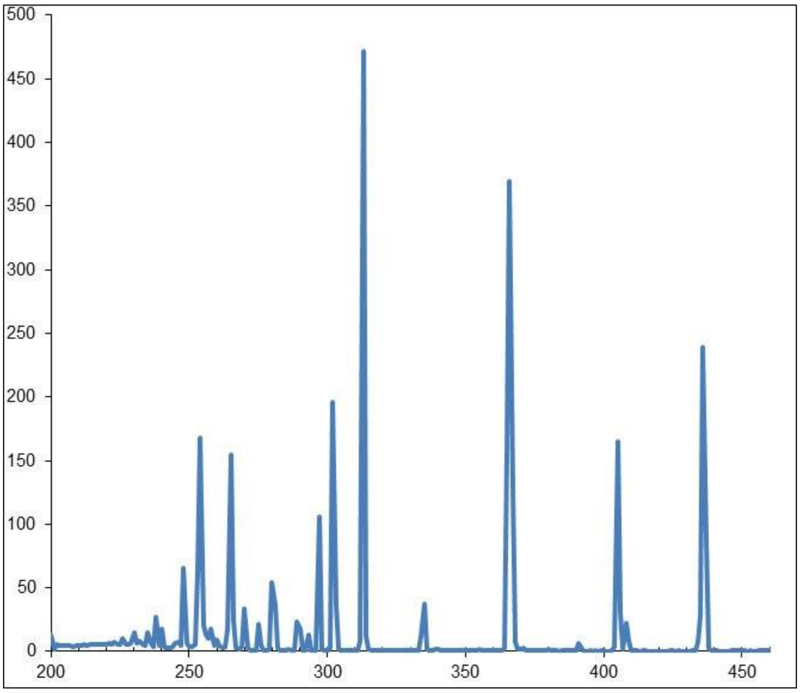

Experimental Procedures

General Procedure A – Synthesis of Isoxazol-5(4*H*)-ones (S1-6)

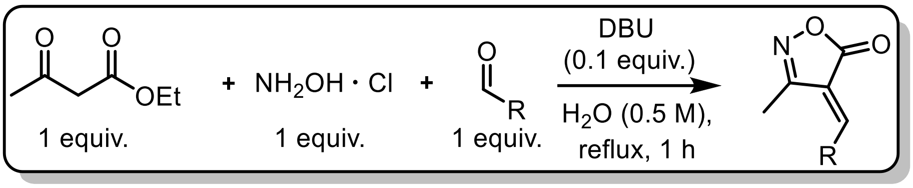

Following a modified reported procedure.[1] Ethyl acetoacetate (130 mg, 1 mmol, 1 equiv.), hydroxylamine hydrochloride (65 mg, 1 mmol, 1 equiv.) and the appropriate aldehyde (1 mmol, 1 equiv.) were dissolved in H_2O (2mL, 0.5 M). 1,8-Diazabicyclo[5.4.0]undec-7-ene (15 mg, 0.1 mmol, 0.1 equiv.) was added and the reaction mixture was heated to reflux by a heating mantle for one hour. The reaction cooled to room temperature and the solid was filtered. The solid was recrystallized in EtOH and a yellow solid was collected by vacuum filtration.

Procedure for Boc protection of Isoxazol-5(4*H*)-one (S7)

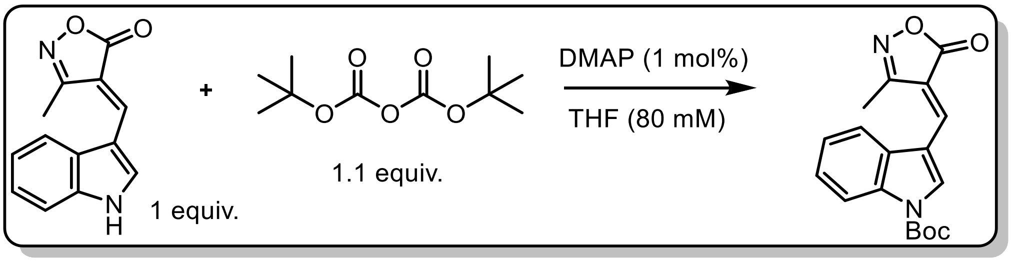

Following a modified reported procedure.[2] To a stirred solution of (*E*)-4-((1*H*-Indol-2-yl)methylene)-3-methylisoxazol-5(4*H*)-one (4.71 g, 20.8 mmol, 1 equiv.) and DMAP (25 mg, 1 mol%) in THF (260 mL, 80 mM), di-tert-butyl carbonate (5.00 g, 22.9 mmol, 1.1 equiv.) was added and the reaction mixture was stirred for 2 h. The reaction mixture was transferred to a separatory funnel, and 1 M HCL was added. The aqueous layer was extracted with DCM 3 times. The combined organic layers were washed with brine and dried with Na_2SO_4. The solvent was evaporated in vacuo to afford the crude product which required no further purification.

General Procedure B – Synthesis of Isoxazol-5(2*H*)-ones (S8-14)

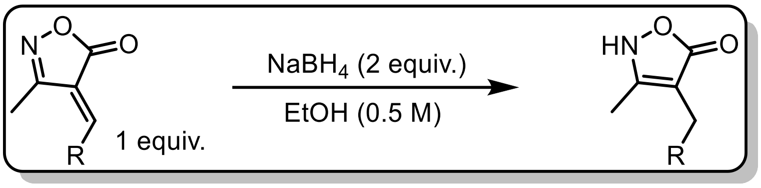

Following a reported procedure.[3] The isoxazol-5(2*H*)-one (1 mmol 1 equiv.) was dissolved in EtOH (2 mL, 0.5 M) and the reaction was cooled to 0 °C using an ice bath. $NaBH_4$ (76 mg, 2 mmol, 2 equiv.) was slowly added over a period of 10 minutes. The reaction was then allowed to warm to room temperature and stirred for one hour. The reaction mixture was transferred to a separatory funnel and quenched with 1 M HCL (the product will precipitate as a white solid). The aqueous layer was extracted with DCM 3 times. The combined organic layers were washed with brine and dried with Na_2SO_4. The solvent was evaporated in vacuo to afford the crude product which required no further purification.

Procedure for Synthesizing Acyl Chlorides

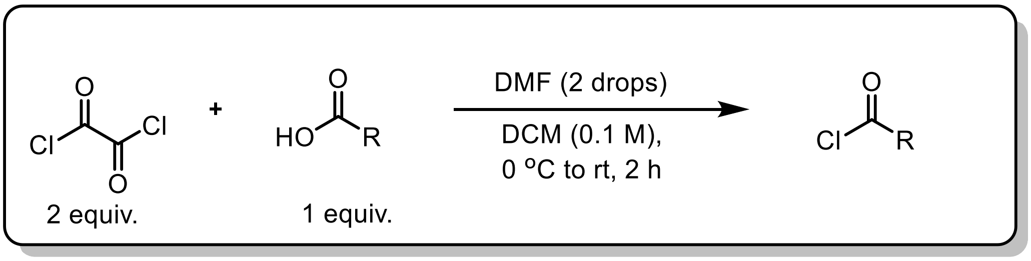

Unless commercially available, a modified reported procedure was followed.[4] The acid (1 mmol, 1 equiv.) was dissolved in DCM (10 mL, 0.1 M) and cooled to 0 °C. Oxalyl chloride (254 mg, 2 mmol, 2 equiv.) was added dropwise to the reaction mixture. DMF (2 drops) was added, and the reaction mixture was allowed to warm to room temperature and stir for 2 h. The solvent was evaporated in vacuo to afford the crude product which required no further purification.

General Procedure C – Synthesis of Acetylated Isoxazol-5(2*H*)-ones (1a-t)

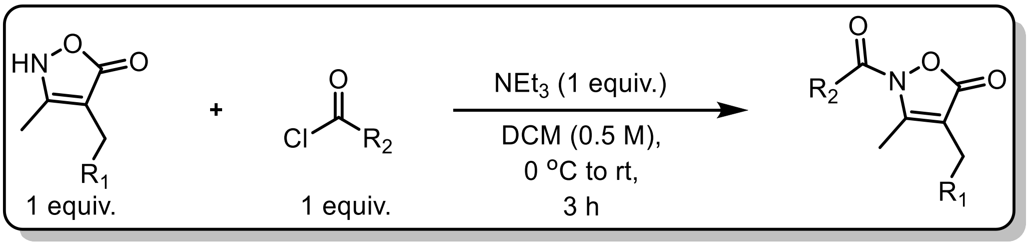

Following a modified previously reported procedure.[5] The isoxazol-5(2*H*)-one (1 mmol, 1 equiv.) was dissolved in DCM (2 mL, 0.5 M) and cooled to 0 °C, NEt_3 (101 mg, 1 mmol, 1 equiv.) was added. The acyl chloride (1 mmol, 1 equiv.) was added dropwise to the reaction mixture over a period of 10 minutes maintaining the 0 °C. After,

the reaction was allowed to warm to room temperature and stirred for 3 hours. The reaction mixture was quenched with H_2O and the aqueous layer was extracted with DCM 3 times. The combined organic layers were washed with brine and dried with Na_2SO_4. The solvent was evaporated in vacuo to afford the crude product which required further purification by SiO_2 chromatography (C-Hex/EtOAc).

General Procedure D – Synthesis of Oxazoles (2a-s)

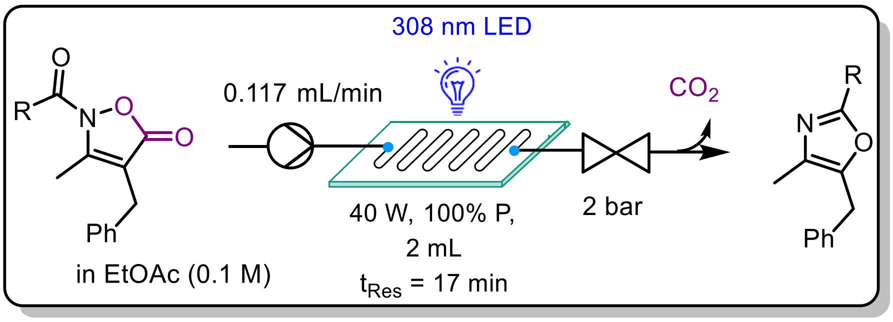

The solvent stream was set to a flow rate of 0.117 mL/min achieving a steady back pressure of 2 bar. The 308 nm LED module was turned on. The acyl isoxazolone (1 mmol) was dissolved in EtOAc (10 mL, 0.1 M) and was passed through the glass microplate. The crude solution was collected in a RBF and the solvent was evaporated in vacuo to afford the crude product which required further purification by SiO_2 chromatography (C-Hex/EtOAc).

Gram Scale Procedure for Synthesis of Oxazole (2t)

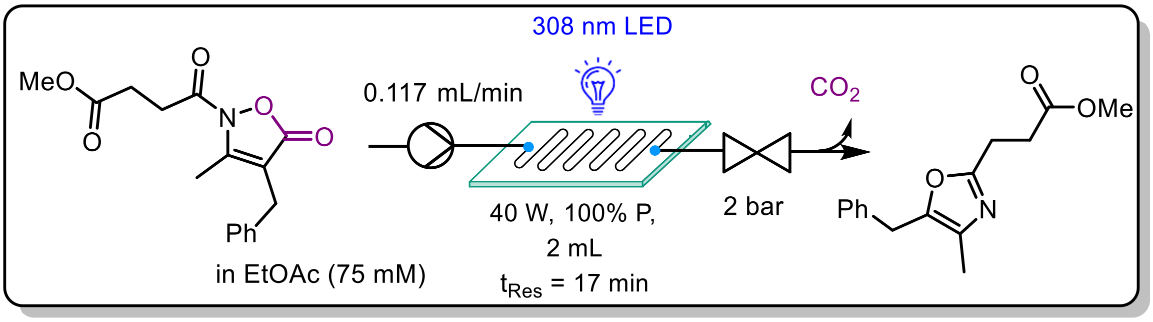

The solvent stream was set to a flow rate of 0.117 mL/min achieving a steady back pressure of 2 bar. The 308 nm LED module was turned on. Methyl 4-(4-benzyl-3-methyl-5-oxoisoxazol-2(5H)-yl)-4-oxobutanoate (2.3 g, 7.58 mmol) was dissolved in EtOAc (101 mL, 0.75 mM) and was passed through the glass microplate. The crude solution was collected in a RBF and the solvent was evaporated in vacuo to afford the crude product which required further purification by SiO_2 chromatography (C-Hex/EtOAc).

Hydrolysis of Methyl Ester (3a)

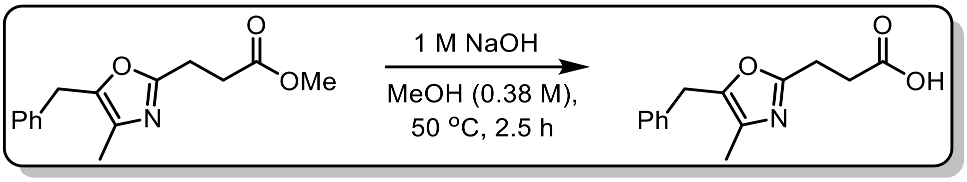

Following a published literature procedure.[6] To a solution of methyl ester (0.294 mg, 1.14 mmol, 1 equiv.) in MeOH (2.98 mL, 0.38 M) was added 1M NaOH (12 mL, 12.08 mmol, 10.6 equiv.). The mixture was stirred at 50 °C by a heating mantle for 2.5 hours. After cooling to room temperature, the reaction was diluted with 1M HCl. The aqueous layer was extracted with DCM 3 times. The combined organic layers were washed with brine and dried with Na_2SO_4. The solvent was evaporated in vacuo to afford the product which required no further purification.

Control Study

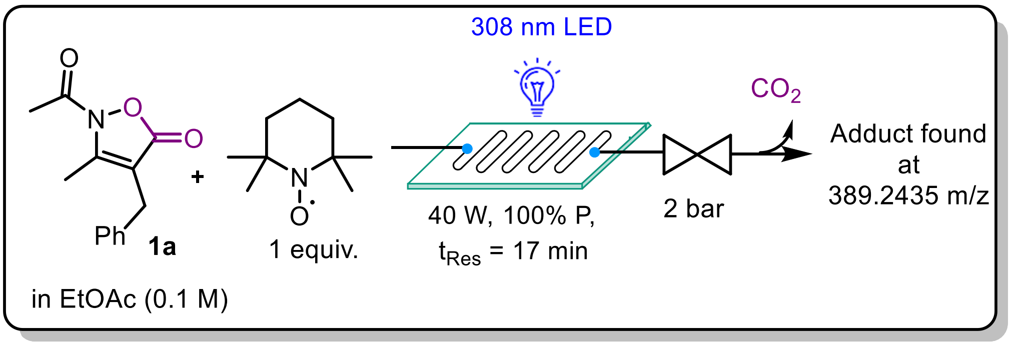

2-Acetyl-4-benzyl-3-methylisoxazol-5(2*H*)-one (231 mg, 1 mmol, 1 equiv.) was dissolved in EtOAc (10 mL, 0.1 M) and 2,2,6,6-tetramethylpiperdin-1-yl)oxyl (156 mg, 1 mmol, 1 equiv.) was pumped through the glass microplate at a flow rate of 0.117 mL/min equipped with the 308 nm LED module. The crude solution was collected in a RBF and the solvent was evaporated in vacuo to afford the crude product which was analyzed by HRMS.

Characterization isoxazol-5(4*H*)-one (S1-S7)

(*E*)-4-Benzylidene-3-methylisoxazol-5(4*H*)-one (S1)

Synthesized via Procedure A

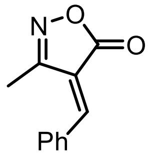

Chemical Formula: $C_{11}H_9NO_2$
Exact Mass: 187.0633

Yield: 33% (2.87 g, 15.3 mmol)

Appearance: Yellow solid

^{1}H-NMR (400 MHz, $CDCl_3$) δ/ppm 8.23 – 8.14 (m, 2H), 7.48 – 7.39 (m, 1H), 7.37 – 7.31 (m, 2H), 7.26 (s, 1H), 2.14 (s, 3H). **^{13}C-NMR (100 MHz, $CDCl_3$)** 167.8 (C), 161.0 (C), 149.8 (CH), 133.9 (C), 133.7 (2CH), 132.1 (C), 128.9 (2CH), 119.6 (C), 11.6 (CH_3). **IR (neat)** ν/cm^{-1}: 2980 (m), 1731 (s), 1618 (s), 1592 (m), 1381 (m), 1112 (m), 955 (m), 755 (s), 593 (w). **HRMS (TOF ES+) m/z:** [M + H]$^+$ Calcd for $C_{11}H_{10}NO_2$ 188.0706; Found 188.0708. In agreement with previously reported. [1]

(*E*)-3-Methyl-4-(thiophen-2-ylmethylene)isoxazol-5(4*H*)-one (S2)

Synthesized via Procedure A

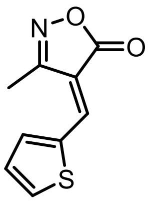

Chemical Formula: $C_9H_7NO_2S$
Exact Mass: 193.0197

Yield: 65% (5.85 g, 30.2 mmol)

Appearance: Yellow solid

^{1}H-NMR (400 MHz, $CDCl_3$) δ/ppm 8.12 (d, *J* = 5.8 Hz, 1H), 7.95 (s, 1H), 7.60 (s, 1H), 7.28 (dd, *J* = 5.0, 3.9 Hz, 1H), 2.30 (s, 3H). **^{13}C-NMR (100 MHz, $CDCl_3$)** 168.5 (C), 160.4 (C), 141.2 (CH), 139.3 (CH), 138.9 (CH), 136.4 (C), 128.8 (CH), 114.6 (C), 11.3 (CH_3). **IR (neat)** ν/cm^{-1}: 2961 (w), 1746 (s), 1699 (s), 1622 (s), 1379 (m), 1174 (w), 1080 (w), 977 (s), 748 (w). **HRMS (TOF ES+) m/z:** [M + H]$^+$ Calcd for $C_9H_8NO_2S$ 194.0270; Found 194.0272. In agreement with previously reported. [7]

(*E*)-4-((1*H*-Indol-2-yl)methylene)-3-methylisoxazol-5(4*H*)-one (S3)

Synthesized via Procedure A

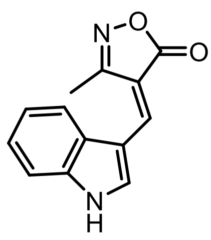

Chemical Formula: $C_{13}H_{10}N_2O_2$
Exact Mass: 226.0742

Yield: 45% (4.71 g, 20.8 mmol)

Appearance: Yellow solid

^{1}H-NMR (400 MHz, DMSO) δ/ppm 12.78 (s, 1H), 9.50 (s, 1H) 8.16 (s, 1H), 8.15 – 8.11 (m, 1H), 7.63 – 7.53 (m, 1H), 7.34 – 7.29 (m, 2H), 2.33 (s, 3H). **^{13}C-NMR (100 MHz, DMSO)** δ/ppm 170.4 (C), 161.7 (C), 140.4 (CH), 138.5 (CH), 136.4 (C), 128.0 (C), 123.9 (CH), 122.5 (CH), 118.8 (CH), 113.1 (CH), 112.7 (C), 108.8 (C), 11.2 (CH_3). **IR (neat)** ν/cm^{-1}: 2980 (s), 1752 (m), 1713 (m), 1444 (s), 1295 (s), 974 (s), 775 (s). 557 (w). **HRMS (TOF ES+) m/z:** [M + H]$^+$ Calcd for $C_{13}H_{11}N_2O_2$ 227.0815; Found 227.0816. In agreement with previously reported. [8]

(*E*)-4-(2-Hydroxybenzylidene)-3-methylisoxazol-5(4*H*)-one (S4)

Synthesized via Procedure A

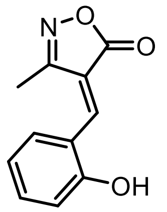

Chemical Formula: $C_{11}H_9NO_3$
Exact Mass: 203.0582

Yield: 45% (4.71 g, 20.8 mmol)

Appearance: Yellow solid

^{1}H-NMR (500 MHz, DMSO) δ/ppm 10.99 (s, 1H), 8.73 (dd, *J* = 8.1, 1.7 Hz, 1H), 8.09 (s, 1H), 7.49 (ddd, *J* = 8.4, 7.2, 1.7 Hz, 1H), 7.03 – 6.99 (m, 1H), 6.96 – 6.91 (m, 1H), 2.26 (s, 3H). **^{13}C-NMR (125 MHz, DMSO)** 168.3 (C), 162.1 (C), 159.6 (C), 145.0 (CH), 136.7 (CH), 132.3 (CH), 119.5 (C), 119.1 (CH), 116.5 (C), 116.1 (CH), 11.2 (CH_3). **IR (neat)** ν/cm^{-1}: **HRMS (TOF ES+) m/z:** $[M + H]^+$ Calcd for $C_{11}H_{10}NO_3$ 204.0655; Found 204.0657. In agreement with previously reported.[7]

(*E*)-4-(4-Bromobenzylidene)-3-methylisoxazol-5(4*H*)-one (S5)

Synthesized via Procedure A

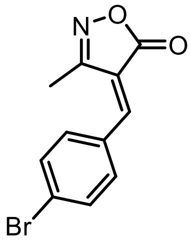

Chemical Formula: $C_{11}H_8BrNO_2$
Exact Mass: 264.9738

Yield: 45% (4.71 g, 20.8 mmol)

Appearance: Yellow solid

^{1}H-NMR (400 MHz, $CDCl_3$) δ/ppm 8.27 – 8.16 (m, 2H), 7.63 (d, *J* = 8.6 Hz, 2H), 7.35 (s, 1H), 2.29 (s, 3H). **^{13}C-NMR (100 MHz, $CDCl_3$)** 167.9 (C), 161.1 (C), 148.3 (CH), 135.1 (2CH), 132.5 (2CH), 131.2 (C), 129.5 (C), 120.4 (C), 11.7 (CH_3).**IR (neat)** ν/cm^{-1}: 2961 (w), 1746 (s), 1700 (s), 1621 (s), 1379 (m), 1131 (w), 1011 (s), 823 (w), 703 (s). **HRMS (TOF ES+) m/z:** $[M + H]^+$ Calcd for $C_{11}H_9BrNO_2$ 265.9812; Found 265.9812**.** In agreement with previously reported.[9]

(*E*)-4-Benzylidene-3-cyclopropylisoxazol-5(4*H*)-one (S6)

Synthesized via Procedure A

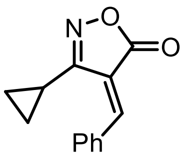

Chemical Formula: $C_{13}H_{11}NO_2$
Exact Mass: 213.0790

Yield: 45% (4.71 g, 20.8 mmol)

Appearance: Yellow solid

^{1}H-NMR (500 MHz, $CDCl_3$) δ/ppm 8.39 – 8.35 (m, 2H), 7.70 (s, 1H), 7.61 – 7.56 (m, 1H), 7.56 – 7.47 (m, 2H), 1.81 – 1.74 (m, 1H), 1.12 – 1.03 (m, 4H). **^{13}C-NMR (125 MHz, $CDCl_3$)** 168.2 (C), 165.1 (C), 149.7 (CH), 133.8 (CH), 133.7 (2CH), 132.3 (C), 128.9 (2CH), 119.8 (CH_2), 6.4 ($2CH_2$), 6.1 (CH). **IR (neat)** ν/cm^{-1}: 2980 (m), 1726 (s), 1619 (s), 1593 (s), 1452 (s), 1389 (m), 1118 (s), 824 (w), 660 (s). **HRMS (TOF ES+) m/z:** $[M + H]^+$ Calcd for $C_{13}H_{12}NO_2$ 214.0863; Found 214.0862**.** In agreement with previously reported. [10]

tert-Butyl (E)-2-((3-methyl-5-oxoisoxazol-4(5H)-ylidene)methyl)-1H-indole-1-carboxylate (S7)

Synthesized by Boc protection of indole analog.

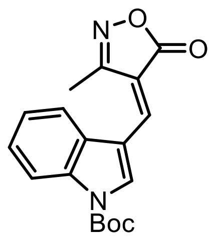

Chemical Formula: $C_{18}H_{18}N_2O_4$
Exact Mass: 326.1267

Yield: 99% (4.71 g, 20.8 mmol)

Appearance: Yellow solid

^{1}H-NMR (500 MHz, $CDCl_3$) δ/ppm 9.87 (s, 1H), 8.30 (dt, *J* = 8.1, 0.9 Hz, 1H), 7.82 – 7.77 (m, 1H), 7.73 (s, 1H), 7.46 (ddd, *J* = 8.2, 7.2, 1.5 Hz, 1H), 7.42 (td, *J* = 7.5, 1.3 Hz, 1H), 2.37 (s, 3H), 1.73 (s, 9H). **^{13}C-NMR (125 MHz, $CDCl_3$)** δ/ppm 169.4 (C), 160.5 (C), 148.4 (C), 136.8 (CH), 136.8 (CH), 135.3 (C), 129.1 (C), 125.9 (CH), 124.2 (CH), 117.6 (CH), 116.2 (C), 115.9 (CH), 114.3 (C), 85.9 (C), 28.0 ($3CH_3$), 11.5 (CH_3). **IR (neat)** ν/cm^{-1}: 2980 (s), 1667 (m), 1452 (m), 1259 (m), 1081 (s), 869 (w), 744 (s), 581 (w). **HRMS (TOF ES+) m/z:** $[M + H]^+$ Calcd for $C_{18}H_{19}N_2O_4$ 327.1339; Found 327.1339.

Characterization isoxazol-5(2*H*)-one (S8-14)

4-Benzyl-3-methylisoxazol-5(2*H*)-one (S8)

Synthesized via Procedure B

Note – Tautomerizes in solution

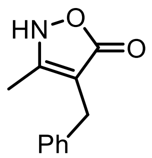

Chemical Formula: $C_{11}H_{11}NO_2$
Exact Mass: 189.0790

Yield: 95% (2.88 g, 15.2 mmol)

Appearance: White solid

^{1}H-NMR (500 MHz, $CDCl_3$) δ/ppm 7.35 – 7.26 (m, 5H), 7.26 –7.23 – 7.12 (m, 5H), 3.60 (ddd, *J* = 6.9, 5.5, 0.8 Hz, 2H), 3.55 (s, 2H), 3.21 (qd, *J* = 14.6, 6.2 Hz, 2H), 2.06 (s, 3H), 1.98 (d, *J* = 0.8 Hz, 3H). **^{13}C-NMR (125 MHz, $CDCl_3$)** δ/ppm 177.6 (C), 173.9 (C), 165.9 (C), 161.7 (C), 138.8 (C), 135.0 (C), 129.0 (2CH), 128.5 (2CH), 128.5 (2CH), 128.1 (2CH), 127.7 (CH), 126.3 (CH), 100.3 (C), 48.5 (CH), 33.4 (CH_2), 27.5 (CH_2), 14.1 (CH_3), 10.9 (CH_3). **IR (neat)** ν/cm^{-1}: 2979 (w), 2735 (w), 1676 (s), 1584 (m), 1492 (w), 1394 (w), 1073, (w), 921 (w), 756 (s). **HRMS (TOF ES+) m/z:** $[M + H]^+$ Calcd for $C_{11}H_{12}NO_2$ 190.0863; Found 190.0865. In agreement with previously reported. [11]

3-Methyl-4-(thiophen-2-ylmethyl)isoxazol-5(2*H*)-one (S9)

Synthesized via Procedure B

Note – Tautomerizes in solution

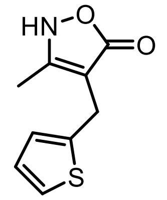

Chemical Formula: $C_9H_9NO_2S$
Exact Mass: 195.0354

Yield: 99% (3.13 g, 16.0 mmol)

Appearance: White solid

^{1}H-NMR (400 MHz, CDCl$_3$) δ/ppm 10.13 (s, 1H), 7.17 (dd, *J* = 5.2, 1.2 Hz, 1H), 7.13 – 7.07 (m, 2H), 6.92 (dd, *J* = 5.2, 3.5 Hz, 1H), 6.87 (dd, *J* = 5.1, 3.4 Hz, 1H), 6.80 (dq, *J* = 3.4, 1.1 Hz, 1H), 3.71 (s, 2H), 3.60 (t, *J* = 5.6 Hz, 1H), 3.50 – 3.36 (m, 2H), 2.10 (s, 3H), 2.08 – 2.06 (m, 3H).**^{13}C-NMR (100 MHz, CDCl$_3$)** δ/ppm 177.2 (C), 173.6 (C), 165.7 (C), 161.4 (C), 141.9 (C), 136.2 (C), 127.3 (CH), 126.8 (CH), 126.7 (CH), 125.0 (CH), 124.7 (CH), 123.7 (CH), 97.7 (C), 48.8 (CH), 27.2 (CH$_2$), 21.8 (CH$_2$), 13.8 (CH$_3$), 10.5 (CH$_3$). **IR (neat)** ν/cm^{-1}: 3391 (w), 2980 (w), 1749 (s), 1696 (s), 1377 (m), 1273 (m), 1097 (w), 974 (w), 705 (m). **HRMS (TOF ES+) m/z:** [M + H]$^+$ Calcd for $C_9H_{10}NO_2S$ 196.0427; Found 196.0428.

4-(4-Bromobenzyl)-3-methylisoxazol-5(2*H*)-one (S10)

Synthesized via Procedure B

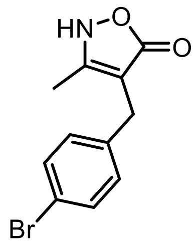

Chemical Formula: $C_{11}H_{10}BrNO_2$
Exact Mass: 266.9895

Yield: 90% (1.90 g, 7.08 mmol)

Appearance: White solid

^{1}H-NMR (500 MHz, DMSO) δ/ppm 12.02 (s, 1H), 7.47 – 7.44 (m, 2H), 7.19 – 7.14 (m, 2H), 3.43 (s, 2H), 2.08 (s, 3H). **^{13}C-NMR (125 MHz, DMSO)** δ/ppm 161.5 (C), 139.4 (C), 131.2 (2CH), 130.2 (2CH), 119.1 (C), 26.2 (CH$_2$), 10.4 (CH$_2$). **IR (neat)** ν/cm^{-1}: 2980 (m), 1731 (s), 1619 (s), 1452 (w), 1351 (w), 1112 (m), 1021 (w), 878 (s), 755 (s). **HRMS (TOF ES+) m/z:** [M + H]$^+$ Calcd for $C_{11}H_{11}BrNO_2$ 267.9968; Found 267.9969.

4-Benzyl-3-cyclopropylisoxazol-5(2*H*)-one (S11)

Synthesized via Procedure B

Note – Tautomerizes in solution

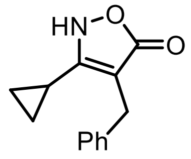

Chemical Formula: $C_{13}H_{13}NO_2$
Exact Mass: 215.0946

Yield: 79% (650 mg, 1.98 mmol)

Appearance: White solid

^{1}H-NMR (400 MHz, CDCl$_3$) δ/ppm 10.03 (s, 1H), 7.34 – 7.28 (m, 2H),7.27-7.23 (m, 7H), 7.22 – 7.16 (m, 1H), 3.70 (ddd, *J* = 6.5, 5.4, 0.9 Hz, 1H), 3.64 (s, 2H), 3.35 – 3.21 (m, 2H), 1.81 – 1.70 (m, 1H), 1.38 – 1.32 (m, 1H), 1.08 – 1.04 (m, 1H), 0.99 – 0.94 (m, 6H), 0.93 – 0.88 (m, 1H). **^{13}C-NMR (100 MHz, CDCl$_3$)** δ/ppm 177.7 (C), 174.2 (C), 170.9 (C), 166.9 (C), 139.1 (C), 135.1 (C), 128.7 (2CH), 128.7 (2CH), 128.4 (2CH), 128.1 (2CH), 127.5 (CH), 126.1 (CH), 100.4 (C), 44.8 (CH), 33.7 (CH$_2$), 27.4 (CH$_2$), 9.4 (CH$_2$), 8.8 (CH$_2$), 8.6 (CH), 7.4

(CH), 7.3 (CH_2), 7.2 (CH_2). **IR (neat)** v/cm^{-1}: 3061 (w), 1687 (s), 1603 (s), 1493 (w), 1452 (w), 1097 (w), 861 (w), 728 (s), 700 (s). **HRMS (TOF ES+) m/z:** $[M + H]^+$ Calcd for $C_{13}H_{14}NO_2$ 216.1019; Found 216.1020.

4-((1*H*-Indol-2-yl)methyl)-3-methylisoxazol-5(2*H*)-one (S12)

Synthesized via Procedure B

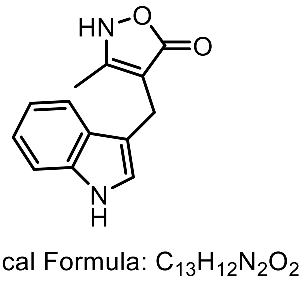

Chemical Formula: $C_{13}H_{12}N_2O_2$
Exact Mass: 228.0899

Yield: 90% (2.53 g, 11.1 mmol)

Appearance: White solid

^{1}H-NMR (400 MHz, DMSO) δ/ppm 11.8 (s, 1H), 10.78 (s, 1H), 7.56 (ddt, *J* = 7.9, 1.4, 0.7 Hz, 1H), 7.33 (dt, *J* = 8.1, 0.9 Hz, 1H), 7.16 – 7.02 (m, 2H), 6.96 (ddd, *J* = 8.0, 7.0, 1.1 Hz, 1H), 3.54 (s, 2H), 2.05 (s, 3H). **^{13}C-NMR (100 MHz, DMSO)** δ/ppm 177.2 (C), 161.5 (C), 136.4 (C), 126.7 (C), 122.7 (CH), 121.0 (CH), 118.5 (CH), 118.3 (CH), 112.5 (C), 111.3 (CH), 97.4 (C), 17.0 (CH_2), 10.4 (CH_3). **IR (neat)** v/cm^{-1}: 2980 (s), 1663 (m), 1588 (m), 1392 (w), 1148 (w), 1057 (w), 956 (w), 734 (m), 517 (w). **HRMS (TOF ES+) m/z:** $[M + H]^+$ Calcd for $C_{13}H_{13}N_2O_2$ 229.0972; Found 229.0972 ($M+H^+$).

tert-Butyl 2-((3-methyl-5-oxo-2,5-dihydroisoxazol-4-yl)methyl)-1H-indole-1-carboxylate (S13)

Synthesized via Procedure B

Note – Tautomerizes in solution

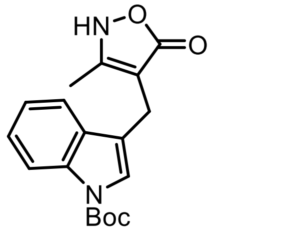

Chemical Formula: $C_{18}H_{20}N_2O_4$
Exact Mass: 328.1423

Yield: 79% (650 mg, 1.98 mmol)

Appearance: Yellow solid

^{1}H-NMR (400 MHz, $CDCl_3$) δ/ppm 8.13 (d, *J* = 8.5 Hz, 1H), 8.06 (s, 1H), 7.57 (dt, *J* = 7.8, 1.0 Hz, 1H), 7.53 (dt, *J* = 7.8, 1.0 Hz, 1H), 7.47 (s, 1H), 7.37 – 7.20 (m, 5H), 3.66 (ddd, *J* = 6.5, 5.5, 1.0 Hz, 1H), 3.60 (d, *J* = 1.3 Hz, 2H), 3.36 – 3.23 (m, 2H), 2.09 (s, 3H), 2.03 (d, *J* = 0.8 Hz, 3H), 1.67 (s, 9H), 1.66 (s, 9H). **^{13}C-NMR (100 MHz, $CDCl_3$)** δ/ppm 177.8 (C), 173.8 (C), 166.3 (C), 162.4 (C), 150.1 (C), 149.6 (C), 135.7 (C), 130.2 (C), 129.6 (C), 125.1 (CH), 124.7 (CH), 123.9 (CH), 123.0 (CH), 123.0 (CH), 122.8 (CH), 122.5 (C), 125.0 (C), 119.3 (CH), 118.5 (CH), 115.6 (CH), 115.4 (CH), 114.4 (C), 99.2 (C), 84.3 (C), 84.0 (C), 47.3 (CH), 28.3 (3CH_3), 28.3 (3CH_3), 23.2 (CH_2), 17.6 (CH_2), 14.2 (CH_3), 11.1 (CH_3). **IR (neat)** v/cm^{-1}: 2979 (w), 1792 (w), 1725 (m), 1451 (m), 1366 (s), 1253 (m), 1081 (m), 908 (w), 731 (m). **HRMS (TOF ES+) m/z:** $[M + H]^+$ Calcd for $C_{18}H_{21}N_2O_4$ 329.1496; Found 329.1495.

4-(2-Hydroxybenzyl)-3-methylisoxazol-5(2*H*)-one (S14)

Synthesized via Procedure B

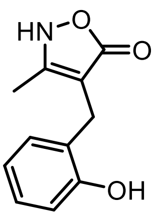

Chemical Formula: $C_{11}H_{11}NO_3$
Exact Mass: 205.0739

Yield: 21% (480 mg, 2.33 mmol)

Appearance: White solid

^{1}H-NMR (500 MHz, DMSO) δ/ppm 11.79 (s, 1H), 9.45 (s, 1H), 7.02 – 6.96 (m, 2H), 6.81 – 6.76 (m, 1H), 6.72 – 6.67 (m, 1H), 3.36 (s, 2H), 2.06 (s, 3H). **^{13}C-NMR (125 MHz, DMSO)** δ/ppm 161.7 (C), 154.8 (C), 129.3 (CH), 127.1 (CH), 125.5 (C), 118.8 (CH), 114.8 (CH), 21.1 (CH_2), 10.5 (CH_2). **IR (neat)** ν/cm^{-1}: 3240 (w), 2715 (w), 1645 (s), 1452 (s), 1359 (s), 1230 (s), 1030 (s), 887 (w), 747 (s), 590 (m). **HRMS (TOF ES+) m/z:** [M + H]$^+$ Calcd for $C_{11}H_{12}NO_3$ 206.0812; Found 206.0813.

Characterization Acetylated isoxazol-5(2H)-one (1a-t)

2-Acetyl-4-benzyl-3-methylisoxazol-5(2*H*)-one (1a)

Synthesized via Procedure C

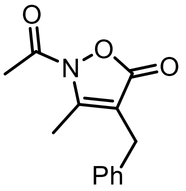

Chemical Formula: $C_{13}H_{13}NO_3$
Exact Mass: 231.0895

Yield: 84% (3.17 g, 13.7 mmol)

Appearance: White solid

^{1}H-NMR (500 MHz, $CDCl_3$) δ/ppm 7.33 – 7.26 (m, 2H), 7.23 (dt, *J* = 8.0, 1.9 Hz, 3H), 3.60 (s, 2H), 2.54 (s, 3H), 2.41 (s, 3H). **^{13}C-NMR (125 MHz, $CDCl_3$)** δ/ppm 166.9 (C), 164.9 (C), 153.7 (C), 137.1 (C), 128.7 (2CH), 128.1 (2CH), 126.7 (CH), 106.3 (C), 27.4 (CH_2), 22.6 (CH_3), 13.7 (CH_3). **IR (neat)** ν/cm^{-1}: 2980 (m), 1754 (m), 1702 (s), 1622 (m), 1408 (m), 1320 (s), 1011 (m), 820 (w), 657 (m). **HRMS (TOF ES+) m/z:** [M + H]$^+$ Calcd for $C_{13}H_{14}NO_3$ 232.0968; Found 232.0970.

4-Benzyl-2-isobutyryl-3-methylisoxazol-5(2*H*)-one (1b)

Synthesized via Procedure C

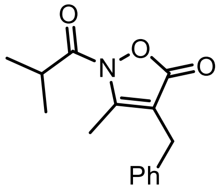

Chemical Formula: $C_{15}H_{17}NO_3$
Exact Mass: 259.1208

Yield: 60% (473 mg, 1.82 mmol)

Appearance: White solid

^{1}H-NMR (500 MHz, $CDCl_3$) δ/ppm 7.32 – 7.28 (m, 2H), 7.26 – 7.20 (m, 3H), 3.61 (s, 2H), 3.21 (hept, *J* = 6.9 Hz, 1H), 2.56 (s, 3H), 1.23 (d, *J* = 6.9 Hz, 6H). **^{13}C-NMR (125 MHz, $CDCl_3$)** δ/ppm 172.0 (C), 167.1 (C), 154.2 (C), 137.9 (C), 128.7 (2CH), 128.2 (2CH), 126.7 (CH), 106.0 (C), 33.1 (CH), 27.5 (CH_2), 18.4 (2CH_3), 14.0 (CH_3)**.** **IR (neat)** ν/cm^{-1}: 2979 (w), 1761 (m), 1704 (s), 1626 (m), 1364 (m), 1162 m(w), 1009 (m), 863 (w), 700 (s). **HRMS (TOF ES+) m/z:** [M + H]$^+$ Calcd for $C_{15}H_{18}NO_3$ 260.1281; Found 260.1268.

4-Benzyl-3-methyl-2-propionylisoxazol-5(2*H*)-one (1c)

Synthesized via Procedure C

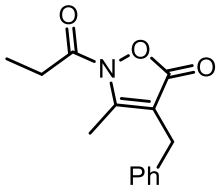

Chemical Formula: $C_{14}H_{15}NO_3$
Exact Mass: 245.1052

Yield: 68% (463 mg, 1.89 mmol)

Appearance: White solid

^{1}H-NMR (500 MHz, CDCl$_3$) δ/ppm 7.32 – 7.27 (m, 2H), 7.25 – 7.19 (m, 3H), 3.60 (s, 2H), 2.75 (q, *J* = 7.4 Hz, 2H), 2.55 (s, 3H), 1.20 (t, *J* = 7.4 Hz, 3H). **^{13}C-NMR (125 MHz, CDCl$_3$)** δ/ppm 168.8 (C), 167.0 (C), 153.9 (C), 137.9 (C), 128.7 (2CH), 128.1 (2CH), 126.7 (CH), 105.9 (C), 28.1 (CH$_2$), 27.4 (CH$_2$), 13.8 (CH$_3$), 7.7 (CH). **IR (neat)** ν/cm^{-1}: 2981 (w), 1747 (m), 1706 (s), 1626 (m), 1371 (s), 1280 (s), 1007 (m), 886 (w), 701 (s). **HRMS (TOF ES+) m/z:** [M + H]$^+$ Calcd for $C_{14}H_{16}NO_3$ 246.1125; Found 246.1127.

4-Benzyl-3-methyl-2-pentanoylisoxazol-5(2*H*)-one (1d)

Synthesized via Procedure C

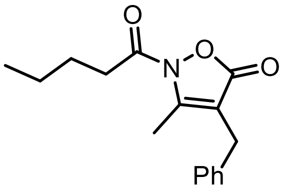

Chemical Formula: $C_{16}H_{19}NO_3$
Exact Mass: 273.1365

Yield: 71% (606 mg, 2.21 mmol)

Appearance: White solid

^{1}H-NMR (500 MHz, CDCl$_3$) δ/ppm 7.31 – 7.27 (m, 2H), 7.25 – 7.19 (m, 3H), 3.60 (s, 2H), 2.72 (t, *J* = 7.4 Hz, 2H), 2.55 (s, 3H), 1.70 – 1.63 (m, 2H), 1.44 – 1.35 (m, 2H), 0.94 (t, *J* = 7.4 Hz, 3H). **^{13}C-NMR (125 MHz, CDCl$_3$)** δ/ppm 168.0 (C), 167.0 (C), 153.9 (C), 137.9 (C), 128.6 (2CH), 128.1 (2CH), 126.6 (CH), 105.9 (C), 34.3 (CH$_2$), 27.4 (CH$_2$), 25.6 (CH$_2$), 22.0 (CH$_2$), 13.8 (CH$_2$), 13.6 (CH$_2$). **IR (neat)** ν/cm^{-1}: 2980 (m), 1746 (m), 1704 (s), 1408 (m), 1351 (m), 1171 (w), 828 (w), 700 (s), 536 (w). **HRMS (TOF ES+) m/z:** [M + H]$^+$ Calcd for $C_{16}H_{20}NO_3$ 274.1438; Found 274.1438.

4-Benzyl-2-(cyclopropanecarbonyl)-3-methylisoxazol-5(2*H*)-one (1e)

Synthesized via Procedure C

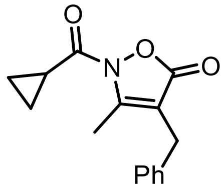

Chemical Formula: $C_{15}H_{15}NO_3$
Exact Mass: 257.1052

Yield: 60% (434 mg, 1.68 mmol)

Appearance: White solid

^{1}H-NMR (500 MHz, CDCl$_3$) δ/ppm 7.32 – 7.28 (m, 2H), 7.26 – 7.20 (m, 3H), 3.62 (s, 2H), 2.53 (s, 3H), 2.40 (tt, *J* = 7.9, 4.6 Hz, 1H), 1.18 (pd, *J* = 4.0, 0.7 Hz, 2H), 1.10 – 1.04 (pd, *J* = 4.0, 0.7 Hz, 2H). **^{13}C-NMR (125 MHz, CDCl$_3$)** δ/ppm 168.8 (C), 167.3 (C), 153.9 (C), 138.0 (C), 128.7 (2CH), 128.11 (2CH), 126.6 (CH), 105.9 (C), 27.4 (CH$_2$), 13.8 (CH$_3$), 12.5 (CH), 10.5 (2CH$_2$). **IR (neat)** ν/cm^{-1}: 2980 (w), 1746 (s), 1625 (m), 1393 (s), 1172 (w), 1031 (s), 921 (w), 703 (s), 576 (w). **HRMS (TOF ES+) m/z:** [M + H]$^+$ Calcd for $C_{15}H_{16}NO_3$ 258.1125; Found 258.1125.

4-Benzyl-2-(cyclobutanecarbonyl)-3-methylisoxazol-5(2*H*)-one (1f)

Synthesized via Procedure C

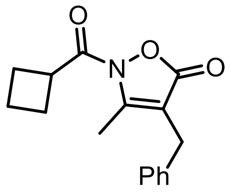

Chemical Formula: $C_{16}H_{17}NO_3$
Exact Mass: 271.1208

Yield: 51% (409 mg, 6.65 mmol)

Appearance: White solid

^{1}H-NMR (500 MHz, $CDCl_3$) δ/ppm 7.32 – 7.27 (m, 2H), 7.24 – 7.21 (m, 3H), 3.69 (td, *J* = 8.6, 1.1 Hz, 1H), 3.59 (s, 2H), 2.56 (s, 3H), 2.44 – 2.25 (m, 4H), 2.12 – 2.00 (m, 1H), 1.97 – 1.85 (m, 1H). **^{13}C-NMR (125 MHz, $CDCl_3$)** δ/ppm 169.5 (C), 167.2 (C), 153.9 (C), 138.0 (C), 128.7 (2CH), 128.1 (2CH), 126.7 (CH), 105.8 (C), 38.2 (CH), 27.4 (CH_2), 24.6 ($2CH_2$), 18.2 (CH), 13.7 (CH_3). **IR (neat)** v/cm^{-1}: 2961 (w), 1746 (s), 1697 (s), 1622 (m), 1285 (m), 1113 (w), 977 (m), 746 (w), 686 (s). **HRMS (TOF ES+) m/z:** $[M + H]^+$ Calcd for $C_{16}H_{18}NO_3$ 272.1281; Found 272.1281.

4-Benzyl-2-(cyclohexanecarbonyl)-3-methylisoxazol-5(2*H*)-one (1g)

Synthesized via Procedure C

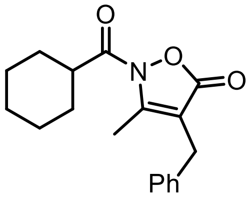

Chemical Formula: $C_{18}H_{21}NO_3$
Exact Mass: 299.1521

Yield: 70% (445 mg, 1.45 mmol)

Appearance: White solid

^{1}H-NMR (500 MHz, $CDCl_3$) δ/ppm 7.34 – 7.27 (m, 2H), 7.26 – 7.20 (m, 3H), 3.60 (s, 2H), 2.96 (tt, *J* = 11.5, 3.4 Hz, 1H), 2.55 (s, 3H), 1.96 – 1.89 (m, 2H), 1.81 (dt, *J* = 13.1, 3.4 Hz, 2H), 1.72 – 1.65 (m, 1H), 1.47 (qd, *J* = 12.5, 3.0 Hz, 2H), 1.39 – 1.30 (m, 2H), 1.30 – 1.18 (m, 1H). **^{13}C-NMR (125 MHz, $CDCl_3$)** δ/ppm 171.2 (C), 167.2 (C), 154.2 (C), 138.0 (C), 128.7 (2CH), 128.1 (2CH), 126.7 (CH), 105.9 (C), 42.5 (CH), 28.4 ($2CH_2$), 27.5 (CH_2), 25.5 (CH_2), 25.2 ($2CH_2$), 14.0 (CH_3). **IR (neat)** v/cm^{-1}: 2980 (w), 1732 (m), 1619 (s), 1380 (m), 11277 (w), 955 (m), 878 (m), 753 (s), 683 (s). **HRMS (TOF ES+) m/z:** $[M + H]^+$ Calcd for $C_{18}H_{22}NO_3$ 300.1594; Found 300.1594.

4-Benzyl-3-methyl-2-(tetrahydro-2H-pyran-4-carbonyl)isoxazol-5(2*H*)-one (1h)

Synthesized via Procedure C

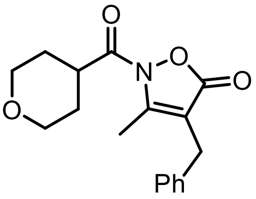

Chemical Formula: $C_{17}H_{19}NO_4$
Exact Mass: 301.1314

Yield: 61% (370 mg, 1.22 mmol)

Appearance: White solid

^{1}H-NMR (400 MHz, $CDCl_3$) δ/ppm 7.33 – 7.27 (m, 2H), 7.25 – 7.19 (m, 3H), 4.08 – 4.02 (m, 1H), 4.02 – 3.99 (m, 1H), 3.60 (s, 2H), 3.52 – 3.43 (m, 2H), 3.29 – 3.15 (m, 1H), 2.56 (s, 3H), 1.83 (m, 4H). **^{13}C-NMR (100 MHz, $CDCl_3$)** δ/ppm 169.1 (C), 166.9 (C), 154.1 (C), 137.8 (C), 128.7 (2CH), 128.1 (2CH), 126.8 (CH), 106.3 (C), 66.9 ($2CH_2$) 39.8 (CH), 27.9 ($2CH_2$), 27.5 (CH_2), 13.9 (CH_3). **IR (neat)** v/cm^{-1}: 2960 (w), 1746 (m), 1700 (s), 1622 (m), 1379 (m), 1122 (w), 1010 (m), 822 (w), 703 (s). **HRMS (TOF ES+) m/z:** $[M + H]^+$ Calcd for $C_{17}H_{20}NO_4$ 302.1387; Found 302.1386.

Methyl 4-(4-benzyl-3-methyl-5-oxo-2,5-dihydroisoxazole-2-carbonyl)bicyclo[2.2.2]octane-1-carboxylate (1i)

Synthesized via Procedure C

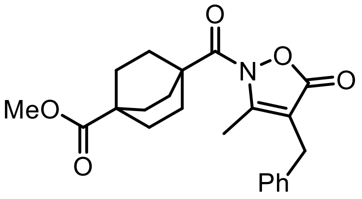

Chemical Formula: $C_{22}H_{25}NO_5$
Exact Mass: 383.1733

Yield: 52% (319 mg, 0.83 mmol)

Appearance: White solid

^{1}H-NMR (400 MHz, $CDCl_3$) δ/ppm 7.33 – 7.26 (m, 2H), 7.28 – 7.18 (m, 3H), 3.66 (s, 3H), 3.60 (s, 2H), 2.52 (s, 3H), 2.04 – 1.98 (m, 4H), 1.92 – 1.81 (m, 8H). **^{13}C-NMR (100 MHz, $CDCl_3$)** δ/ppm 177.5 (C), 171.7 (C), 166.9 (C), 155.2 (C), 138.0 (C), 128.7 (2CH), 128.1 (2CH), 126.7 (CH), 105.2 (C), 51.8 (CH_3), 42.0 (C), 38.5 (C), 27.5 (CH_2), 27.4 ($3CH_2$), 26.5 ($3CH_2$), 14.7 (CH_3). **IR (neat)** ν/cm^{-1}: 2980 (m), 1732 (s), 1619 (s), 1381 (m), 1112 (m), 990 (w), 878 (s), 754 (s), 684 (s). **HRMS (TOF ES+) m/z:** $[M + H]^+$ Calcd for $C_{22}H_{26}NO_5$ 384.1805; Found 384.1806.

4-Benzyl-3-methyl-2-(3-oxocyclobutane-1-carbonyl)isoxazol-5(2*H*)-one (1j)

Synthesized via Procedure C

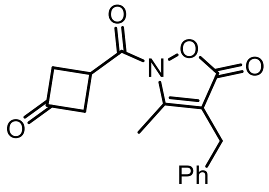

Chemical Formula: $C_{16}H_{15}NO_4$
Exact Mass: 285.1001

Yield: 78% (587 mg, 2.05 mmol)

Appearance: White solid

^{1}H-NMR (400 MHz, $CDCl_3$) δ/ppm 7.34 – 7.25 (m, 2H), 7.25 – 7.17 (m, 3H), 3.84 – 3.72 (m, 1H), 3.60 (s, 2H), 3.54 – 3.42 (m, 2H), 3.42 – 3.28 (m, 2H), 2.58 (s, 3H). **^{13}C-NMR (100 MHz, $CDCl_3$)** δ/ppm 202.2 (C), 167.3 (C), 166.5 (C), 153.8 (C), 137.5 (C), 128.7 (2CH), 128.1 (2CH), 126.7 (C), 106.9 (C), 50.6 ($2CH_2$), 27.4 (CH), 27.4 (CH_2), 13.7 (CH_3). **IR (neat)** ν/cm^{-1}: 2961 (w), 1747 (s), 1697 (s), 1623 (m), 1371 (m), 1174 (w), 1009 (m), 746 (m), 569 (w). **HRMS (TOF ES+) m/z:** $[M + H]^+$ Calcd for $C_{16}H_{16}NO_4$ 286.1074; Found 286.1073.

2-((1r,3r,5r,7r)-Adamantane-2-carbonyl)-4-benzyl-3-methylisoxazol-5(2*H*)-one (1k)

Synthesized via Procedure C

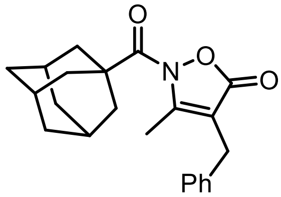

Chemical Formula: $C_{22}H_{25}NO_3$
Exact Mass: 351.1834

Yield: 88% (804 mg, 2.28 mmol)

Appearance: White solid

^{1}H-NMR (400 MHz, $CDCl_3$) δ/ppm 7.33 – 7.20 (m, 5H), 3.60 (s, 2H), 2.53 (s, 3H), 2.14 – 2.03 (m, 9H), 1.79 – 1.74 (m, 6H). **^{13}C-NMR (100 MHz, $CDCl_3$)** δ/ppm 172.2 (C), 167.1 (C), 155.3 (C), 138.1 (C), 128.6 (2CH), 128.1 (2CH), 126.6 (CH), 104.8 (C), 43.8 (C), 37.3 ($3CH_2$), 36.2 ($3CH_2$), 27.8 (3CH), 27.4 (CH_2), 14.7 (CH_3). **IR (neat)** ν/cm^{-1}: 2980 (m), 1765 (m), 1685 (s), 1444 (w), 1342 (s), 1077 (w), 947 (m), 788 (w), 692 (s). **HRMS (TOF ES+) m/z:** $[M + H]^+$ Calcd for $C_{22}H_{26}NO_3$ 352.1907; Found 352.1906.

Isobutyl 4-benzyl-3-methyl-5-oxoisoxazole-2(5*H*)-carboxylate (1l)

Synthesized via Procedure C

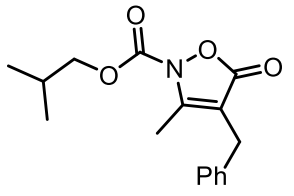

Chemical Formula: $C_{16}H_{19}NO_4$
Exact Mass: 289.1314

Yield: 74% (579 mg, 2.00 mmol)

Appearance: White solid

^{1}H-NMR (500 MHz, $CDCl_3$) δ/ppm 7.31 – 7.25 (m, 2H), 7.24 – 7.18 (m, 3H), 4.08 (d, *J* = 6.6 Hz, 2H), 3.59 (s, 2H), 2.49 – 2.47 (s, 3H), 2.05 (dpd, *J* = 13.4, 6.7, 0.9 Hz, 1H), 0.99 (d, *J* = 6.8 Hz, 6H). **^{13}C-NMR (125 MHz, $CDCl_3$)** δ/ppm 167.3 (C), 154.4 (C), 147.3 (C), 137.9 (C), 128.5 (2CH), 128.0 (2CH), 126.5 (CH), 105.5 (C), 73.9 (CH_2), 27.6 (CH) 27.5 (CH_2), 18.7 ($2CH_2$), 13.2 (CH_3). **IR (neat)** ν/cm^{-1}: 2959 (w), 1719 (s), 1626 (m), 1415 (m), 1347 (m), 1073 (s), 916 (m), 734 (s). **HRMS (TOF ES+) m/z:** $[M + H]^+$ Calcd for $C_{16}H_{20}NO_4$ 290.1387; Found 290.1387.

4-Benzyl-3-methyl-2-(morpholine-4-carbonyl)isoxazol-5(2*H*)-one (1m)

Synthesized via Procedure C

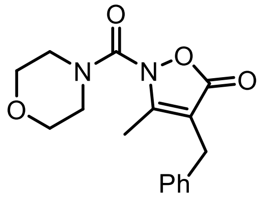

Chemical Formula: $C_{16}H_{18}N_2O_4$
Exact Mass: 302.1267

Yield: 35% (253 mg, 0.84 mmol)

Appearance: Colorless oil

^{1}H-NMR (500 MHz, $CDCl_3$) δ/ppm 7.31 – 7.26 (m, 2H), 7.24 – 7.18 (m, 3H), 3.77 – 3.72 (m, 4H), 3.65 – 3.62 (m, 4H), 3.59 (s, 1H), 2.34 (s, 1H). **^{13}C-NMR (125 MHz, $CDCl_3$)** δ/ppm 168.8 (C), 159.6 (C), 150.7 (C), 137.9 (C), 128.6 (2CH), 128.1 (2CH), 126.5 (CH), 106.1 (C), 66.4 ($2CH_2$), 46.0 ($2CH_2$) 27.7 (CH_2), 13.4 (CH_3). **IR (neat)** ν/cm^{-1}: 2968 (w), 1749 (m), 1696 (s), 1495 (w), 1367 (w), 1301 (m), 1114 (m), 1020 (m), 703 (m). **HRMS (TOF ES+) m/z:** $[M + H]^+$ Calcd for $C_{16}H_{19}N_2O_4$ 303.1339; Found 303.1341.

2-Acetyl-4-benzyl-3-cyclopropylisoxazol-5(2*H*)-one (1n)

Synthesized via Procedure C

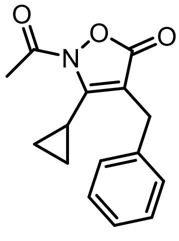

Chemical Formula: $C_{15}H_{15}NO_3$
Exact Mass: 257.1052

Yield: 75% (364 mg, 1.41 mmol)

Appearance: Colorless oil

^{1}H-NMR (500 MHz, $CDCl_3$) δ/ppm 7.31 – 7.27 (m, 2H), 7.21 (m , 3H), 3.67 (s, 2H), 2.44 (s, 3H), 2.21 (tt, *J* = 8.8, 5.7 Hz, 1H), 1.14 – 1.08 (m, 2H), 0.93 – 0.88 (m, 2H). **^{13}C-NMR (125 MHz, $CDCl_3$)** δ/ppm 167.9 (C), 164.6 (C), 157.8 (C), 138.3 (C), 128.8 (2CH), 128.1 (2CH), 126.8 (CH), 106.1 (C), 27.9 (CH_2), 23.2 (CH_3), 9.6 (CH), 7.8 ($2CH_2$). **IR (neat)** ν/cm^{-1}: 2980 (w), 1759 (m), 1710 (s), 1412 (w), 1307 (s), 1033 (w), 974 (m), 818 (w), 698 (m). **HRMS (TOF ES+) m/z:** $[M + H]^+$ Calcd for $C_{15}H_{16}NO_3$ 258.1125; Found 258.1122.

4-(2-hydroxybenzyl)-2-isobutyryl-3-methylisoxazol-5(2*H*)-one (1o)

Synthesized via Procedure C

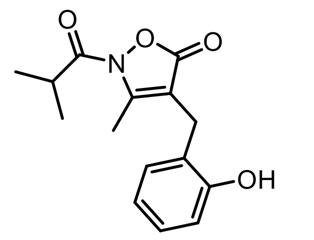

Chemical Formula: $C_{15}H_{17}NO_4$
Exact Mass: 275.1158

Yield: 71% (284 mg, 1.03 mmol)

Appearance: Colorless oil

^{1}H-NMR (500 MHz, CDCl$_3$) δ/ppm 7.17 – 7.12 (m, 2H), 7.09 (s, 1H), 6.92 (dt, *J* = 7.6, 1.1 Hz, 1H), 6.87 (td, *J* = 7.5, 1.2 Hz, 1H), 3.58 (s, 2H), 3.19 (hept, *J* = 6.9 Hz, 1H), 2.68 (s, 3H), 1.22 (d, *J* = 6.9 Hz, 6H). **^{13}C-NMR (125 MHz, CDCl$_3$)** δ/ppm 172.3 (C), 169.4 (C), 154.5 (C), 154.5 (C), 130.4 (CH), 128.8 (CH), 124.8 (C), 121.2 (CH), 118.1 (CH), 106.4 (C), 33.4 (CH$_2$), 22.7 (CH$_2$), 18.5 (2CH$_3$), 14.1 (CH$_3$). **IR (neat)** ν/cm^{-1}: 2980 (m), 1672 (s), 1571 (m), 1375 (w), 1240 (w), 967 (m), 876 (s), 761 (w), 696 (s). **HRMS (TOF ES+) m/z:** [M + H]$^+$ Calcd for $C_{15}H_{18}NO_4$ 276.1230; Found 276.1232.

4-(4-Bromobenzyl)-2-isobutyryl-3-methylisoxazol-5(2*H*)-one (1p)

Synthesized via Procedure C

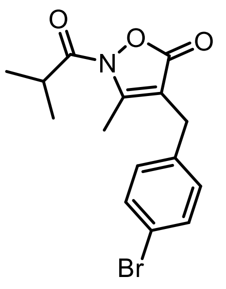

Chemical Formula: $C_{15}H_{16}BrNO_3$
Exact Mass: 337.0314

Yield: 88% (556 mg, 1.15 mmol)

Appearance: Colorless oil

^{1}H-NMR (500 MHz, CDCl$_3$) δ/ppm 7.45 – 7.38 (m, 2H), 7.16 – 7.08 (m, 2H), 3.54 (s, 2H), 3.20 (hept, *J* = 6.9 Hz, 1H), 2.55 (s, 3H), 1.23 (d, *J* = 6.9 Hz, 6H). **^{13}C-NMR (125 MHz, CDCl$_3$)** δ/ppm 172.2 (C), 167.1 (C), 154.4 (C), 137.1 (C), 132.0 (2CH), 130.1 (2CH), 120.8 (C), 105.5 (C), 33.3 (CH), 27.1 (CH$_2$), 18.5 (2CH$_3$), 14.1 (CH$_3$). **IR (neat)** ν/cm^{-1}: 2980 (m), 1732 (s), 1619 (s), 1381 (m), 1113 (m), 991 (m), 878 (m), 761 (s), 684 (s). **HRMS (TOF ES+) m/z:** [M + H]$^+$ Calcd for $C_{15}H_{17}BrNO_3$ 338.0386; Found 338.0381.

4-((1*H*-Indol-3-yl)methyl)-2-isobutyryl-3-methylisoxazol-5(2*H*)-one (1q)

Synthesized via Procedure C

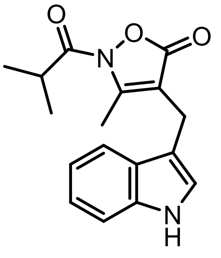

Chemical Formula: $C_{17}H_{18}N_2O_3$
Exact Mass: 298.1317

Yield: 90% (680 mg, 2.28 mmol)

Appearance: Colorless oil

^{1}H-NMR (400 MHz, CDCl$_3$) δ/ppm 8.05 (s, 1H), 7.69 – 7.60 (m, 1H), 7.36 (dt, *J* = 8.1, 1.0 Hz, 1H), 7.21 (ddd, *J* = 8.1, 7.0, 1.2 Hz, 1H), 7.14 (ddd, *J* = 8.0, 7.0, 1.1 Hz, 1H), 7.08 (dd, *J* = 2.2, 1.1 Hz, 1H), 3.81 – 3.66 (m, 2H), 3.20 (hept, *J* = 6.8 Hz, 1H), 2.59 (s, 3H), 1.22 (d, *J* = 6.9 Hz, 6H). **^{13}C-NMR (100 MHz, CDCl$_3$)** δ/ppm 172.0 (C), 167.2 (C), 154.0 (C), 136.3 (C), 126.7 (C),

122.3 (CH), 122.2 (CH), 119.6 (CH), 118.6 (CH), 112.3 (C), 111.2 (CH), 106.0 (C), 33.1 (CH), 18.4 (2CH_3), 17.5 (CH_2), 13.9 (CH_3). **IR (neat)** v/cm^{-1}: 3391 (w), 2881 (w), 1749 (s), 1693 (s), 1410 (w), 1310 (m), 1224 (m), 1007 (w), 872 (w), 731 (s), 582 (w). **HRMS (TOF ES+) m/z:** $[M + H]^+$ Calcd for $C_{17}H_{19}N_2O_3$ 299.1390; Found 299.1390 ($M+H^+$).

***tert*-Butyl 3-((2-isobutyryl-3-methyl-5-oxo-2,5-dihydroisoxazol-4-yl)methyl)-1*H*-indole-1-carboxylate (1r)**

Synthesized via Procedure C

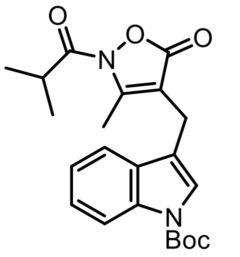

Chemical Formula: $C_{22}H_{26}N_2O_5$
Exact Mass: 398.1842

Yield: 75% (461 mg, 1.15 mmol)

Appearance: Colorless oil

^{1}H-NMR (500 MHz, $CDCl_3$) δ/ppm 8.16 – 8.02 (s, 1H), 7.63 – 7.57 (m, 1H), 7.41 (s, 1H), 7.33 (ddd, *J* = 8.4, 7.2, 1.3 Hz, 1H), 7.28 – 7.23 (m, 1H), 3.68 (d, *J* = 1.2 Hz, 2H), 3.23 (hept, *J* = 6.9 Hz, 1H), 2.59 (s, 3H), 1.68 (s, 9H), 1.24 (d, *J* = 6.9 Hz, 6H). **^{13}C-NMR (125 MHz, $CDCl_3$)** δ/ppm 172.0 (C), 167.0 (C), 154.7 (C), 149.7 (C), 135.5 (C), 129.7 (C), 124.6 (CH), 123.1 (CH), 122.6 (CH), 119.0 (C), 117.0 (CH), 115.3 (CH), 104.5 (C), 83.8 (C), 33.2 (CH), 28.2 (3CH_3) 18.4 (2CH_3), 17.4 (CH_2), 14.0 (CH_3). **IR (neat)** v/cm^{-1}: 2979 (w), 1709 (s), 1629 (w), 1359 (s), 1254 (m), 1080 (m), 906 (w), 856 (w), 729 (s). **HRMS (TOF ES+) m/z:** $[M + H]^+$ Calcd for $C_{22}H_{27}N_2O_5$ 399.1914; Found 399.1914 ($M+H^+$).

2-Isobutyryl-3-methyl-4-(thiophen-2-ylmethyl)isoxazol-5(2*H*)-one (1s)

Synthesized via Procedure C

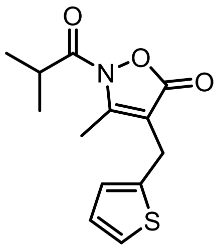

Chemical Formula: $C_{13}H_{15}NO_3S$
Exact Mass: 265.0773

Yield: 78% (500 mg, 1.88 mmol)

Appearance: White solid

^{1}H-NMR (400 MHz, $CDCl_3$) δ/ppm 7.14 (dd, *J* = 5.1, 1.3 Hz, 1H), 6.91 (dd, *J* = 5.1, 3.5 Hz, 1H), 6.88 (dq, *J* = 3.4, 1.0 Hz, 1H), 3.79 (s, 2H), 3.21 (hept, *J* = 6.9 Hz, 1H), 2.58 (s, 3H), 1.23 (d, *J* = 6.9 Hz, 6H). **^{13}C-NMR (100 MHz, $CDCl_3$)** 172.3 (C), 166.7 (C), 154.5 (C), 140.4 (C), 127.2 (2CH), 125.5 (2CH), 124.3 (CH), 105.6 (C), 33.3 (CH), 22.0 (CH_2), 18.5 (2CH_3), 14.1 (CH_3)**. IR (neat)** v/cm^{-1}: 2979 (w), 1762 (m), 1704 (s), 1336 (m), 1273 (s), 1008 (w), 966 (m), 773 (w), 697 (s). **HRMS (TOF ES+) m/z:** $[M + H]^+$ Calcd for $C_{13}H_{16}NO_3S$ 266.0845; Found 266.0845.

Methyl 4-(4-benzyl-3-methyl-5-oxoisoxazol-2(5H)-yl)-4-oxobutanoate (1t)

Synthesized via Procedure C

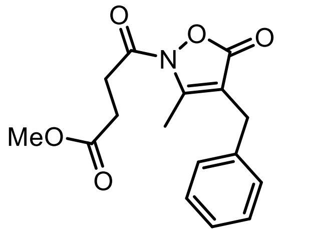

Chemical Formula: $C_{16}H_{17}NO_5$
Exact Mass: 303.1107

Yield: 84% (2.69 g, 8.87 mmol)

Appearance: White solid

^{1}H-NMR (500 MHz, CDCl$_3$) δ/ppm 7.32 – 7.27 (m, 2H), 7.24 – 7.18 (m, 3H), 3.70 (s, 3H), 3.60 (s, 2H), 3.05 (ddd, *J* = 7.5, 6.0, 1.1 Hz, 2H), 2.68 (ddd, *J* = 7.5, 6.0, 1.1 Hz, 2H), 2.53 (d, *J* = 1.0 Hz, 3H). **^{13}C-NMR (125 MHz, CDCl$_3$)** δ/ppm 172.3 (C), 166.8 (C), 166.4 (C), 153.8 (C), 137.7 (C), 128.7 (2CH), 128.1 (2CH), 126.7 (CH), 106.3 (C), 51.9 (CH$_3$), 29.6 (CH$_2$), 27.4 (CH$_2$), 27.4 (CH$_2$), 13.7 (CH$_3$). **IR (neat)** ν/cm^{-1}: 3061 (w), 1731 (s), 1708 (s), 1437 (w), 1366 (m), 1210 (m), 1162 (s), 946 (w), 845 (w), 702 (s). **HRMS (TOF ES+) m/z:** [M + H]$^+$ Calcd for $C_{16}H_{18}NO_5$ 304.1179; Found 304.1179.

Characterization of Oxazoles (2a-3a)

5-Benzyl-2,4-dimethyloxazole (2a)

Synthesized via Procedure D

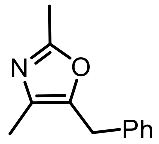

Chemical Formula: $C_{12}H_{13}NO$
Exact Mass: 187.0997

Yield: 61% (199 mg, 1.05 mmol)

Appearance: Yellow oil

^{1}H-NMR (500 MHz, CDCl$_3$) δ/ppm 7.32 – 7.26 (m, 2H), 7.21 (m, 1H), 7.19 – 7.16 (m, 2H), 3.88 (s, 2H), 2.34 (s, 3H), 2.09 (s, 3H). **^{13}C-NMR (125 MHz, CDCl$_3$)** δ/ppm 159.3 (C), 144.6 (C), 137.7 (C), 130.9 (C), 128.4 (2CH), 128.2 (2CH) 126.4 (CH), 30.7 (CH$_2$), 13.7 (CH$_3$), 11.0 (CH$_3$). **IR (neat)** ν/cm^{-1}: 2980 (w), 1754 (m), 1666 (m), 1581 (w), 1495 (w), 1373 (w), 1088 (w), 914 (w), 700 (s). **HRMS (TOF ES+) m/z:** [M + H]$^+$ Calcd for $C_{12}H_{14}NO$ 188.1070; Found 188.1071. In agreement with previously reported.[12]

5-Benzyl-2-isopropyl-4-methyloxazole (2b)

Synthesized via Procedure D

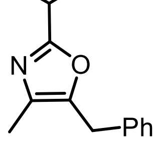

Chemical Formula: $C_{14}H_{17}NO$
Exact Mass: 215.1310

Yield: 67% (149 mg, 0.69 mmol)

Appearance: Yellow oil

^{1}H-NMR (500 MHz, CDCl$_3$) δ/ppm 7.33 – 7.27 (m, 2H), 7.24 – 7.19 (m, 1H), 7.21 – 7.15 (m, 2H), 3.91 (s, 2H), 2.98 (hept, *J* = 7.0 Hz, 1H), 2.09 (s, 3H), 1.30 (d, *J* = 7.0 Hz, 6H). **^{13}C-NMR (125 MHz, CDCl$_3$)** δ/ppm 167.0 (C), 144.1 (C),

137.8 (C), 130.7 (C), 128.5 (2CH), 128.2 (2CH), 126.4 (CH), 30.8 (CH_2), 28.2 (CH), 20.4 ($2CH_3$), 11.2 (CH_3). **IR (neat)** v/cm^{-1}: 2975 (s), 1702 (m), 1567 (w), 1454 (m), 1250 (w), 1084 (m), 956 (w), 688 (s). **HRMS (TOF ES+) m/z:** $[M + H]^+$ Calcd for $C_{14}H_{18}NO$ 216.1383; Found 216.1393.

5-Benzyl-2-ethyl-4-methyloxazole (2c)

Synthesized via Procedure D

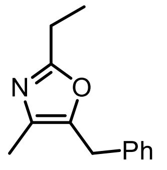

Chemical Formula: $C_{13}H_{15}NO$
Exact Mass: 201.1154

Yield: 66% (139 mg, 0.69 mmol)

Appearance: Yellow oil

^{1}H-NMR (500 MHz, $CDCl_3$) δ/ppm 7.31 – 7.27 (m, 2H), 7.25 – 7.20 (m, 1H), 7.21 – 7.15 (m, 2H), 3.90 (s, 2H), 2.68 (q, *J* = 7.6 Hz, 2H), 2.10 (s, 3H), 1.28 (t, *J* = 7.6 Hz, 3H). **^{13}C-NMR (125 MHz, $CDCl_3$)** δ/ppm 163.7 (C), 144.3, 137.7 (C), 130.7 (C), 128.4 (2CH), 128.1 (2CH), 126.4 (CH), 30.7 (CH_2), 21.4 (CH_2), 11.0 (CH_3), 11.0 (CH_3). **IR (neat)** v/cm^{-1}: 2980 (s), 1701 (m), 1582 (w), 1379 (m), 1248 (m), 1174 (m), 1073 (w), 956 (w), 699 (s). **HRMS (TOF ES+) m/z:** $[M + NH_4]^+$ Calcd for $C_{13}H_{19}N_2O$ 219.1492 found 219.1492.

5-Benzyl-2-butyl-4-methyloxazole (2d)

Synthesized via Procedure D

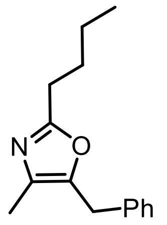

Chemical Formula: $C_{15}H_{19}NO$
Exact Mass: 229.1467

Yield: 62% (143 mg, 0.62 mmol)

Appearance: Yellow oil

^{1}H-NMR (500 MHz, $CDCl_3$) δ/ppm 7.33 – 7.28 (m, 2H), 7.26 – 7.19 (m, 1H), 7.20 – 7.15 (m, 2H), 3.90 (s, 2H), 2.68 – 2.61 (m, 2H), 2.09 (s, 3H), 1.75 – 1.67 (m, 2H), 1.36 (m, 2H), 0.91 (t, *J* = 7.4 Hz, 3H). **^{13}C-NMR (125 MHz, $CDCl_3$)** δ/ppm 163.2 (C), 144.5 (C), 138.0 (C), 131.1 (C), 128.7 (2CH), 128.4 (2CH), 126.7 (CH), 31.0 (CH_2), 29.2 (CH_2), 28.0 (CH_2), 22.4 (CH_2), 13.8 (CH_3), 11.3 (CH_3). **IR (neat)** v/cm^{-1}: 2980 (s), 1701 (m), 1582 (w), 1379 (m), 1248 (m), 1174 (m), 1073 (w), 956 (w), 699 (s). **HRMS (TOF ES+) m/z:** $[M + H]^+$ Calcd for $C_{15}H_{20}NO$ 230.1539; Found 230.1540.

5-Benzyl-2-cyclopropyl-4-methyloxazole (2e)

Synthesized via Procedure D

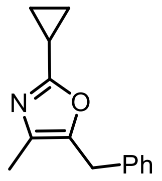

Chemical Formula: $C_{14}H_{15}NO$
Exact Mass: 213.1154

Yield: 57% (124 mg, 0.58 mmol)

Appearance: Yellow oil

^{1}H-NMR (500 MHz, CDCl$_3$) δ/ppm 7.32 – 7.26 (m, 2H), 7.25 – 7.20 (m, 1H), 7.19 – 7.14 (m, 2H), 3.87 (s, 2H), 2.06 (s, 3H), 1.95 (m, 1H), 1.01 – 0.96 (m, 2H), 0.96 – 0.92 (m, 2H). **^{13}C-NMR (125 MHz, CDCl$_3$)** δ/ppm 164.0 (C), 143.8 (C), 137.7 (C), 130.8 (C), 128.4 (2CH), 128.2 (2CH), 126.4 (CH), 30.7 (CH$_2$), 11.0 (CH$_3$), 8.7 (CH), 7.8 (2CH$_2$). **IR (neat)** ν/cm^{-1}: 3349 (w), 3190 (w), 2980 (s), 1669 (s), 1655 (s), 1495 (w), 1264 (m), 1029 (w), 698 (s). **HRMS (TOF ES+) m/z:** [M + H]$^+$ Calcd for $C_{14}H_{16}NO$ 214.1226; Found 214.1228 (M+H$^+$).

5-Benzyl-2-cyclobutyl-4-methyloxazole (2f)

Synthesized via Procedure D

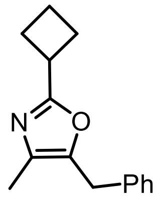

Chemical Formula: $C_{15}H_{17}NO$
Exact Mass: 227.1310

Yield: 52% (125 mg, 0.58 mmol)

Appearance: Yellow oil

^{1}H-NMR (500 MHz, CDCl$_3$) δ/ppm 7.32 – 7.27 (m, 2H), 7.25 – 7.20 (m, 1H), 7.20 – 7.15 (m, 2H), 3.91 (s, 2H), 3.53 (pd, *J* = 8.6, 1.1 Hz, 1H), 2.43 – 2.33 (m, 2H), 2.34 – 2.28 (m, 2H), 2.10 (s, 3H), 2.04 – 1.98 (m, 1H), 1.96 – 1.89 (m, 1H). **^{13}C-NMR (125 MHz, CDCl$_3$)** δ/ppm 165.3 (C), 144.3 (C), 137.7 (C), 130.9 (C), 128.5 (2CH), 128.2 (2CH), 126.5 (CH), 33.0 (CH), 30.8 (CH$_2$), 27.3 (2CH$_2$), 18.5 (CH$_2$), 11.1 (CH$_3$). **IR (neat)** ν/cm^{-1}: 2980 (s), 1701 (m), 1655 (m), 1389 (w), 1250 (m), 1187 (m), 1087 (w), 966 (w), 696 (s). **HRMS (TOF ES+) m/z:** [M + NH$_4$]$^+$ Calcd for $C_{15}H_{21}N_2O$ 245.1648; Found 245.1648.

5-Benzyl-2-cyclohexyl-4-methyloxazole (2g)

Synthesized via Procedure D

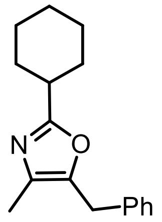

Chemical Formula: $C_{17}H_{21}NO$
Exact Mass: 255.1623

Yield: 63% (160 mg, 0.63 mmol)

Appearance: Yellow oil

^{1}H-NMR (500 MHz, CDCl$_3$) δ/ppm 7.33 – 7.24 (m, 2H), 7.24 – 7.18 (m, 1H), 7.20 – 7.13 (m, 2H), 3.89 (s, 2H), 2.68 (tt, *J* = 11.5, 3.6 Hz, 1H), 2.09 (s, 3H), 2.06 – 1.97 (m, 2H), 1.84 – 1.74 (m, 2H), 1.72 – 1.62 (m, 1H), 1.59 – 1.46 (m, 2H), 1.37 – 1.18 (m, 3H). **^{13}C-NMR (125 MHz, CDCl$_3$)** δ/ppm 166.2 (C), 143.9 (C), 137.7 (C), 130.5 (C), 128.4 (2CH), 128.1 (2CH), 126.4 (CH), 37.3 (CH), 30.7 (2CH$_2$), 30.5 (CH$_2$), 25.7 (2CH$_2$),

25.5 (CH_2), 11.0 (CH_3). **IR (neat)** v/cm^{-1}: 2980 (s), 1703 (m), 1554 (w), 1450 (m), 1388 (m), 1174 (m), 1088 (m), 892 (w), 696 (s). **HRMS (TOF ES+) m/z:** $[M + H]^+$ Calcd for $C_{17}H_{22}NO$ 256.1696; Found 256.1699.

5-Benzyl-4-methyl-2-(tetrahydro-2H-pyran-4-yl)oxazole (2h)

Synthesized via Procedure D

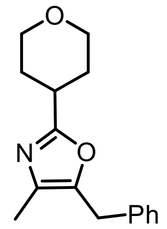

Chemical Formula: $C_{16}H_{19}NO_2$
Exact Mass: 257.1416

Yield: 56% (125 mg, 0.58 mmol)

Appearance: Yellow oil

^{1}H-NMR (400 MHz, $CDCl_3$) δ/ppm 7.32 – 7.27 (m, 2H), 7.24 – 7.19 (m, 1H), 7.19 – 7.14 (m, 2H), 3.99 (ddd, *J* = 11.6, 3.9, 2.8 Hz, 2H), 3.91 (s, 2H), 3.47 (ddd, *J* = 11.7, 10.7, 3.0 Hz, 2H), 2.93 (tt, *J* = 10.7, 4.5 Hz, 1H), 2.10 (s, 3H), 1.96 – 1.82 (m, 4H). **^{13}C-NMR (100 MHz, $CDCl_3$)** δ/ppm 164.3 (C), 144.5 (C), 137.6 (C), 130.9 (C), 128.5 (2CH), 128.2 (2CH), 126.5 (CH), 67.1 (2CH_2), 34.5 (CH), 30.8 (2CH_2), 30.1 (CH_2), 11.1 (CH_3). **IR (neat)** v/cm^{-1}: 2979 (m), 2849 (w), 1702 (m), 1567 (w), 1447 (w), 1240 (m), 1128 (s), 983 (w), 835 (w). **HRMS (TOF ES+) m/z:** $[M + H]^+$ Calcd for $C_{16}H_{20}NO_2$ 258.1489; found 258.1498.

Methyl 4-(5-Benzyl-4-methyloxazol-2-yl)bicyclo[2.2.2]octane-1-carboxylate (2i)

Synthesized via Procedure D

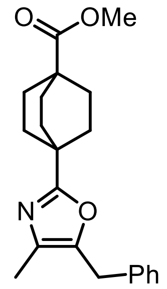

Chemical Formula: $C_{21}H_{25}NO_3$
Exact Mass: 339.1834

Yield: 59% (130 mg, 0.38 mmol)

Appearance: Yellow oil

^{1}H-NMR (400 MHz, $CDCl_3$) δ/ppm 7.34 – 7.24 (m, 2H), 7.24 – 7.18 (m, 1H), 7.17 – 7.12 (m, 2H), 3.89 (s, 2H), 3.64 (s, 3H), 2.07 (s, 3H), 1.95 – 1.83 (m, 12H). **^{13}C-NMR (100 MHz, $CDCl_3$)** δ/ppm 178.0 (C), 167.7 (C), 144.4 (C), 137.8 (C), 130.8 (C), 128.6 (2CH), 128.2 (2CH), 126.5 (CH), 51.7 (CH_2), 38.7 (C), 33.2 (C), 30.8 (CH_3), 29.2 (3CH_2), 27.9 (3CH_2), 11.2 (CH_3). **IR (neat)** v/cm^{-1}: 3569 (w), 2980 (s), 1720 (m), 1555 (w), 1472 (w), 1251 (m), 1069 (m), 943 (w), 845 (w), 730 (s). **HRMS (TOF ES+) m/z:** $[M + H]^+$ Calcd for $C_{21}H_{26}NO_3$ 340.1907; Found 340.1908.

3-(5-Benzyl-4-methyloxazol-2-yl)cyclobutan-1-one (2j)

Synthesized via Procedure D

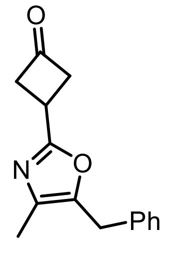

Chemical Formula: $C_{15}H_{15}NO_2$
Exact Mass: 241.1103

Yield: 50% (110 mg, 0.46 mmol)

Appearance: Yellow oil

^{1}H-NMR (400 MHz, $CDCl_3$) δ/ppm 7.32 – 7.28 (m, 2H), 7.26 – 7.20 (m, 1H), 7.19 – 7.14 (m, 2H), 3.92 (s, 2H), 3.69 – 3.59 (m, 1H), 3.55 – 3.33 (m, 4H), 2.12 (s, 3H). **^{13}C-NMR (100 MHz, $CDCl_3$)** δ/ppm 204.4 (C), 162.9 (C), 145.4 (C), 137.3 (C), 131.3 (C), 128.5 (2CH), 128.2 (2CH), 126.6 (CH), 52.9 ($2CH_2$), 30.8 (CH_2), 22.1 (CH), 11.1 (CH_3). **IR (neat)** ν/cm^{-1}: 2980 (m), 1787 (s), 1662 (m), 1670 (m), 1377 (m), 1171 (m), 1092 (m), 699 (m). **HRMS (TOF ES+) m/z:** $[M + H]^+$ Calcd for $C_{15}H_{16}NO_2$ 242.1176; Found 242.1178.

2-((3r,5r,7r)-Adamantane-1-carbonyl)-4-benzyl-3-methylisoxazol-5(2*H*)-one (2k)

Synthesized via Procedure D

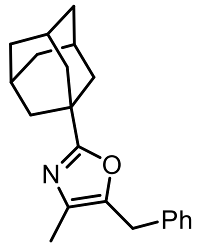

Chemical Formula: $C_{21}H_{25}NO$
Exact Mass: 307.1936

Yield: 78% (370 mg, 1.22 mmol)

Appearance: White solid

^{1}H-NMR (400 MHz, $CDCl_3$) δ/ppm 7.32 – 7.27 (m, 2H), 7.24 – 7.19 (m, 1H), 7.20 – 7.13 (m, 2H), 3.91 (s, 2H), 2.08 (s, 3H), 2.04 (m, 3H), 2.00 (m, 6H), 1.75 (m, 6H). **^{13}C-NMR (100 MHz, $CDCl_3$)** δ/ppm 168.9 (C), 143.7 (C), 137.9 (C), 130.6 (C), 128.4 (2CH), 128.1 (2CH), 126.3 (CH), 40.3 ($3CH_2$), 36.4 ($3CH_2$) , 35.3 (C) , 30.8 (3CH) , 28.0 (CH_2), 11.2 (CH_3). **IR (neat)** ν/cm^{-1}: 2980 (s), 2902 (m), 1560 (w), 1453 (w), 1252 (w), 1058 (w), 726 (m), 638 (w). **HRMS (TOF ES+) m/z:** $[M + H]^+$ Calcd for $C_{21}H_{26}NO$ 308.2009; Found 308.2010.

5-Benzyl-2-isobutoxy-4-methyloxazole (2l)

Synthesized via Procedure D

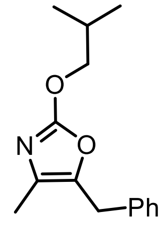

Chemical Formula: $C_{15}H_{19}NO_2$
Exact Mass: 245.1416

^{1}H-NMR Yield: 40% (66 mg, 0.27 mmol)

Appearance: Yellow oil

^{1}H-NMR (400 MHz, $CDCl_3$) δ/ppm 7.31 – 7.26 (m, 0H), 7.23 – 7.17 (m, 3H), 4.05 (d, *J* = 6.7 Hz, 2H), 3.81 (s, 2H), 2.07 (m, 1H), 2.05 (s, 3H), 0.98 (d, *J* = 4.6 Hz, 6H). **^{13}C-NMR (100 MHz, $CDCl_3$)** δ/ppm) 160.5 (C), 154.5 (C), 139.5 (C), 138.0 (C), 128.5 (2CH), 128.3 (2CH), 126.5 (CH), 74.0 (CH_2), 30.7 (CH_2),

27.8 (CH), 18.8 (2CH_3), 11.5 (CH_3). **IR (neat)** ν/cm^{-1}: 2962 (w), 1597 (s), 1454 (w), 1382 (m), 1214 (w), 1080 (w), 1002 (w), 704 (m). **HRMS (TOF ES+) m/z:** $[M + H]^+$ Calcd for $C_{15}H_{20}NO_2$ 246.1489; Found 246.1490.

5-Benzyl-4-cyclopropyl-2-methyloxazole (2n)

Synthesized via Procedure D

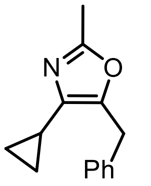

Chemical Formula: $C_{14}H_{15}NO$
Exact Mass: 213.1154

Yield: 57% (115 mg, 0.54 mmol)

Appearance: Yellow oil

^{1}H-NMR (500 MHz, $CDCl_3$) δ/ppm 7.34 – 7.29 (m, 2H), 7.25 – 7.20 (m, 3H), 3.98 (s, 2H), 2.32 (s, 3H), 1.73 – 1.65 (m, 1H), 0.95 – 0.68 (m, 4H). **^{13}C-NMR (100 MHz, $CDCl_3$)** δ/ppm 159.4 (C), 144.7 (C), 137.9 (C), 136.3 (C), 128.5 (2CH), 128.3 (2CH), 126.5 (CH), 30.8 (CH_2), 13.9 (CH_3), 6.4 (CH), 6.0 (2CH_2). **IR (neat)** ν/cm^{-1}: 3337 (w), 2980 (w), 1696 (m), 1659 (m), 1386 (m), 1175 (w), 1046 (w), 916 (w), 737 (w), 696 (s). **HRMS (TOF ES+) m/z:** $[M + H]^+$ Calcd for $C_{14}H_{16}NO$ 214.1226; Found 214.1230.

2-((2-Isopropyl-4-methyloxazol-5-yl)methyl)phenol (2o)

Synthesized via Procedure D

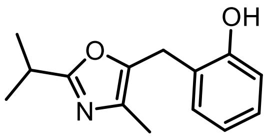

Chemical Formula: $C_{14}H_{17}NO_2$
Exact Mass: 231.1259

Yield: 59% (64 mg, 0.28 mmol)

Appearance: Yellow oil

^{1}H-NMR (500 MHz, $CDCl_3$) δ/ppm 7.10 – 7.04 (m, 2H), 6.85 – 6.78 (m, 2H), 3.94 (s, 2H), 3.01 (hept, *J* = 7.0 Hz, 1H), 2.06 (s, 3H), 1.29 (d, *J* = 7.0 Hz, 6H). **^{13}C-NMR (125 MHz, $CDCl_3$)** δ/ppm 167.3 (C), 154.6 (C), 144.6 (C), 130.4 (C), 130.0 (CH), 127.9 (CH), 124.4 (C), 120.1 (CH), 115.4 (CH), 28.4 (CH), 25.1 (CH_2), 20.4 (2CH_3), 10.9 (CH_3). **IR (neat)** ν/cm^{-1}: 2972 (w), 2872 (w), 1749 (w), 1595 (m), 1455 (w), 1268 (m), 1106 (m), 844 (w), 752 (s). **HRMS (TOF ES+) m/z:** $[M + H]^+$ Calcd for $C_{14}H_{18}NO_2$ 232.1332; Found 232.1333.

5-(4-Bromobenzyl)-2-isopropyl-4-methyloxazole (2p)

Synthesized via Procedure D

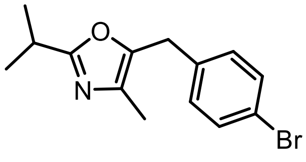

Chemical Formula: $C_{14}H_{16}BrNO$
Exact Mass: 293.0415

Yield: 67% (152 mg, 0.51 mmol)

Appearance: Yellow oil

^{1}H-NMR (400 MHz, $CDCl_3$) δ/ppm 7.42 – 7.35 (m, 2H), 7.05 – 6.96 (m, 2H), 3.84 (s, 2H), 2.96 (hept, *J* = 7.0 Hz, 1H), 2.08 (s, 3H), 1.28 (dd, *J* = 7.0, 1.8 Hz, 6H). **^{13}C-NMR (100 MHz, $CDCl_3$)** δ/ppm 167.3 (C), 143.7 (C), 136.9 (C), 131.7 (2CH), 131.1 (C), 130.1 (2CH), 120.5 (C), 30.4 (CH_2), 28.3 (CH), 20.5 (2CH_3), 11.3 (CH_3). **IR (neat)** ν/cm^{-1}:

2979 (m), 1698 (m), 1487 (m), 1398 (m), 1170 (w), 1069 (s), 1010 (s), 800 (w), 751 (w). **HRMS (TOF ES+) m/z:** [M + H]$^+$ Calcd for $C_{14}H_{17}BrNO$ 294.0488; Found 294.0486.

***tert*-Butyl 3-((2-isopropyl-4-methyloxazol-5-yl)methyl)-1H-indole-1-carboxylate (2r)**

Synthesized via Procedure D

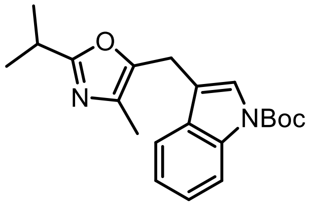

Chemical Formula: $C_{21}H_{26}N_2O_3$
Exact Mass: 354.1943

Yield: 57% (117 mg, 0.33 mmol)

Appearance: Yellow oil

^{1}H-NMR (500 MHz, CDCl$_3$) δ/ppm 8.12 (s, 1H), 7.50 (dt, *J* = 7.8, 1.0 Hz, 1H), 7.36 (s, 1H), 7.32 (ddd, *J* = 8.4, 7.2, 1.3 Hz, 1H), 7.23 (ddd, *J* = 8.1, 7.2, 1.1 Hz, 1H), 3.96 (s, 2H), 2.99 (hept, *J* = 7.0 Hz, 1H), 2.13 (s, 3H), 1.66 (s, 9H), 1.30 (d, *J* = 7.0 Hz, 6H). **^{13}C-NMR (125 MHz, CDCl$_3$)** δ/ppm) 167.1 (C), 149.8 (C), 143.3 (C), 135.6 (C), 130.6 (C), 130.1 (C), 124.6 (CH), 123.4 (CH), 122.6 (CH), 119.0 (CH), 117.0 (C), 115.3 (CH), 83.6 (C), 28.4 (CH), 28.3 (3CH$_3$), 21.0 (CH$_2$), 20.5 (2CH$_3$), 11.4 (CH$_3$). **IR (neat)** ν/cm^{-1}: 2975 (w), 1729 (m), 1607 (w), 1538 (w), 1451 (s), 1253 (m), 1149 (s), 1016 (m), 850 (w). **HRMS (TOF ES+) m/z:** [M + H]$^+$ Calcd for $C_{21}H_{27}N_2O_3$ 355.2016; Found 355.2018.

2-Isopropyl-4-methyl-5-(thiophen-2-ylmethyl)oxazole (2s)

Synthesized via Procedure D

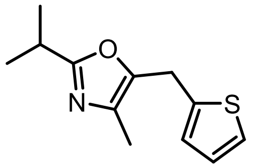

Chemical Formula: $C_{12}H_{15}NOS$
Exact Mass: 221.0874

Yield: 66% (174 mg, 0.79 mmol)

Appearance: Yellow oil

^{1}H-NMR (400 MHz, CDCl$_3$) δ/ppm 7.15 (dd, *J* = 5.1, 1.2 Hz, 1H), 6.92 (dd, *J* = 5.1, 3.5 Hz, 1H), 6.81 (dq, *J* = 3.4, 1.1 Hz, 1H), 4.09 (t, *J* = 0.8 Hz, 2H), 2.99 (hept, *J* = 7.0 Hz, 1H), 2.09 (s, 3H), 1.31 (d, *J* = 7.0 Hz, 7H). **^{13}C-NMR (100 MHz, CDCl$_3$)** δ/ppm 167.3 (C), 143.6 (C), 140.6 (C), 130.9 (C), 127.0 (CH), 125.2 (CH), 124.2 (CH), 28.4 (CH), 25.4 (CH$_2$), 20.6 (2CH$_3$), 11.4 (CH$_3$). **IR (neat)** ν/cm^{-1}: 2979 (m), 2844 (w), 1768 (w), 1647 (w), 1386 (m), 1155 (w), 1081 (w). 850 (w), 693 (s). **HRMS (TOF ES+) m/z:** [M + H]$^+$ Calcd for $C_{12}H_{16}NOS$ 222.0947; Found 222.0950.

Methyl 3-(5-benzyl-4-methyloxazol-2-yl)propanoate (2t)

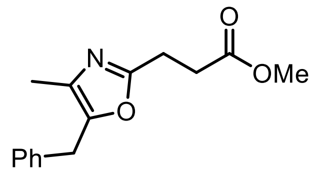

Chemical Formula: $C_{15}H_{17}NO_3$
Exact Mass: 259.1208

Yield: 55% (1.07 g, 4.12 mmol)

Appearance: Yellow oil

^{1}H-NMR (400 MHz, CDCl$_3$) δ/ppm 7.32 – 7.26 (m, 2H), 7.24 – 7.19 (m, 1H), 7.18 – 7.13 (m, 2H), 3.89 (s, 2H), 3.65 (s, 3H), 2.99 (t, *J* = 7.4 Hz, 2H), 2.76 (t, *J* = 7.4 Hz, 2H), 2.08 (s, 3H). **^{13}C-NMR (125 MHz, CDCl$_3$)** δ/ppm 172.6 (C) 161.2 (C), 145.1 (C), 137.7 (C), 131.2 (C), 128.7 (2CH), 128.4 (2CH), 126.7 (CH), 51.9 (CH_3), 30.9 (CH_2), 30.9 (CH_2), 23.5 (CH_2), 11.2 (CH_3). **IR (neat)** v/cm^{-1}: 3389 (w), 2952 (w), 1733 (s), 1671 (m), 1495 (w), 1363 (m), 1199 (s), 1027 (w), 843 (w), 700 (s). **HRMS (TOF ES+) m/z:** $[M + H]^+$ Calcd for $C_{15}H_{18}NO_3$ 260.1281; Found 260.1286.

3-(5-Benzyl-4-methyloxazol-2-yl)propanoic acid (3a)

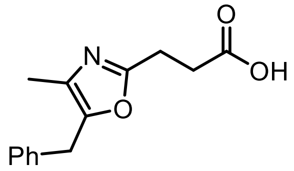

Chemical Formula: $C_{14}H_{15}NO_3$
Exact Mass: 245.1052

Yield: 99% (279 mg, 1.14 mmol)

Appearance: Yellow oil

^{1}H-NMR (500 MHz, CDCl$_3$) δ/ppm 9.60 (s, 1H), 7.32 – 7.27 (m, 2H), 7.24 – 7.20 (m, 1H), 7.18 – 7.14 (m, 2H), 3.89 (s, 2H), 3.02 (t, *J* = 7.4 Hz, 2H), 2.78 (t, *J* = 7.4 Hz, 2H), 2.09 (s, 3H). **^{13}C-NMR (125 MHz, CDCl$_3$)** δ/ppm) 175.5 (C), 161.8 (C), 145.4 (C), 137.4 (C), 130.7 (C), 128.7 (2CH), 128.4 (2CH), 126.8 (CH), 31.0 (CH_2), 30.8 (CH_2), 23.2 (CH_2), 10.7 (CH_3). **IR (neat)** v/cm^{-1}: 3004 (w), 1717 (s), 1570 (w), 1421 (w), 1353 (w), 1244 (m), 1183 (s), 974 (w), 702 (s). **HRMS (TOF ES+) m/z:** $[M + H]^+$ Calcd for $C_{14}H_{16}NO_3$ 246.1125; Found 246.1129 ($M+H^+$).

References

(1) Thakur, A.; Verma, M.; Setia, P.; Bharti, R.; Sharma, R.; Sharma, A.; Negi, N. P.; Anand, V.; Bansal, R. DFT analysis and in vitro studies of isoxazole derivatives as potent antioxidant and antibacterial agents synthesized via one-pot methodology. *Res. Chem. Intermed.* **2023**, *49* (3), 859-883. DOI: 10.1007/s11164-022-04910-7.
(2) Ciszewski, Ł. W.; Durka, J.; Gryko, D. Photocatalytic Alkylation of Pyrroles and Indoles with α-Diazo Esters. *Org. Lett.* **2019**, *21* (17), 7028-7032. DOI: 10.1021/acs.orglett.9b02612.
(3) Loftus, A.; De Gregorio, R.; Baumann, M. Continuous flow synthesis of alkynes from isoxazolones. *Org. Biomol. Chem.* **2025**, *23* (6), 1314-1319. DOI: 10.1039/D4OB01772B.
(4) Rocaboy, R.; Dailler, D.; Zellweger, F.; Neuburger, M.; Salomé, C.; Clot, E.; Baudoin, O. Domino Pd0-Catalyzed C(sp3)–H Arylation/Electrocyclic Reactions via Benzazetidine Intermediates. *Angew. Chem. Int. Ed.* **2018**, *57* (37), 12131-12135. DOI: 10.1002/anie.201807097.
(5) Mei, M.; Anand, D.; Zhou, L. Divergent Conversion of N-Acyl-isoxazol-5(2H)-ones to Oxazoles and 1,3-Oxazin-6-ones Using Photoredox Catalysis. *Org. Lett.* **2019**, *21* (10), 3548-3553. DOI: 10.1021/acs.orglett.9b00903.
(6) Zheng, Y.; Li, X.; Ren, C.; Zhang-Negrerie, D.; Du, Y.; Zhao, K. Synthesis of Oxazoles from Enamides via Phenyliodine Diacetate-Mediated Intramolecular Oxidative Cyclization. *J. Org. Chem* **2012**, *77* (22), 10353-10361. DOI: 10.1021/jo302073e.
(7) Haydari, F.; Kiyani, H. Urea-catalyzed multicomponent synthesis of 4-arylideneisoxazol-5(4H)-one derivatives under green conditions. *Res. Chem. Intermed.* **2023**, *49* (3), 837-858. DOI: 10.1007/s11164-022-04907-2.
(8) Boureghda, C.; Krid, A.; Dems, M. A.; Boutebdja, M.; Boulcina, R.; Debache, A. Facile synthesis, crystal structure, Hirshfeld surface analysis, DFT calculations, IR and UV–visible spectra analyzes, ADMET and molecular docking studies of arylideneisoxazolone derivatives. *J. Mol. Struct.* **2024**, *1317*, 139005.
(9) Patil, M. S.; Mudaliar, C.; Chaturbhuj, G. U. Sulfated polyborate catalyzed expeditious and efficient three-component synthesis of 3-methyl-4-(hetero) arylmethylene isoxazole-5 (4H)-ones. *Tetrahedron Lett.* **2017**, *58* (33), 3256-3261.
(10) Martínez-Pardo, P.; Laviós, A.; Sanz-Marco, A.; Vila, C.; Pedro, J. R.; Blay, G. Enantioselective Synthesis of Functionalized Diazaspirocycles from 4-Benzylideneisoxazol-5(4H)-one Derivatives and Isocyanoacetate Esters. *Adv. Synth, Catal.* **2020**, *362* (17), 3564-3569. DOI: 10.1002/adsc.202000611.
(11) Hellmuth, T.; Frey, W.; Peters, R. Regioselective Catalytic Asymmetric C-Alkylation of Isoxazolinones by a Base-Free Palladacycle-Catalyzed Direct 1,4-Addition. *Angew. Chem. Int. Ed.* **2015**, *54* (9), 2788-2791. DOI: 10.1002/anie.201410933.
(12) Cunico, R. F.; Kuan, C. P. Synthesis of oxazoles from O-trimethylsilyl acyltrimethylsilane cyanohydrins. *J. Org. Chem* **1992**, *57* (12), 3331-3336.

Copy of NMR Spectra

(*E*)-4-Benzylidene-3-methylisoxazol-5(4*H*)-one (S1)

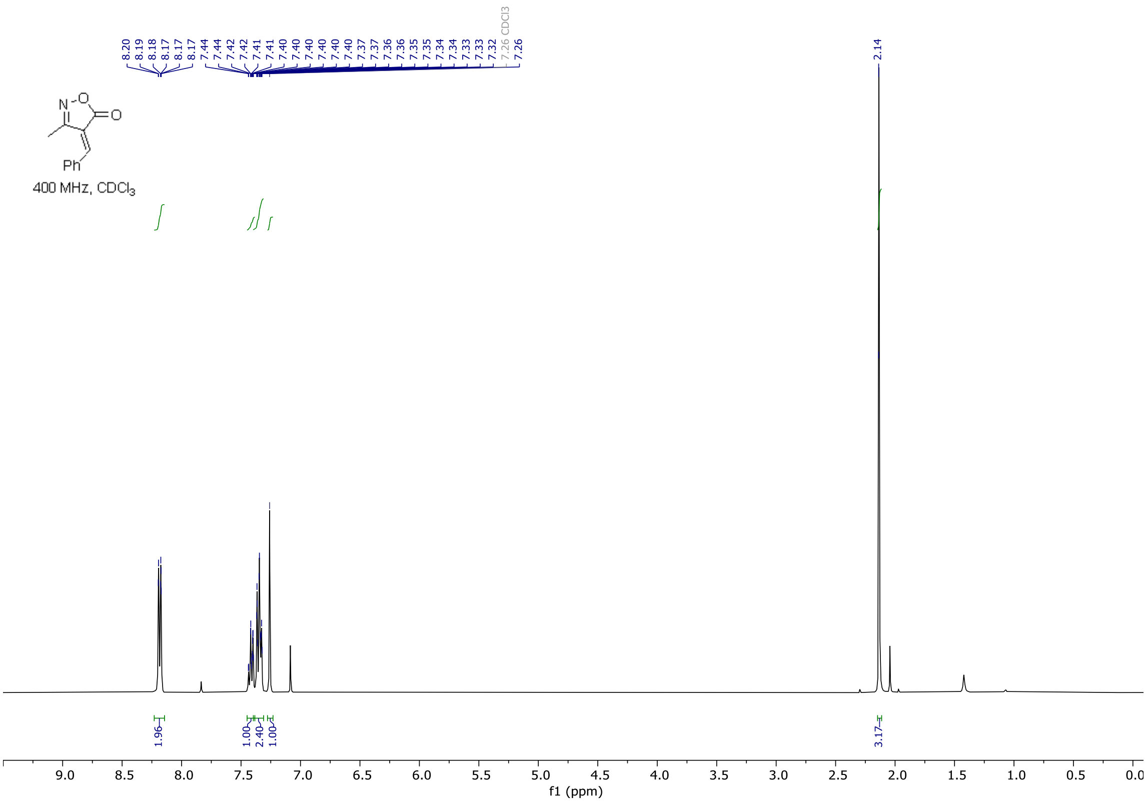

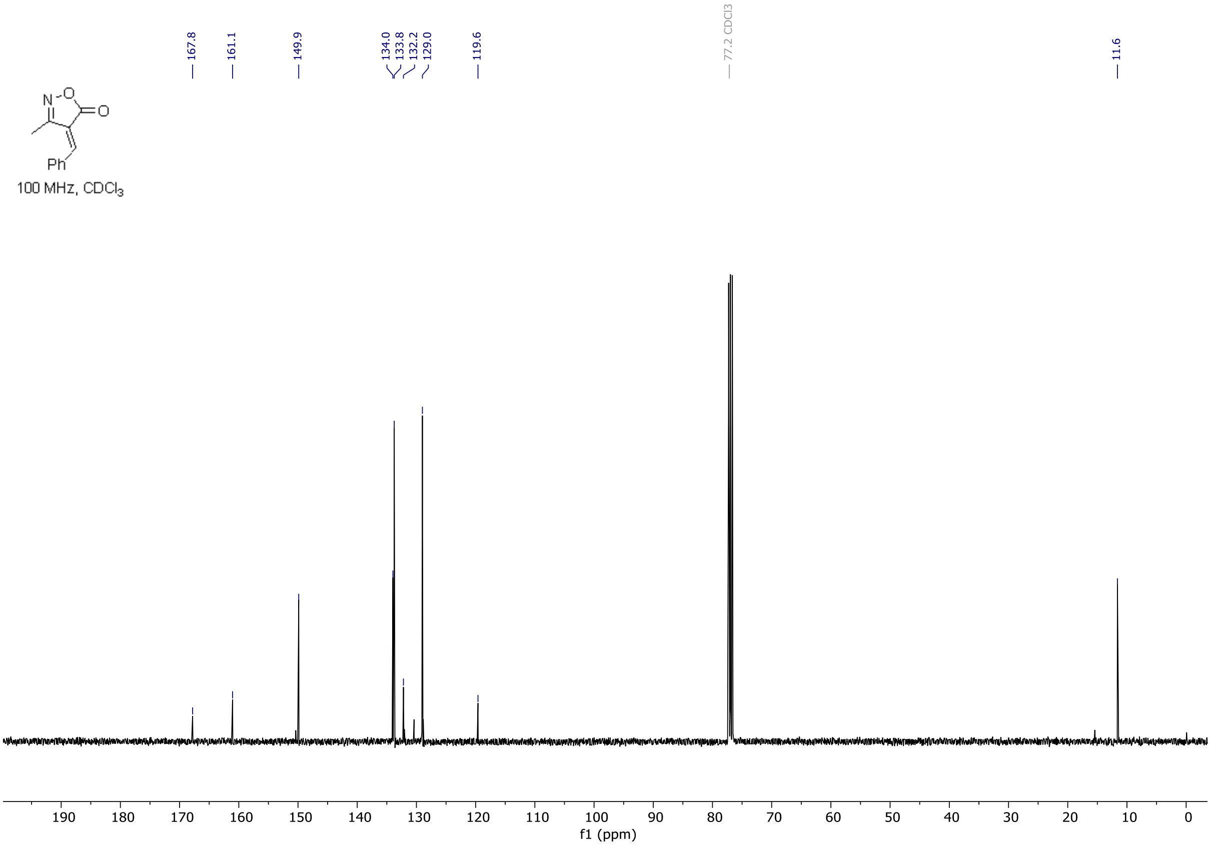

HSQC Data

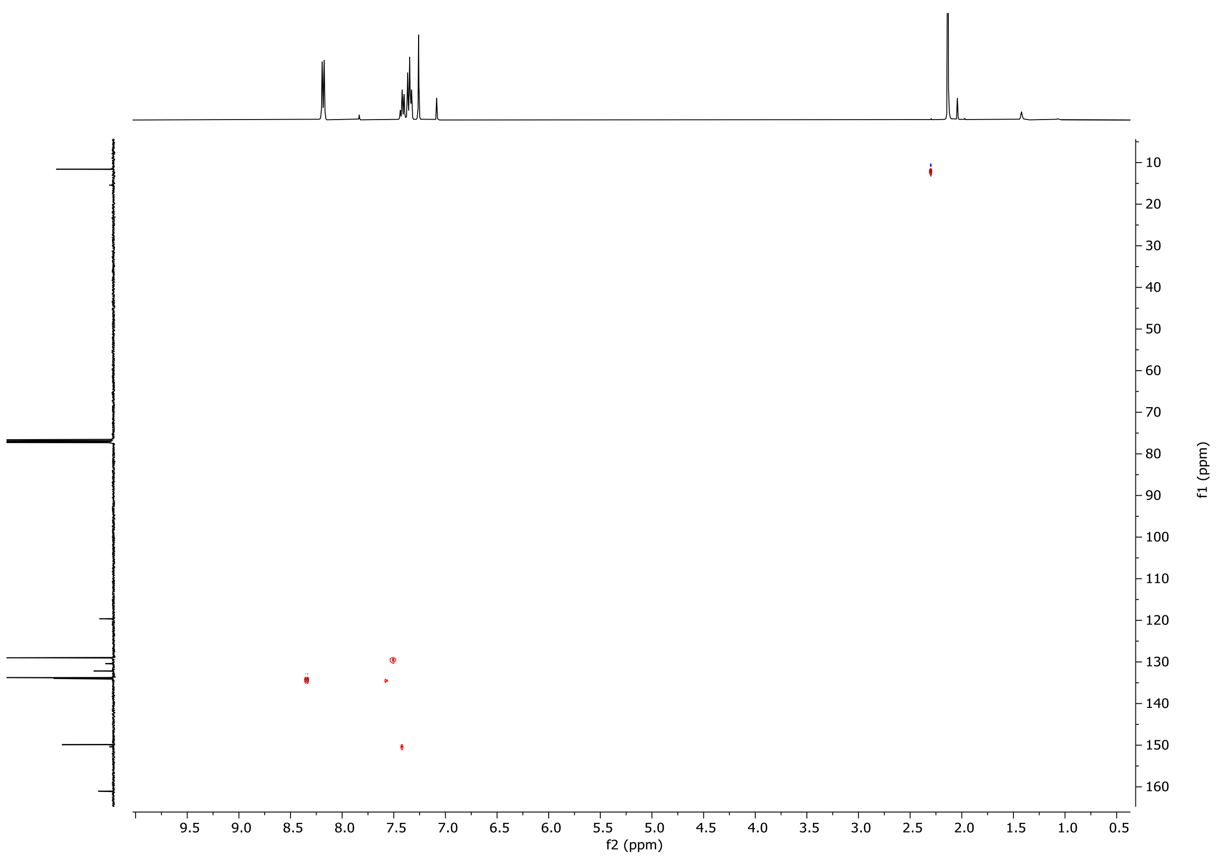

(*E*)-3-Methyl-4-(thiophen-2-ylmethylene)isoxazol-5(4*H*)-one (S2)

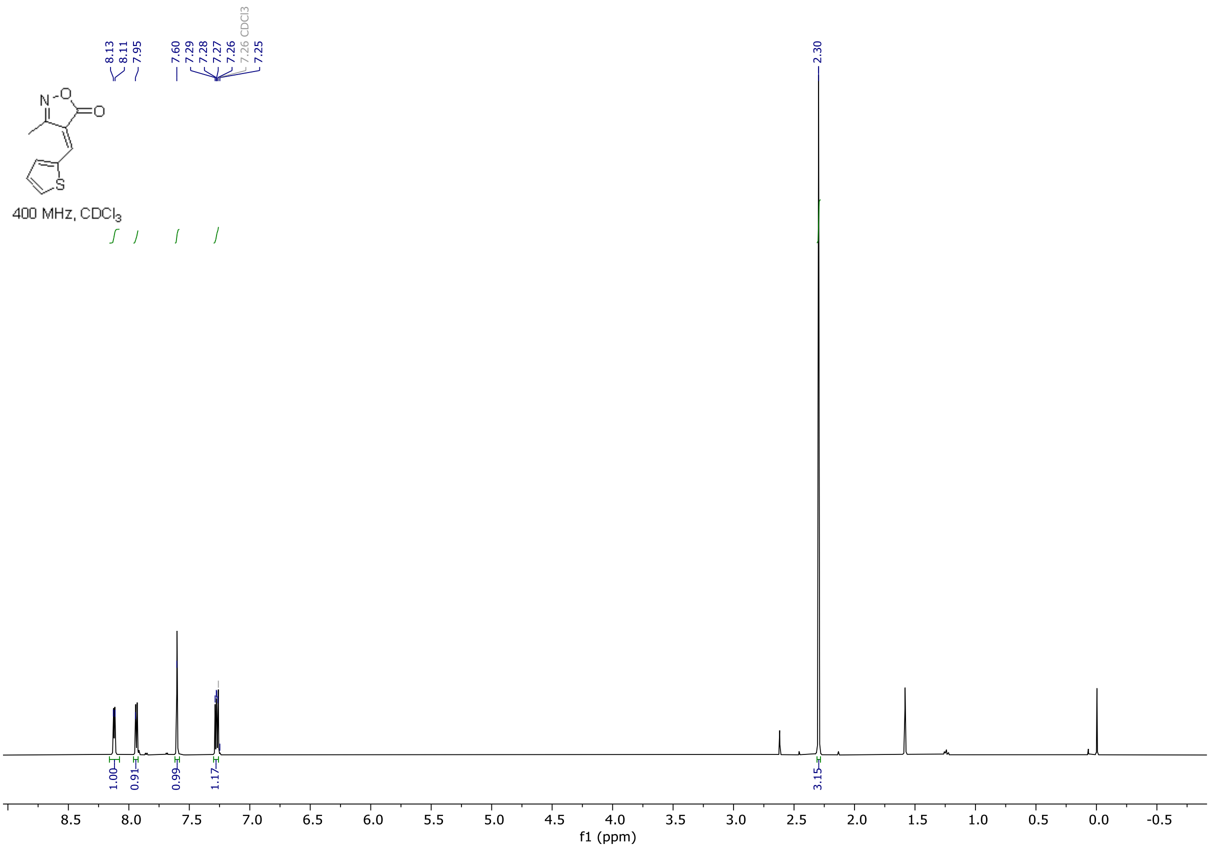

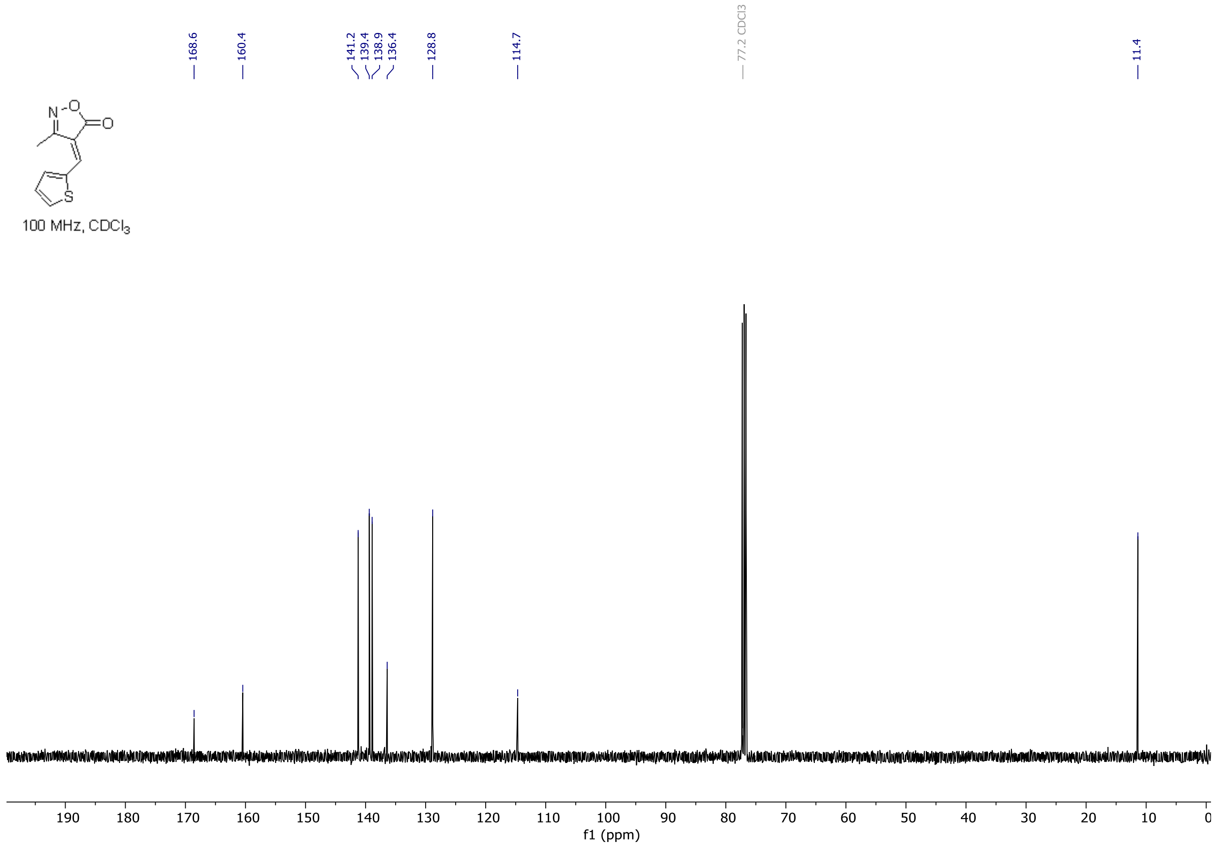
168.6
160.4
141.2
139.4
138.9
136.4
128.8
114.7
77.2 CDCl3
11.4
100 MHz, CDCl3
f1 (ppm)

HSQC Data

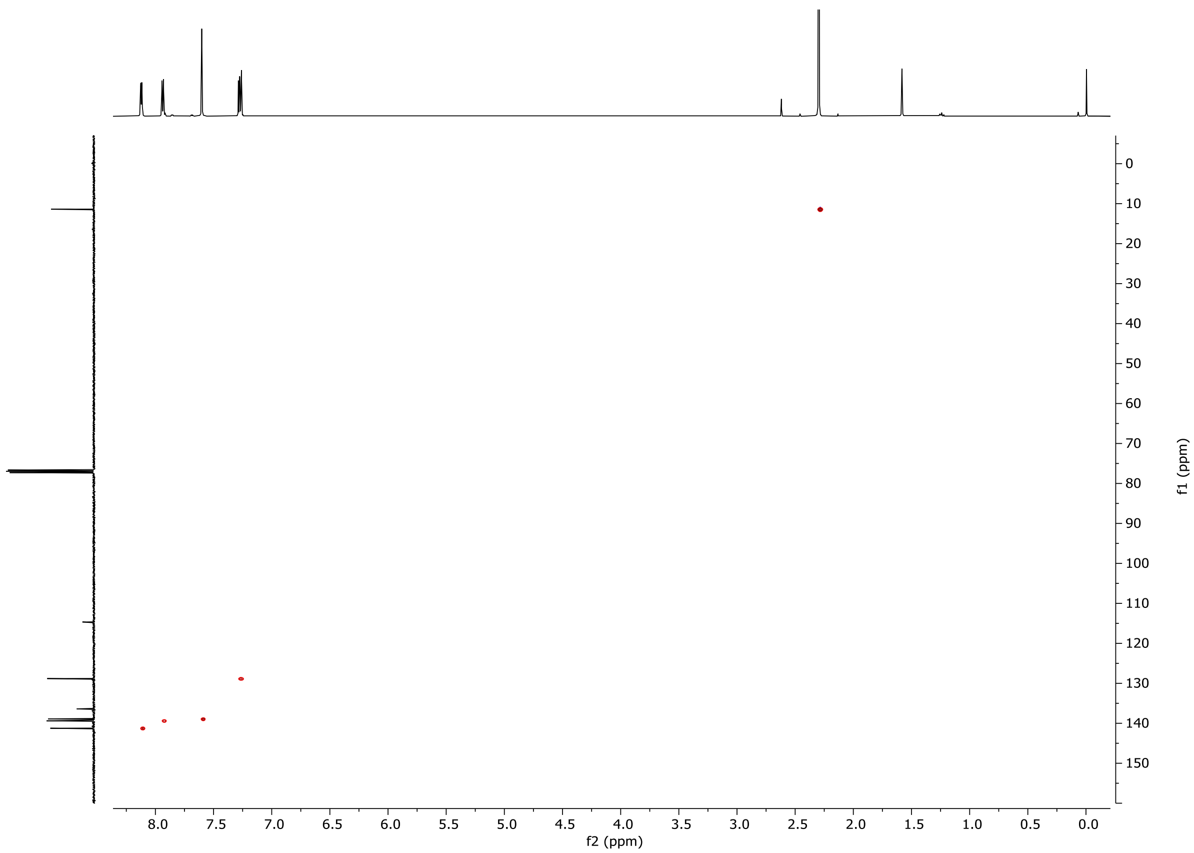
f2 (ppm)
f1 (ppm)

(*E*)-4-((1*H*-Indol-2-yl)methylene)-3-methylisoxazol-5(4*H*)-one (S3)

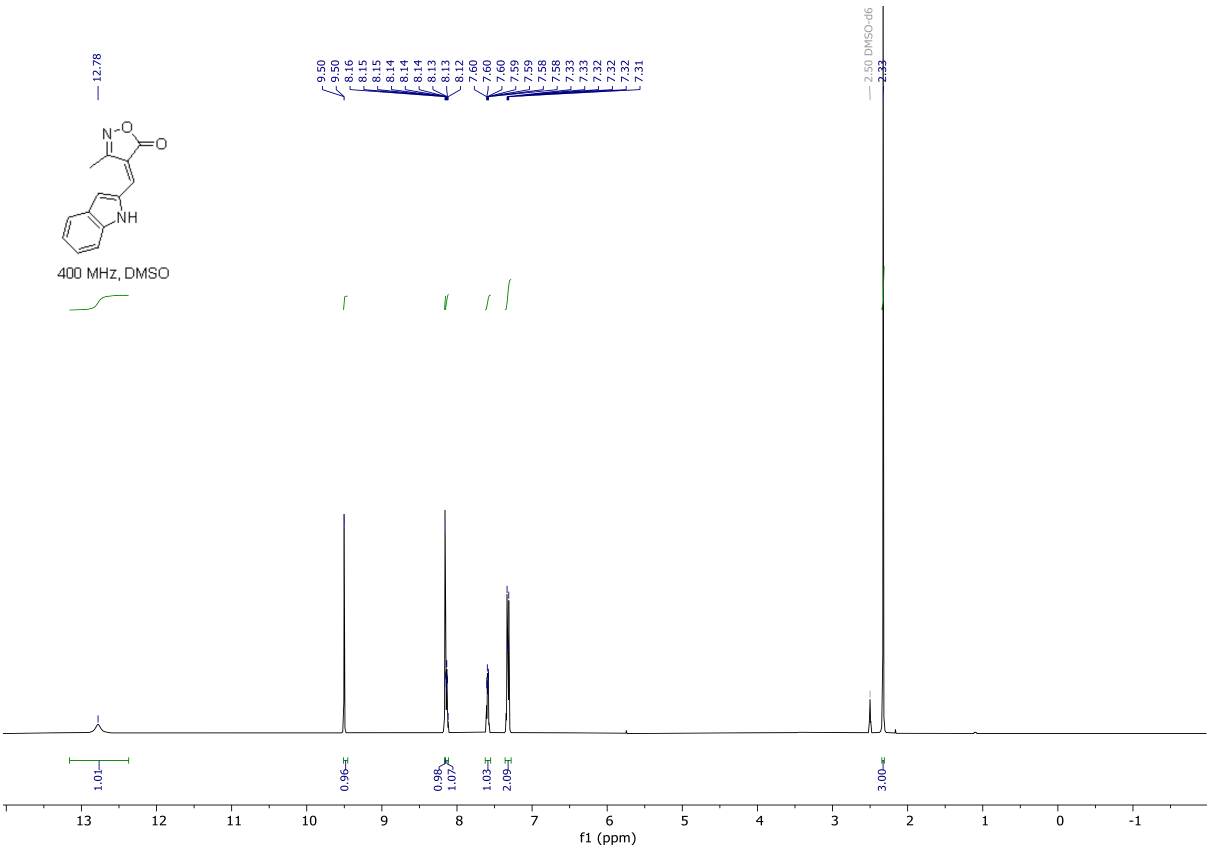

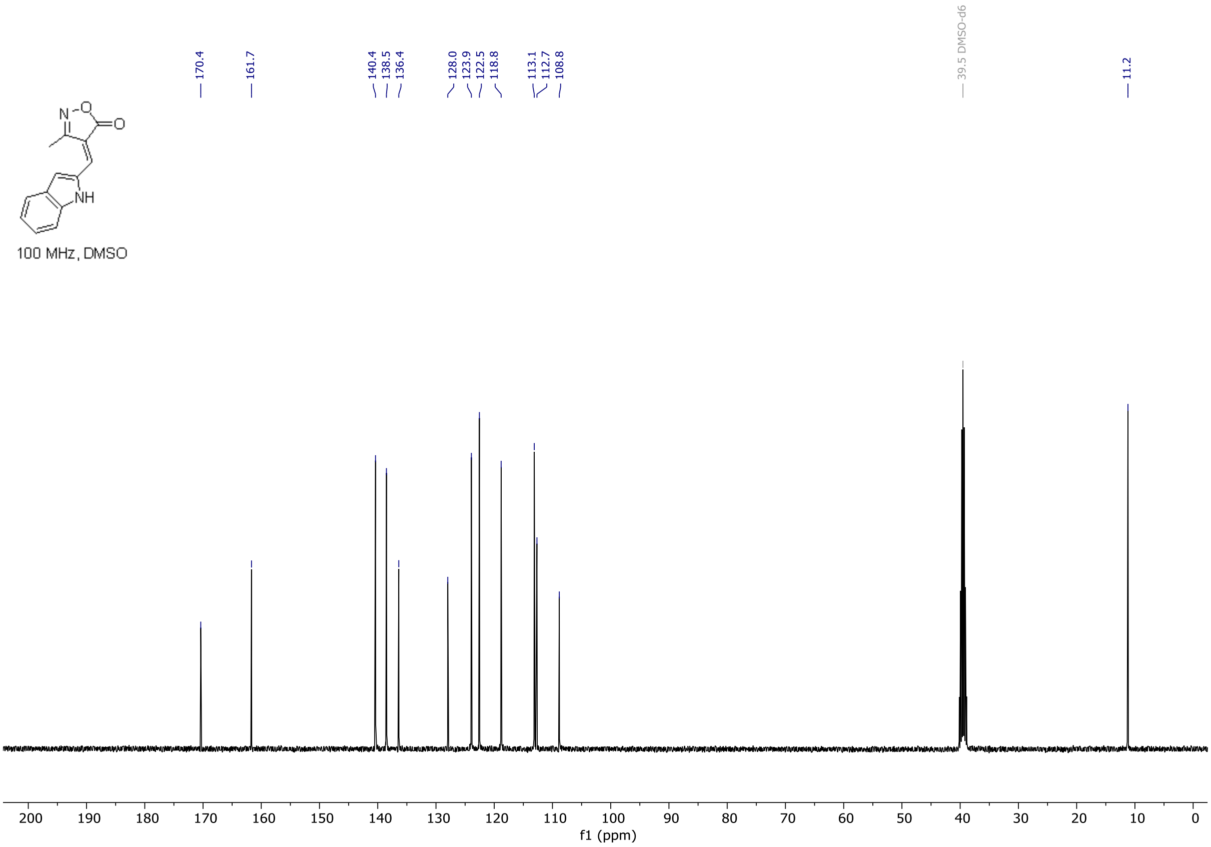

HSQC Data

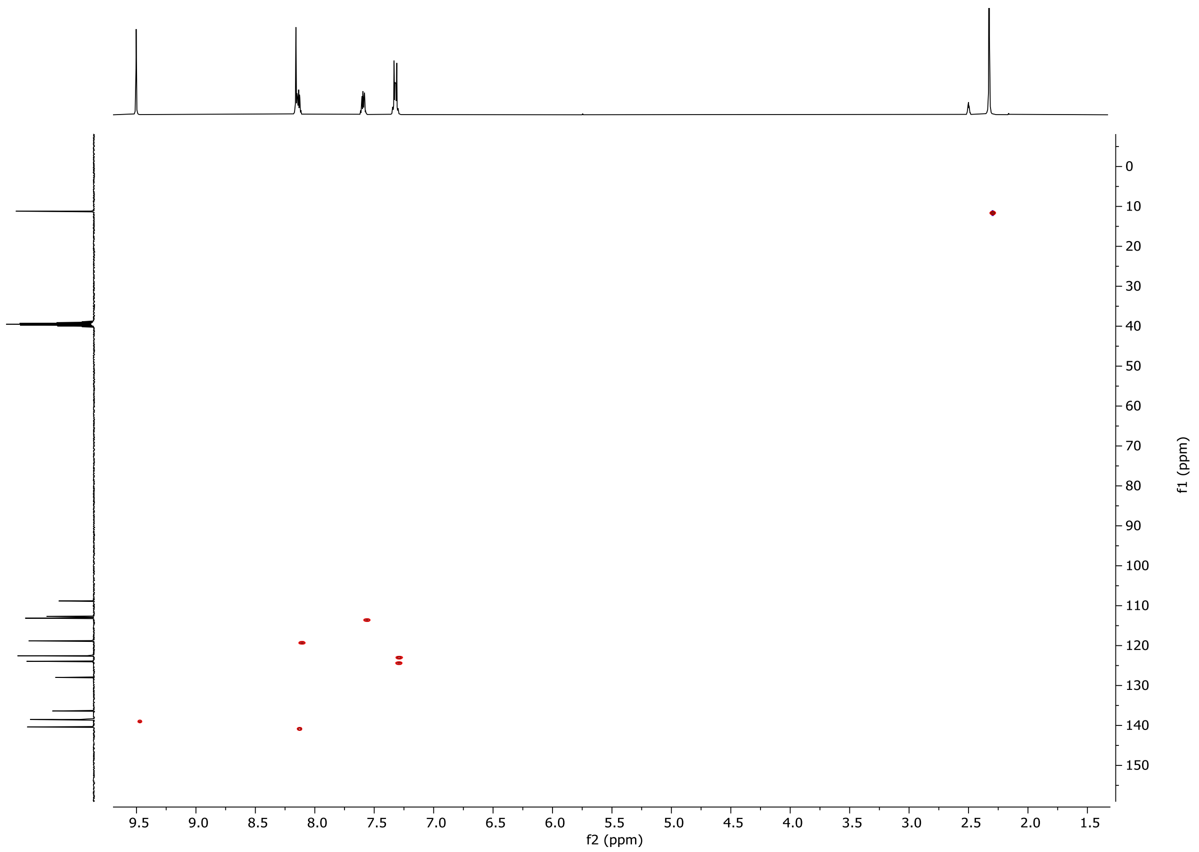

(*E*)-4-(2-Hydroxybenzylidene)-3-methylisoxazol-5(4*H*)-one (S4)

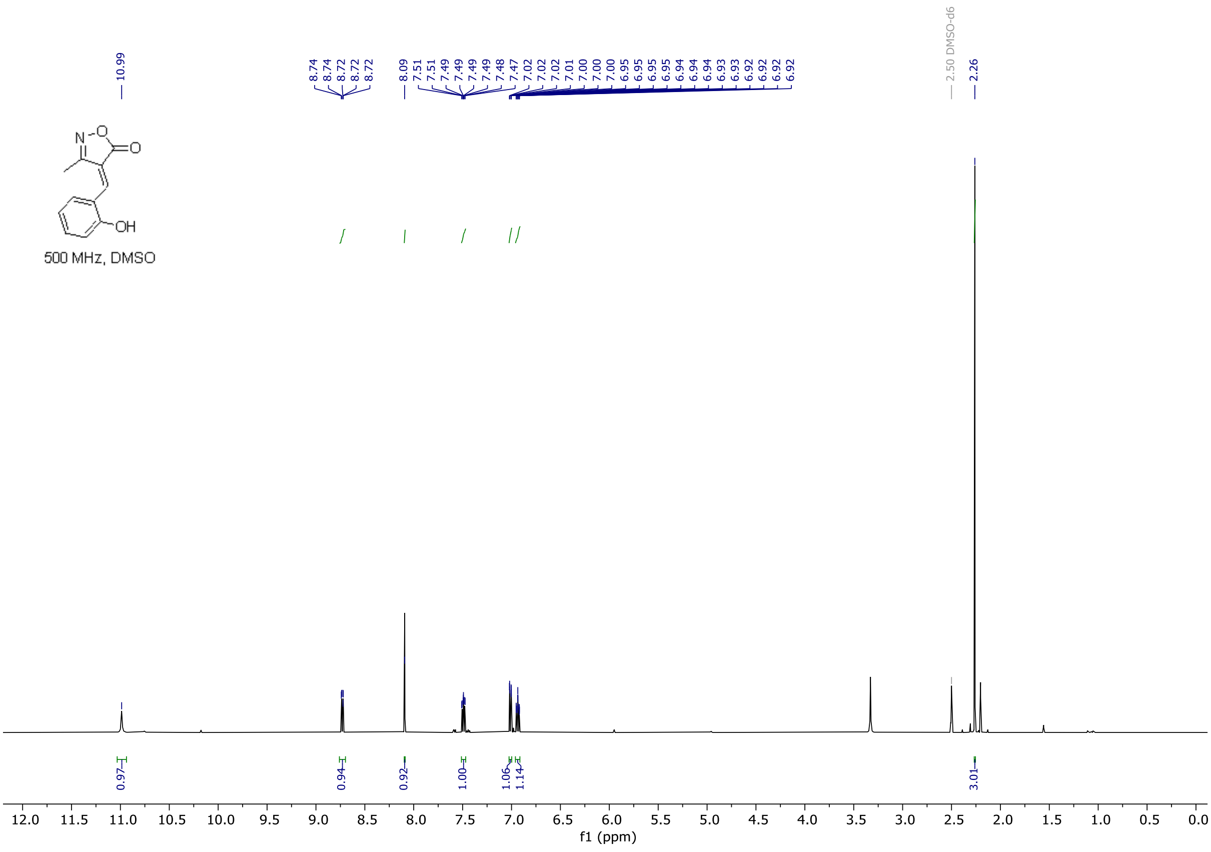

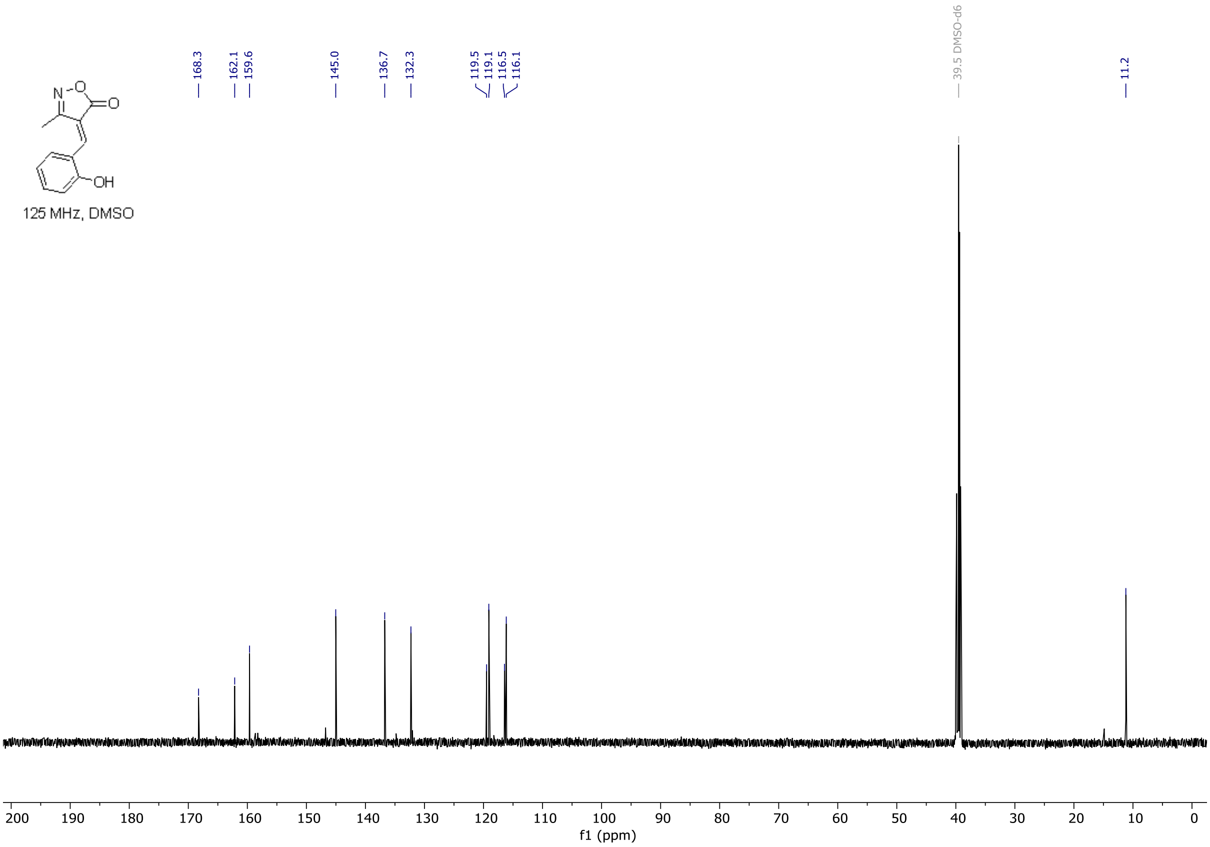
125 MHz, DMSO
N
O
O
OH
168.3
162.1
159.6
145.0
136.7
132.3
119.5
119.1
116.5
116.1
39.5 DMSO-d6
11.2
200 190 180 170 160 150 140 130 120 110 100 90 80 70 60 50 40 30 20 10 0
f1 (ppm)

HSQC Data

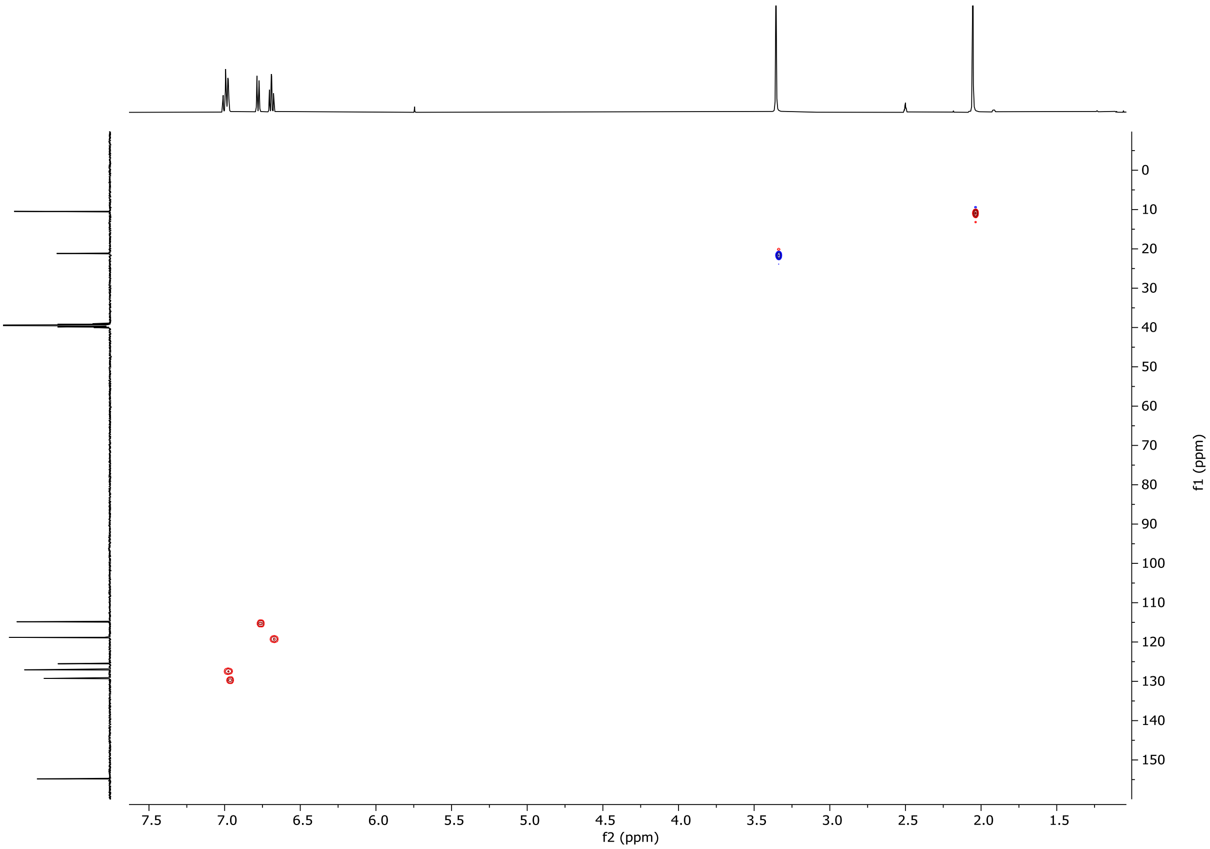
7.5 7.0 6.5 6.0 5.5 5.0 4.5 4.0 3.5 3.0 2.5 2.0 1.5
f2 (ppm)
0 10 20 30 40 50 60 70 80 90 100 110 120 130 140 150
f1 (ppm)

(*E*)-4-(4-Bromobenzylidene)-3-methylisoxazol-5(4*H*)-one (S5)

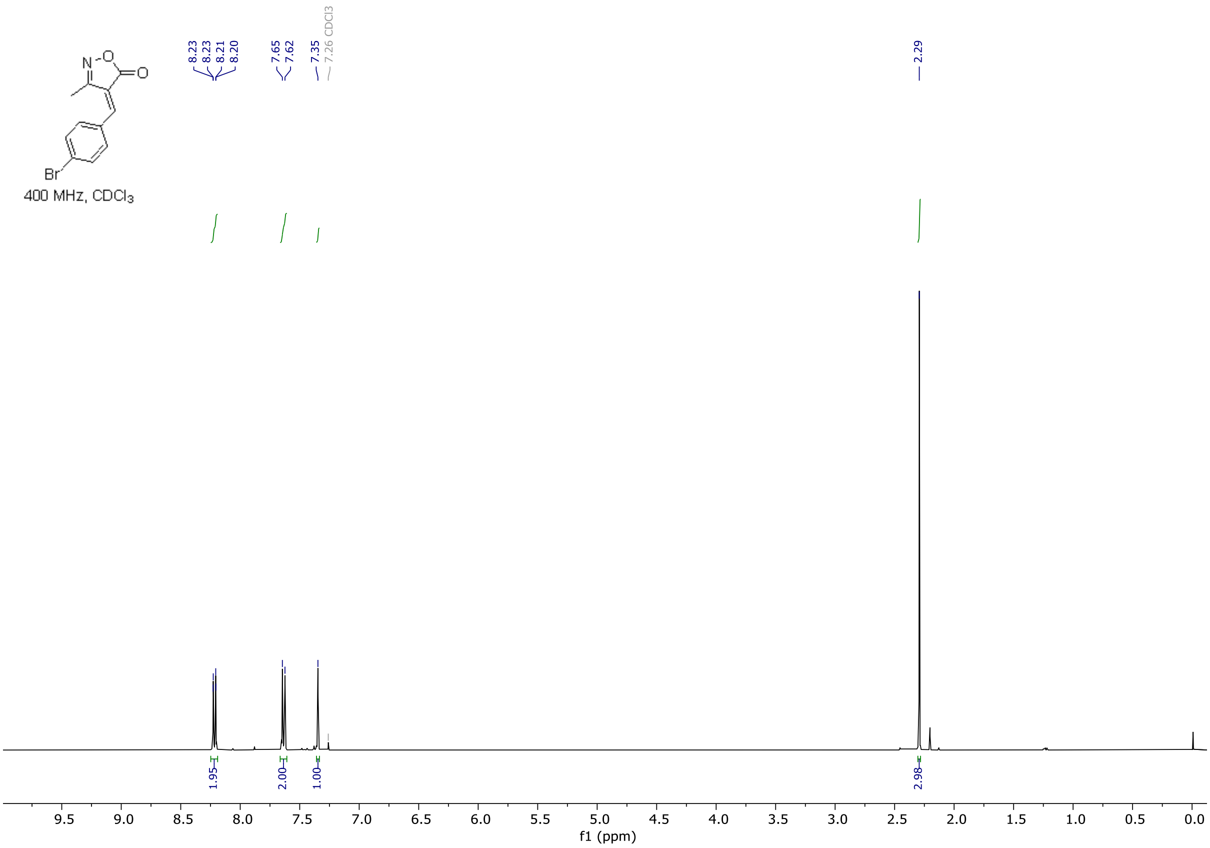

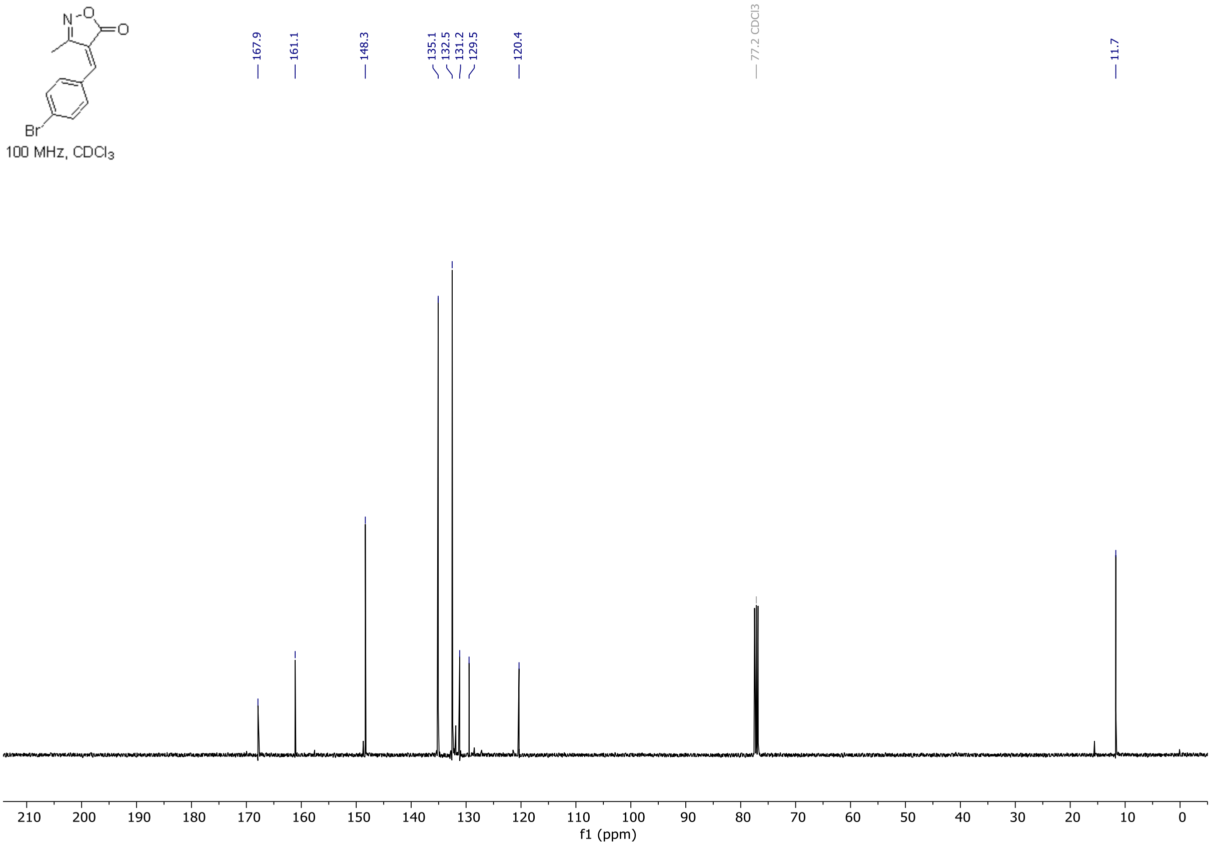

HSQC Data

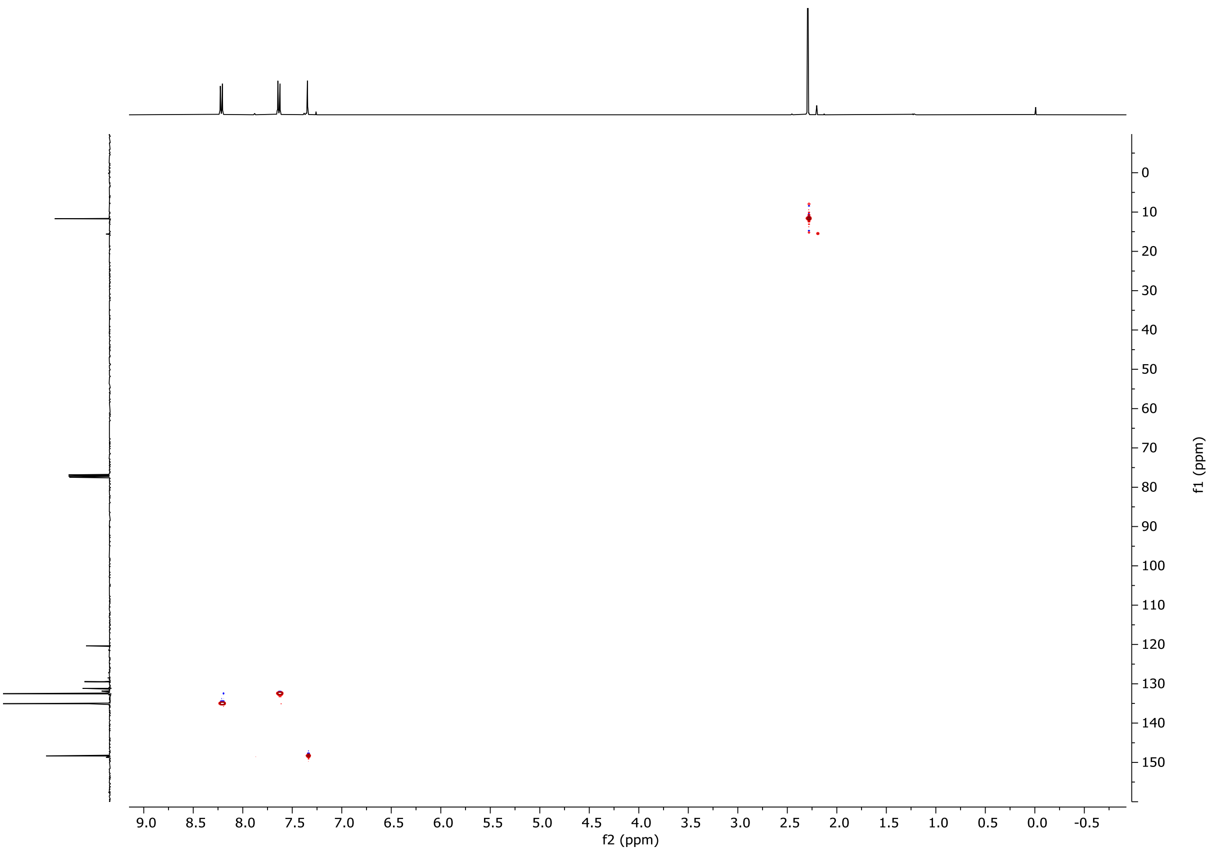

(*E*)-4-Benzylidene-3-cyclopropylisoxazol-5(4*H*)-one (S6)

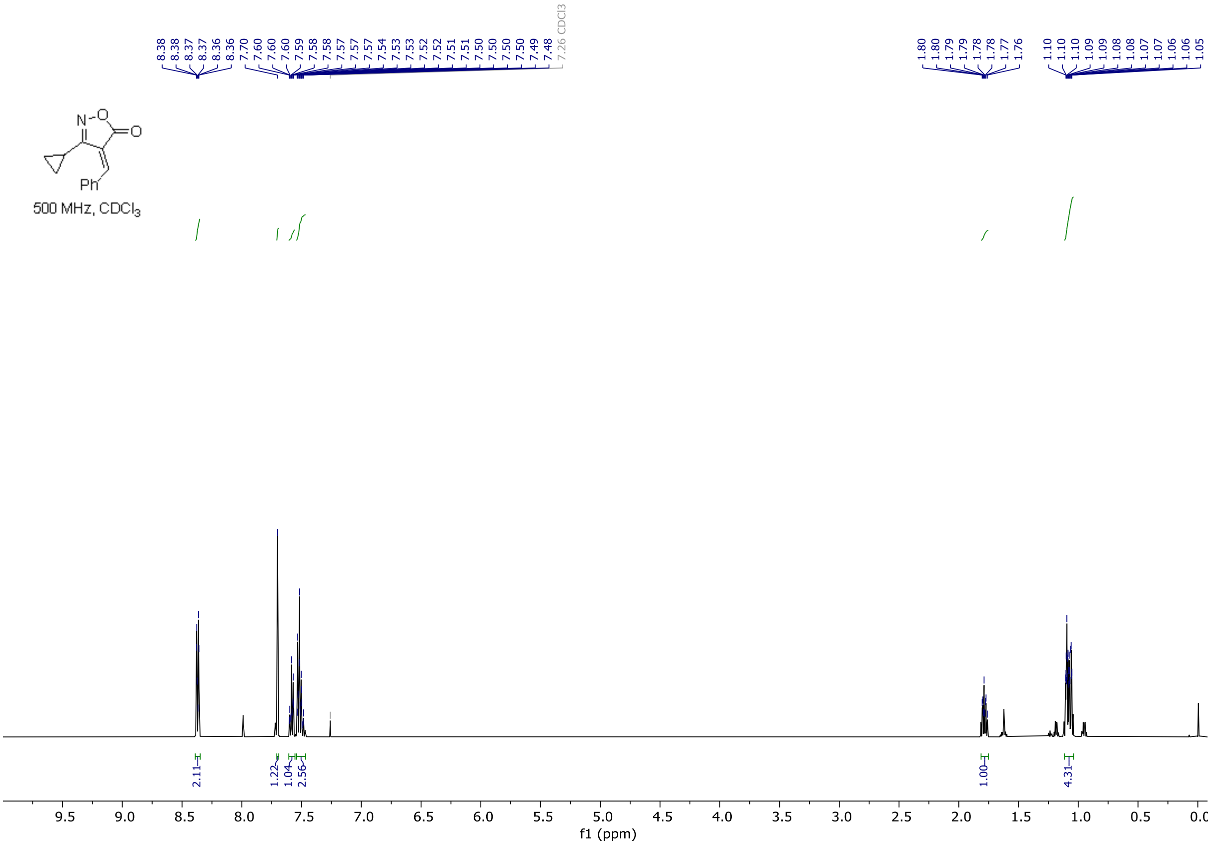

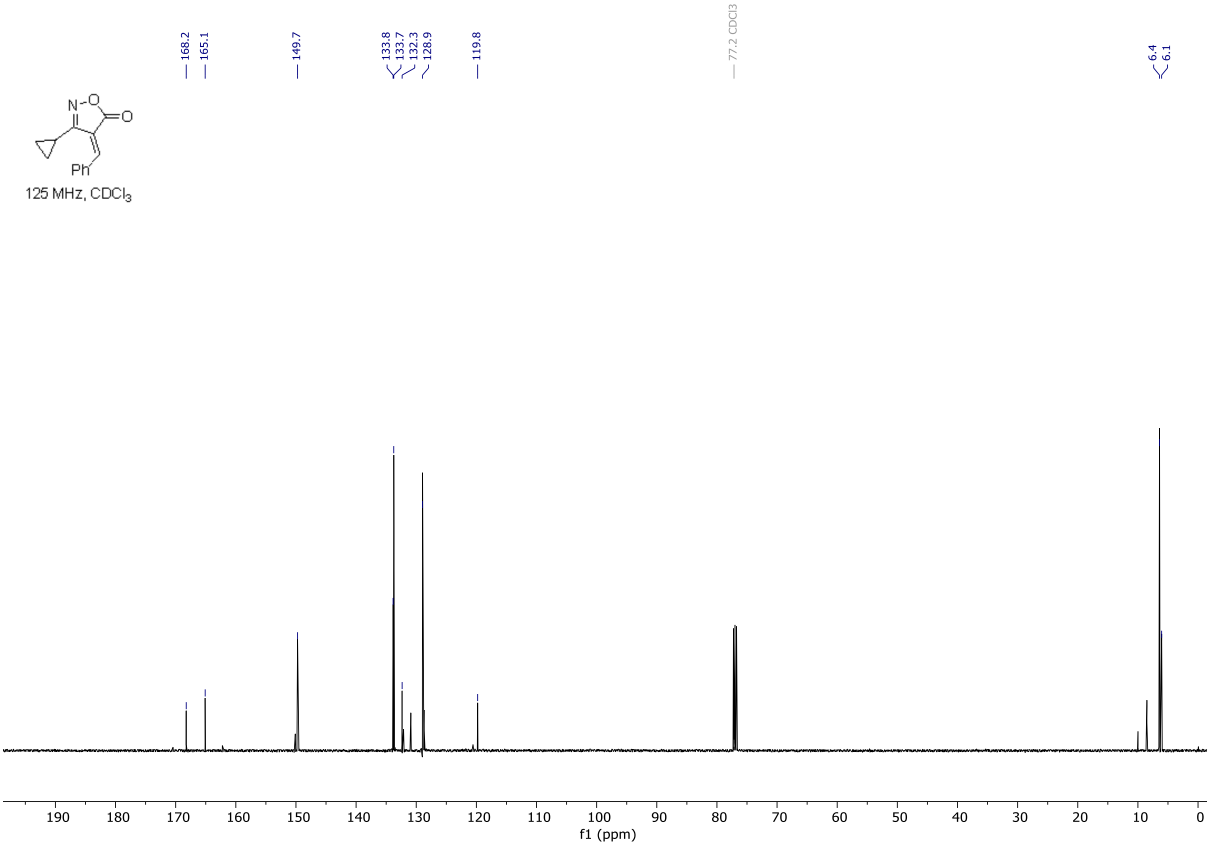

168.2
165.1
149.7
133.8
133.7
132.3
128.9
119.8
77.2 CDCl3
6.4
6.1
125 MHz, CDCl3
f1 (ppm)

HSQC Data

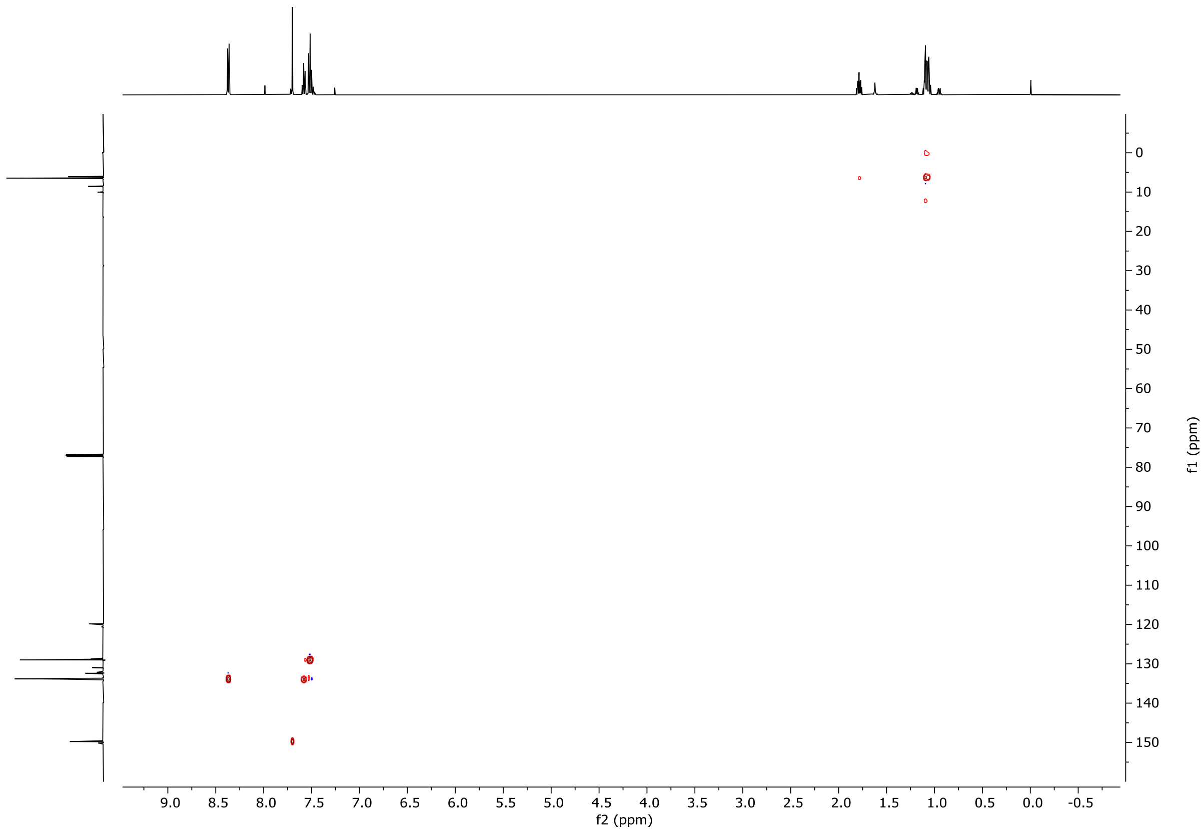

f2 (ppm)
f1 (ppm)

***tert*-butyl(*E*)-2-((3-Methyl-5-oxoisoxazol-4(5*H*)-ylidene)methyl)-1*H*-indole-1-carboxylate (S7)**

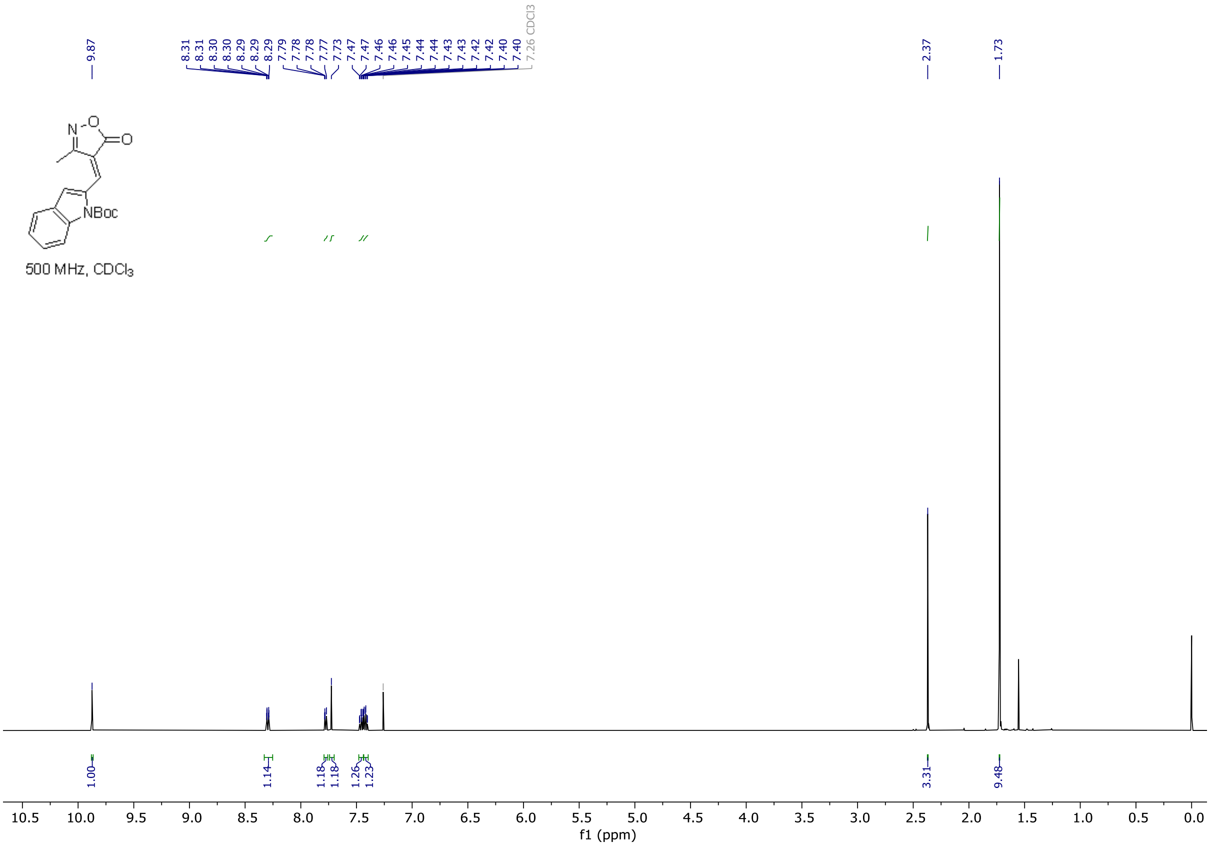

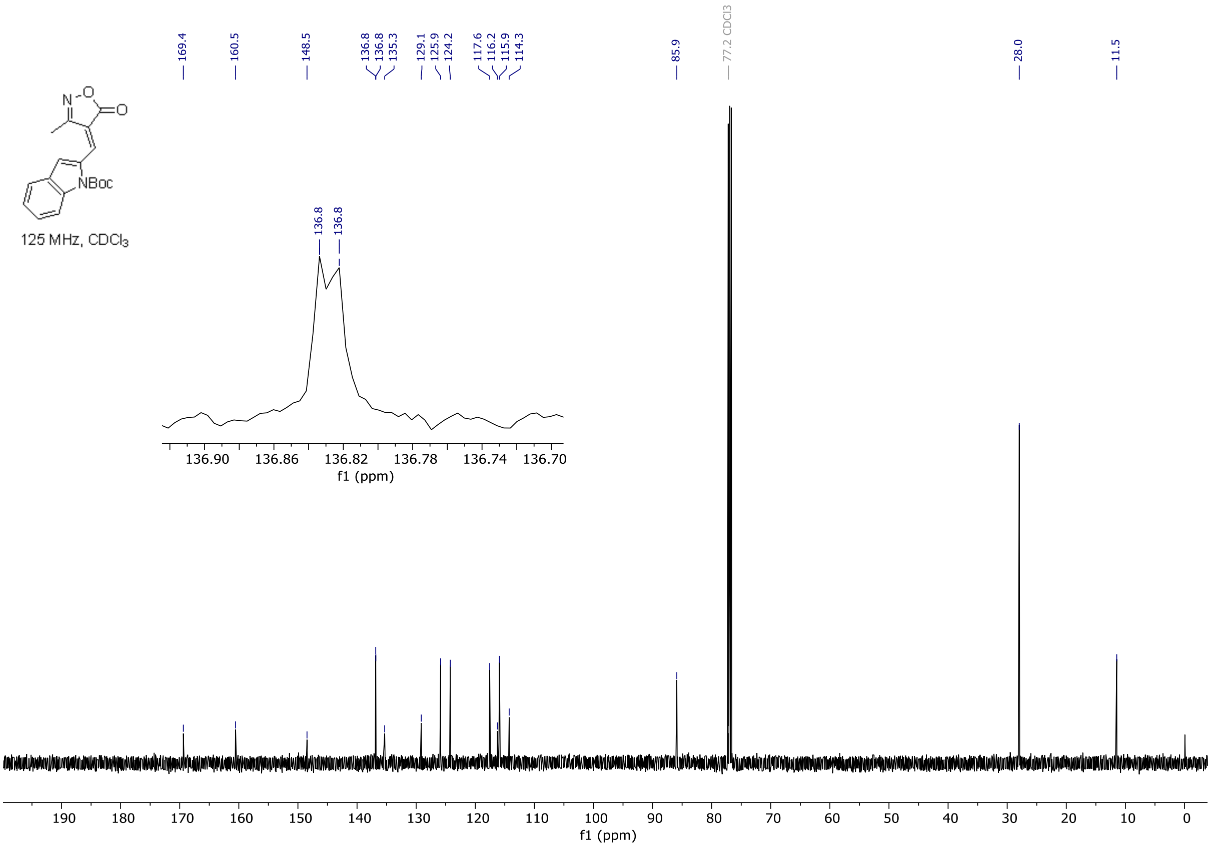

HSQC Data

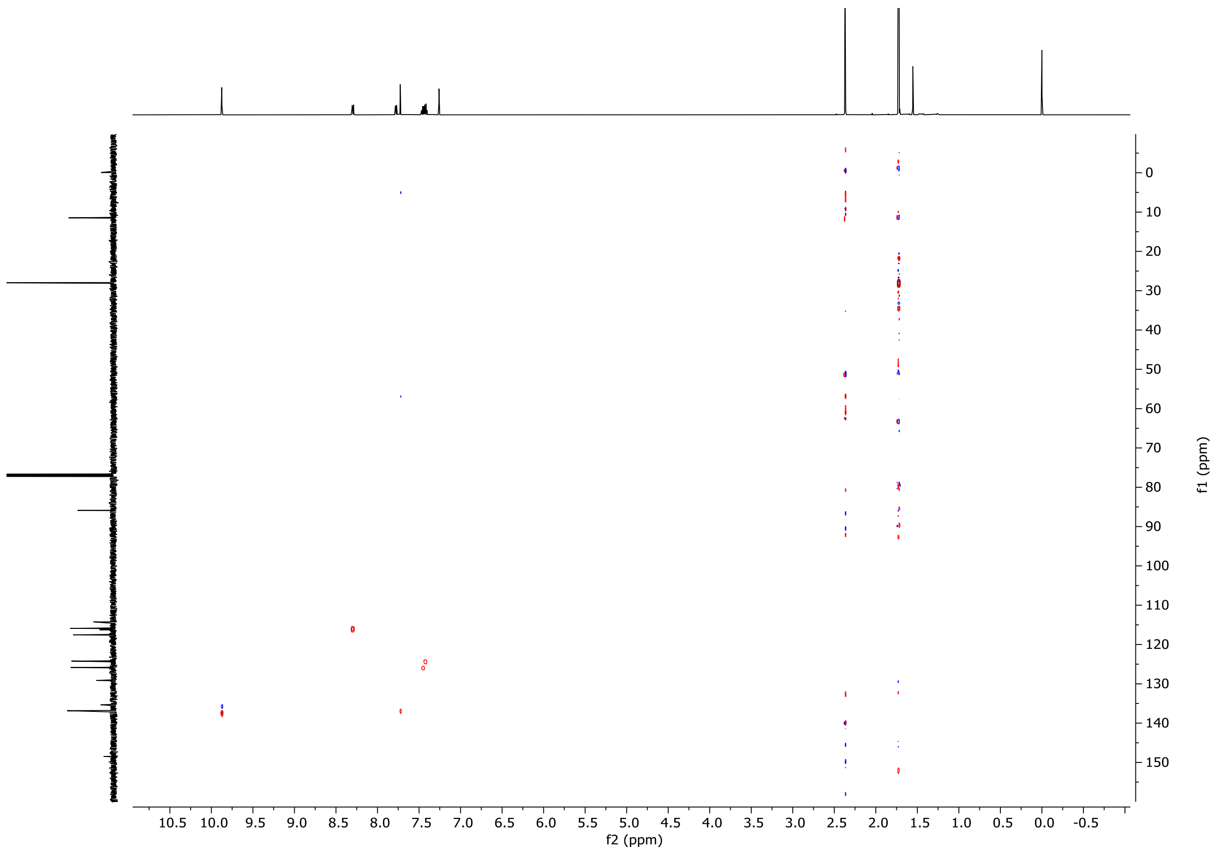

4-Benzyl-3-methylisoxazol-5(2*H*)-one (S8)

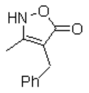

500 MHz, $CDCl_3$

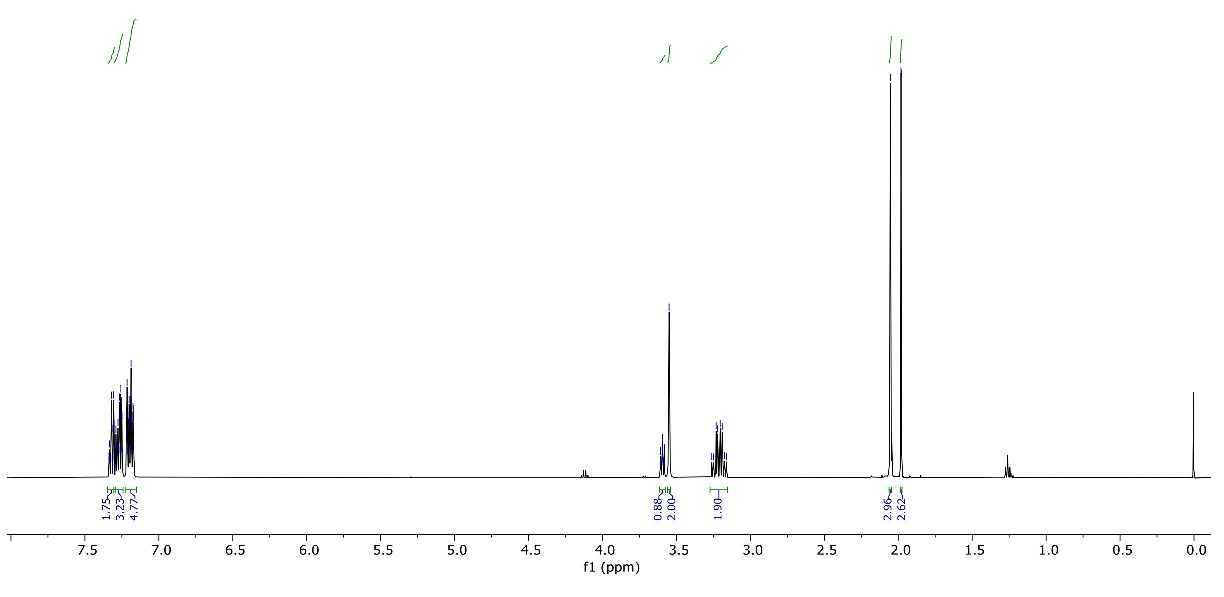

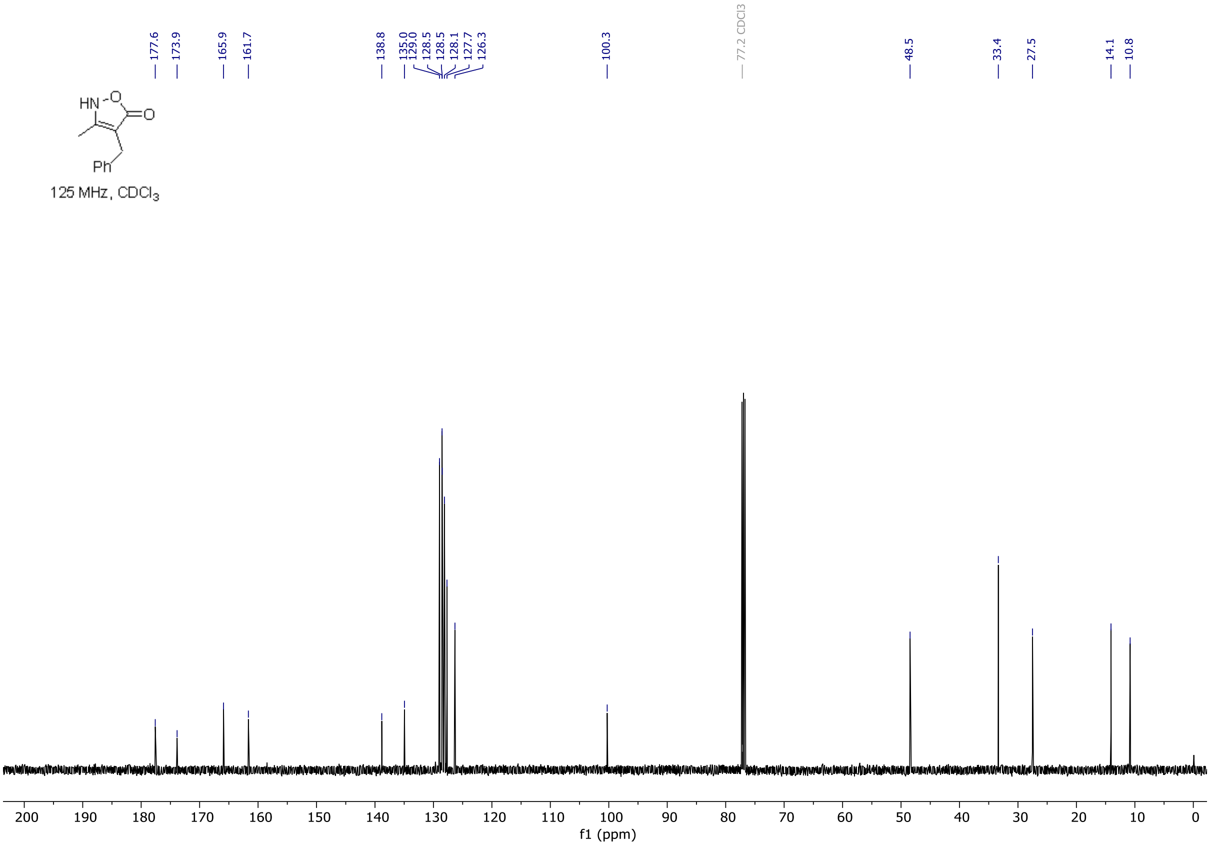
177.6
173.9
165.9
161.7
138.8
135.0
129.0
128.5
128.5
128.1
127.7
126.3
100.3
77.2 CDCl3
48.5
33.4
27.5
14.1
10.8
HN
O
O
Ph
125 MHz, CDCl3
f1 (ppm)

HSQC Data

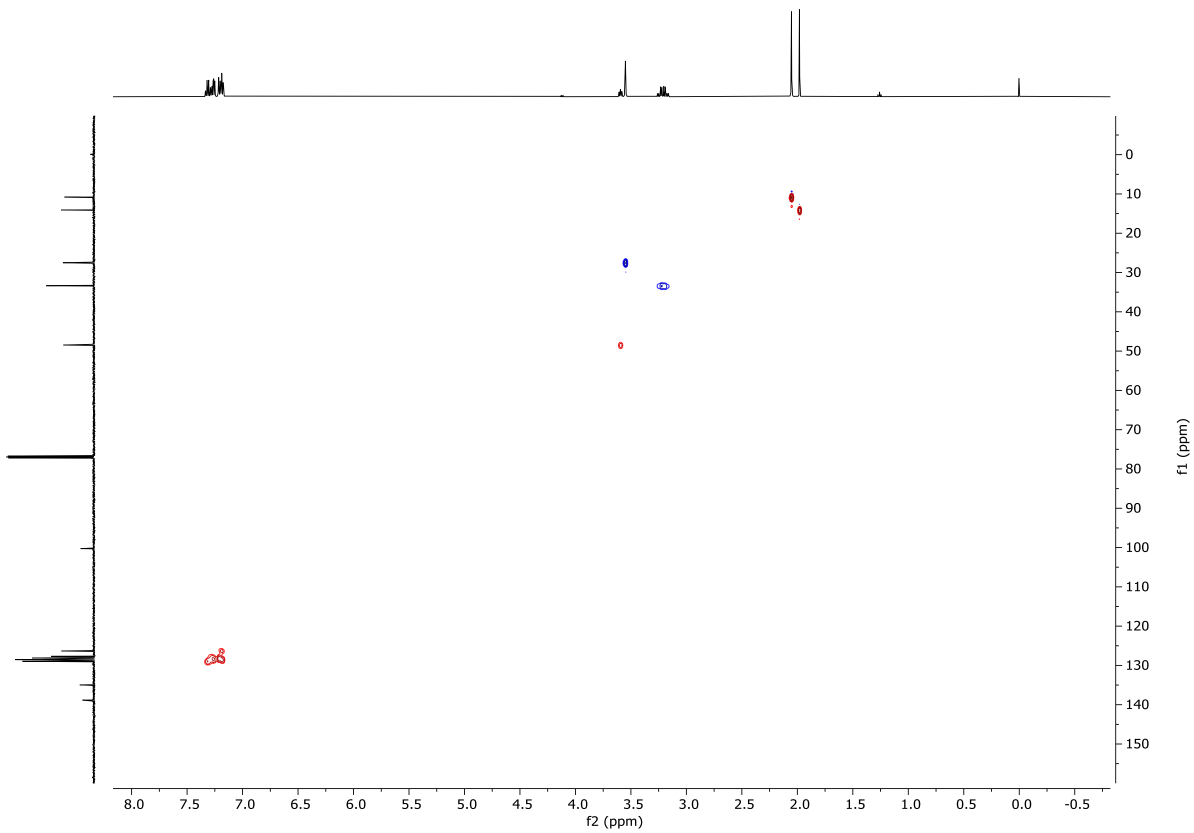
f2 (ppm)
f1 (ppm)

3-Methyl-4-(thiophen-2-ylmethyl)isoxazol-5(2*H*)-one (S9)

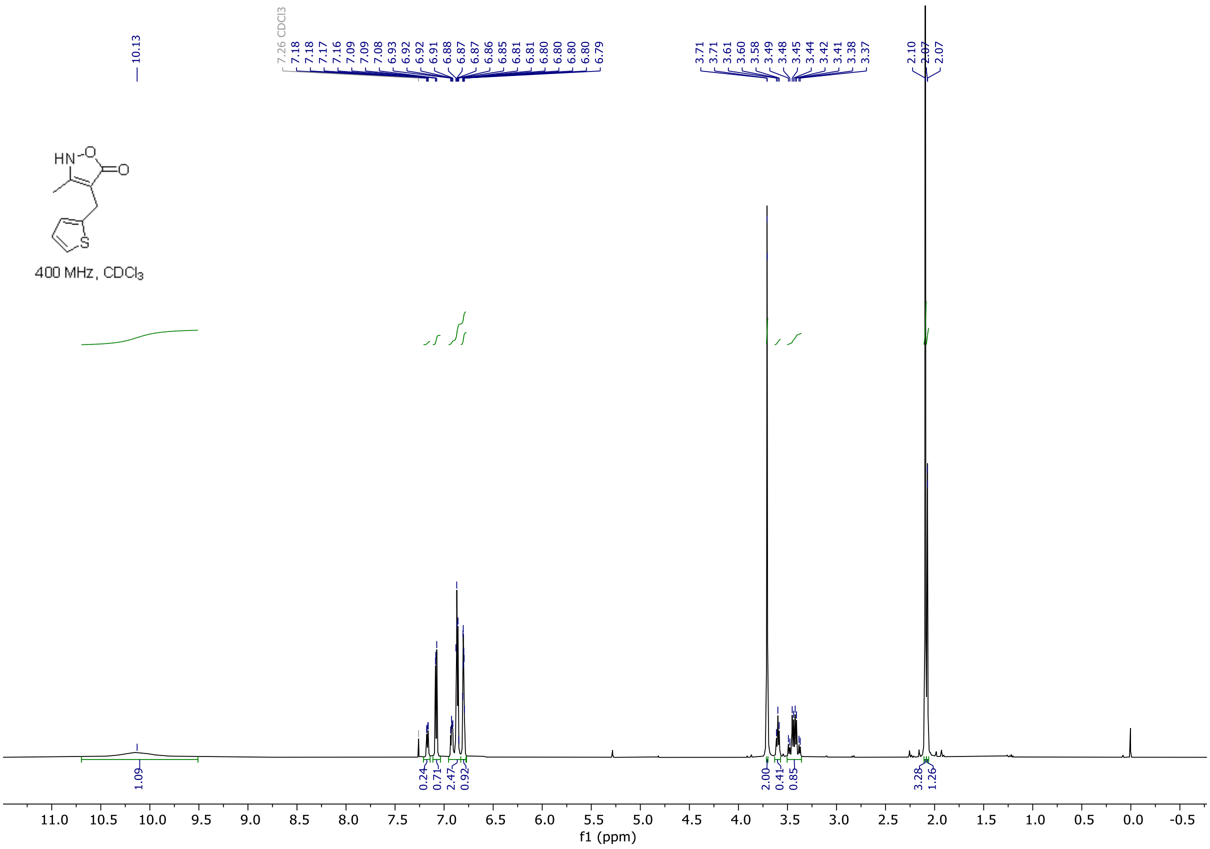

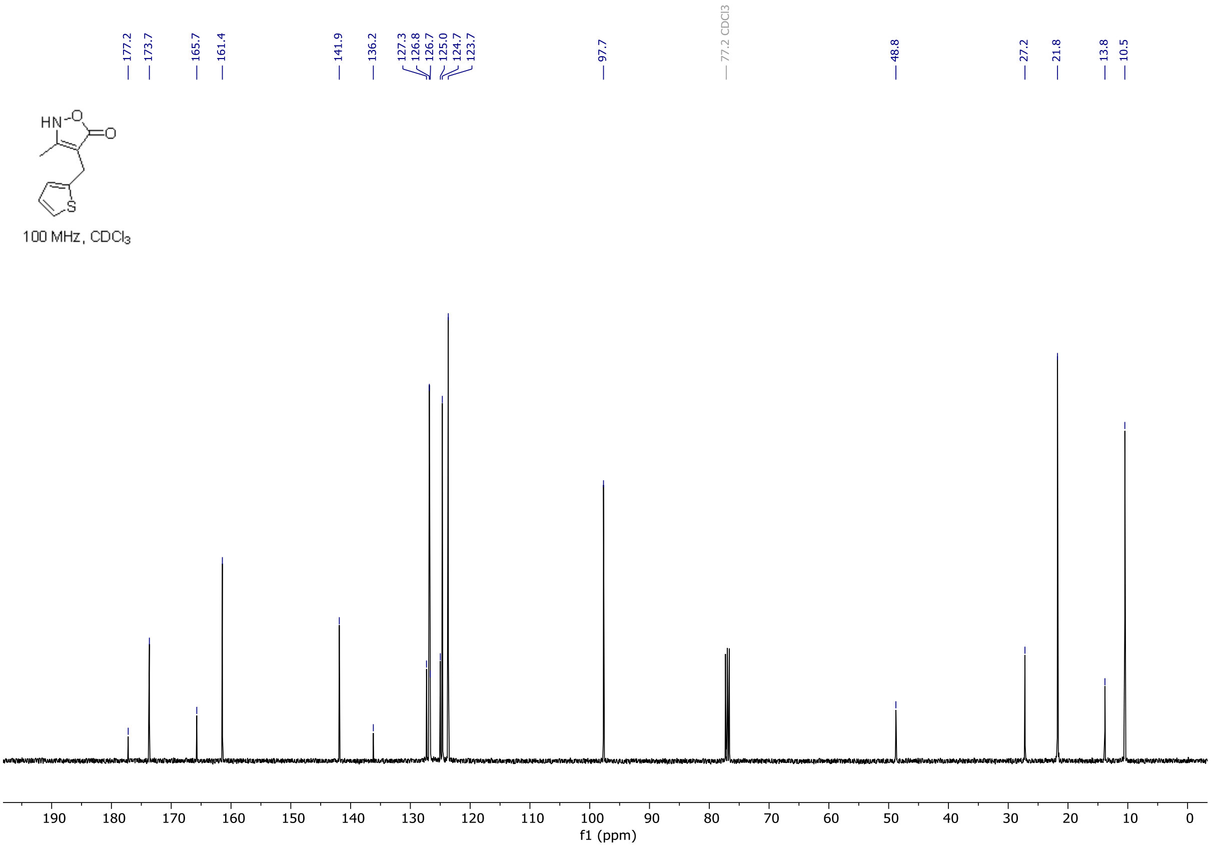

HSQC Data

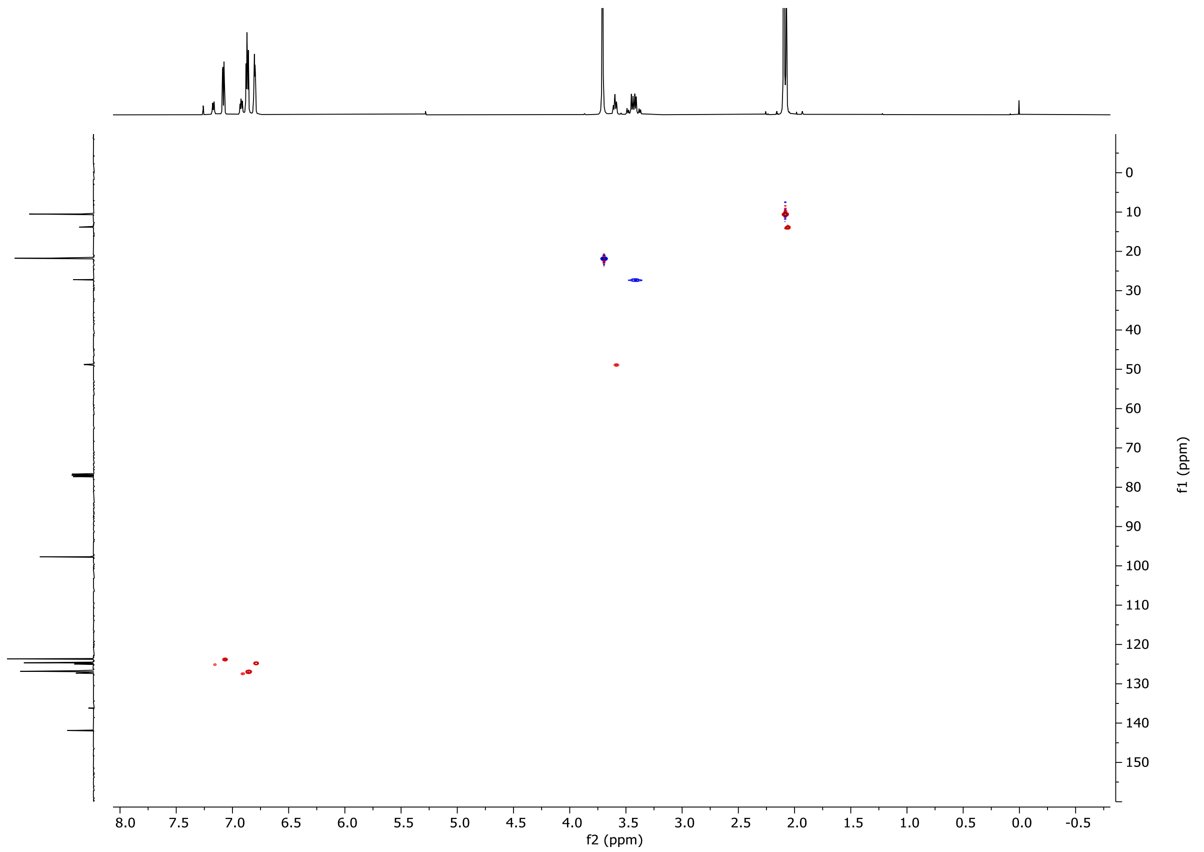

4-(4-Bromobenzyl)-3-methylisoxazol-5(2*H*)-one (S10)

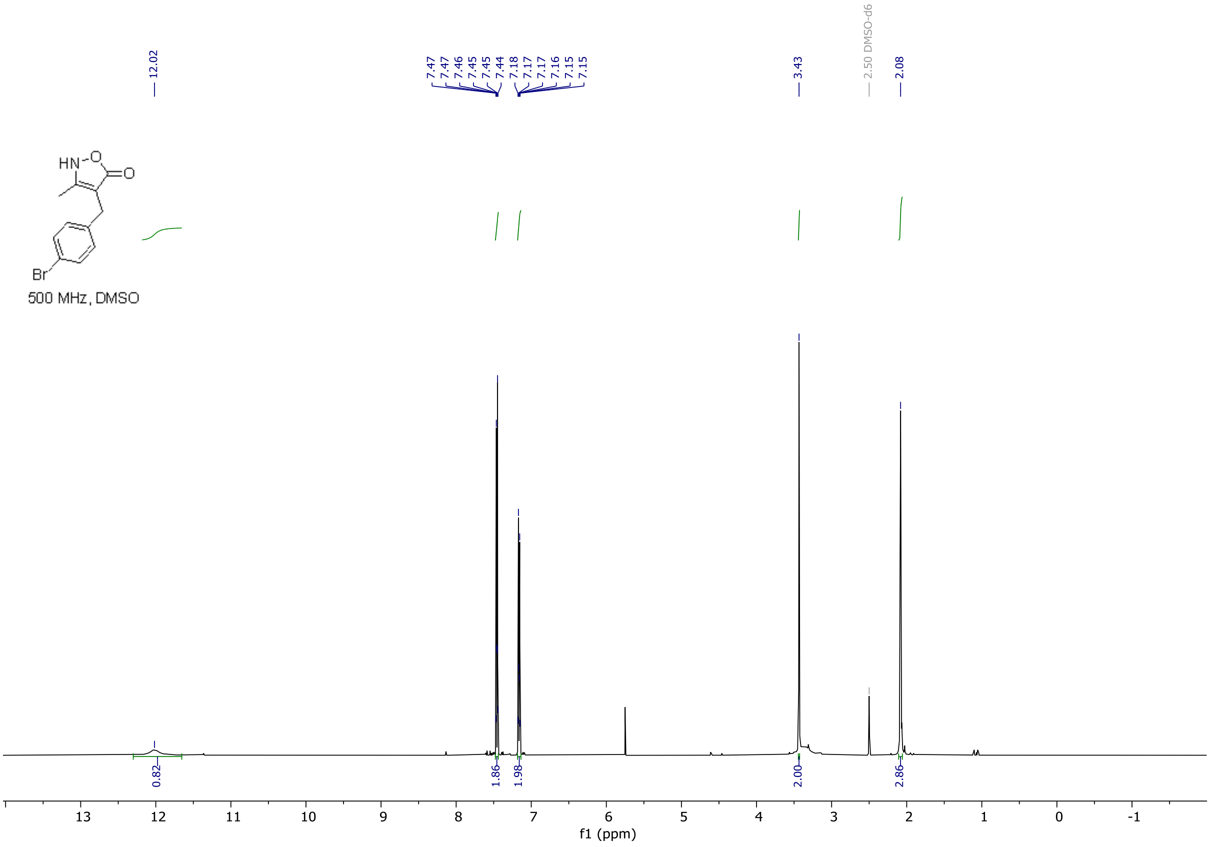

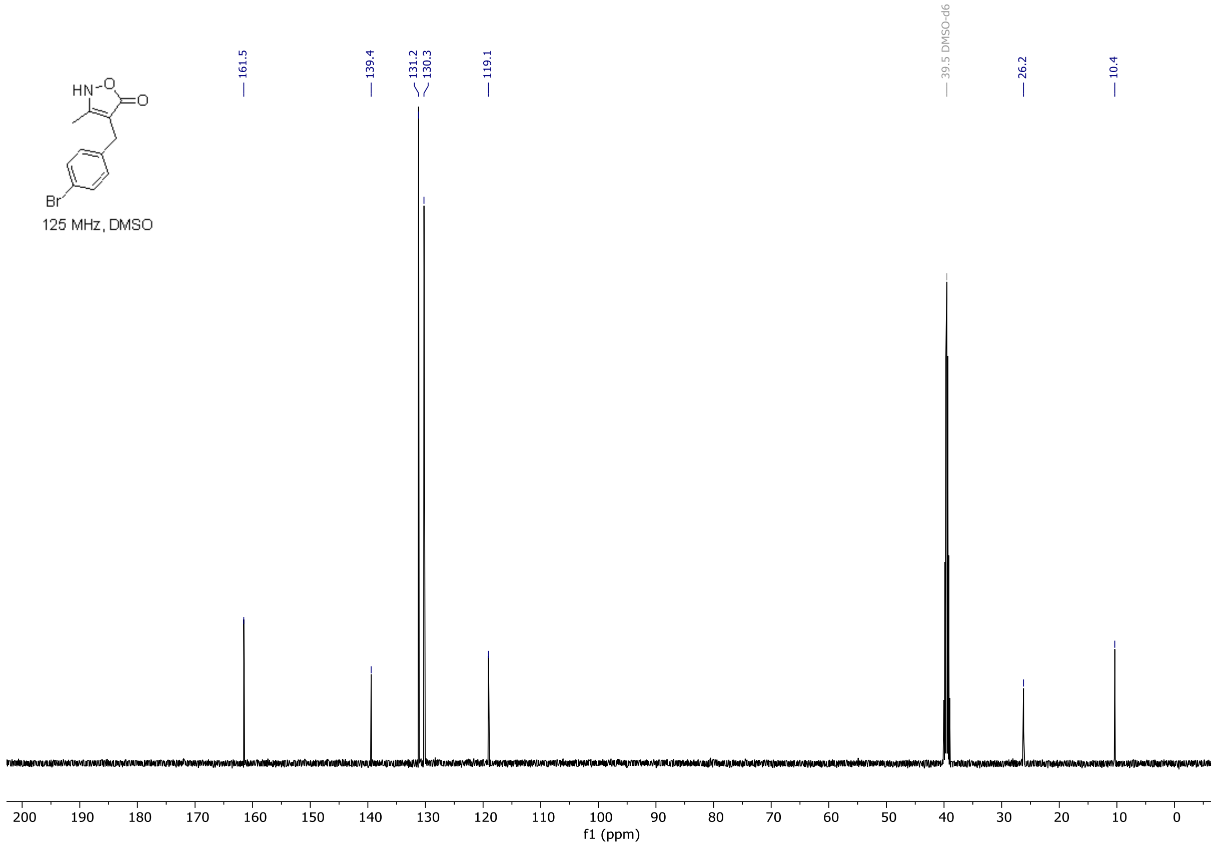
HN
O
O
Br
125 MHz, DMSO
161.5
139.4
131.2
130.3
119.1
39.5 DMSO-d6
26.2
10.4
f1 (ppm)

HSQC Data

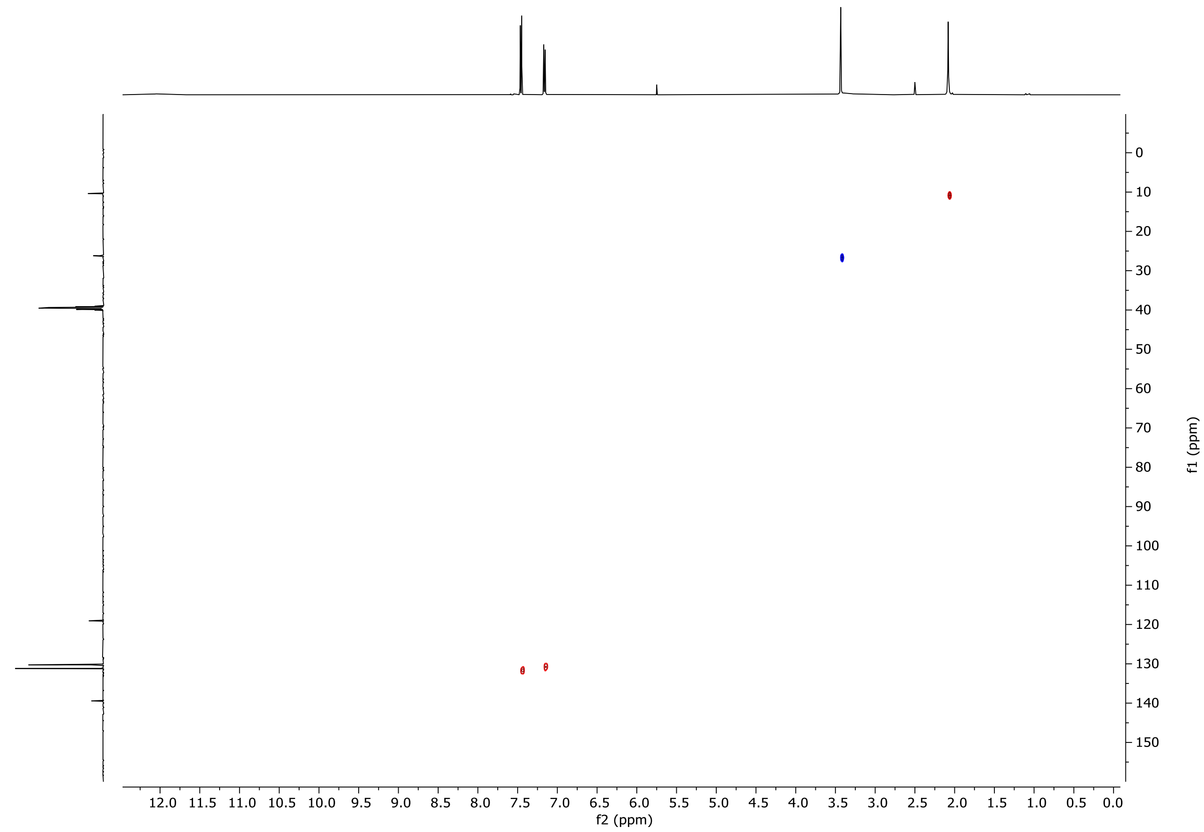
f2 (ppm)
f1 (ppm)

4-Benzyl-3-cyclopropylisoxazol-5(2*H*)-one (S11)

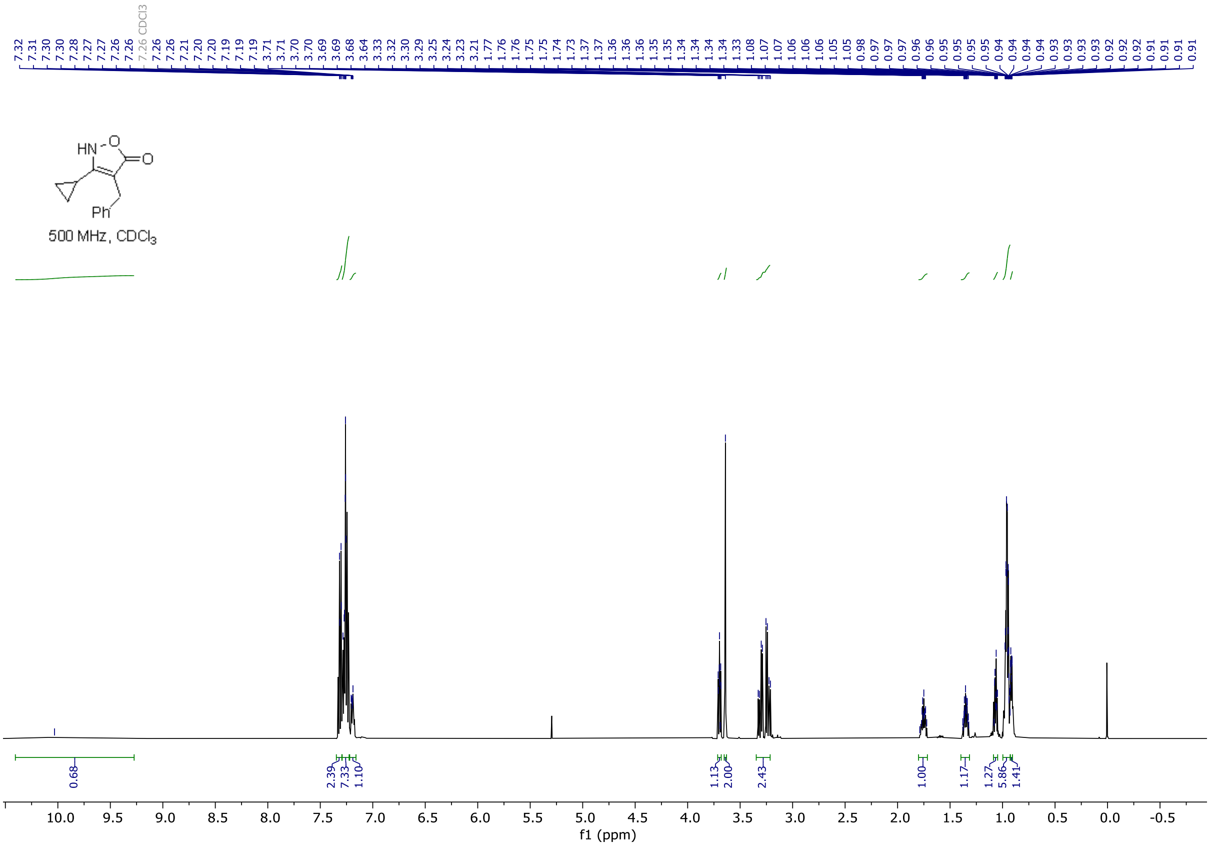

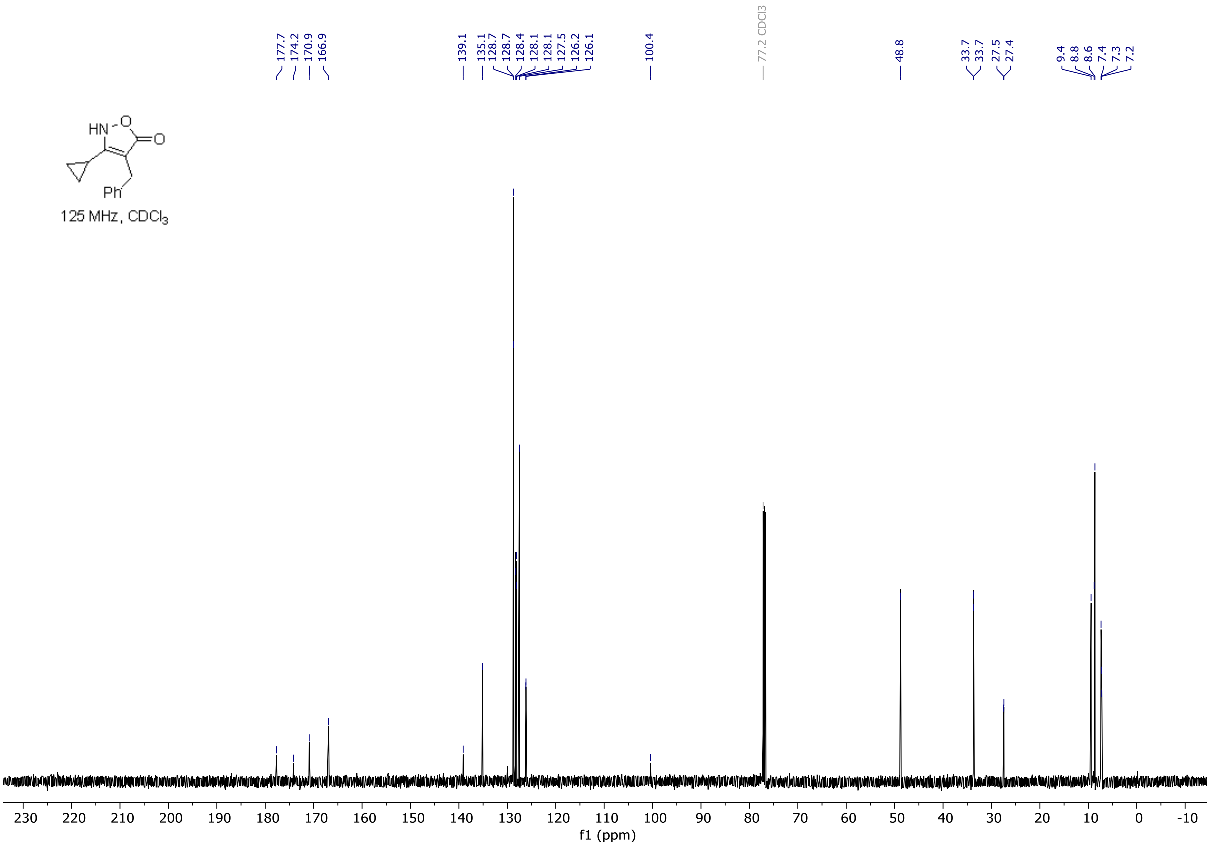

HSQC Data

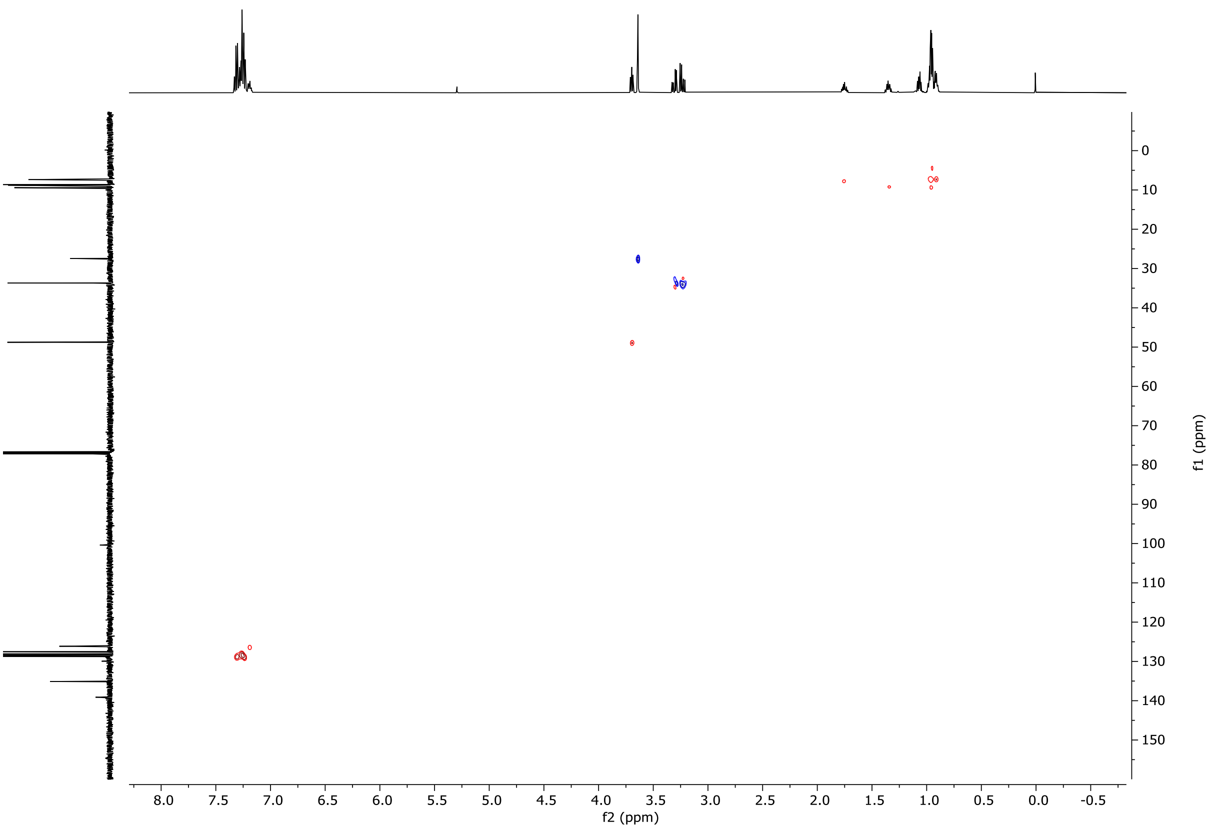

4-((1*H*-indol-2-yl)methyl)-3-methylisoxazol-5(2*H*)-one (S12)

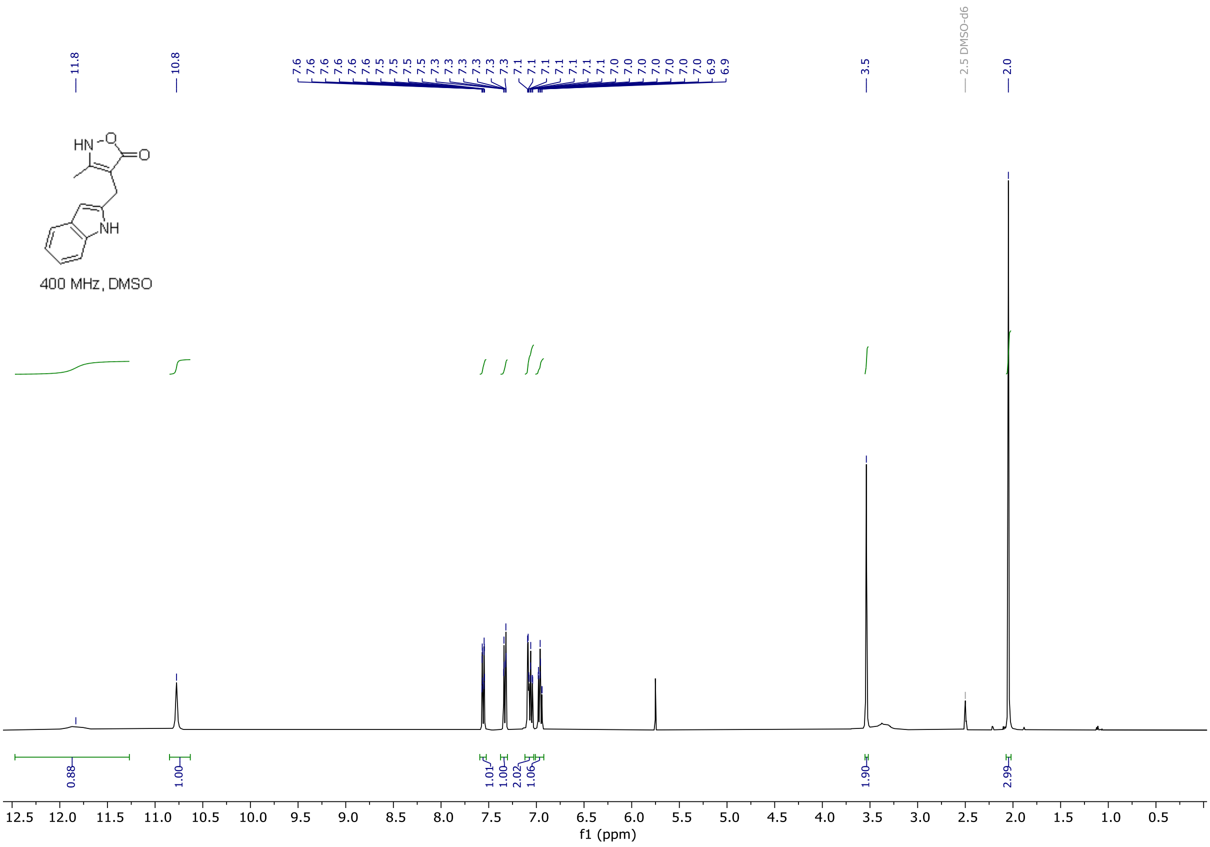

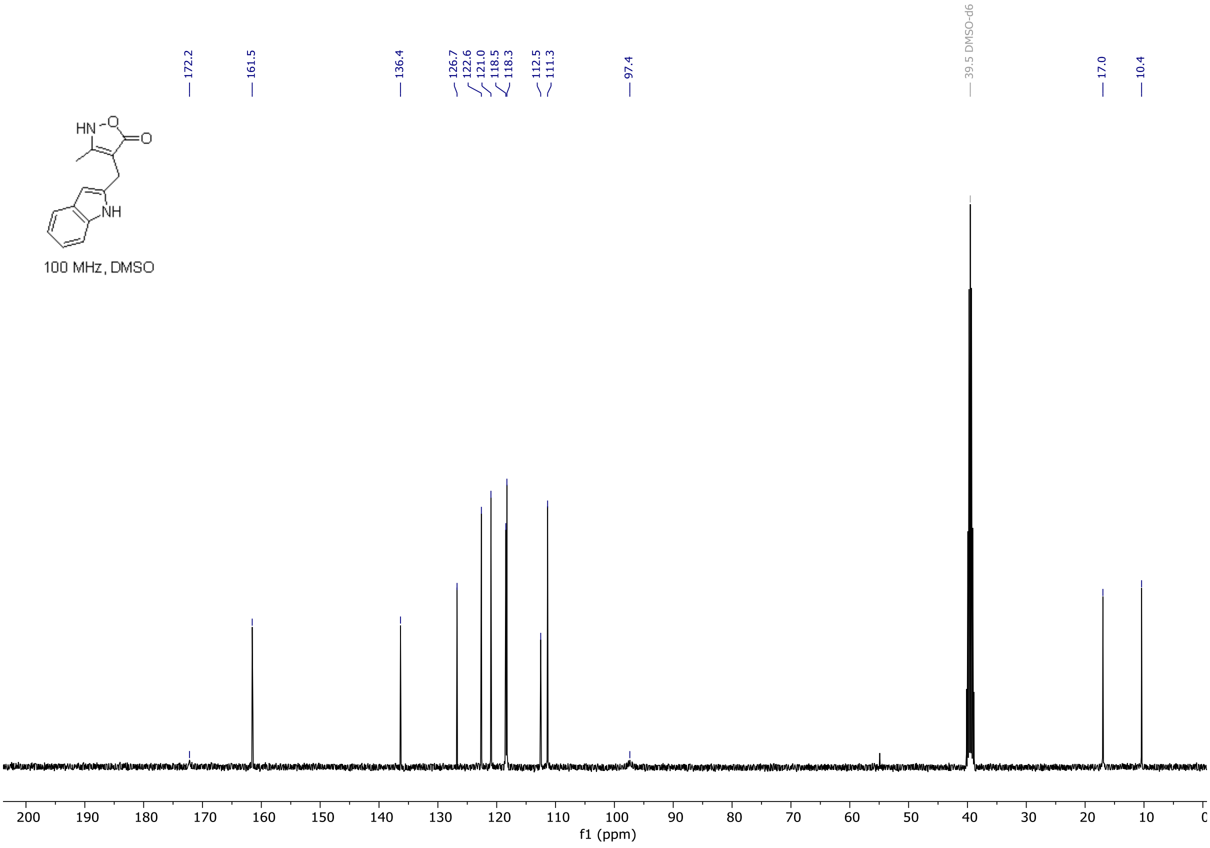
172.2
161.5
136.4
126.7
122.6
121.0
118.5
118.3
112.5
111.3
97.4
39.5 DMSO-d6
17.0
10.4
HN
O
O
NH
100 MHz, DMSO
f1 (ppm)

HSQC Data

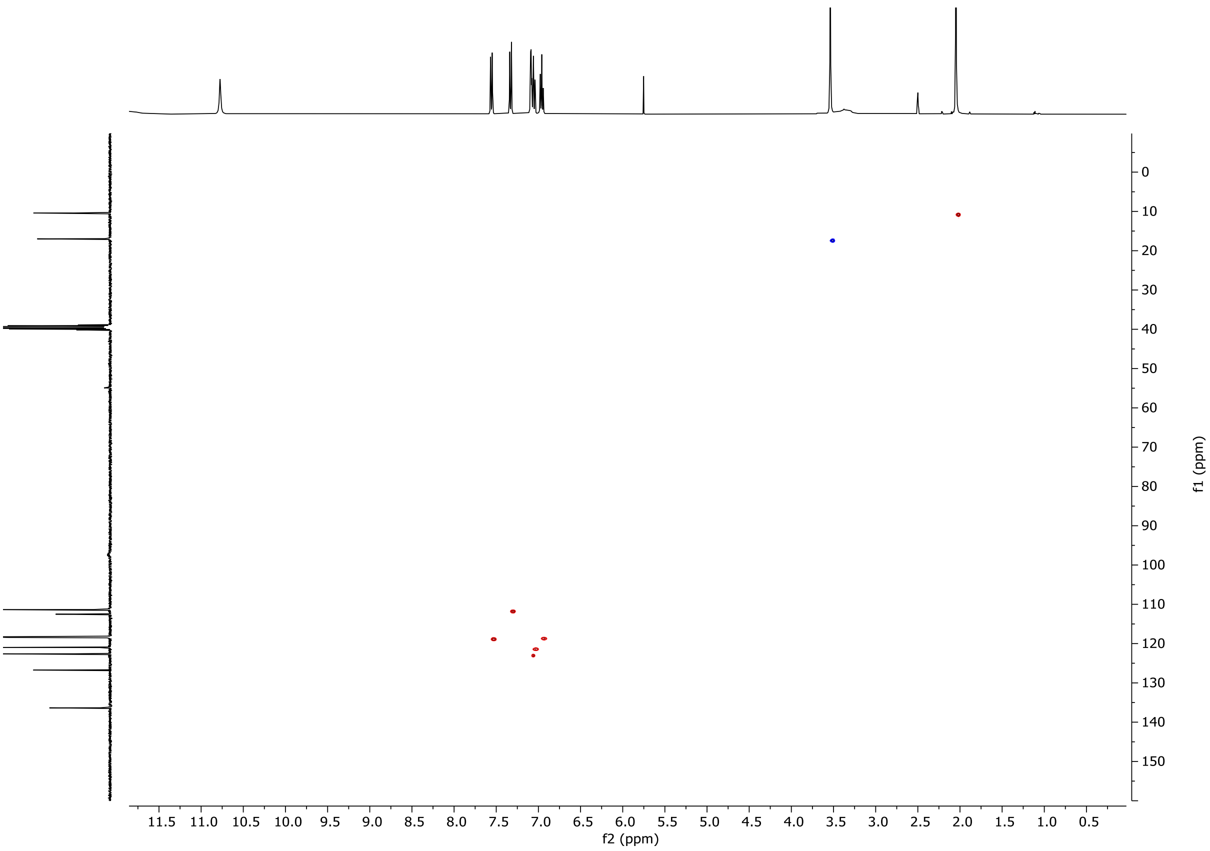
f2 (ppm)
f1 (ppm)

***tert*-Butyl 2-((3-methyl-5-oxo-2,5-dihydroisoxazol-4-yl)methyl)-1H-indole-1-carboxylate (S13)**

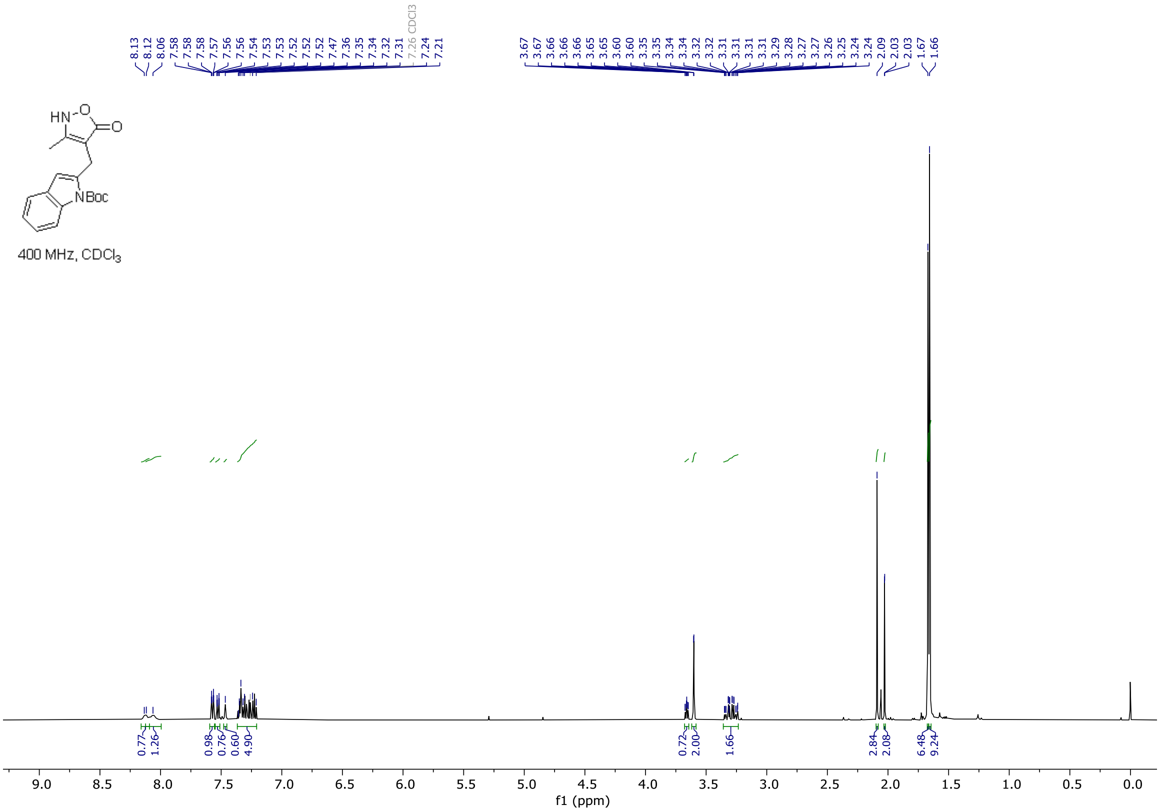

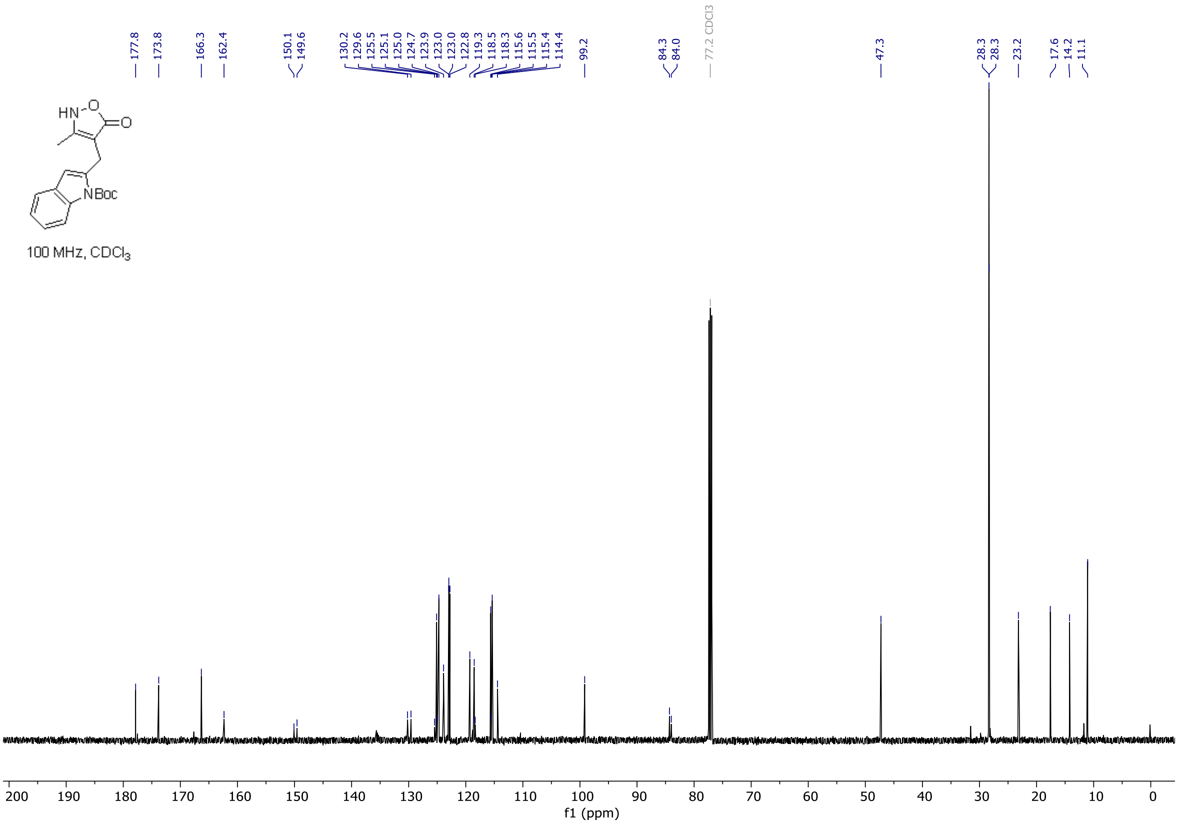

HSQC Data

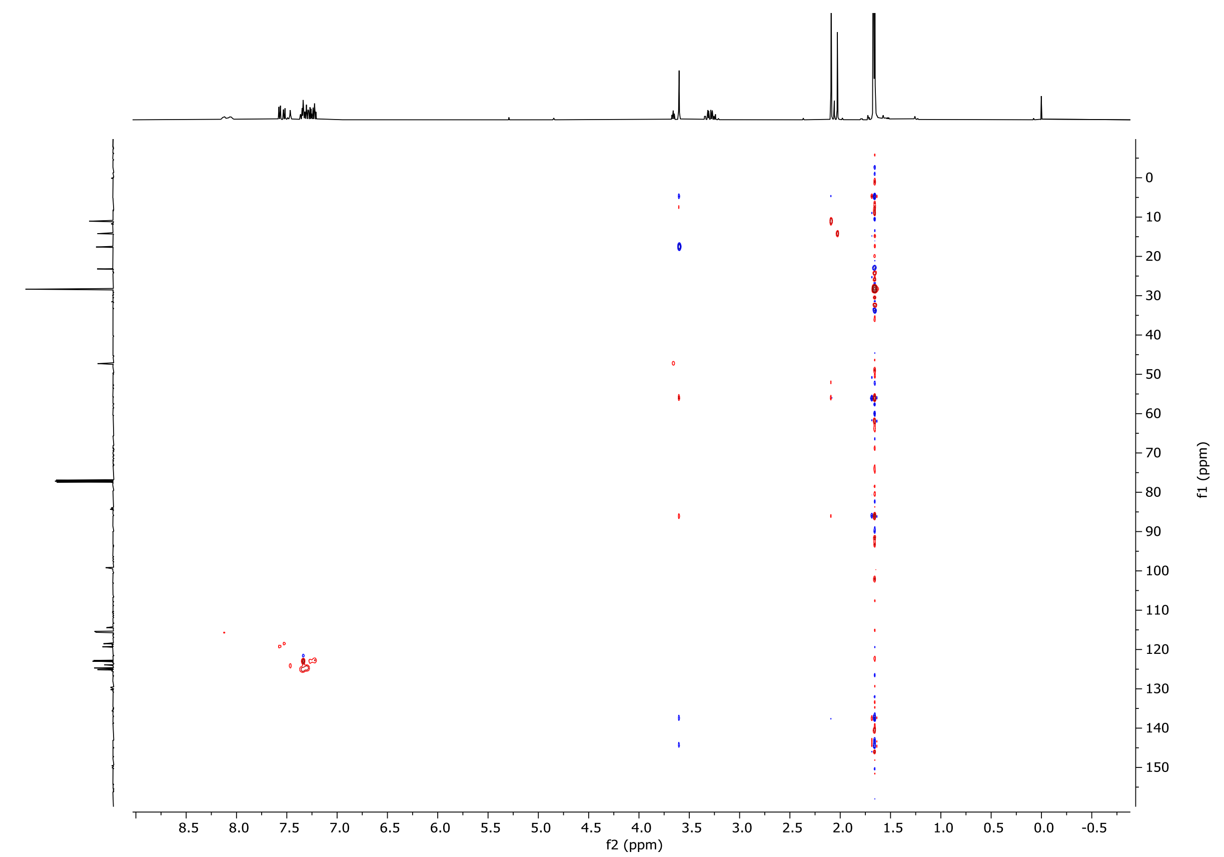

4-(2-Hydroxybenzyl)-3-methylisoxazol-5(2*H*)-one (S14)

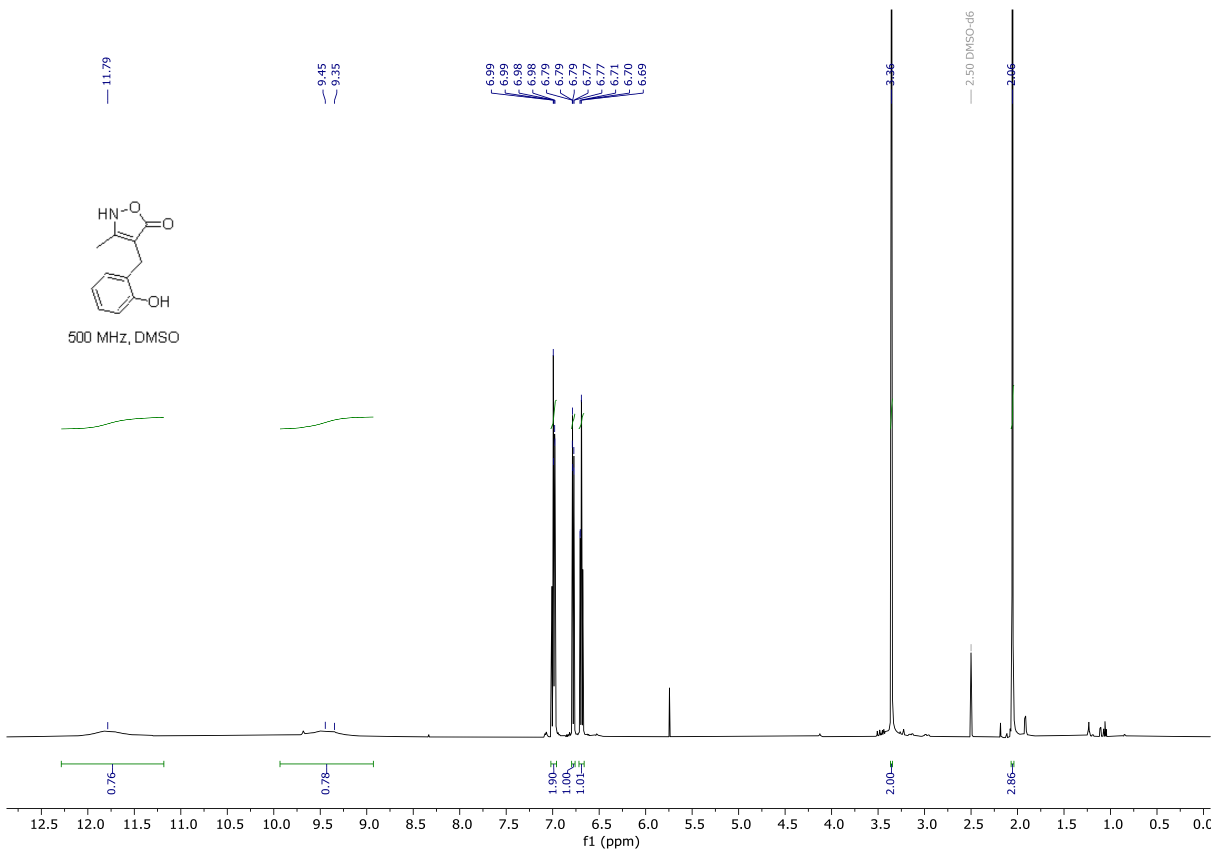

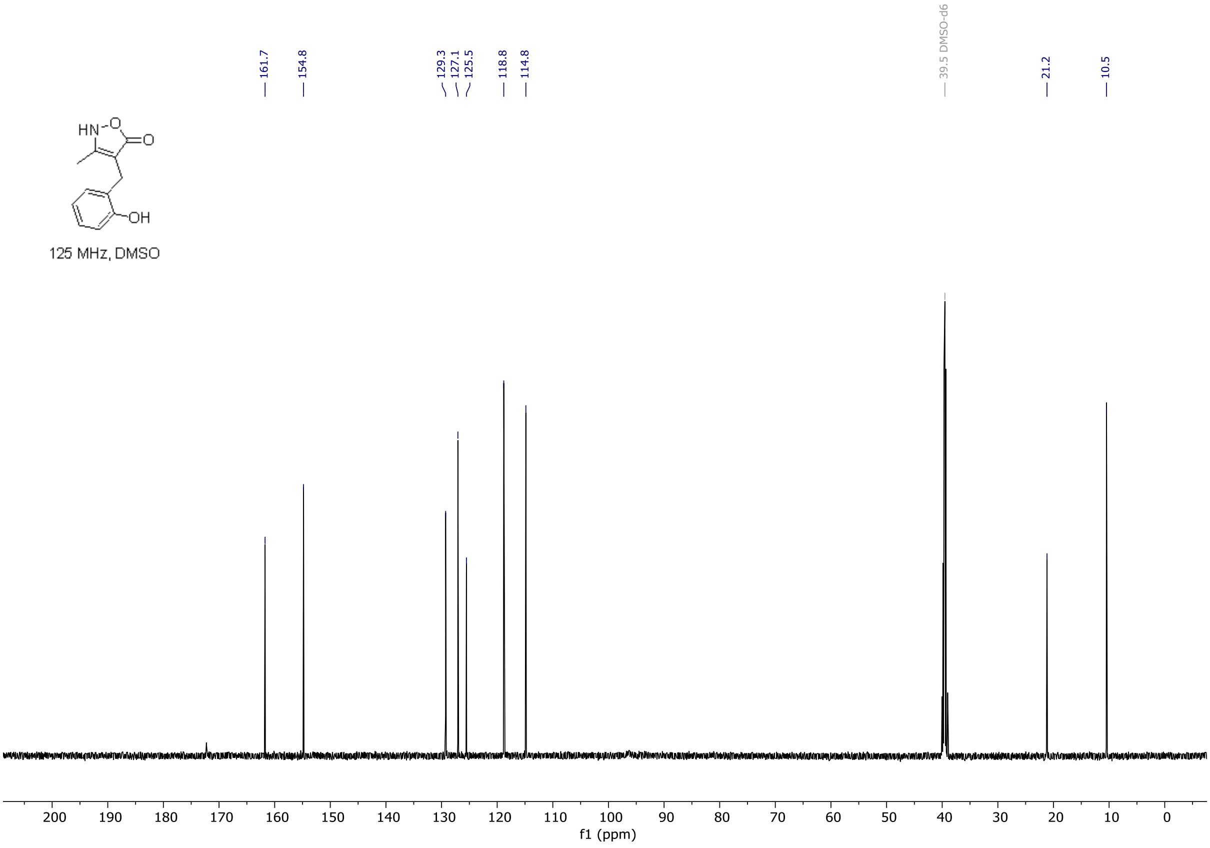
161.7
154.8
129.3
127.1
125.5
118.8
114.8
39.5 DMSO-d6
21.2
10.5
HN
O
O
OH
125 MHz, DMSO
200 190 180 170 160 150 140 130 120 110 100 90 80 70 60 50 40 30 20 10 0
f1 (ppm)

HSQC Data

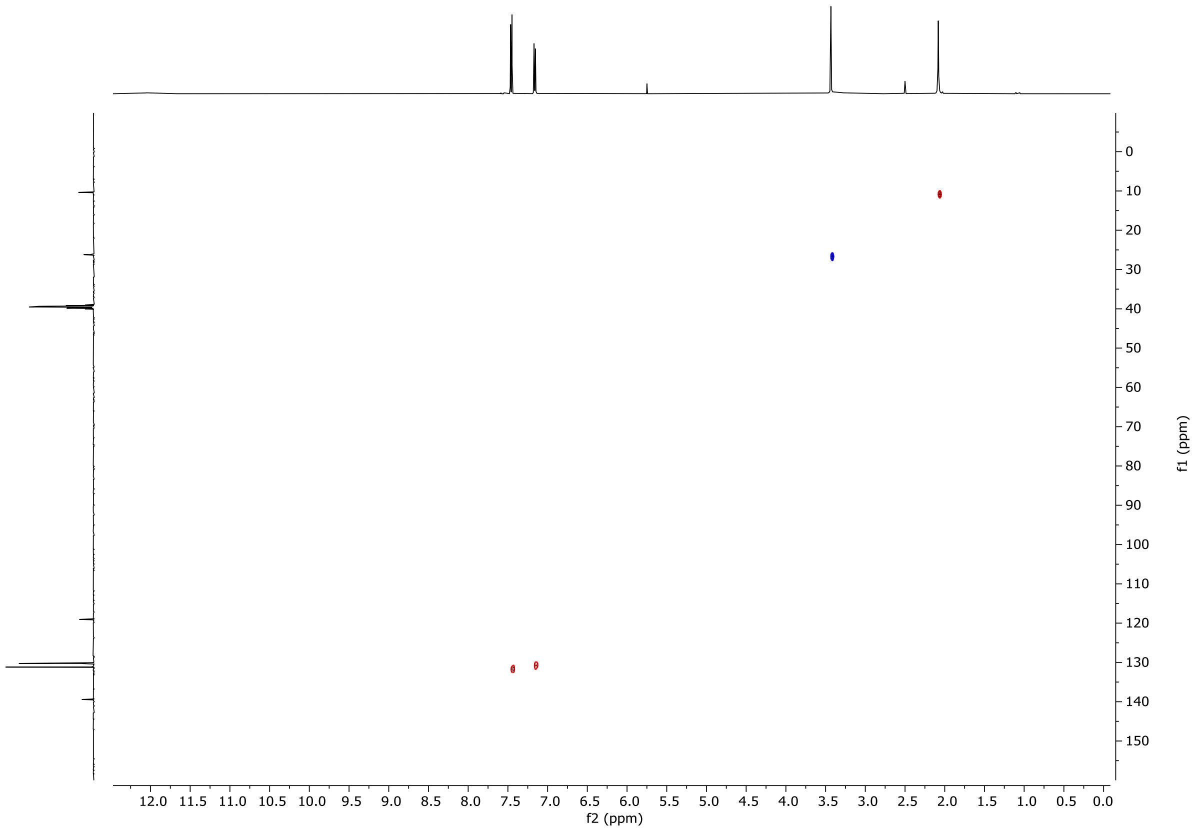
12.0 11.5 11.0 10.5 10.0 9.5 9.0 8.5 8.0 7.5 7.0 6.5 6.0 5.5 5.0 4.5 4.0 3.5 3.0 2.5 2.0 1.5 1.0 0.5 0.0
f2 (ppm)
0 10 20 30 40 50 60 70 80 90 100 110 120 130 140 150
f1 (ppm)

2-Acetyl-4-benzyl-3-methylisoxazol-5(2*H*)-one (1a)

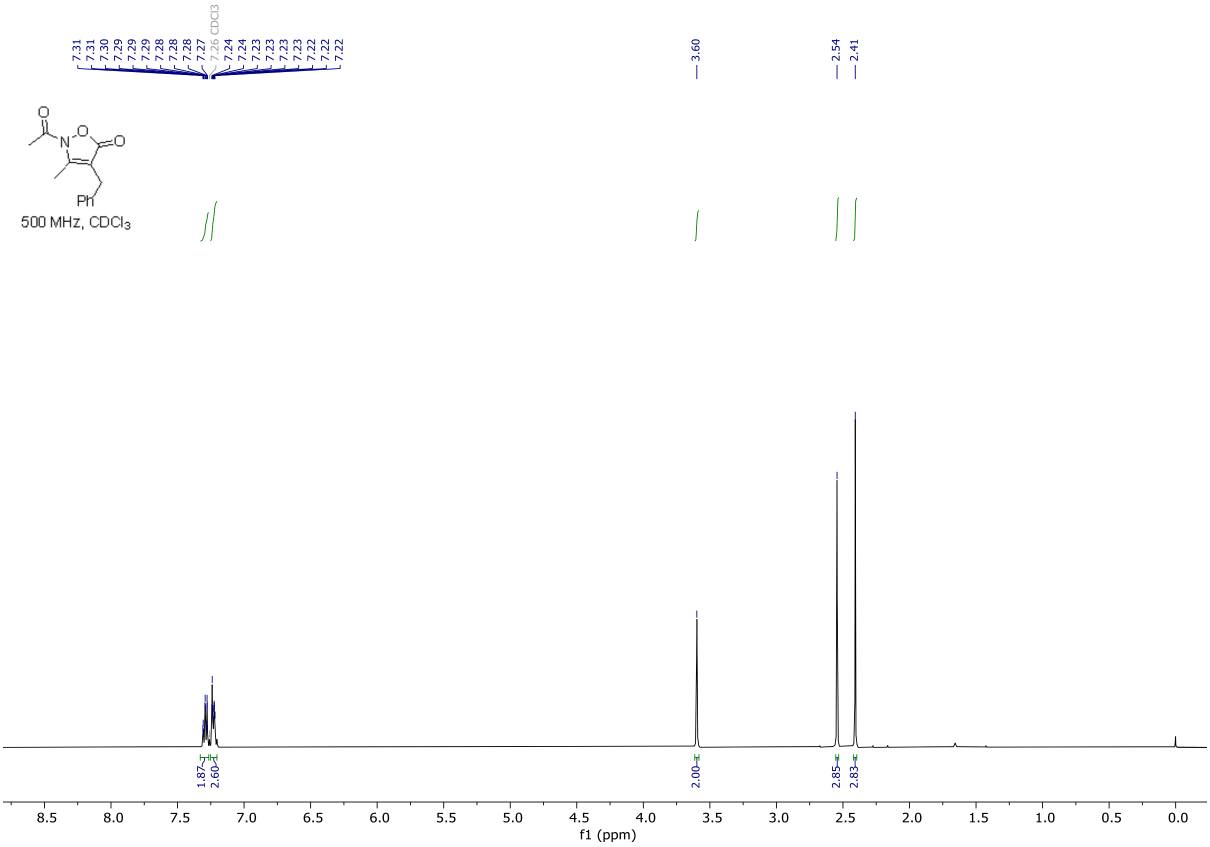

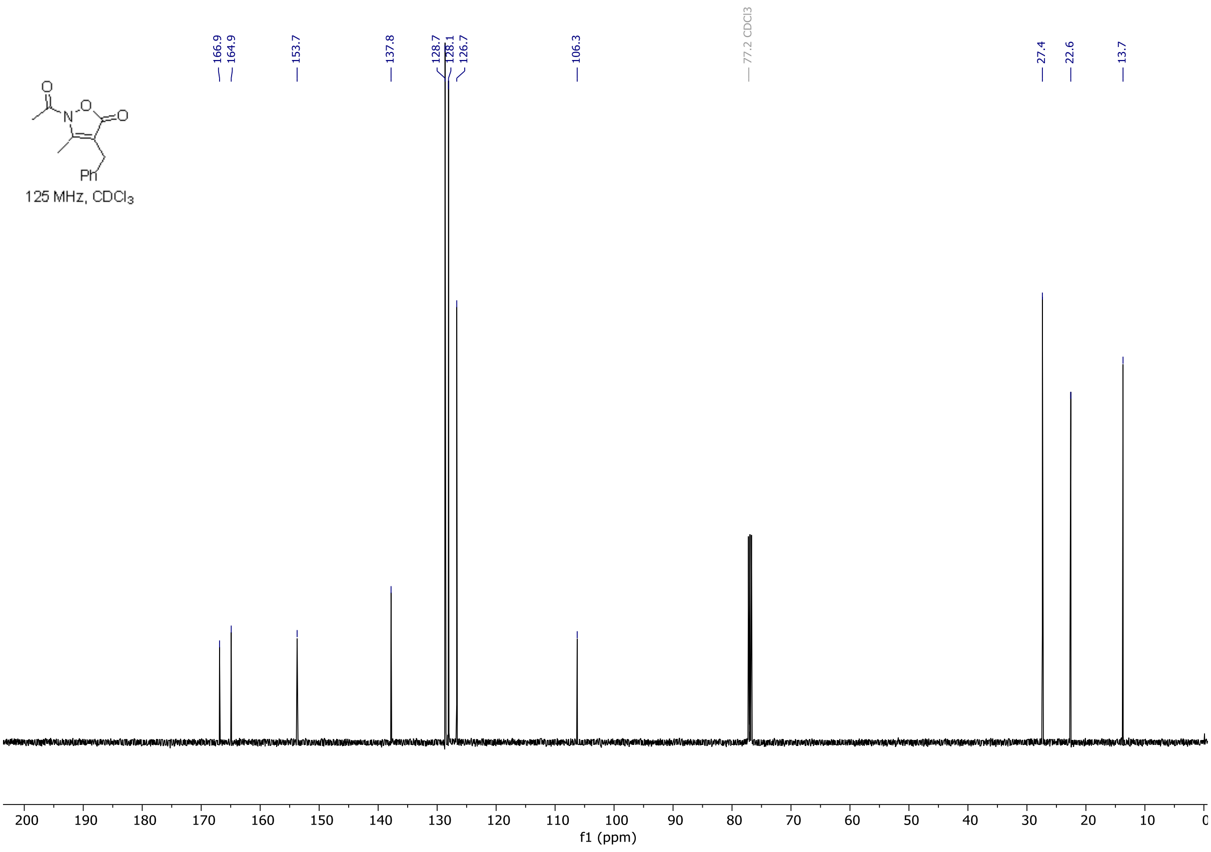

HSQC Data

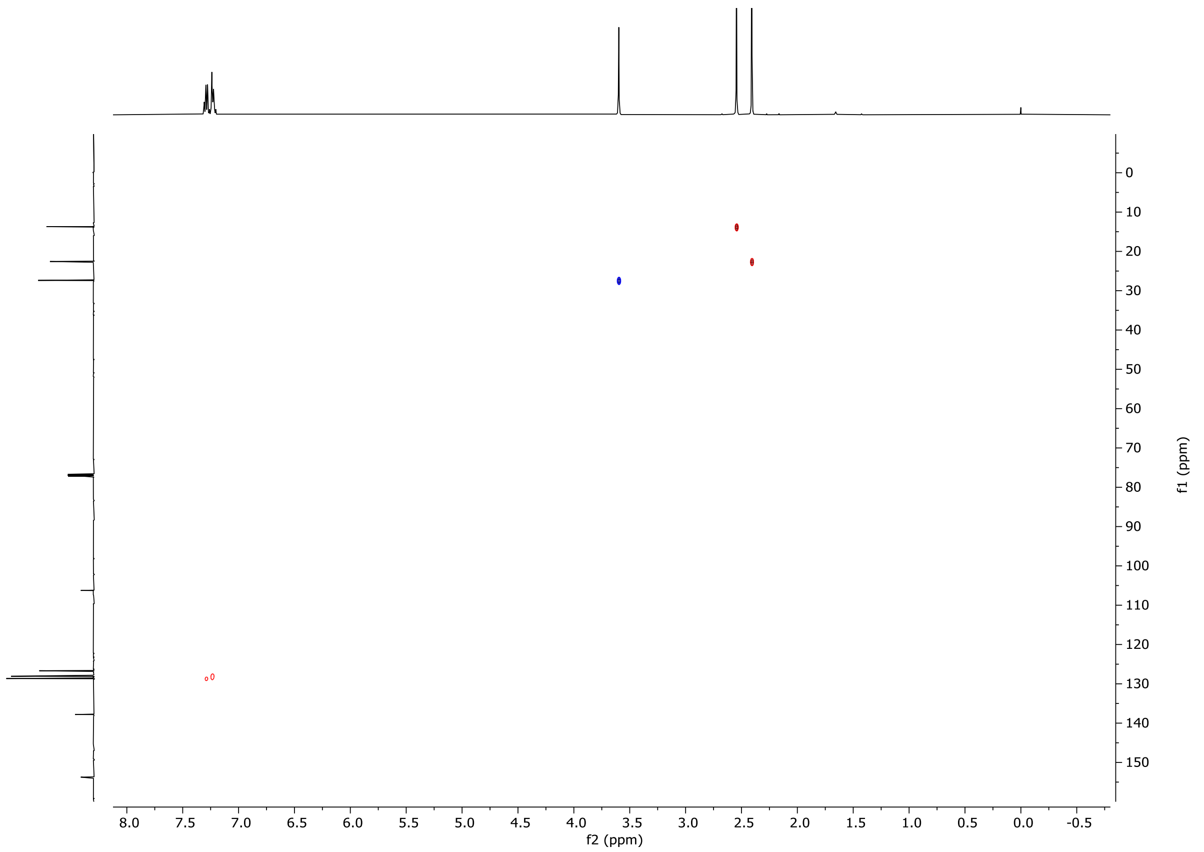

4-Benzyl-2-isobutyryl-3-methylisoxazol-5(2*H*)-one (1b)

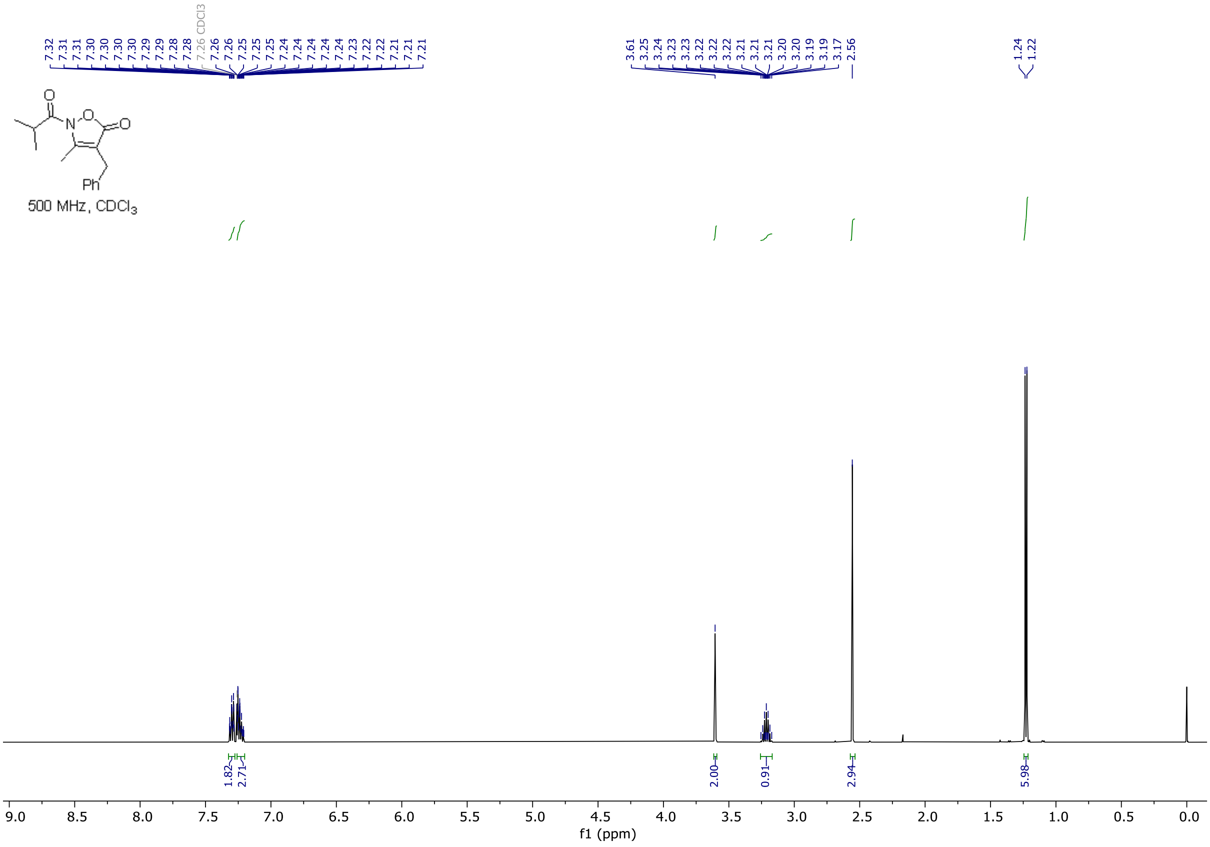

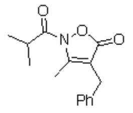

125 MHz, $CDCl_3$

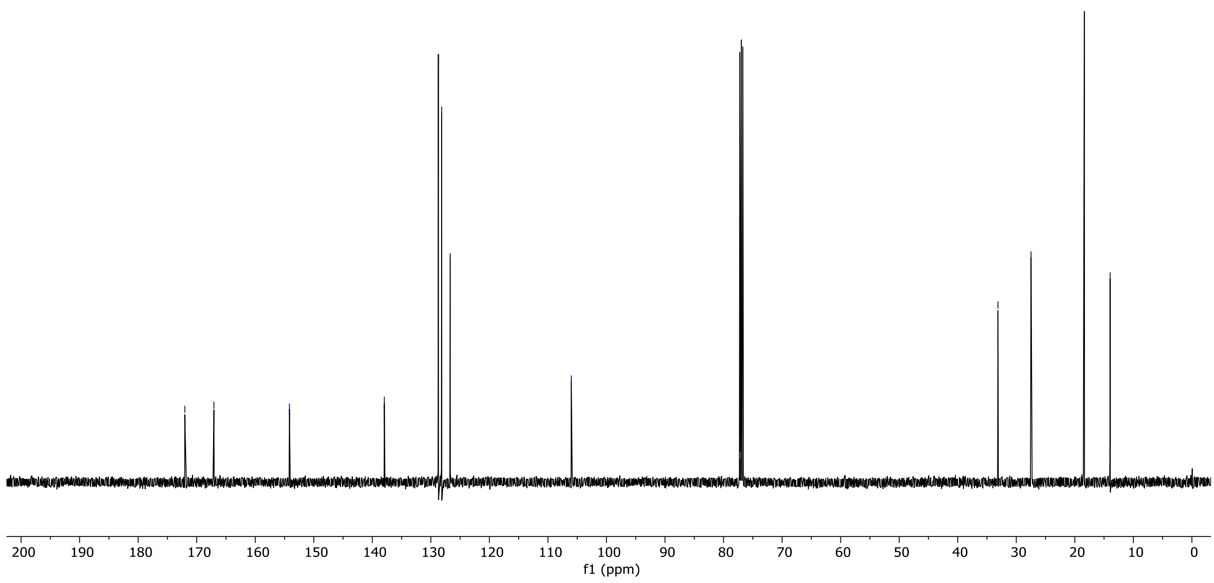

HSQC Data

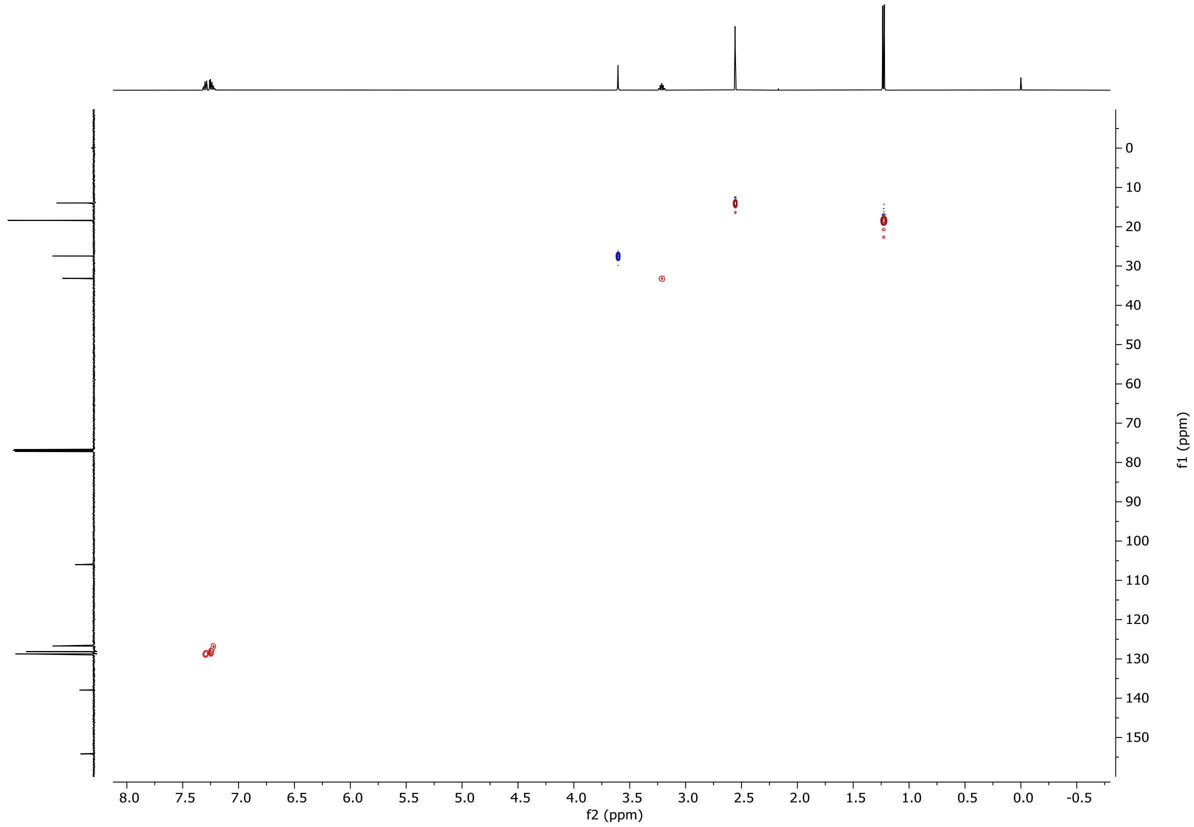

4-Benzyl-3-methyl-2-propionylisoxazol-5(2*H*)-one (1c)

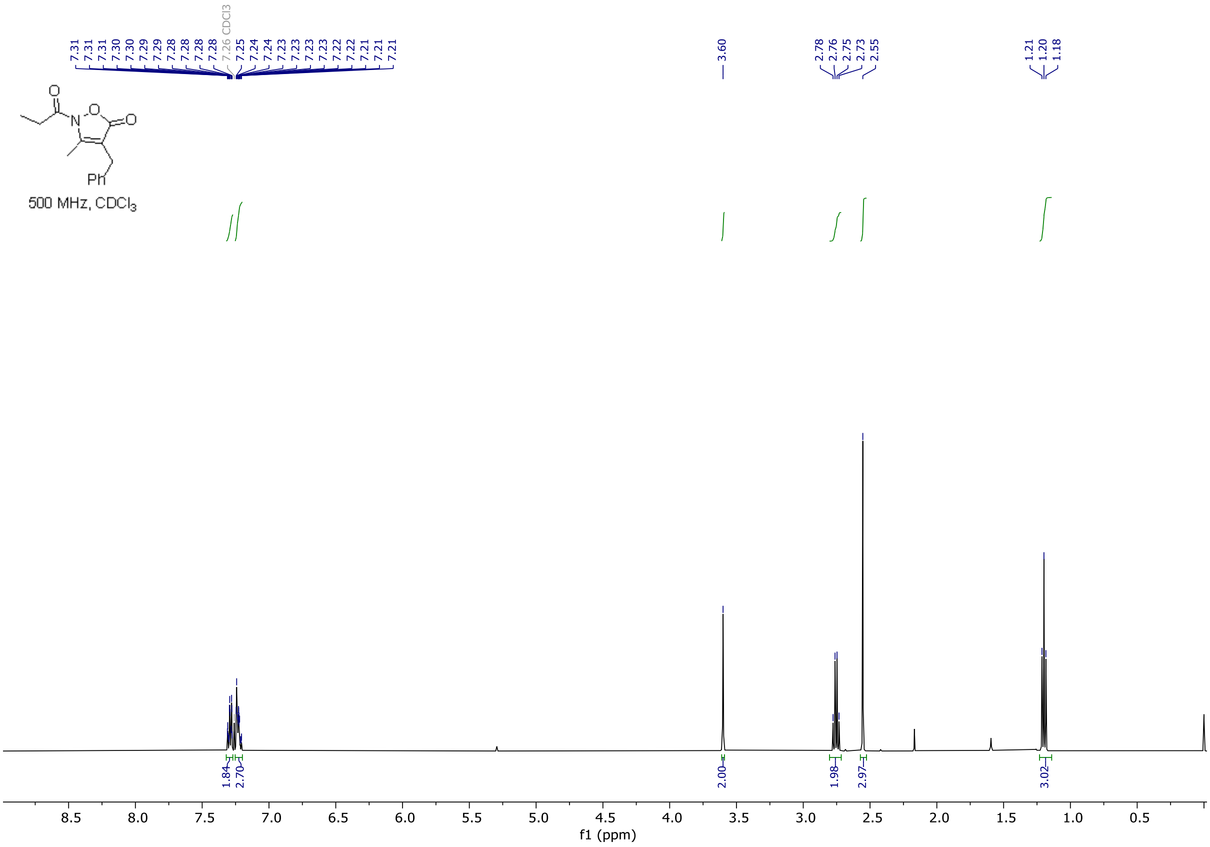

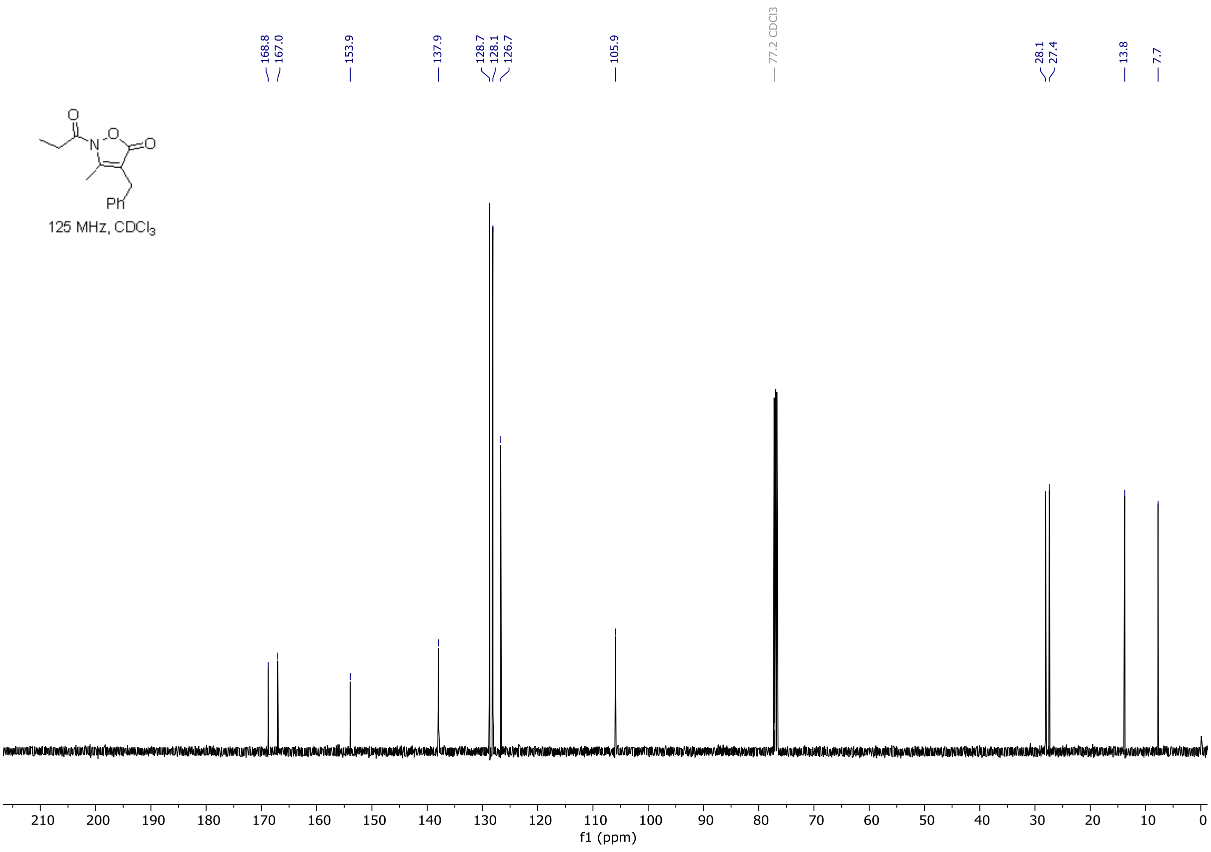

HSQC Data

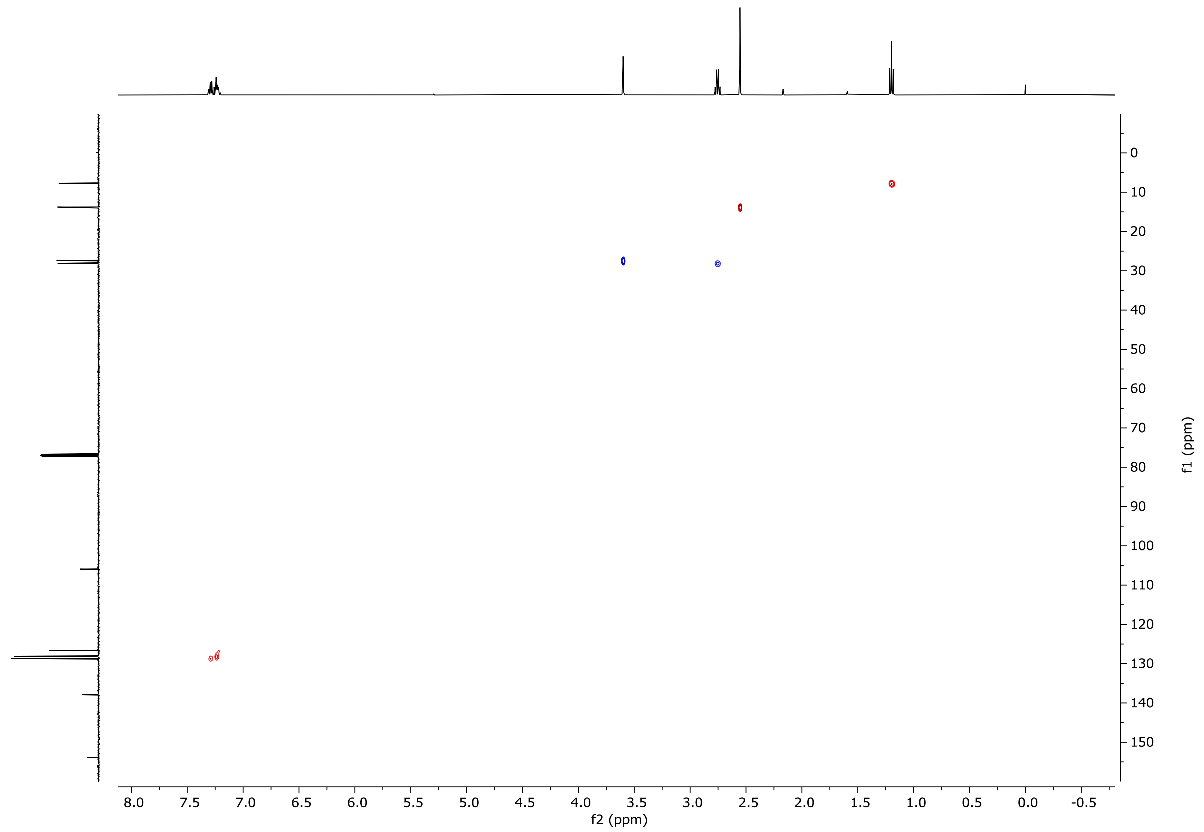

4-Benzyl-3-methyl-2-pentanoylisoxazol-5(2*H*)-one (1d)

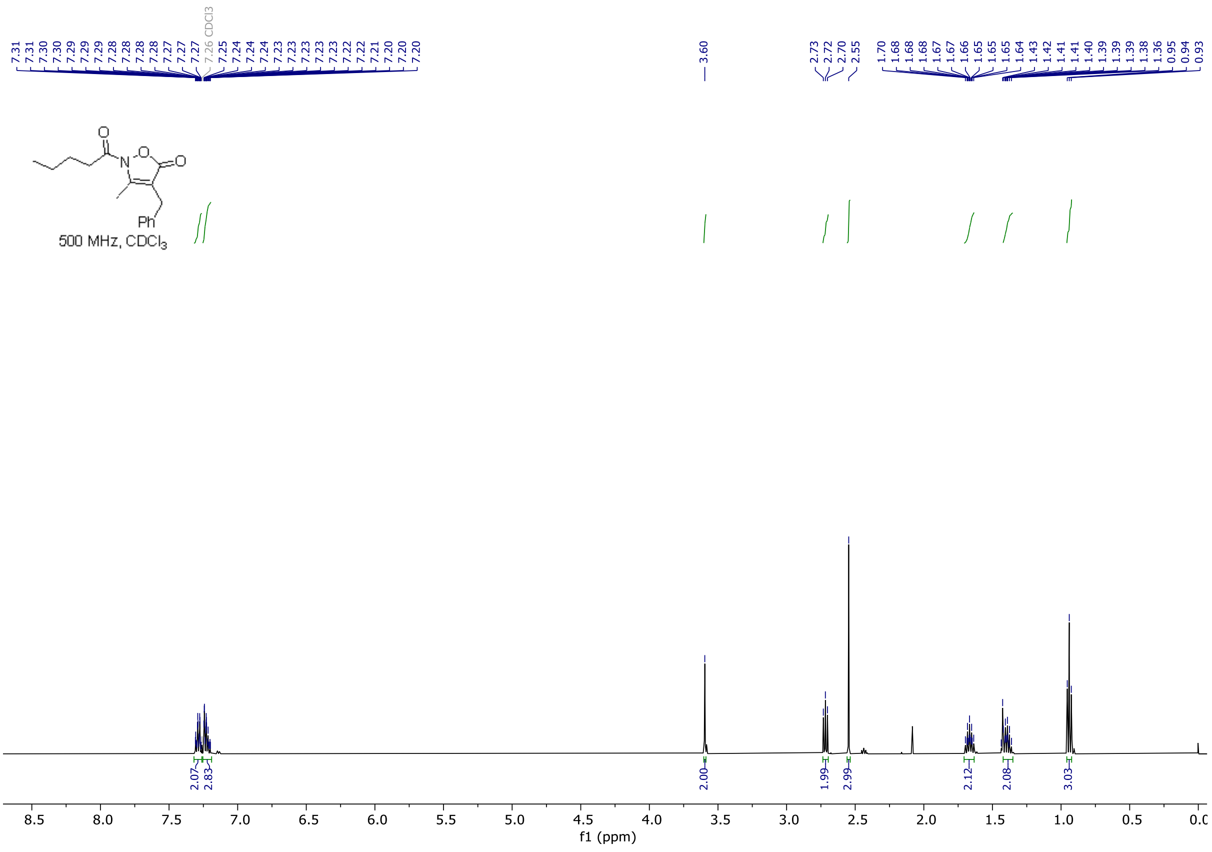

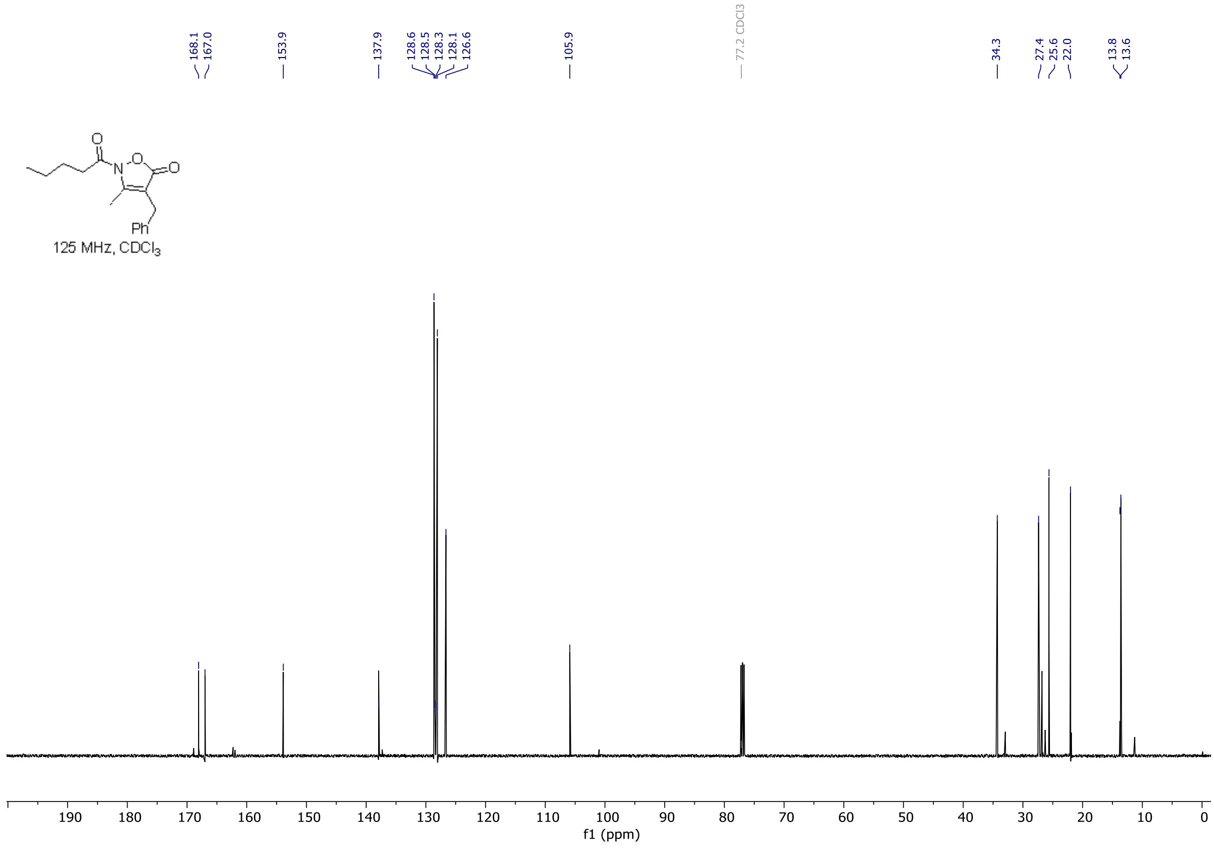
168.1
167.0
153.9
137.9
128.6
128.5
128.3
128.1
126.6
105.9
77.2 CDCl3
34.3
27.4
25.6
22.0
13.8
13.6
Ph
125 MHz, CDCl3
f1 (ppm)

HSQC Data

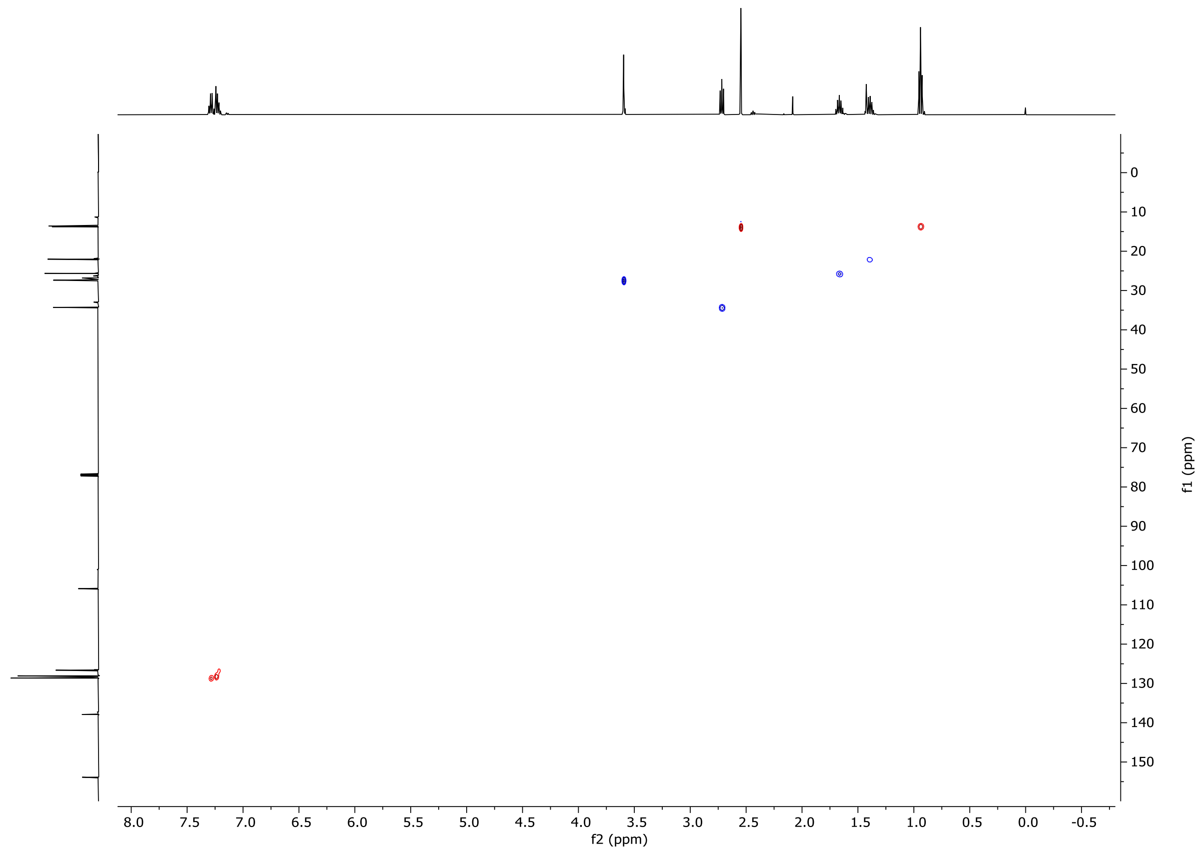
f2 (ppm)
f1 (ppm)

4-Benzyl-2-(cyclopropanecarbonyl)-3-methylisoxazol-5(2*H*)-one (1e)

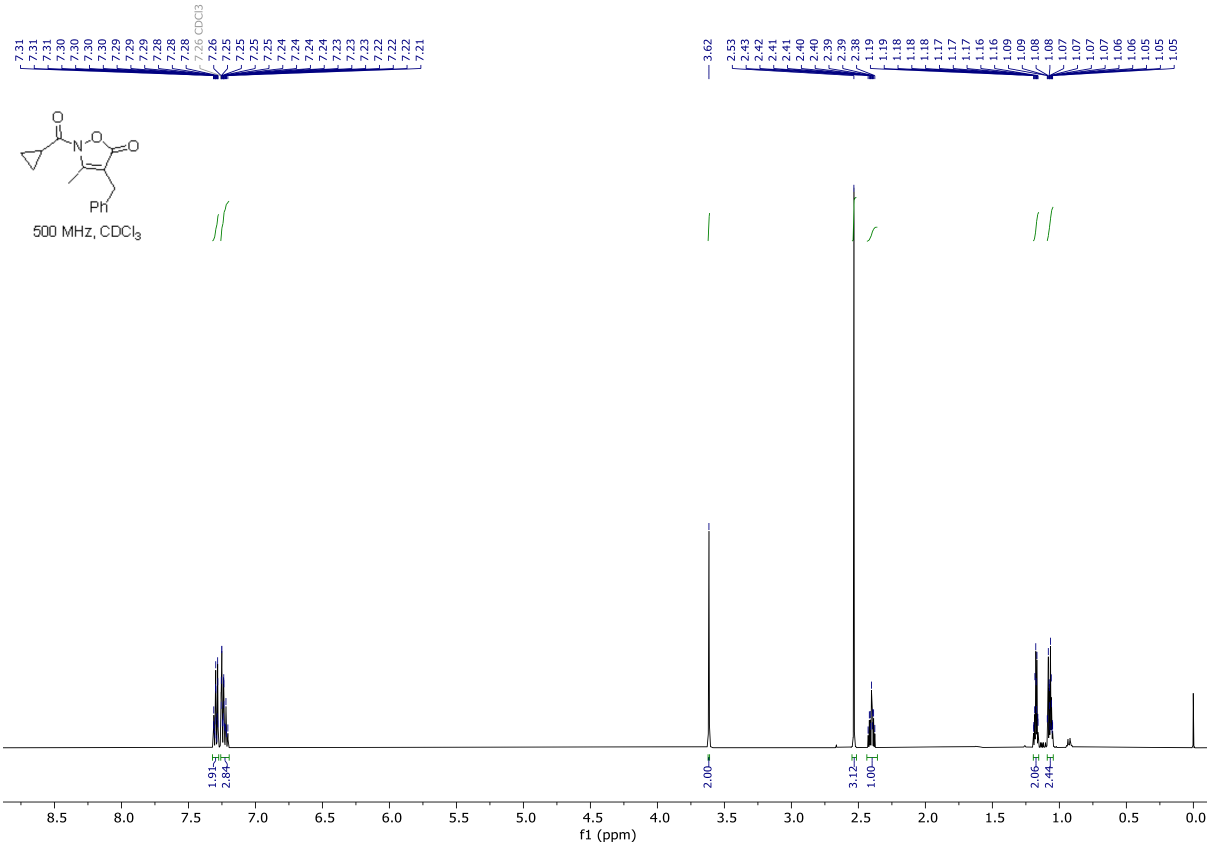

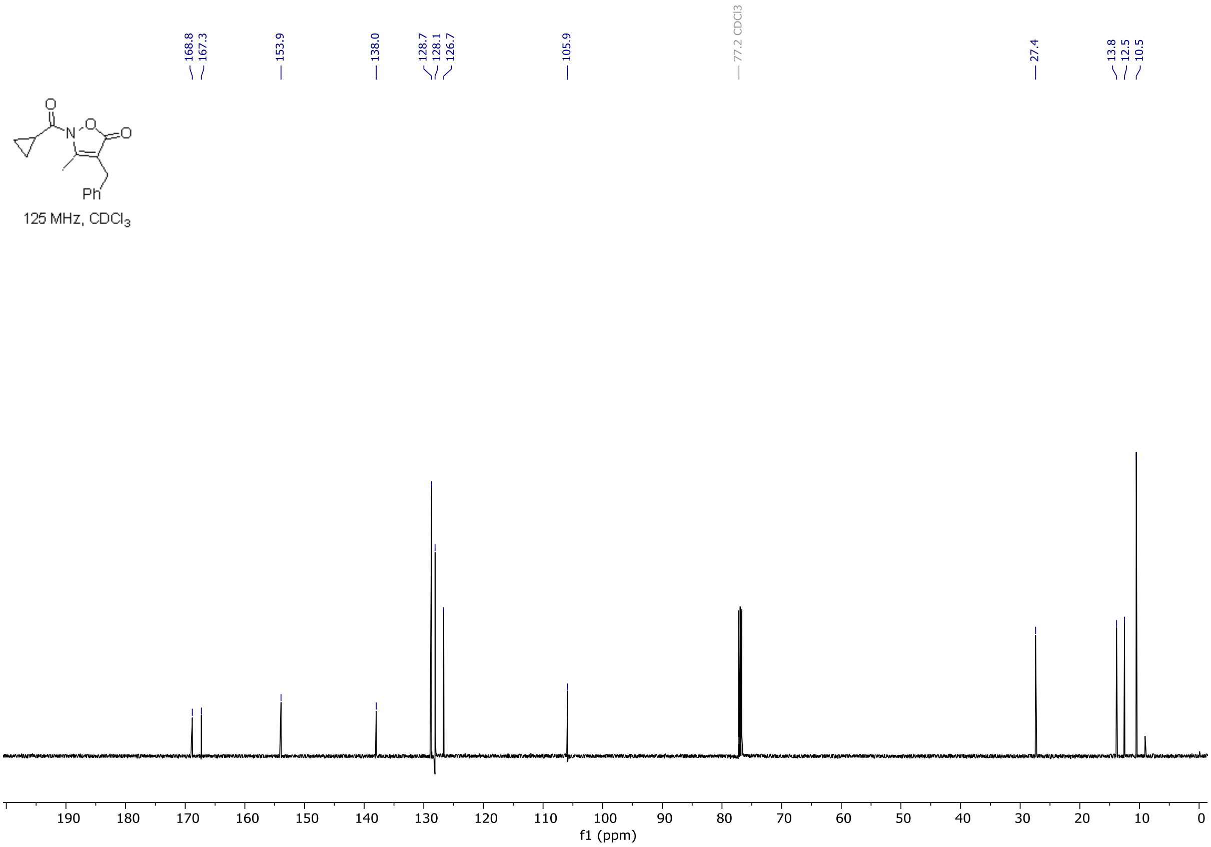

HSQC Data

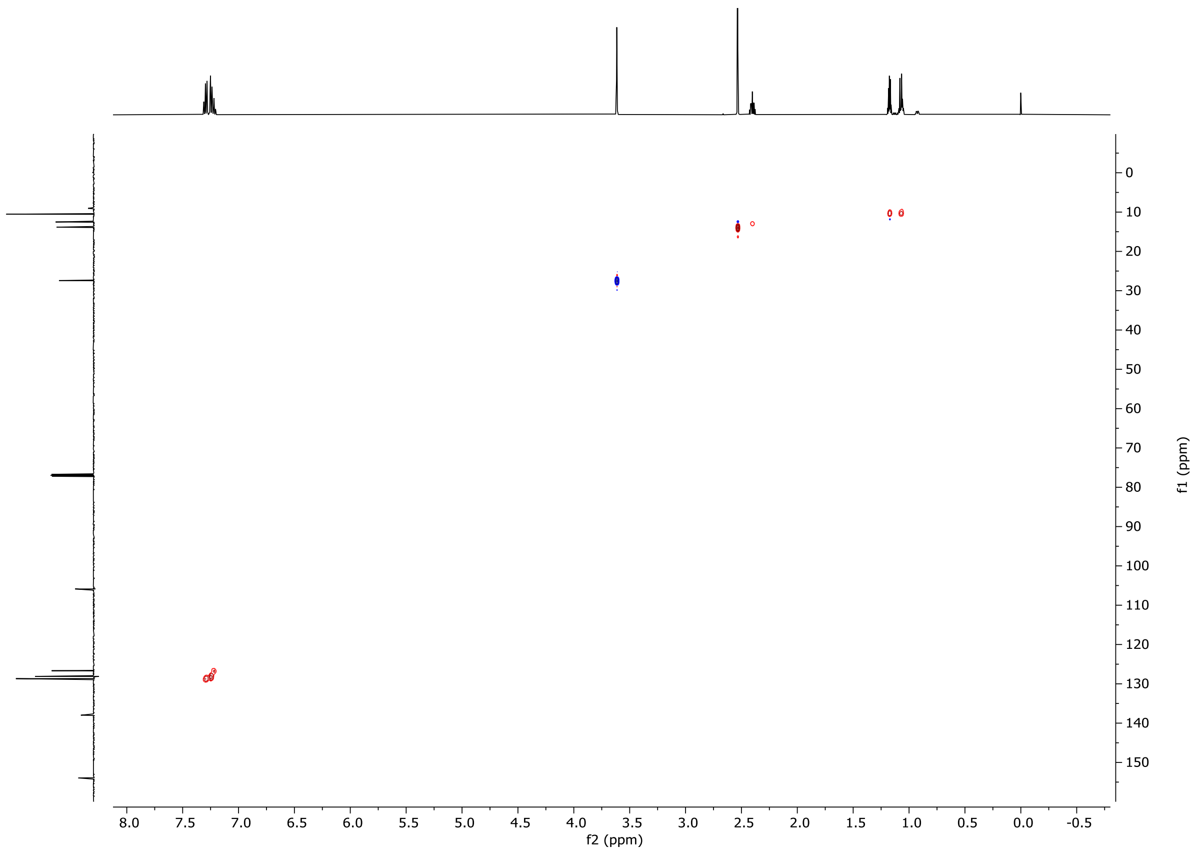

4-Benzyl-2-(cyclobutanecarbonyl)-3-methylisoxazol-5(2*H*)-one (1f)

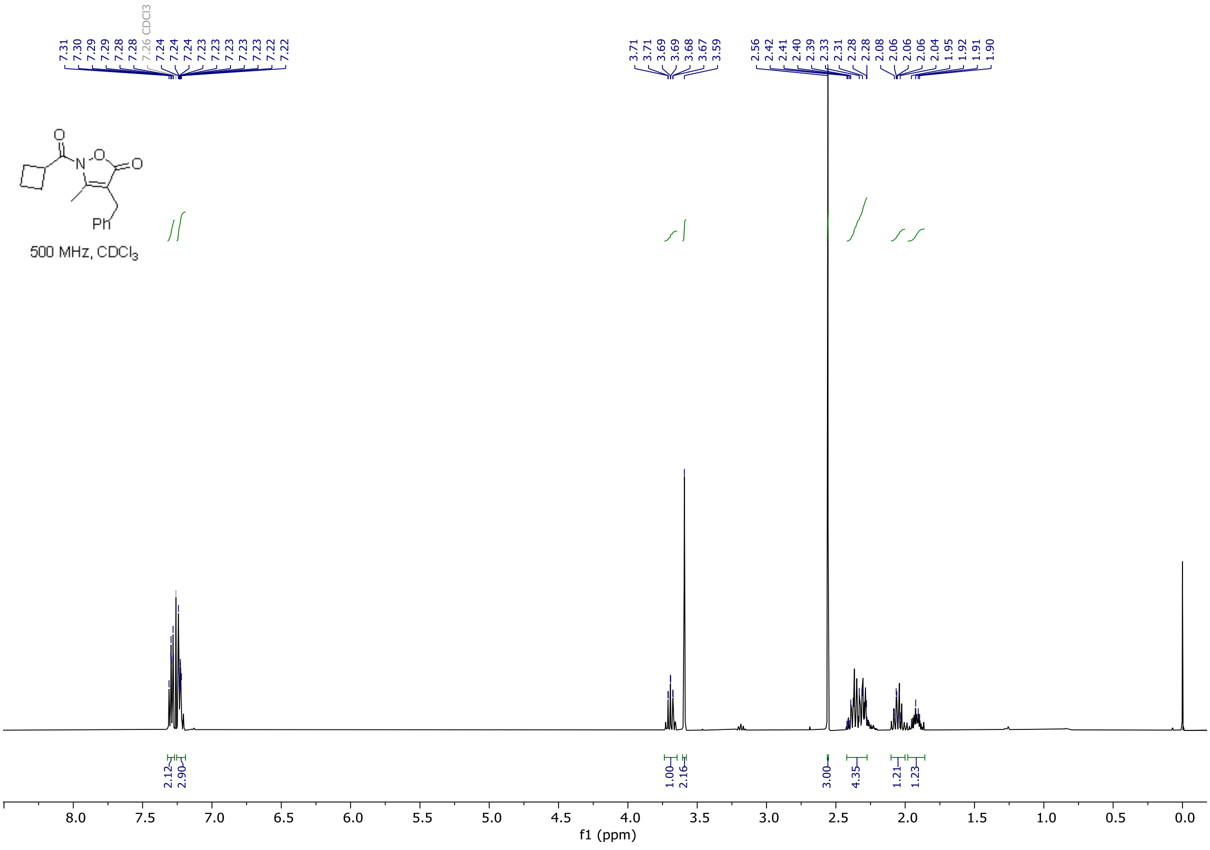

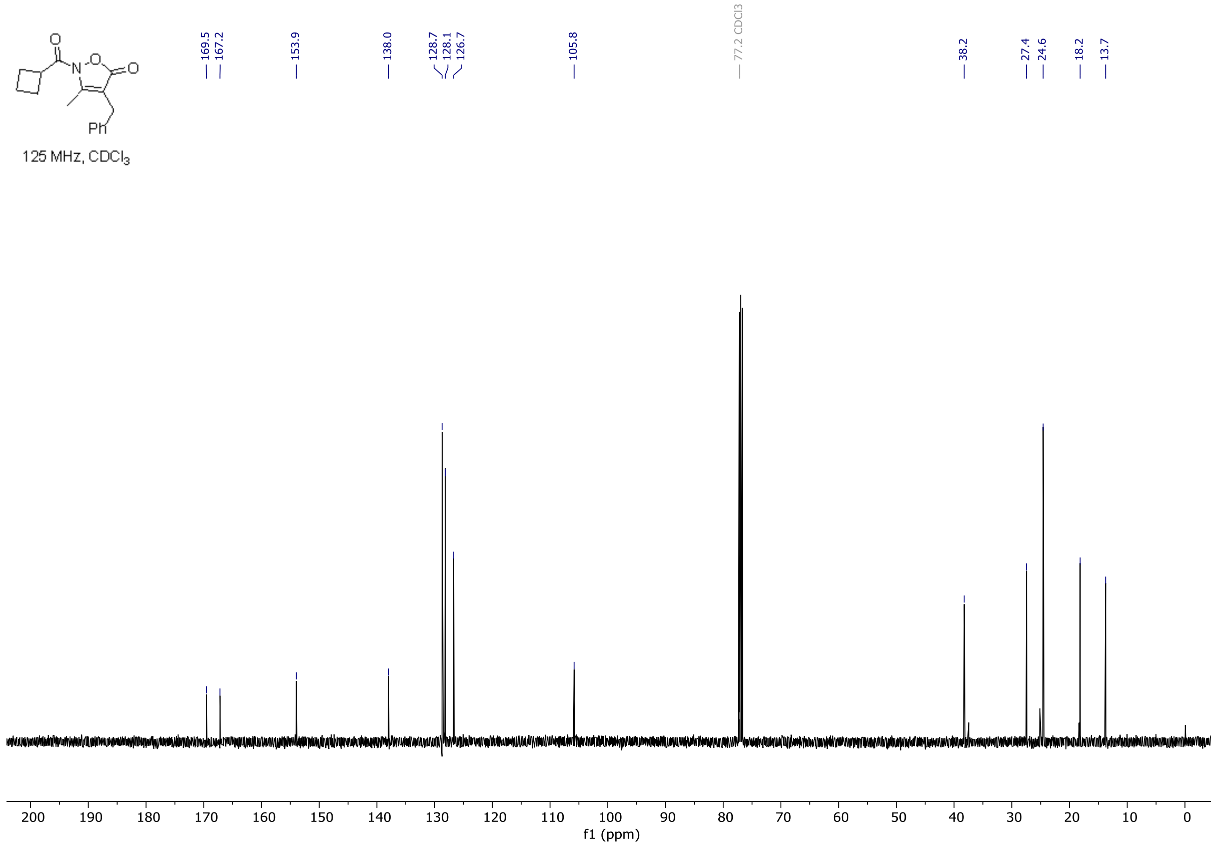
125 MHz, CDCl3
169.5
167.2
153.9
138.0
128.7
128.1
126.7
105.8
77.2 CDCl3
38.2
27.4
24.6
18.2
13.7
f1 (ppm)

HSQC Data

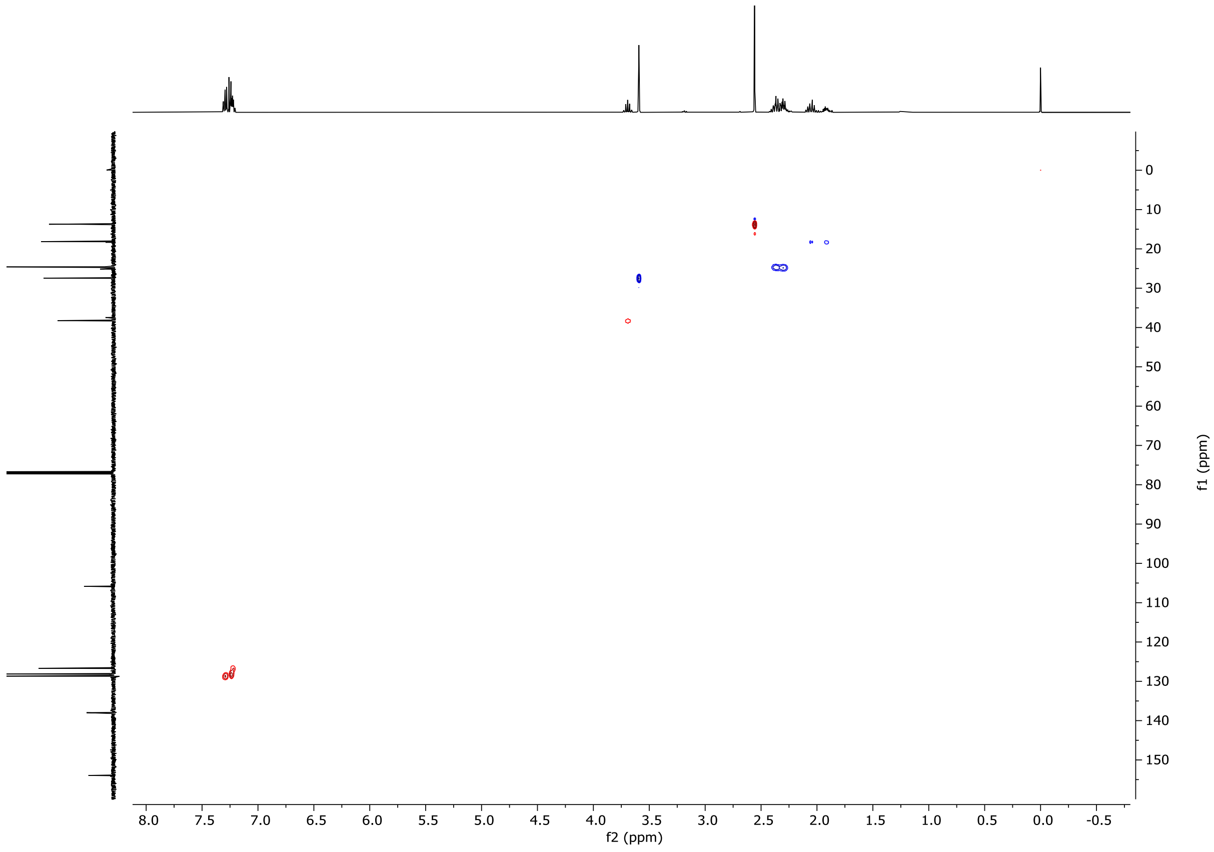
f2 (ppm)
f1 (ppm)

4-Benzyl-2-(cyclohexanecarbonyl)-3-methylisoxazol-5(2*H*)-one (1g)

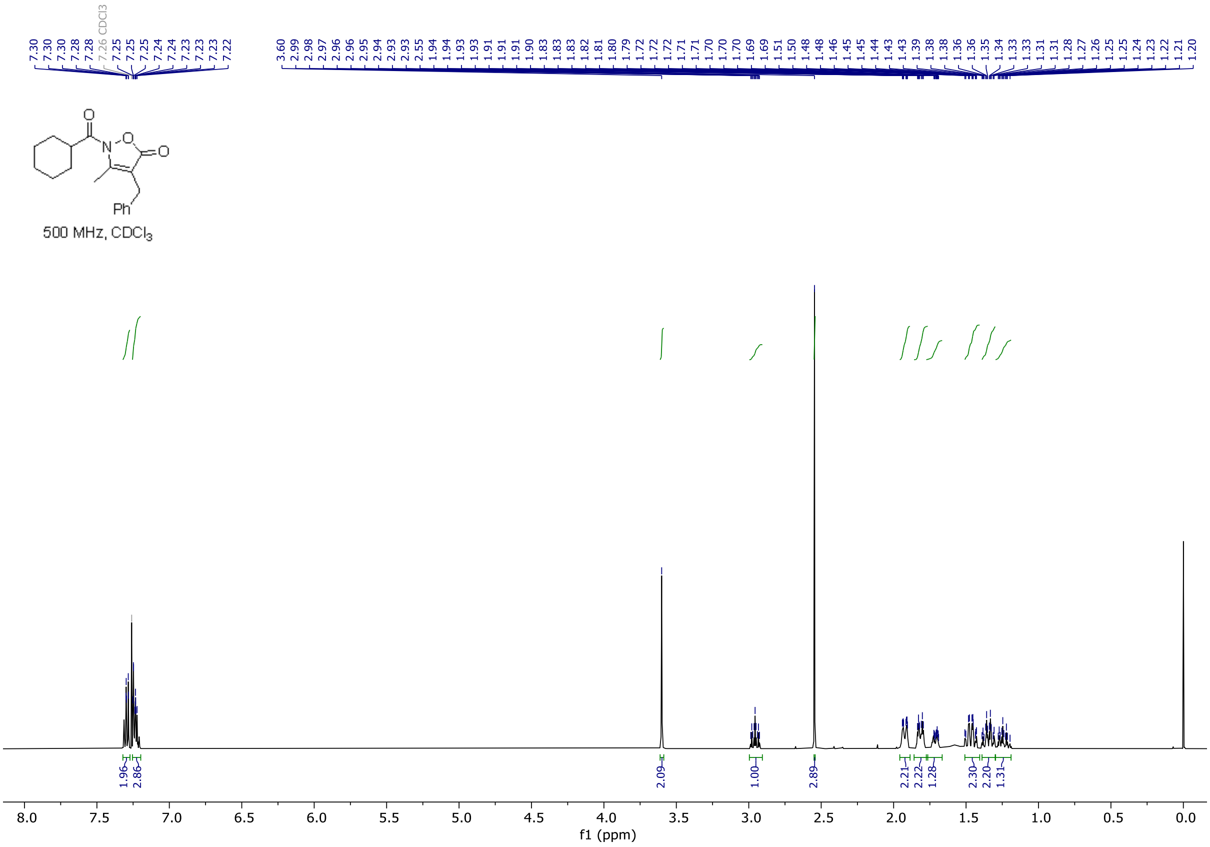

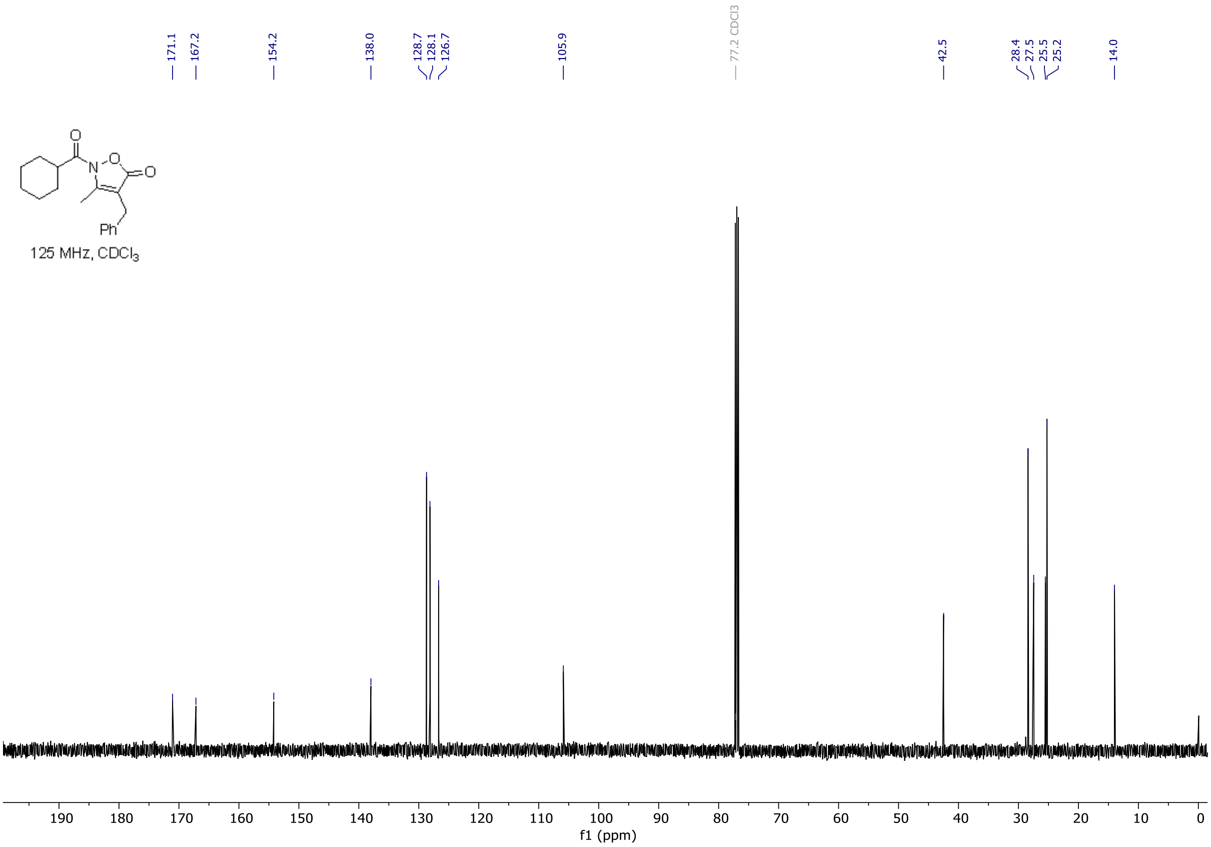

HSQC Data

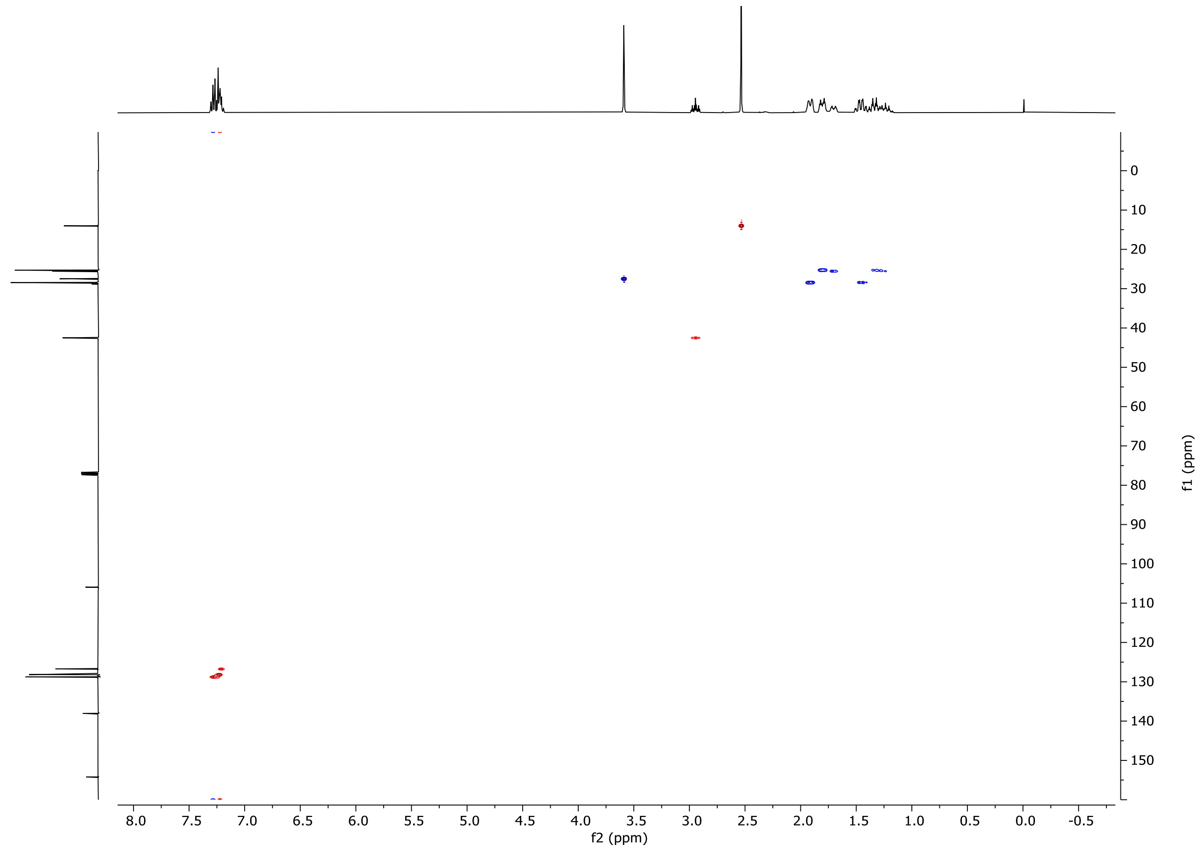

4-Benzyl-3-methyl-2-(tetrahydro-2H-pyran-4-carbonyl)isoxazol-5(2*H*)-one (1h)

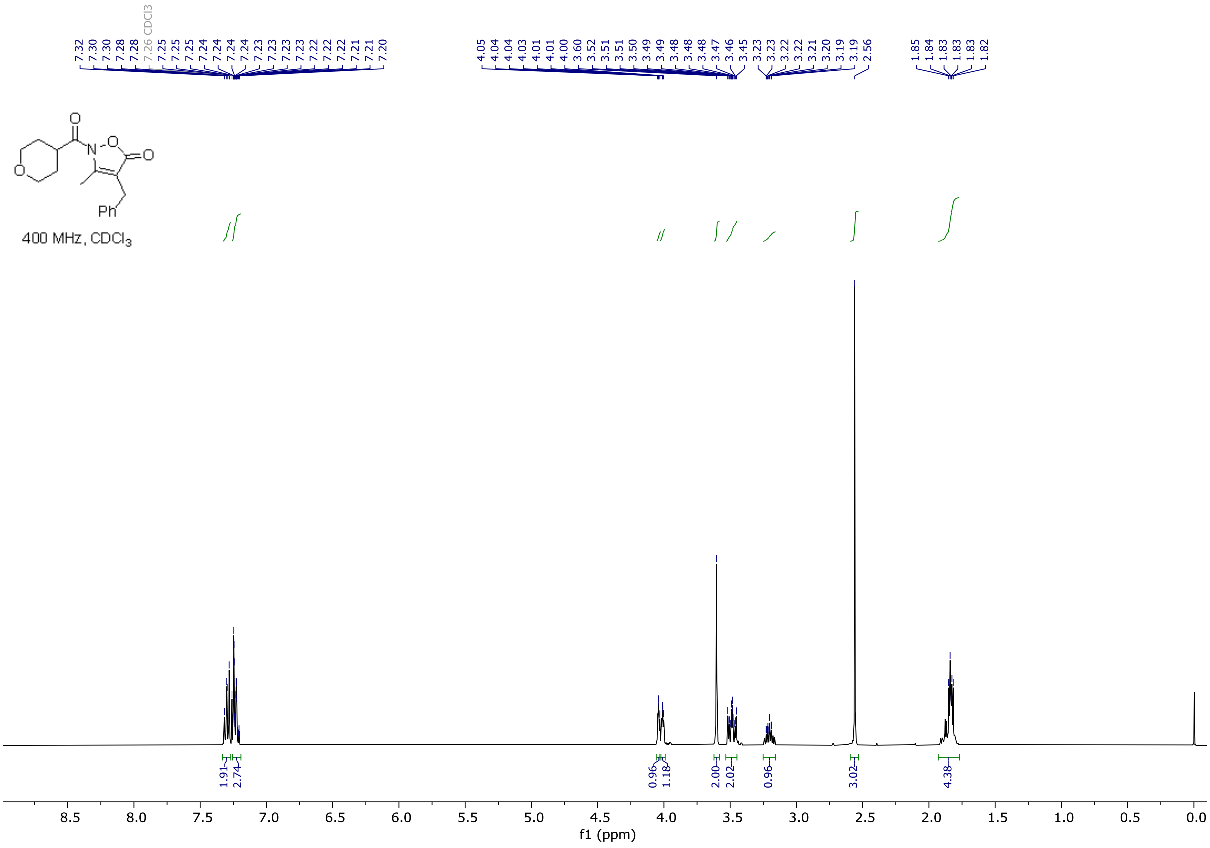

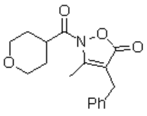

100 MHz, $CDCl_3$

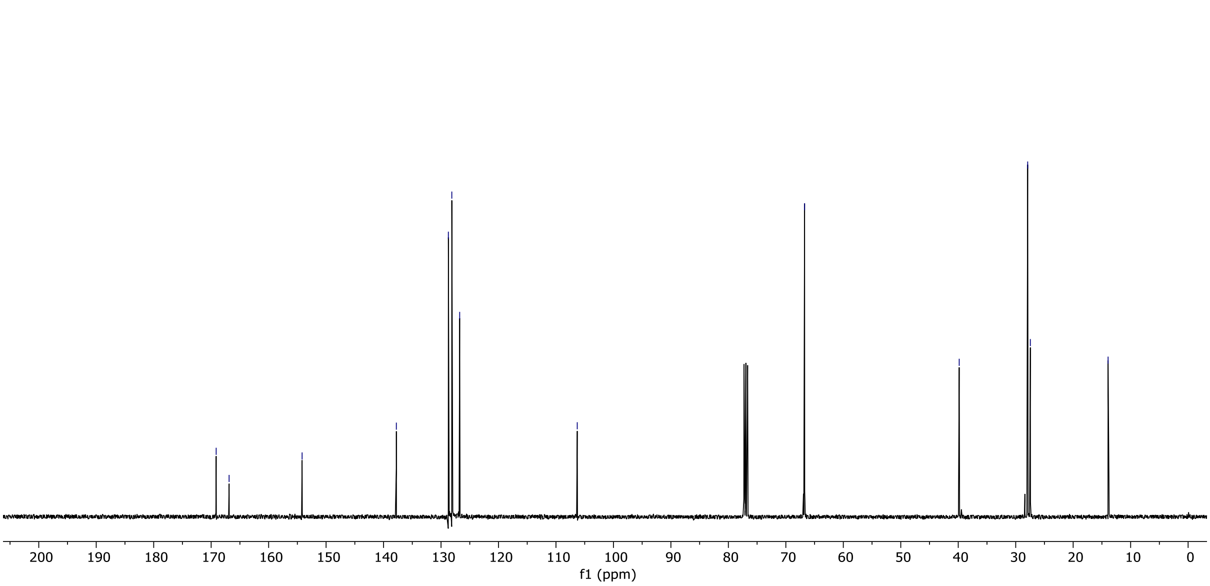

HSQC Data

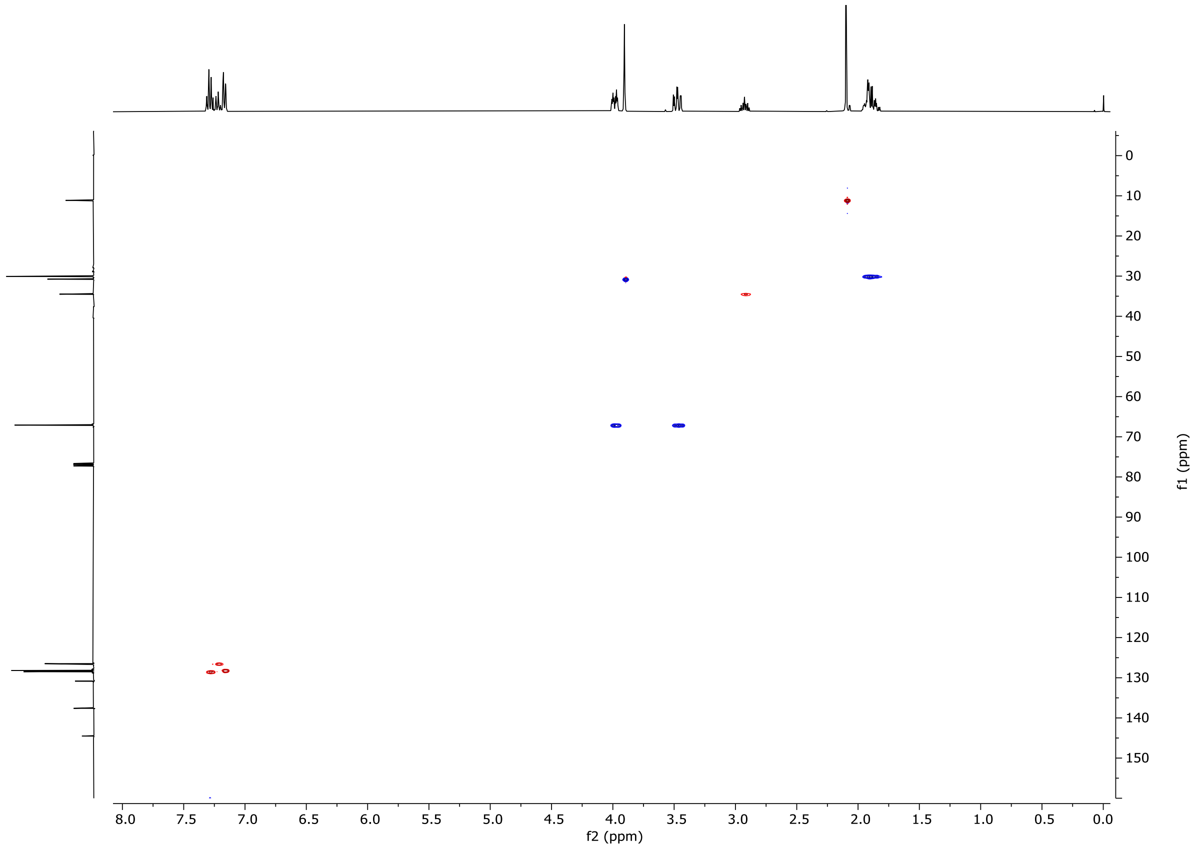

Methyl 4-(4-benzyl-3-methyl-5-oxo-2,5-dihydroisoxazole-2-carbonyl)bicyclo[2.2.2]octane-1-carboxylate (1i)

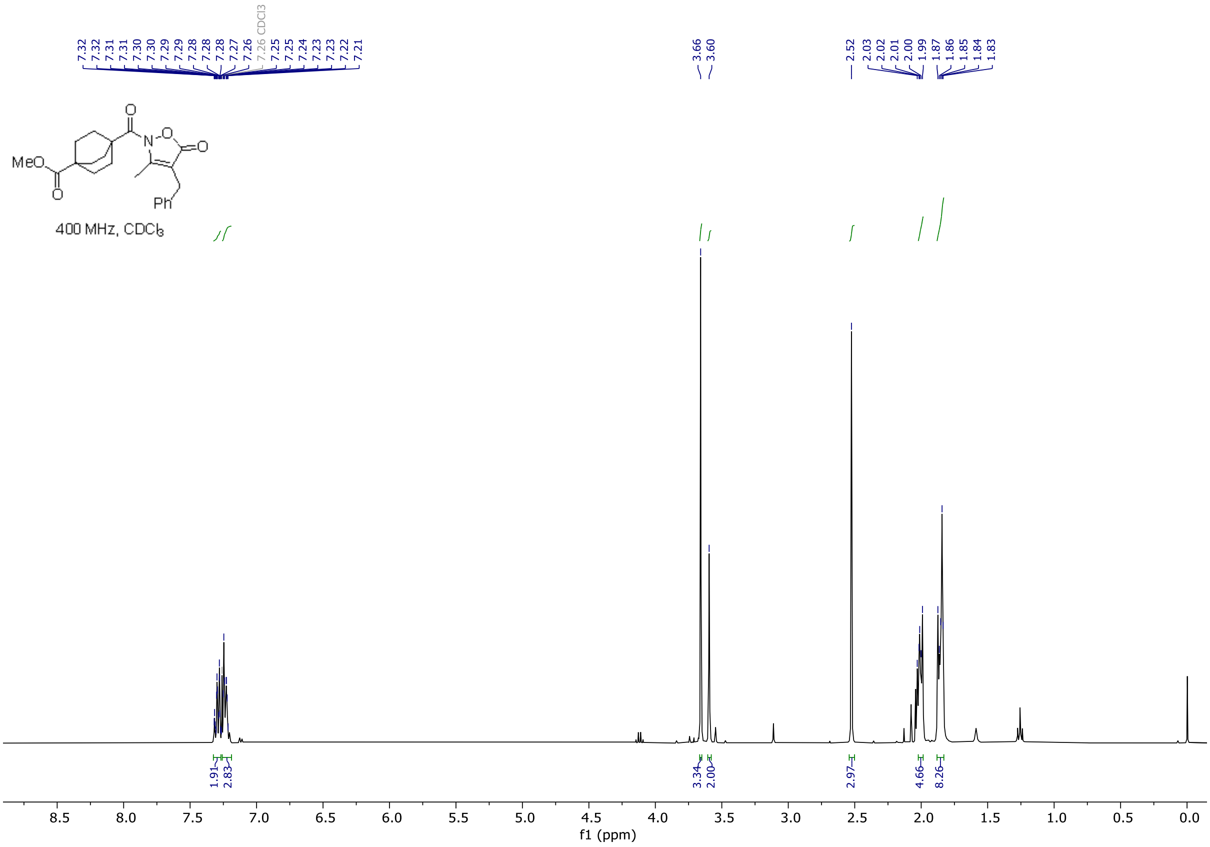

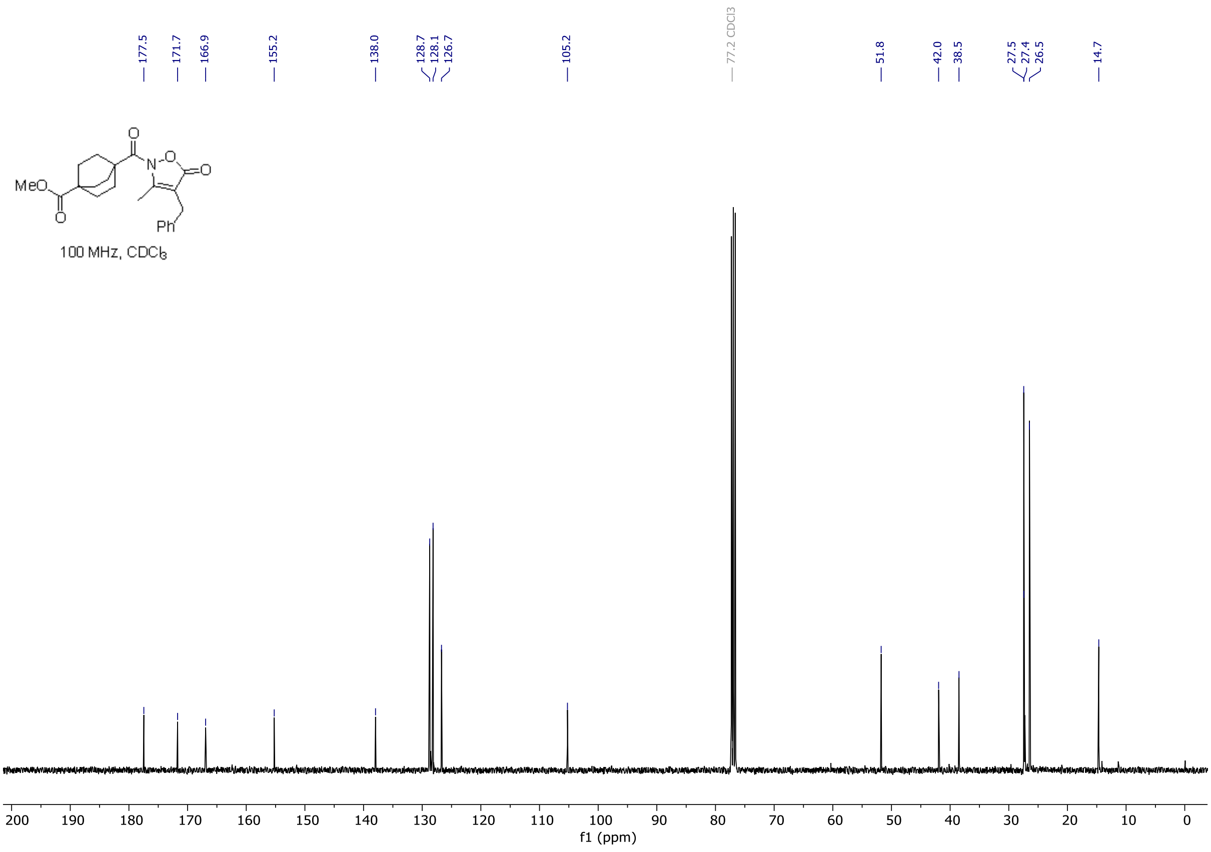

HSQC Data

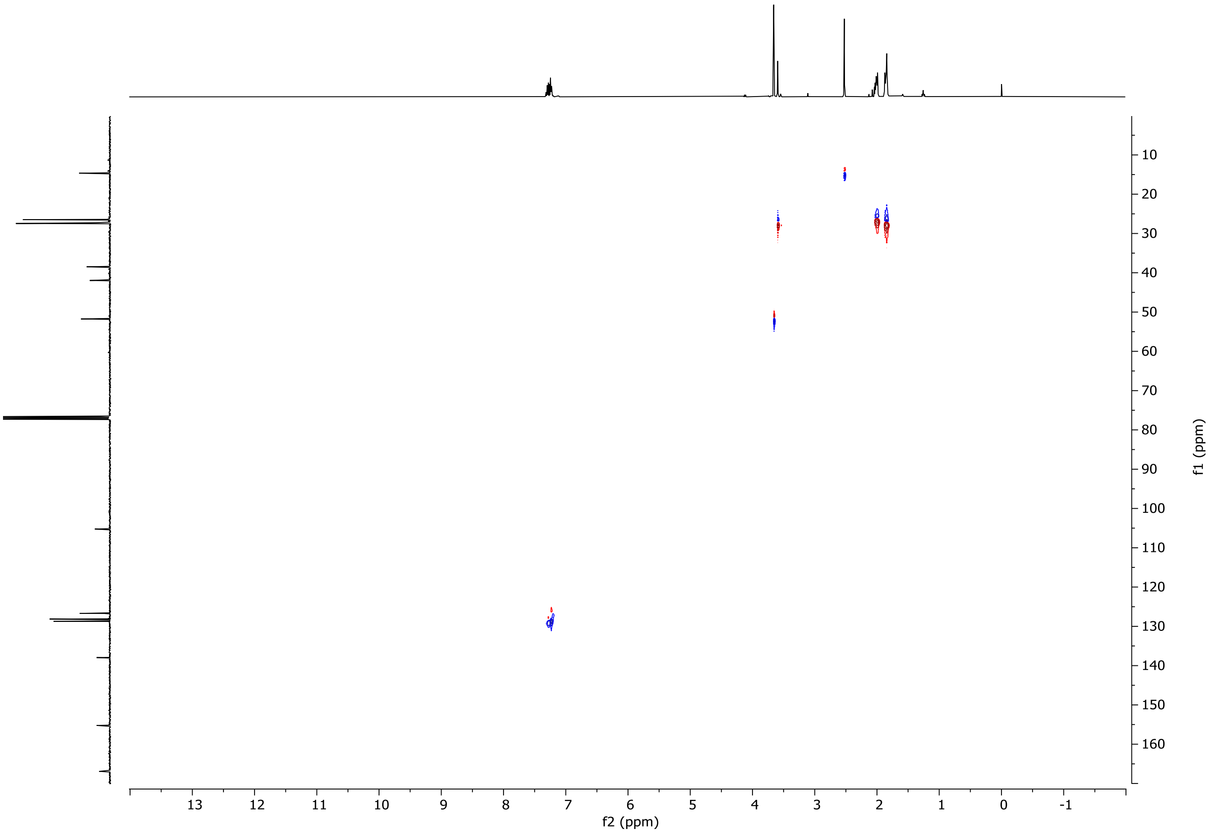

4-Benzyl-3-methyl-2-(3-oxocyclobutane-1-carbonyl)isoxazol-5(2*H*)-one (1j)

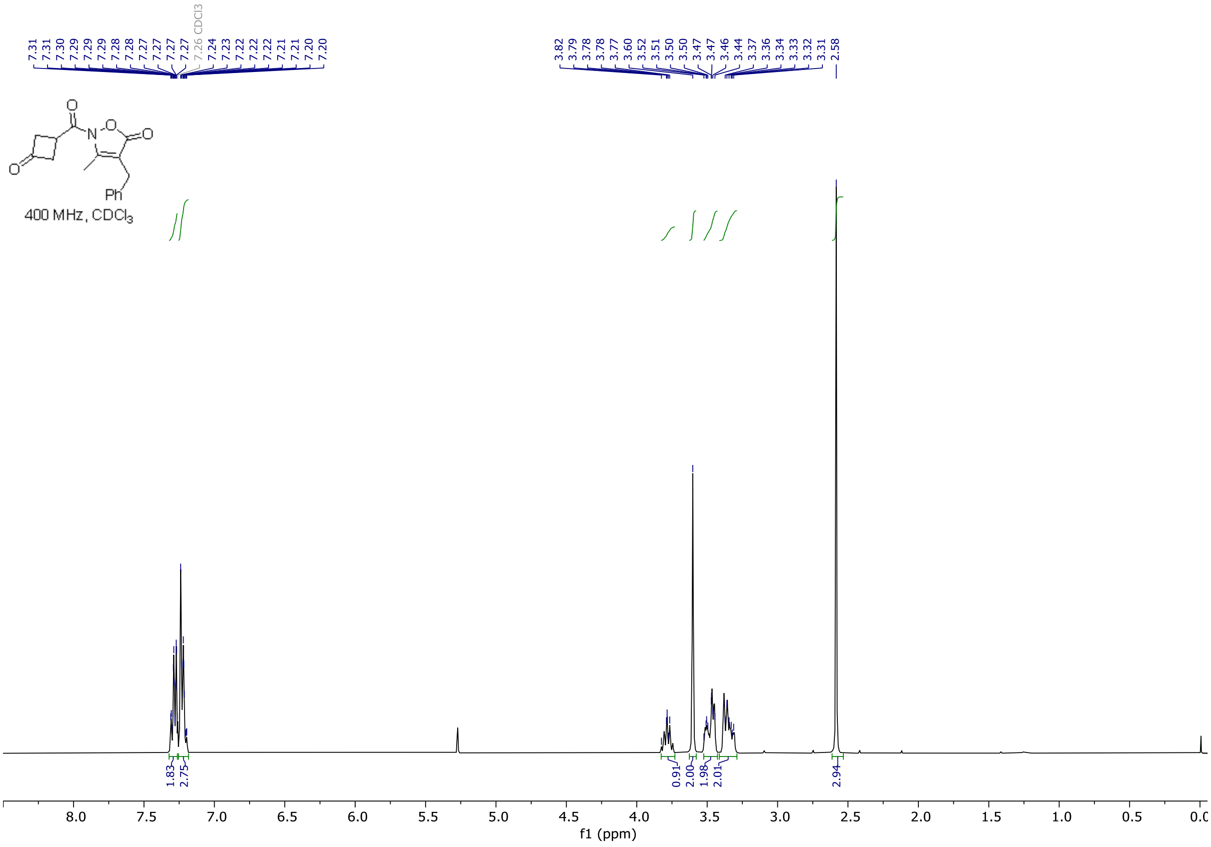

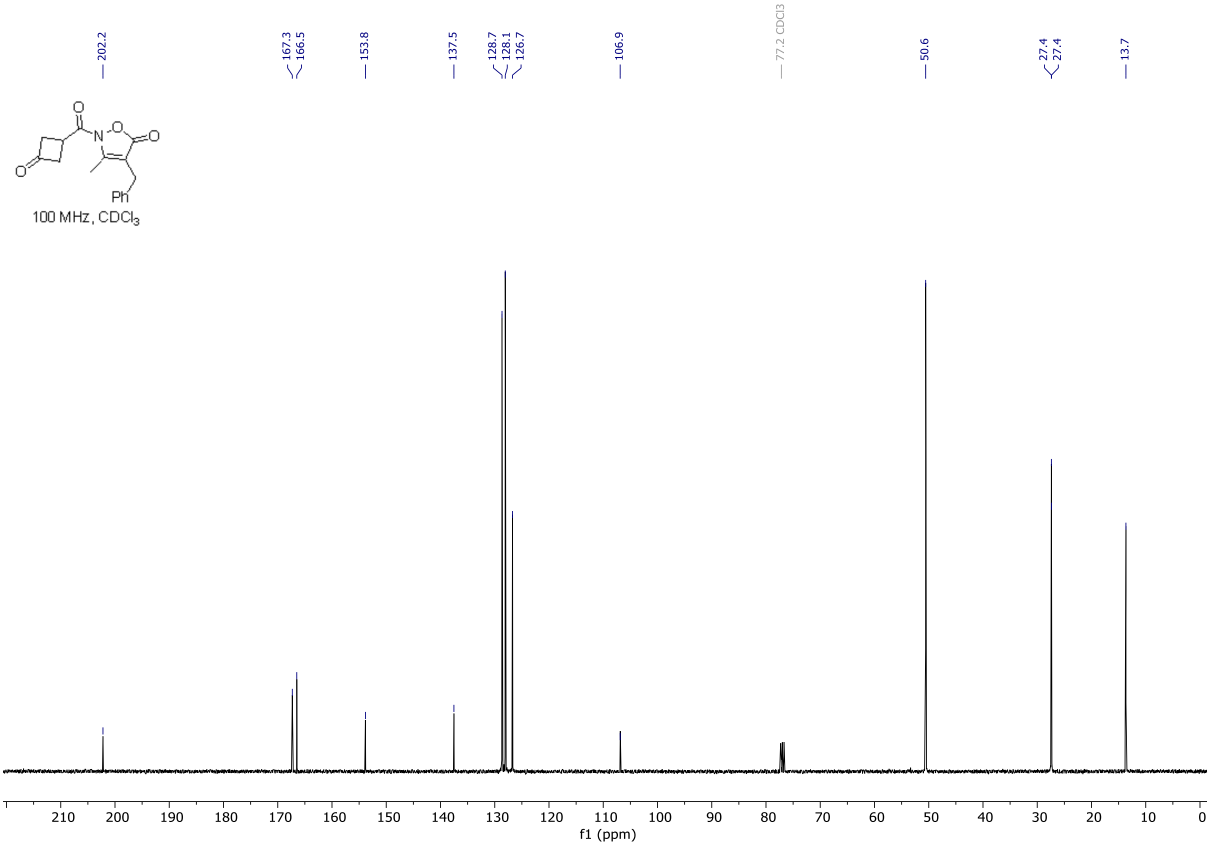
202.2
167.3
166.5
153.8
137.5
128.7
128.1
126.7
106.9
77.2 CDCl3
50.6
27.4
27.4
13.7
100 MHz, CDCl3
f1 (ppm)

HSQC Data

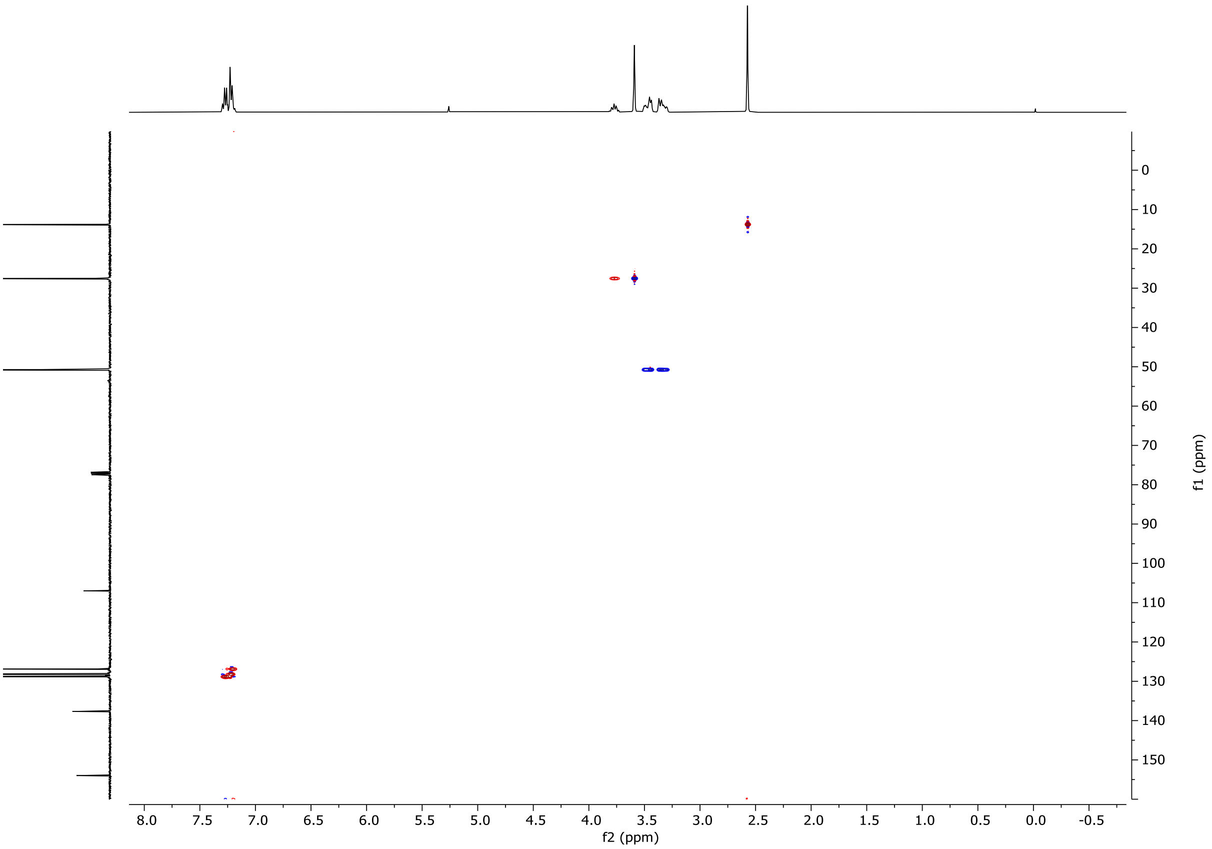
f2 (ppm)
f1 (ppm)

2-((1r,3r,5r,7r)-Adamantane-2-carbonyl)-4-benzyl-3-methylisoxazol-5(2*H*)-one (1k)

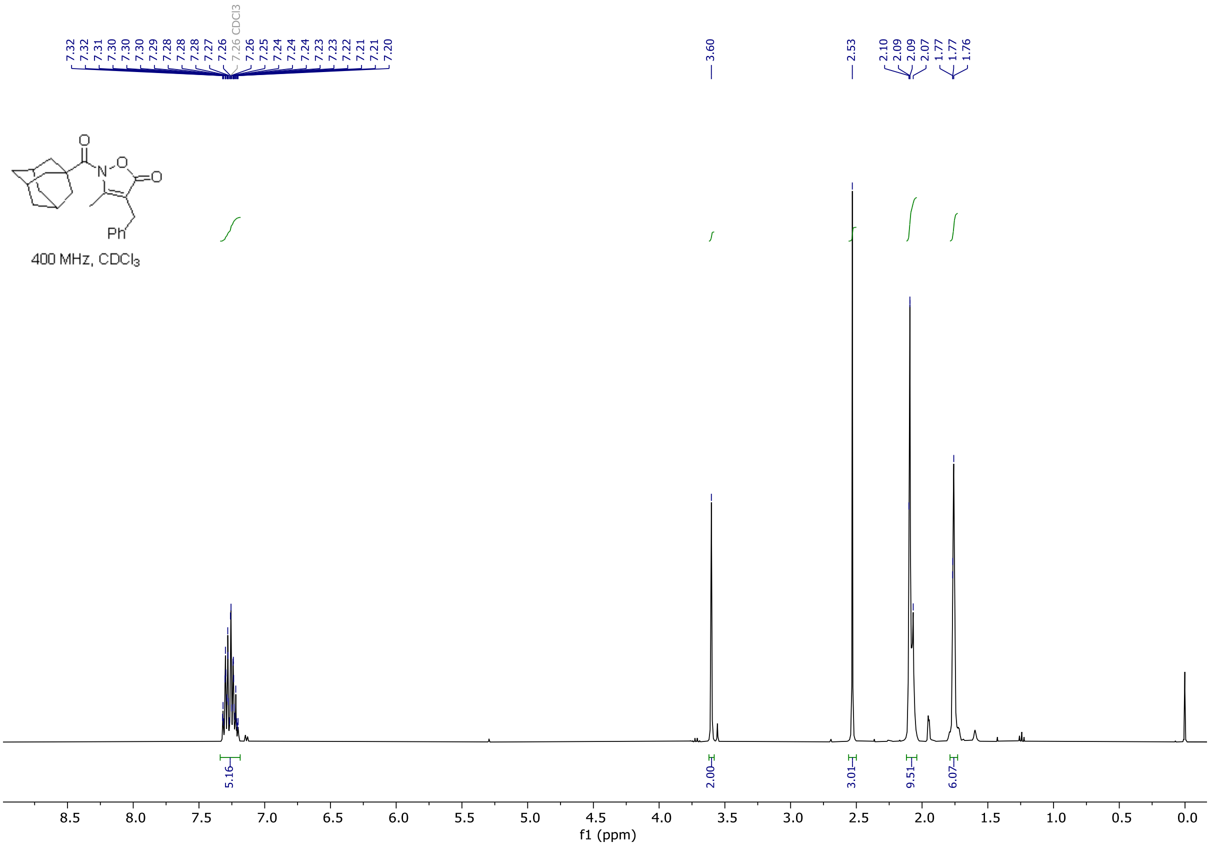

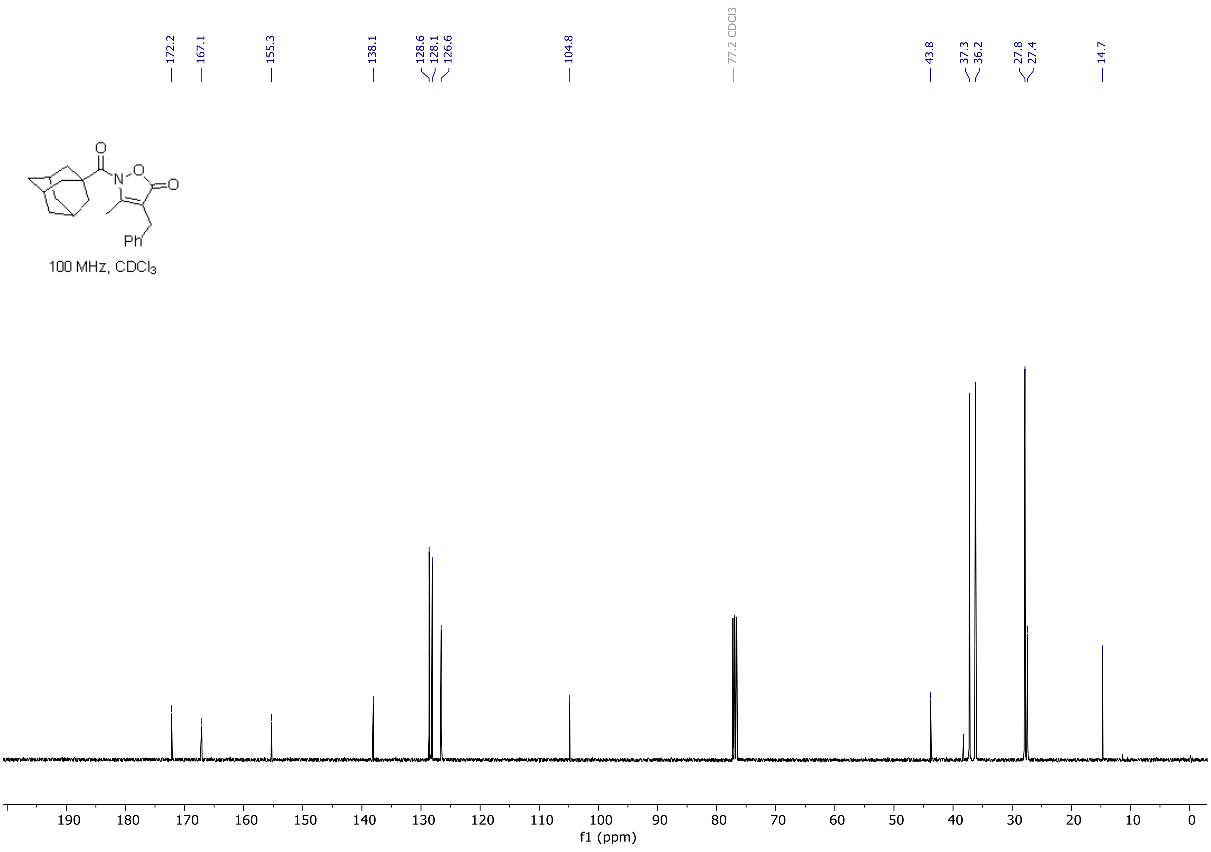

HSQC Data

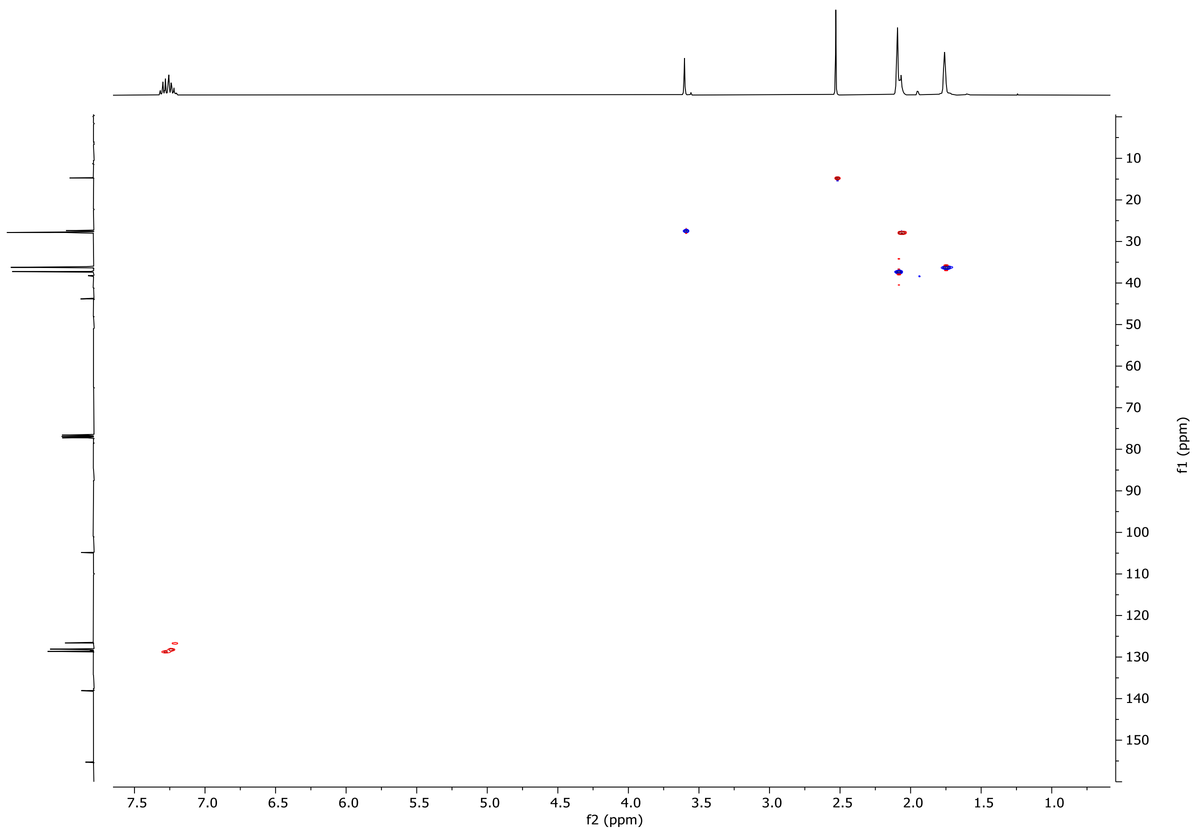

Isobutyl 4-benzyl-3-methyl-5-oxoisoxazole-2(5*H*)-carboxylate (1l)

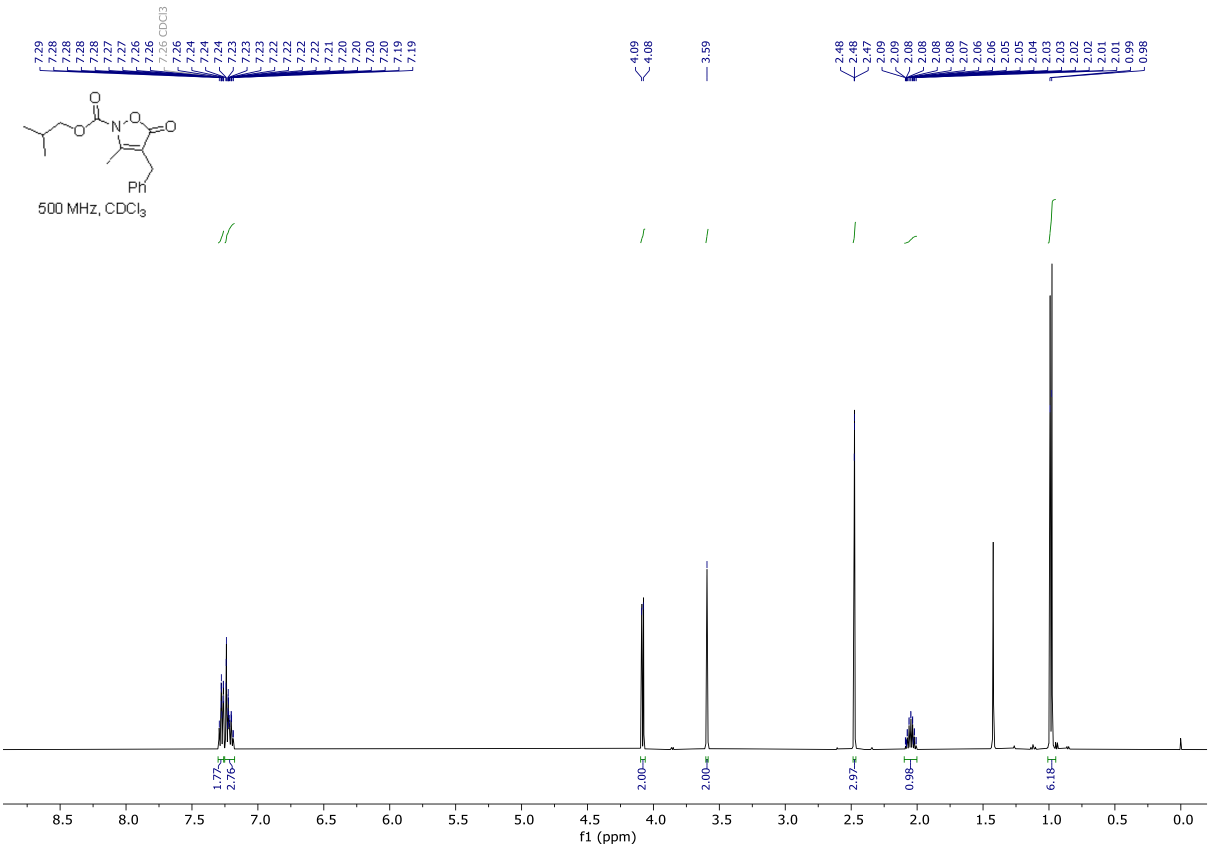

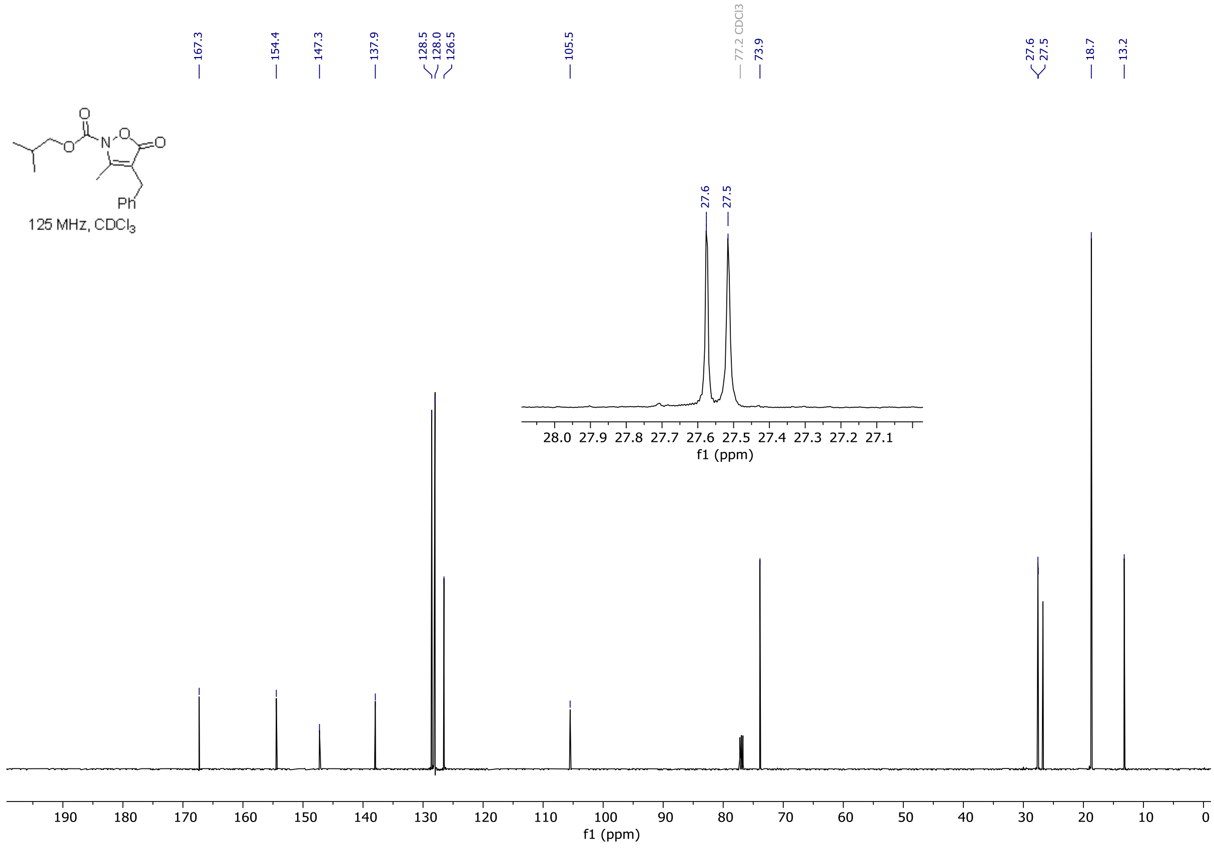

167.3
154.4
147.3
137.9
128.5
128.0
126.5
105.5
77.2 CDCl3
73.9
27.6
27.5
18.7
13.2
125 MHz, CDCl3
f1 (ppm)

HSQC Data

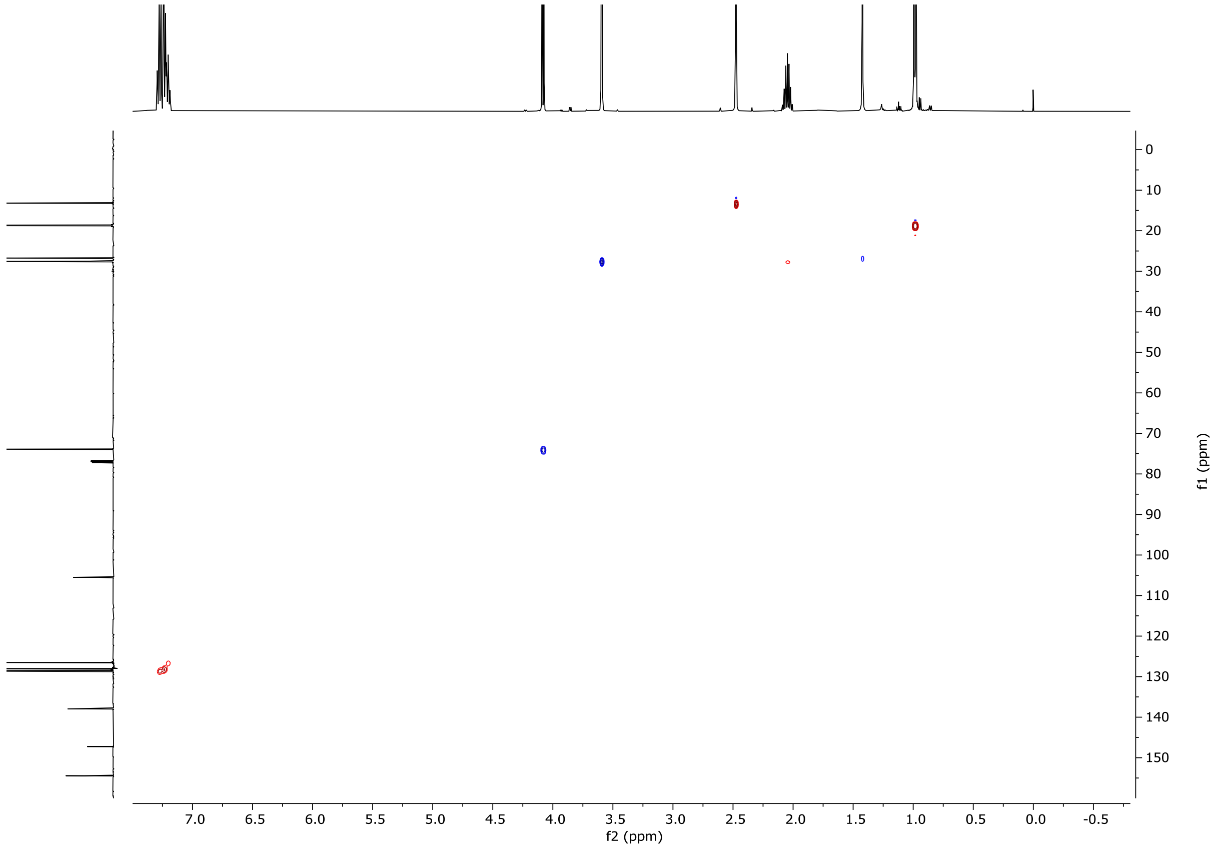

f2 (ppm)
f1 (ppm)

4-Benzyl-3-methyl-2-(morpholine-4-carbonyl)isoxazol-5(2*H*)-one (1m)

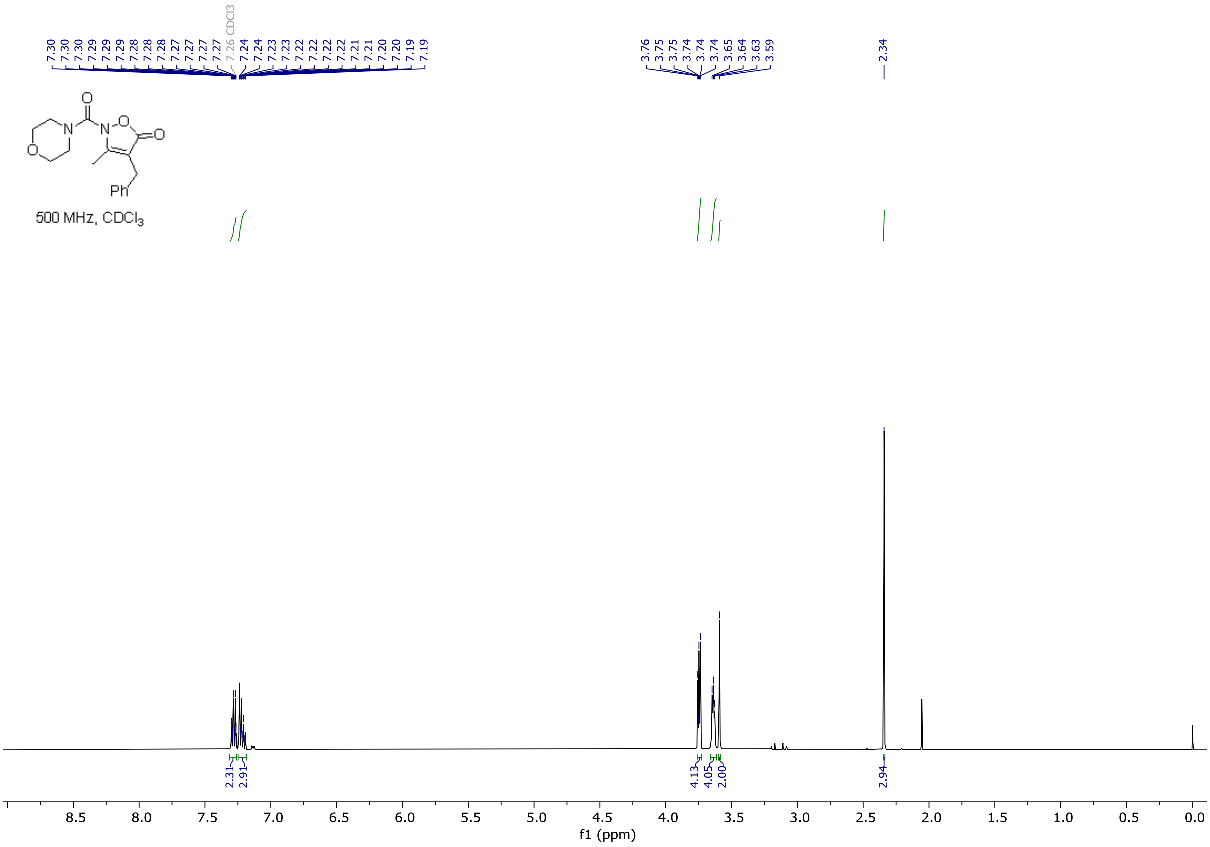

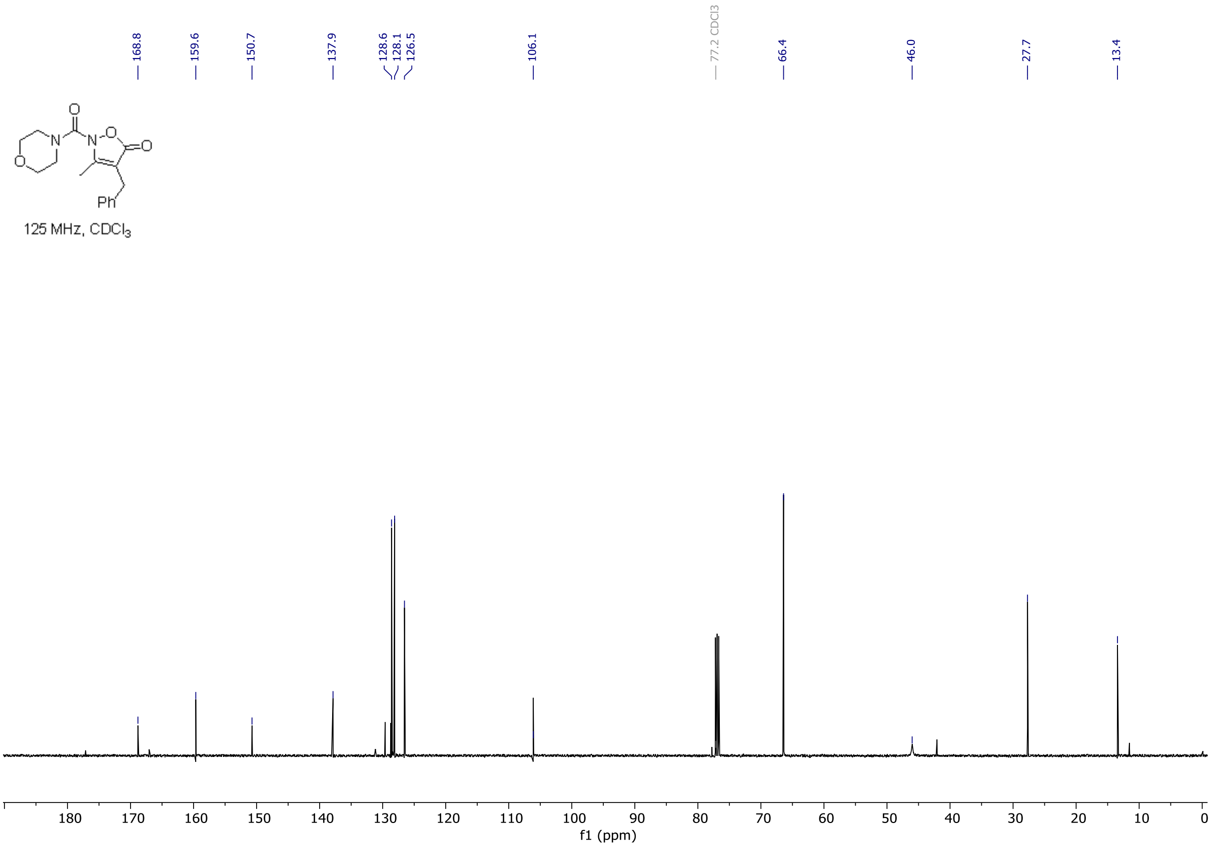

HSQC Data

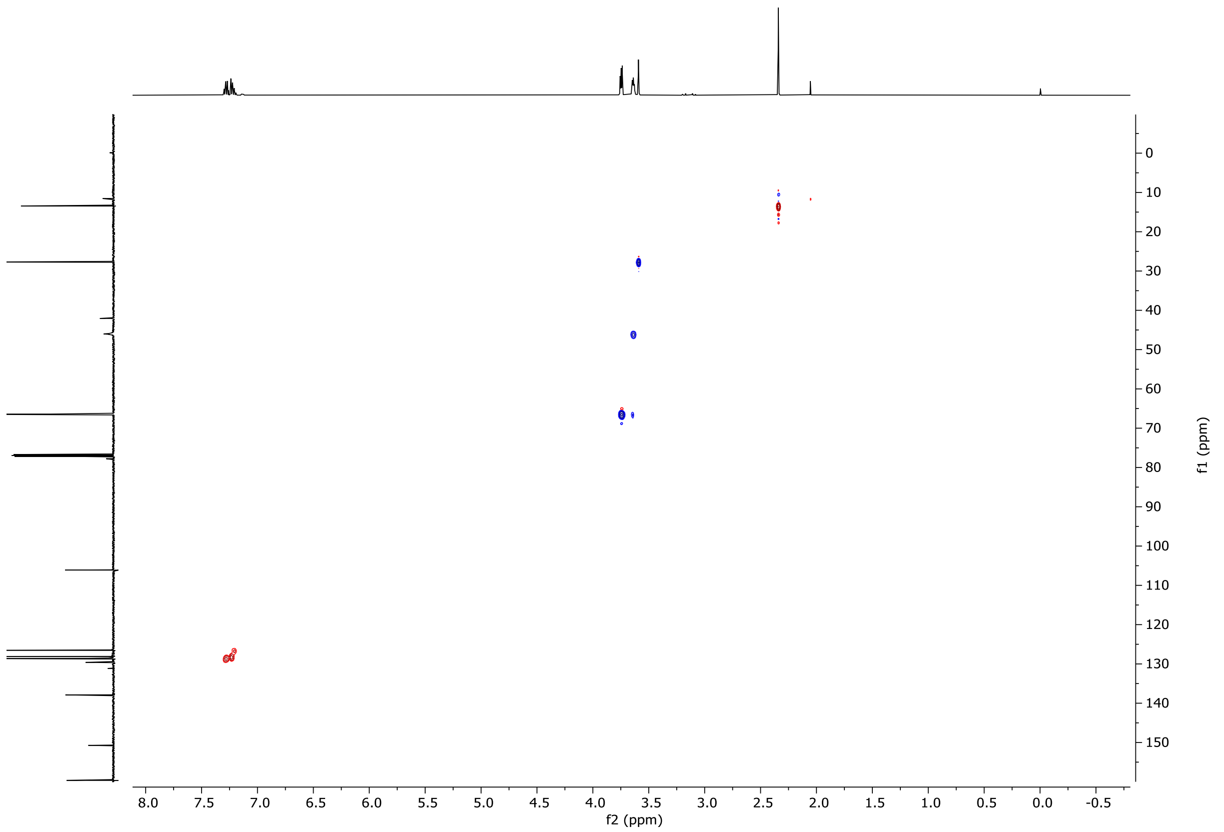

2-Acetyl-4-benzyl-3-cyclopropylisoxazol-5(2*H*)-one (1n)

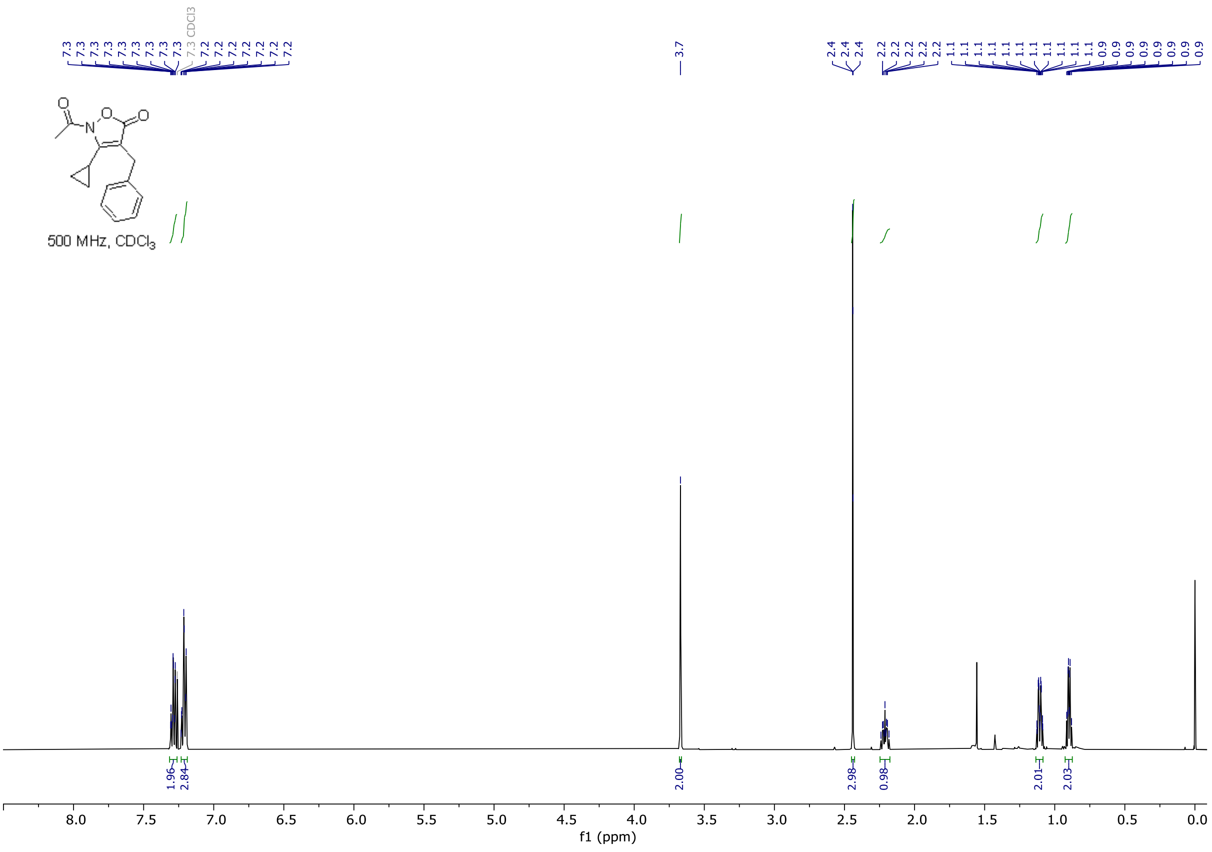

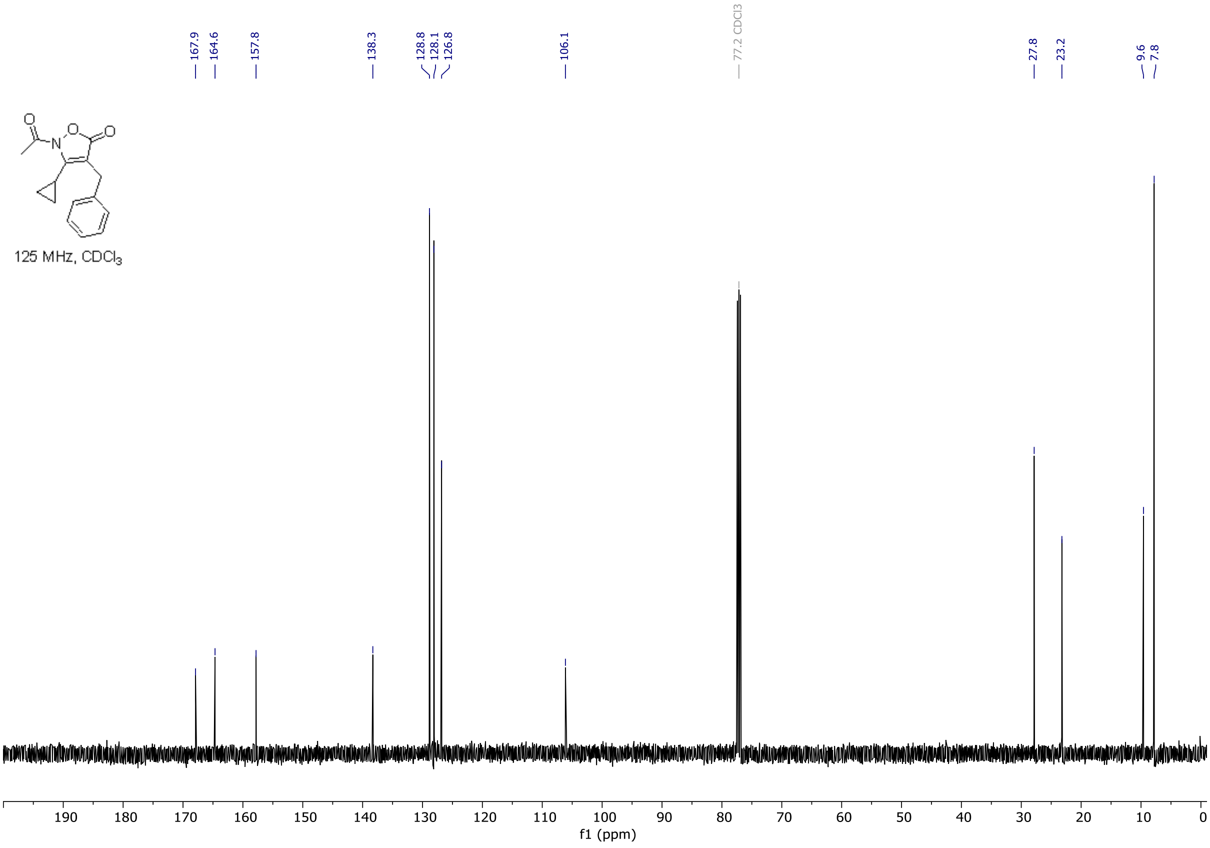
167.9
164.6
157.8
138.3
128.8
128.1
126.8
106.1
77.2 CDCl3
27.8
23.2
9.6
7.8
125 MHz, CDCl$_3$
f1 (ppm)

HSQC Data

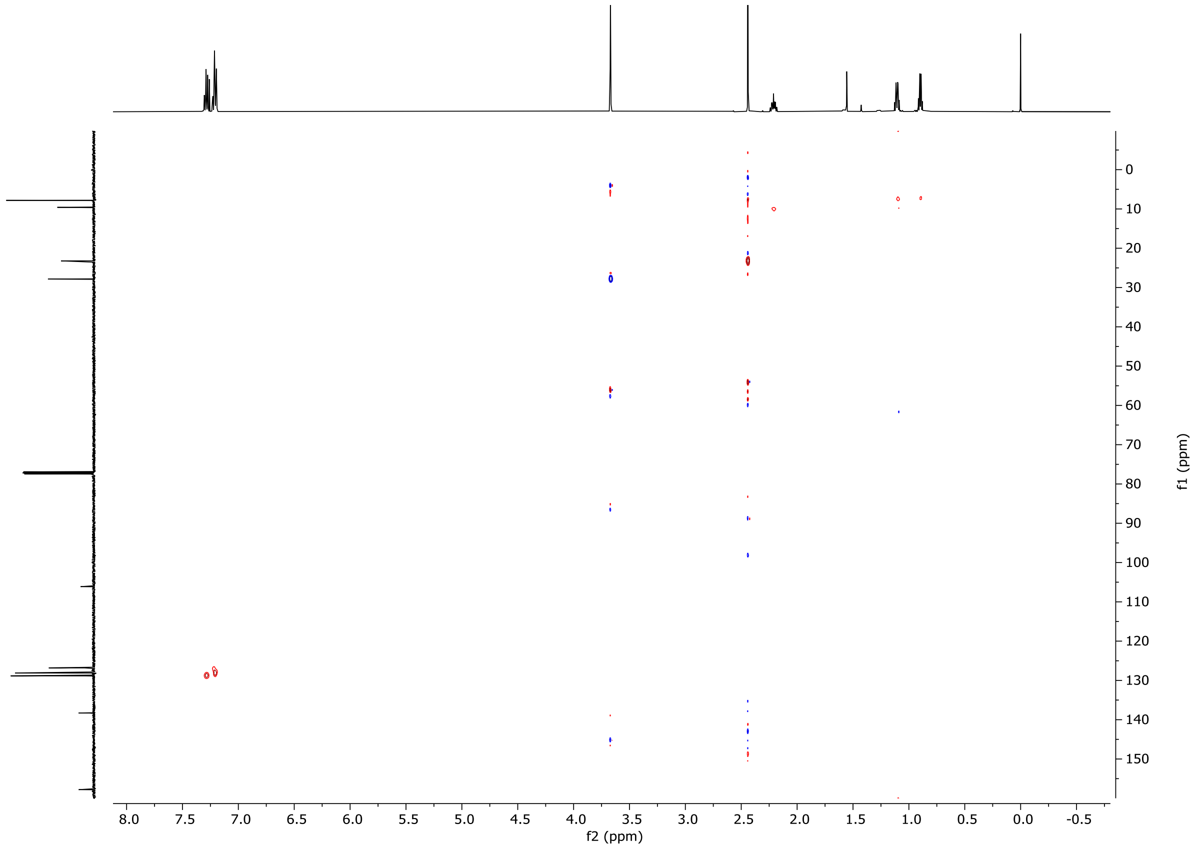
f2 (ppm)
f1 (ppm)

4-(2-Hydroxybenzyl)-2-isobutyryl-3-methylisoxazol-5(2*H*)-one (1o)

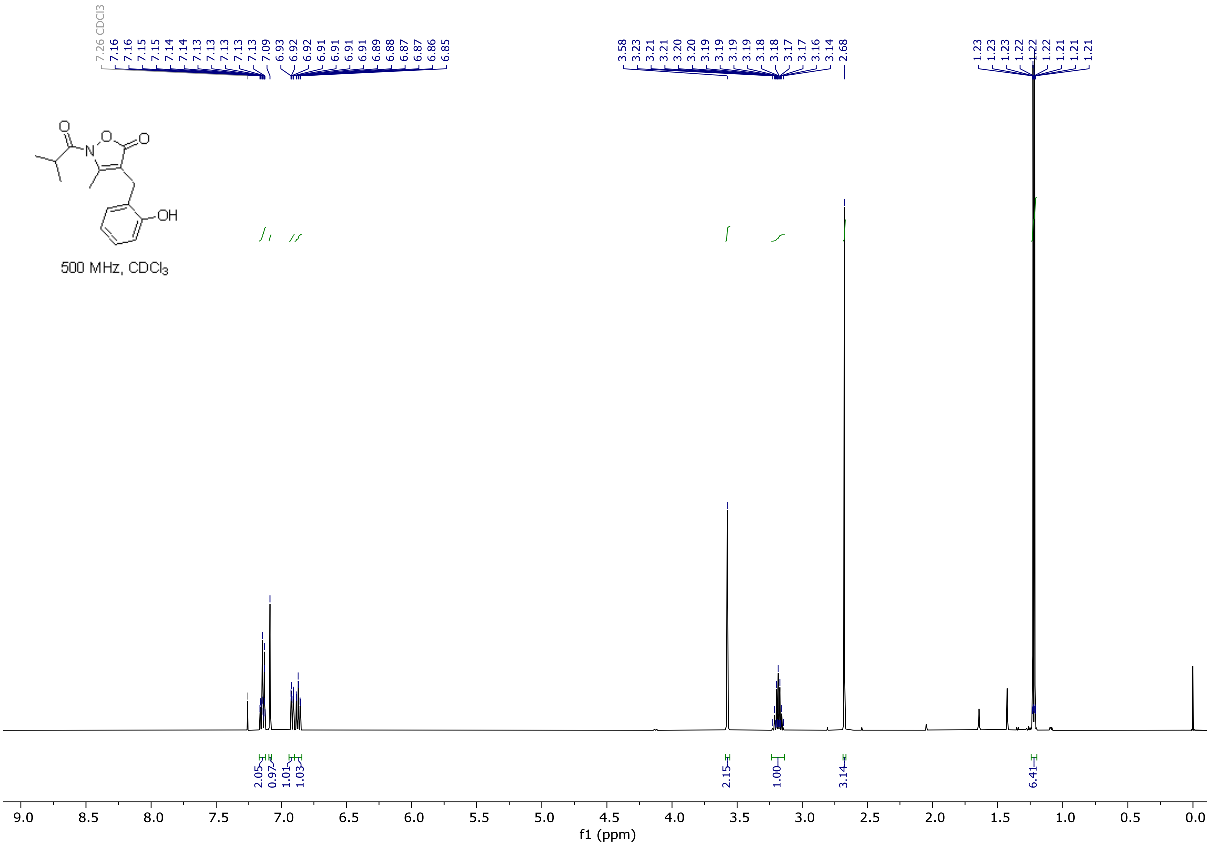

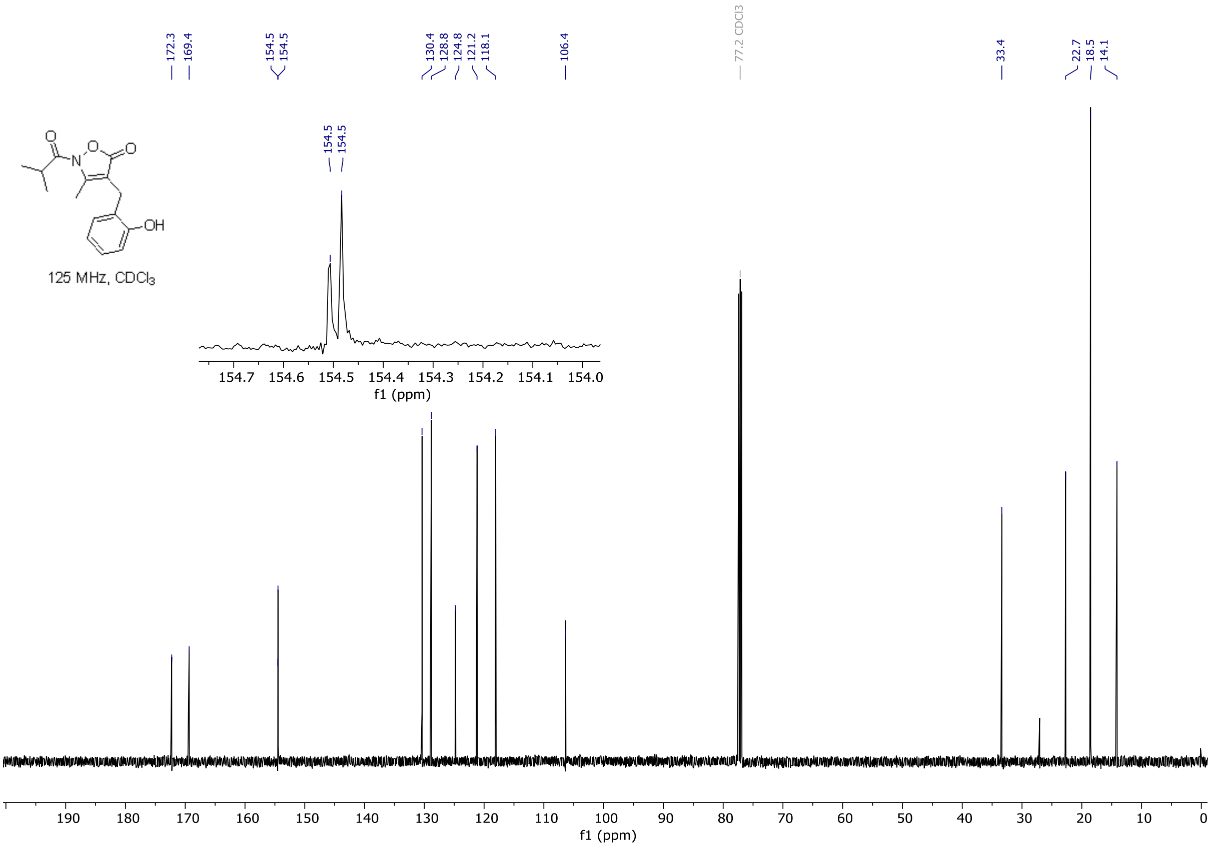

HSQC Data

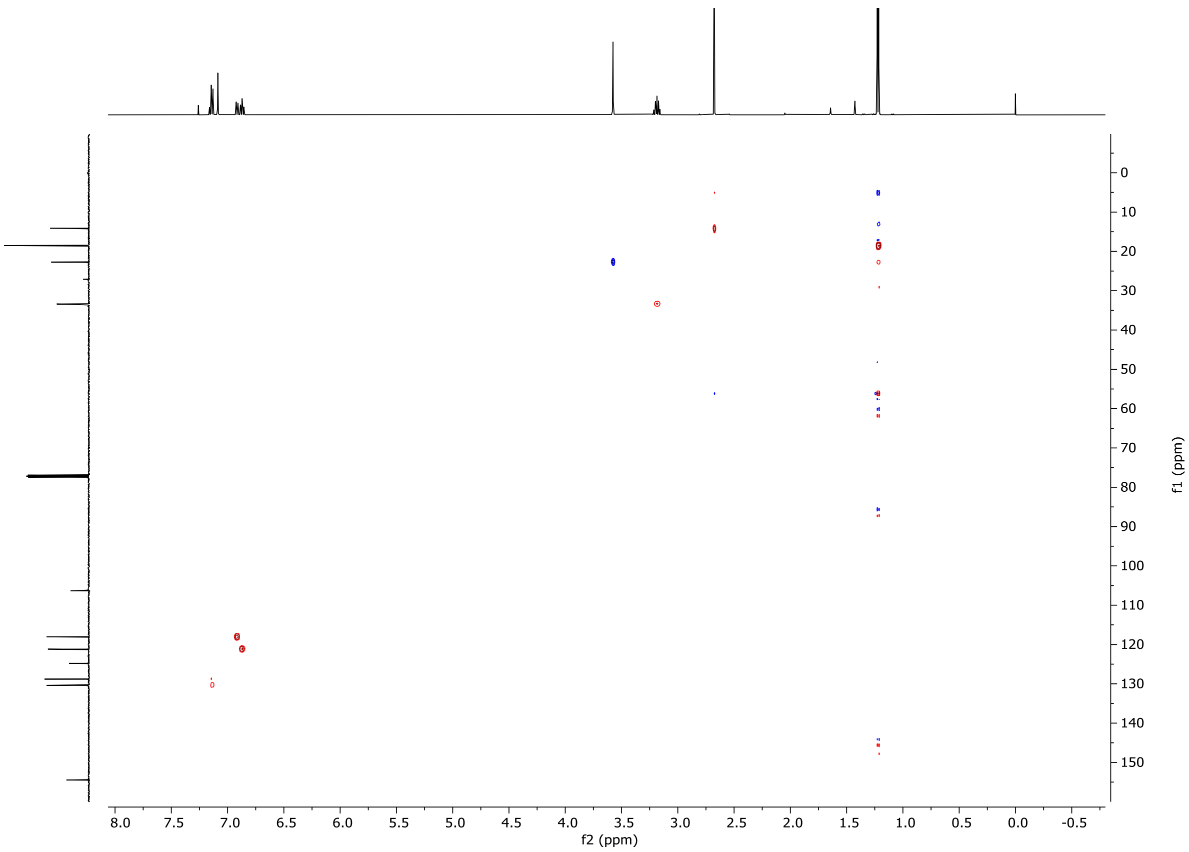

4-(4-Bromobenzyl)-2-isobutyryl-3-methylisoxazol-5(2*H*)-one (1p)

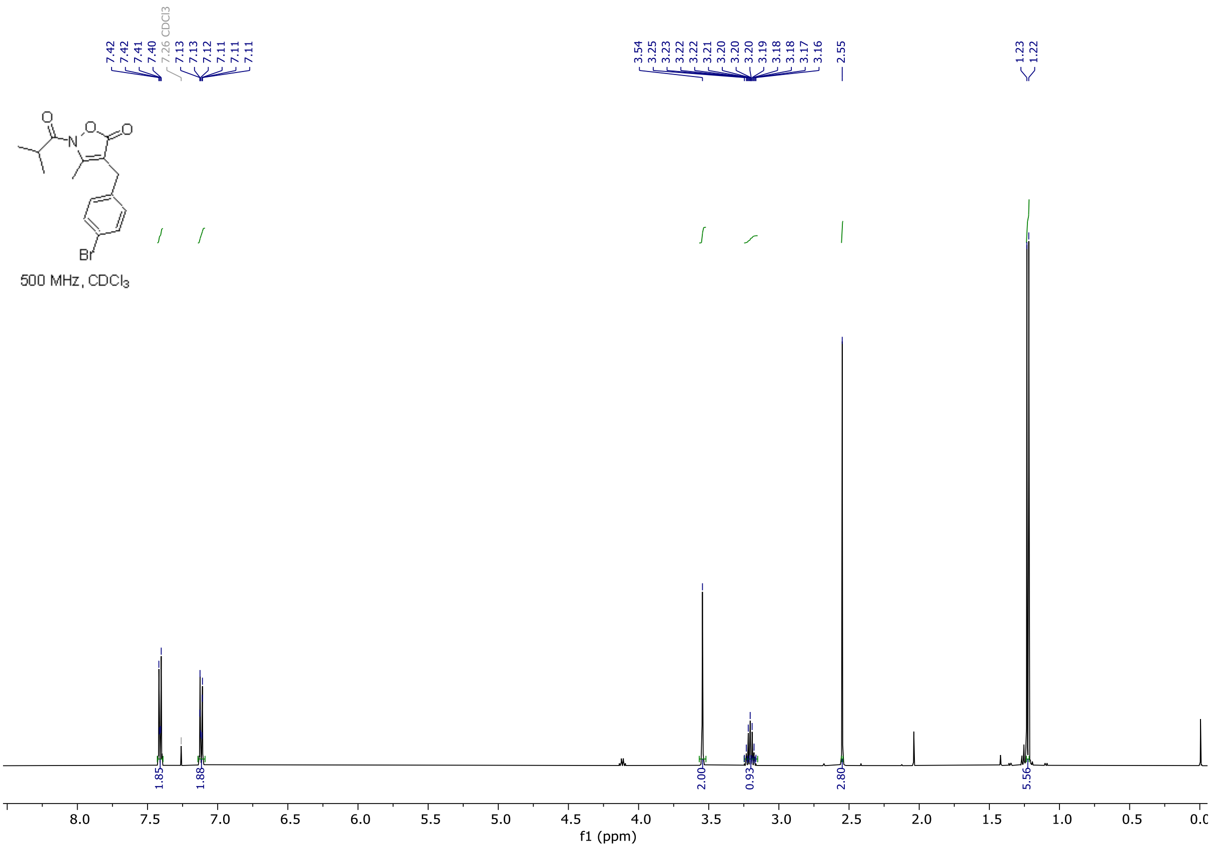

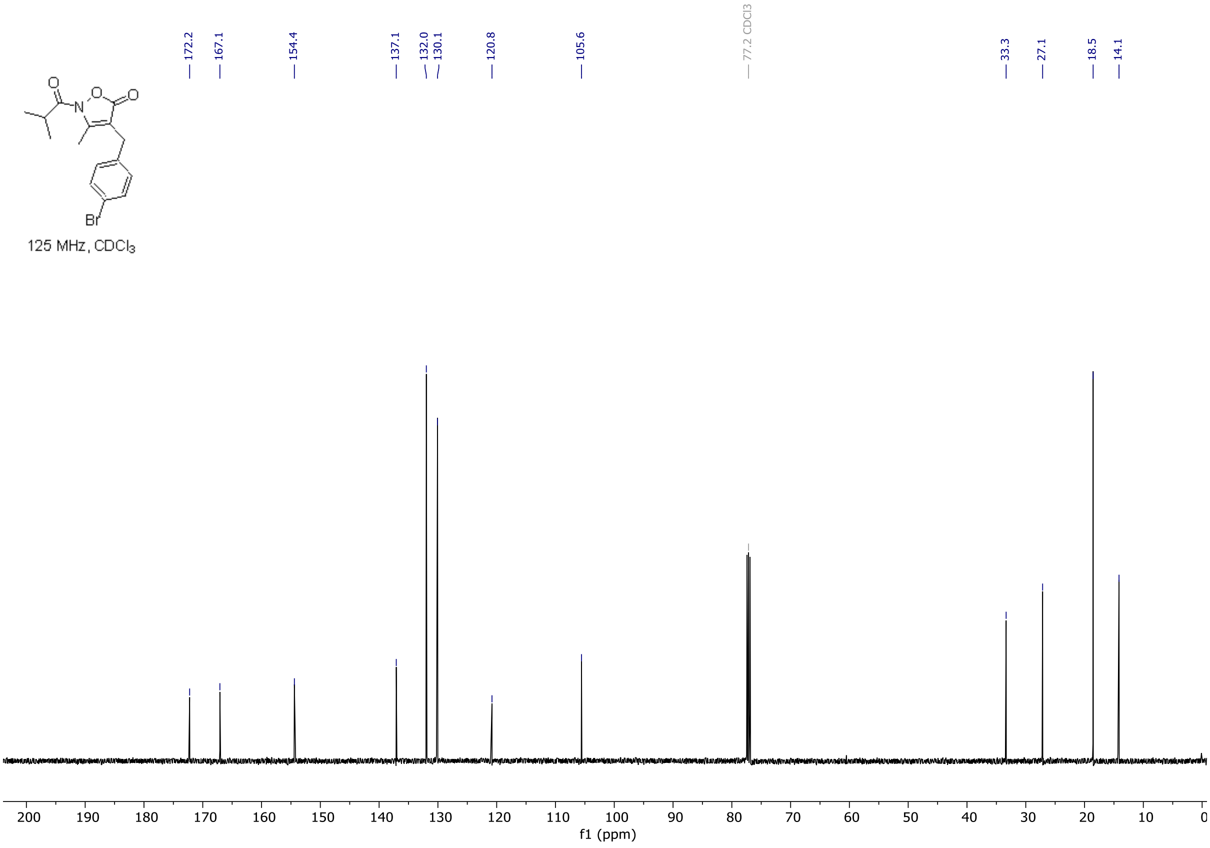

172.2
167.1
154.4
137.1
132.0
130.1
120.8
105.6
77.2 CDCl3
33.3
27.1
18.5
14.1
Br
125 MHz, CDCl3
f1 (ppm)

HSQC Data

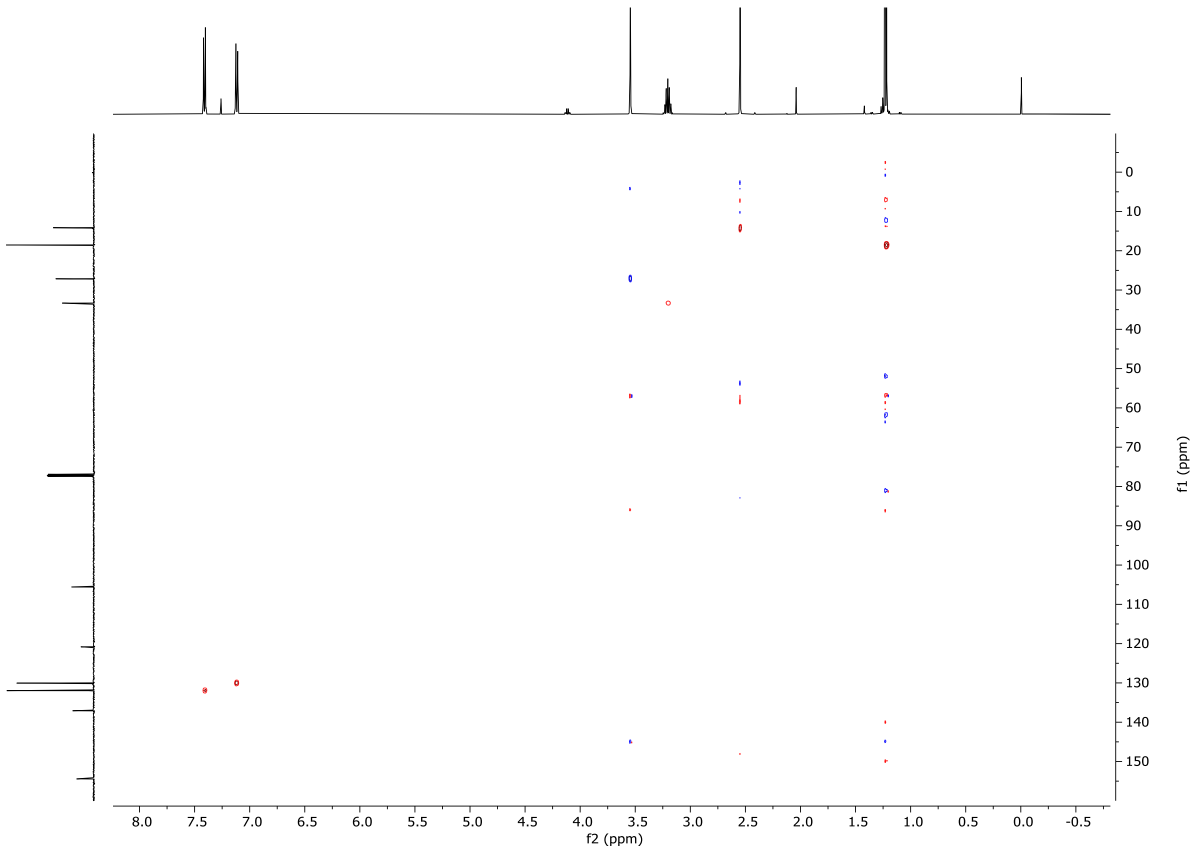

f2 (ppm)
f1 (ppm)

4-((1*H*-Indol-3-yl)methyl)-2-isobutyryl-3-methylisoxazol-5(2*H*)-one (1q)

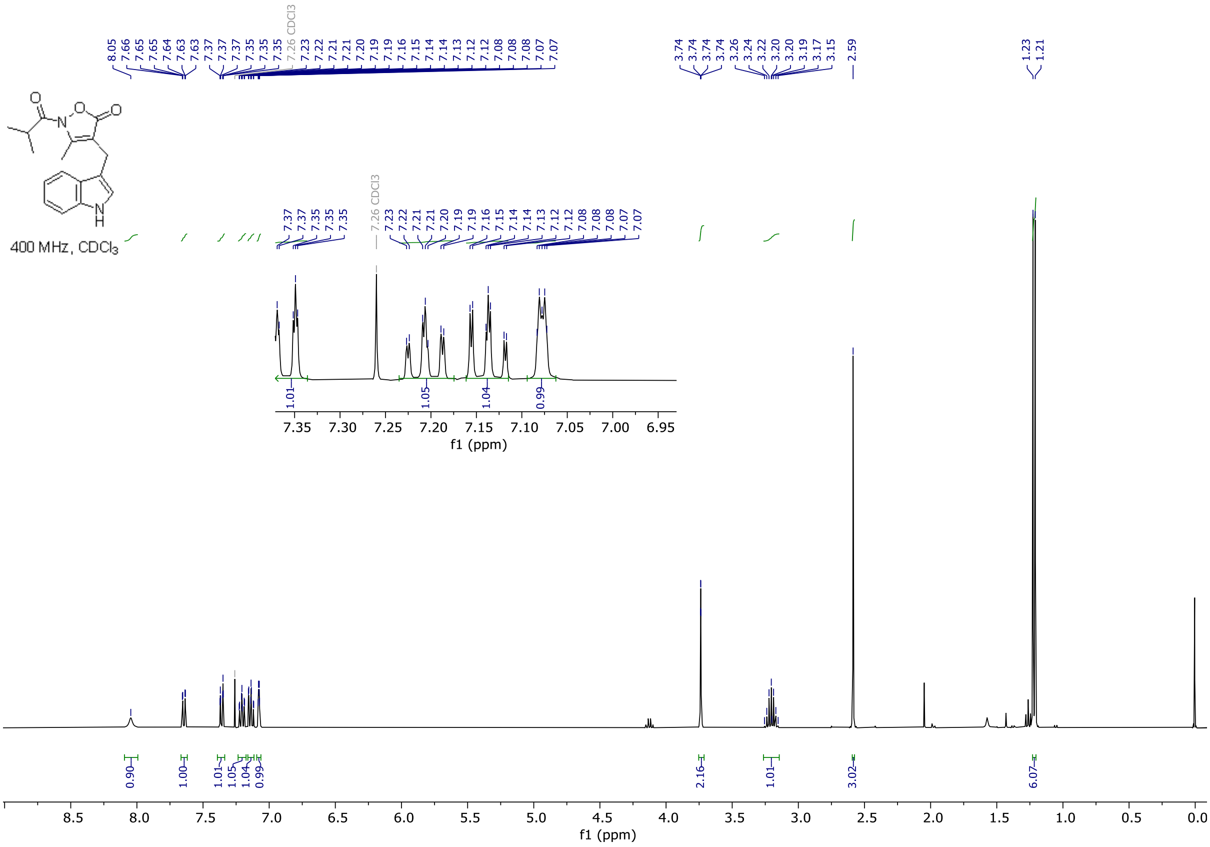

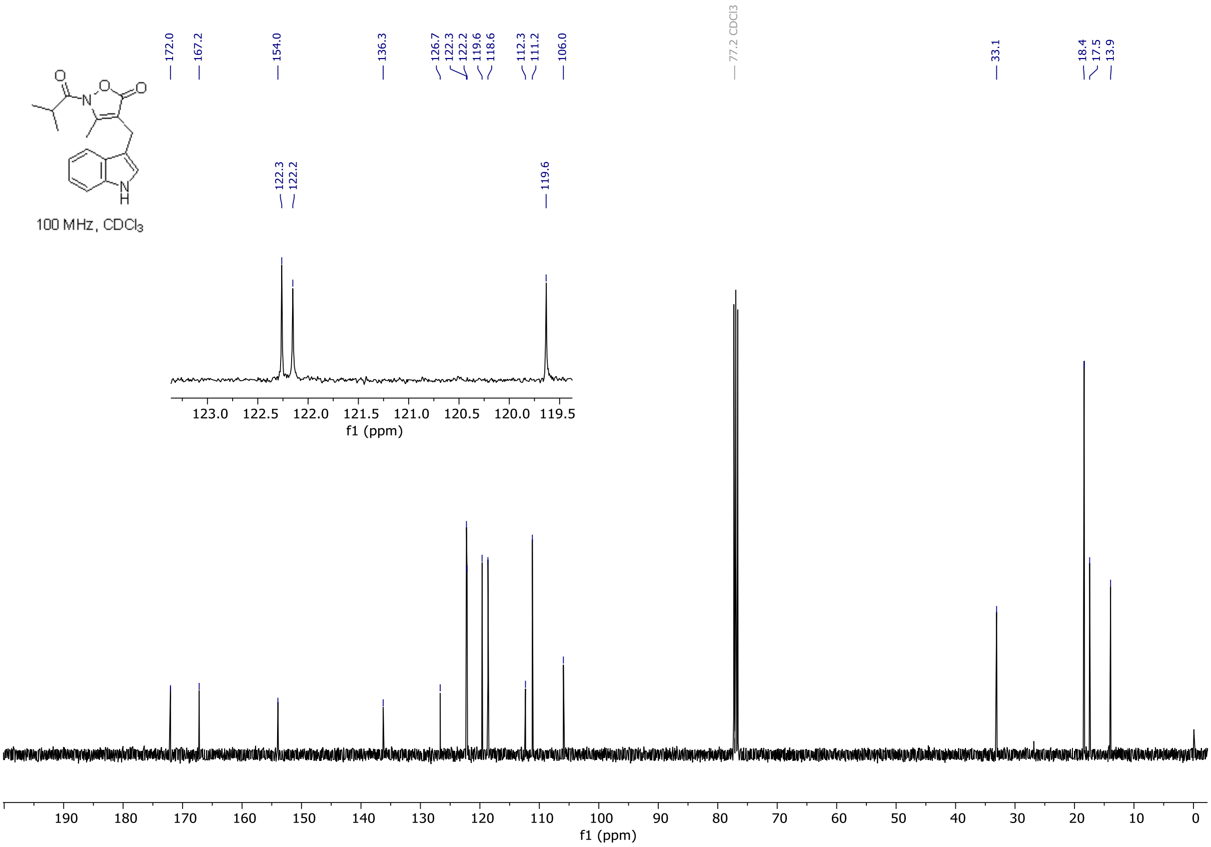

HSQC Data

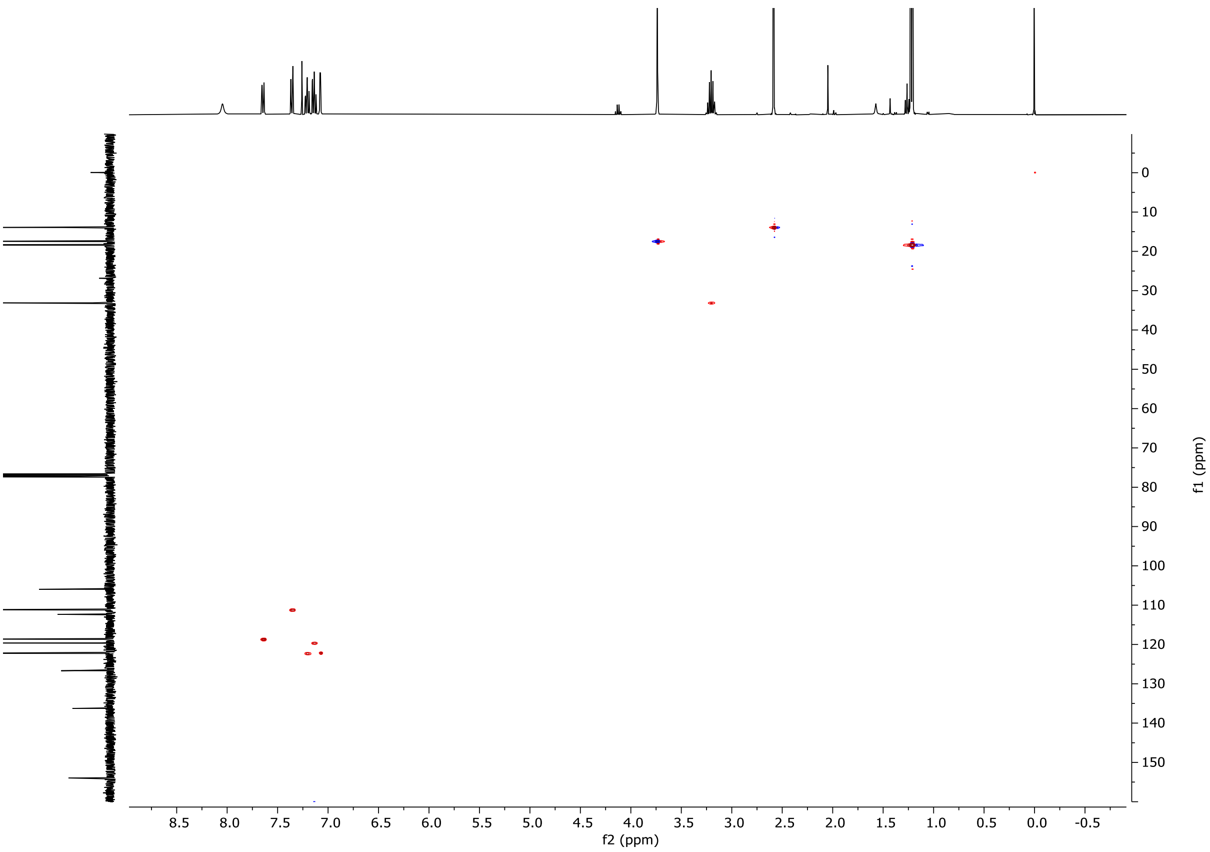

tert-Butyl 3-((2-isobutyryl-3-methyl-5-oxo-2,5-dihydroisoxazol-4-yl)methyl)-1*H*-indole-1-carboxylate (1r)

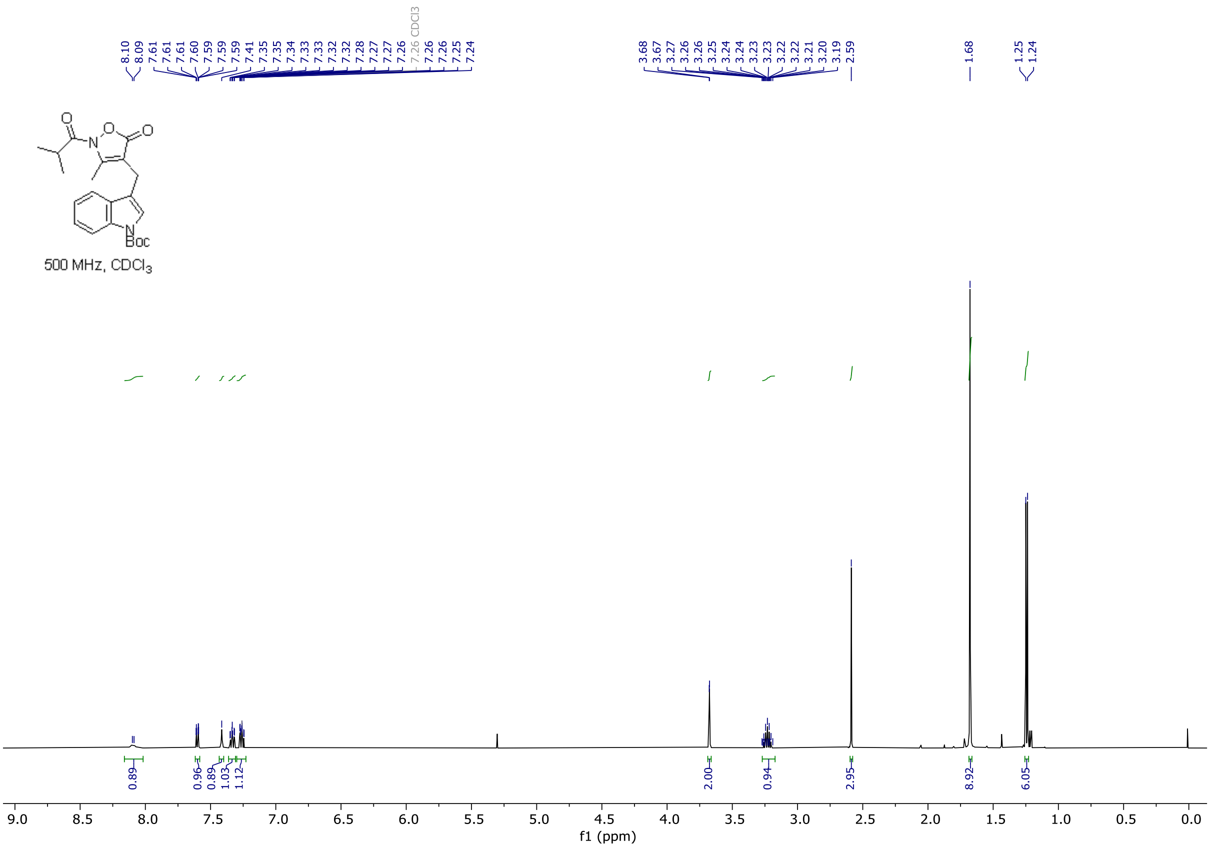

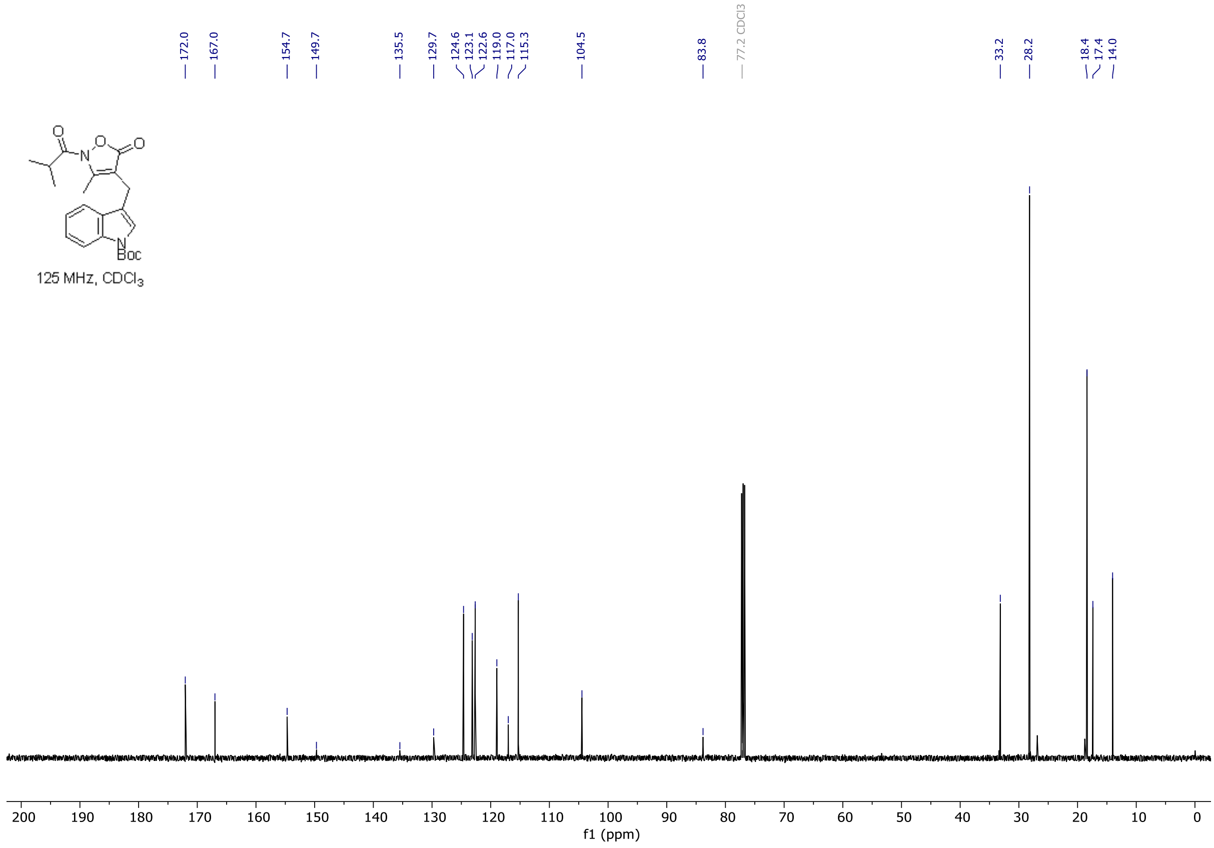
172.0
167.0
154.7
149.7
135.5
129.7
124.6
123.1
122.6
119.0
117.0
115.3
104.5
83.8
77.2 CDCl3
33.2
28.2
18.4
17.4
14.0
O
N
O
O
Boc
N
125 MHz, $CDCl_3$
f1 (ppm)

HSQC Data

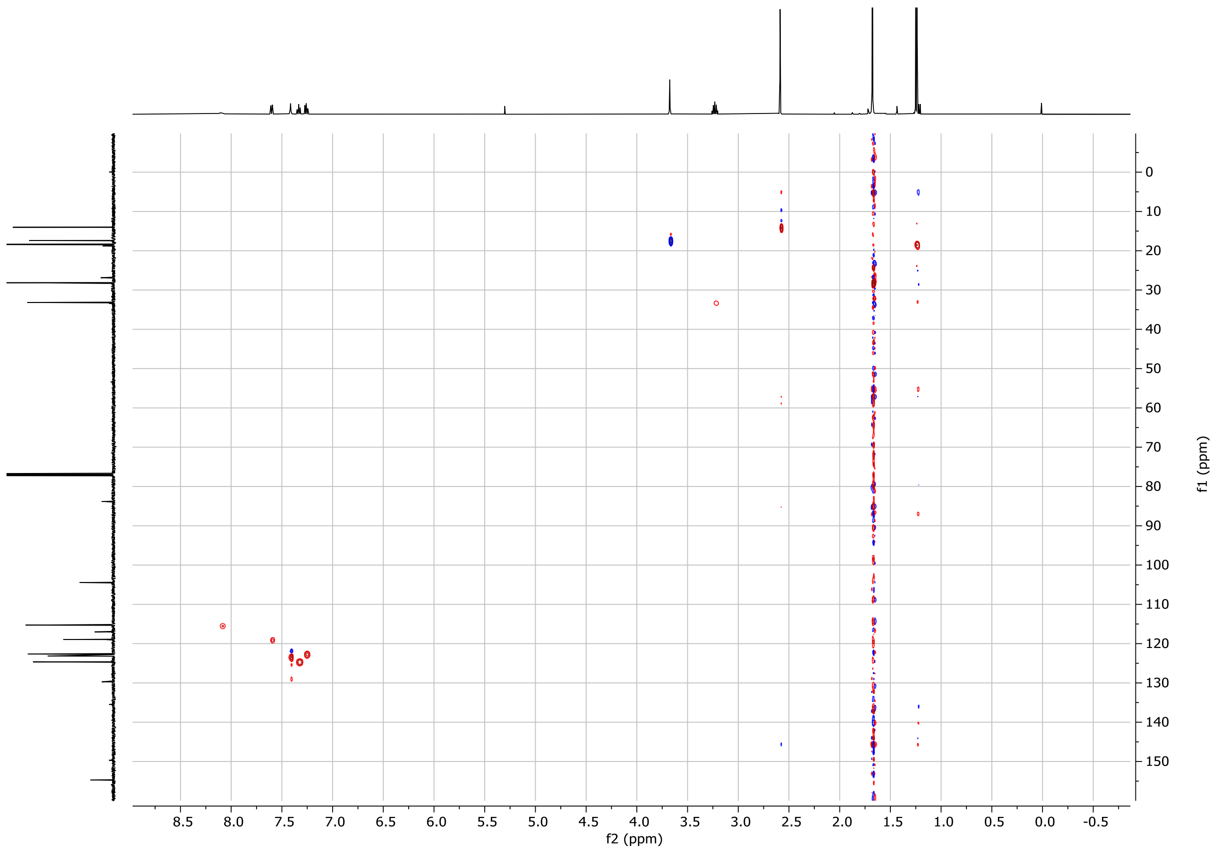
f2 (ppm)
f1 (ppm)

2-Isobutyryl-3-methyl-4-(thiophen-2-ylmethyl)isoxazol-5(2*H*)-one (1s)

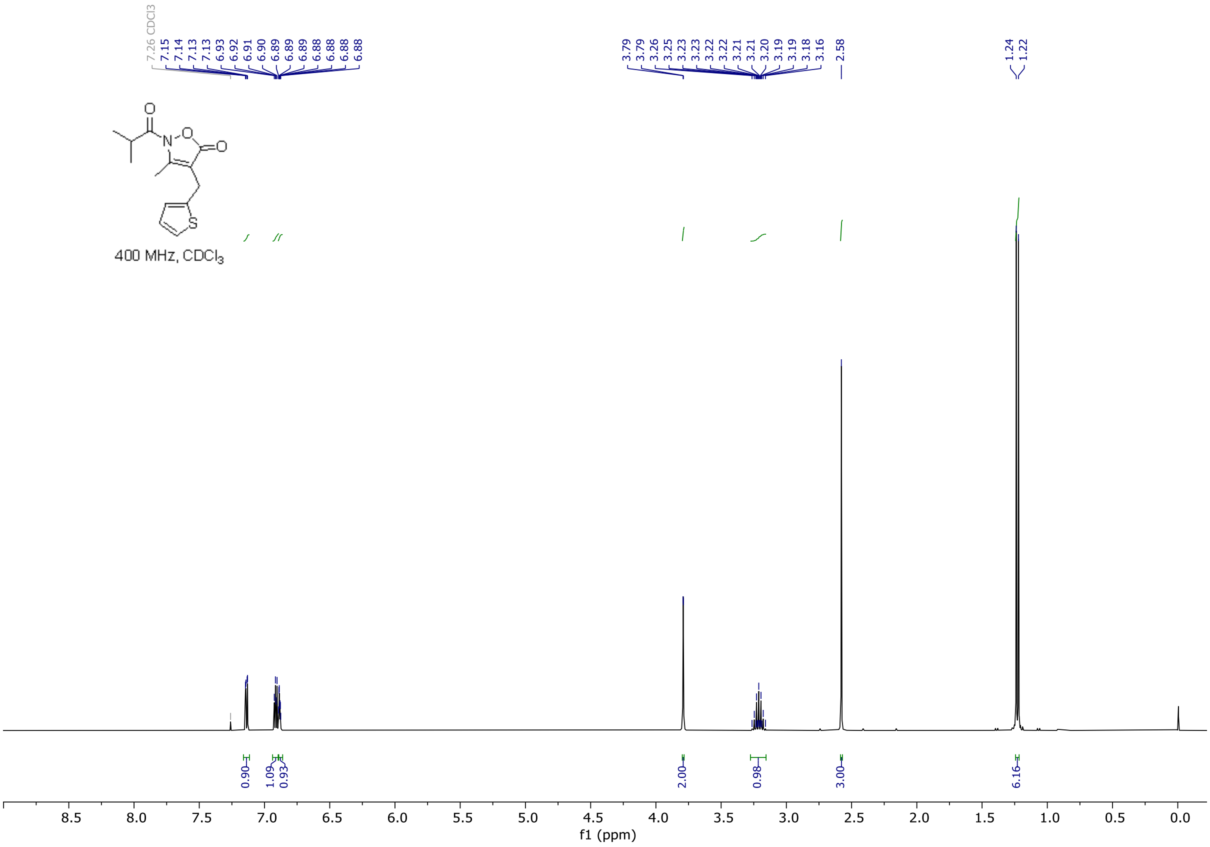

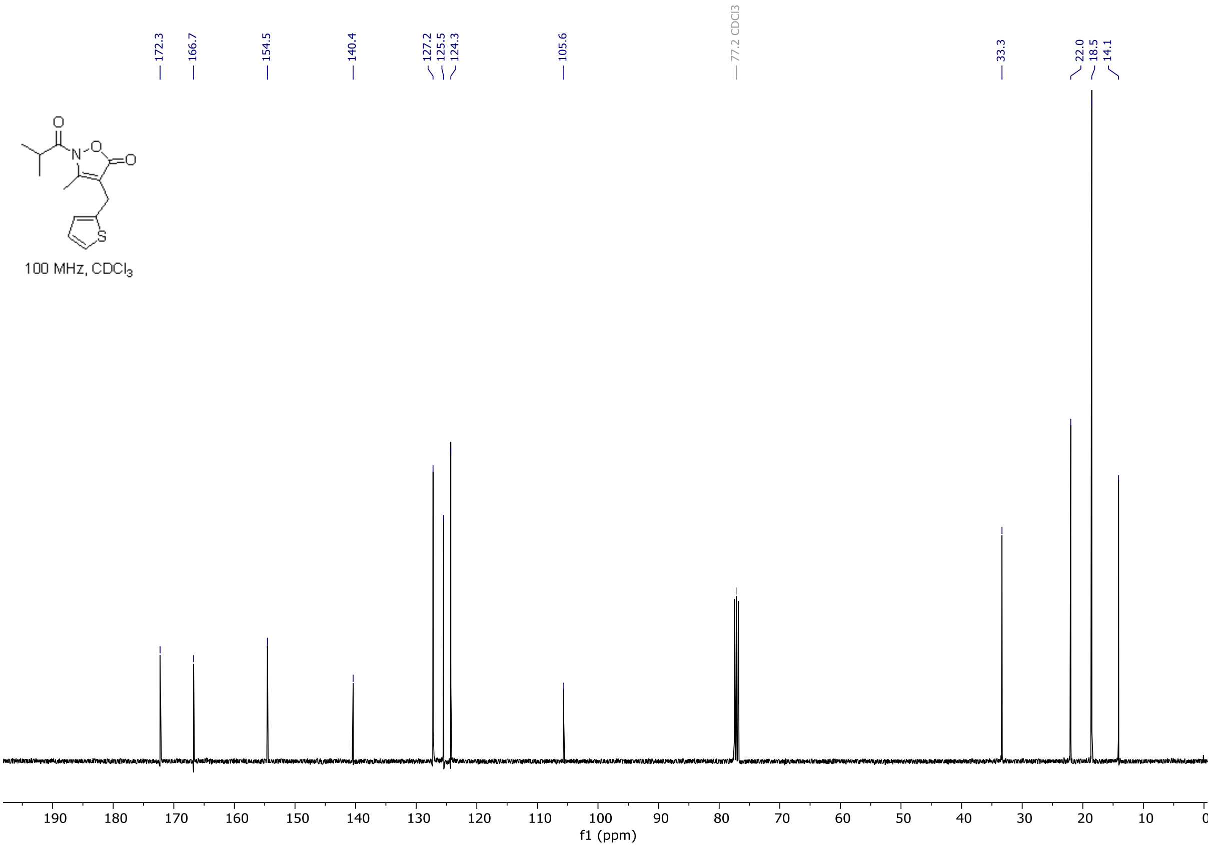

HSQC Data

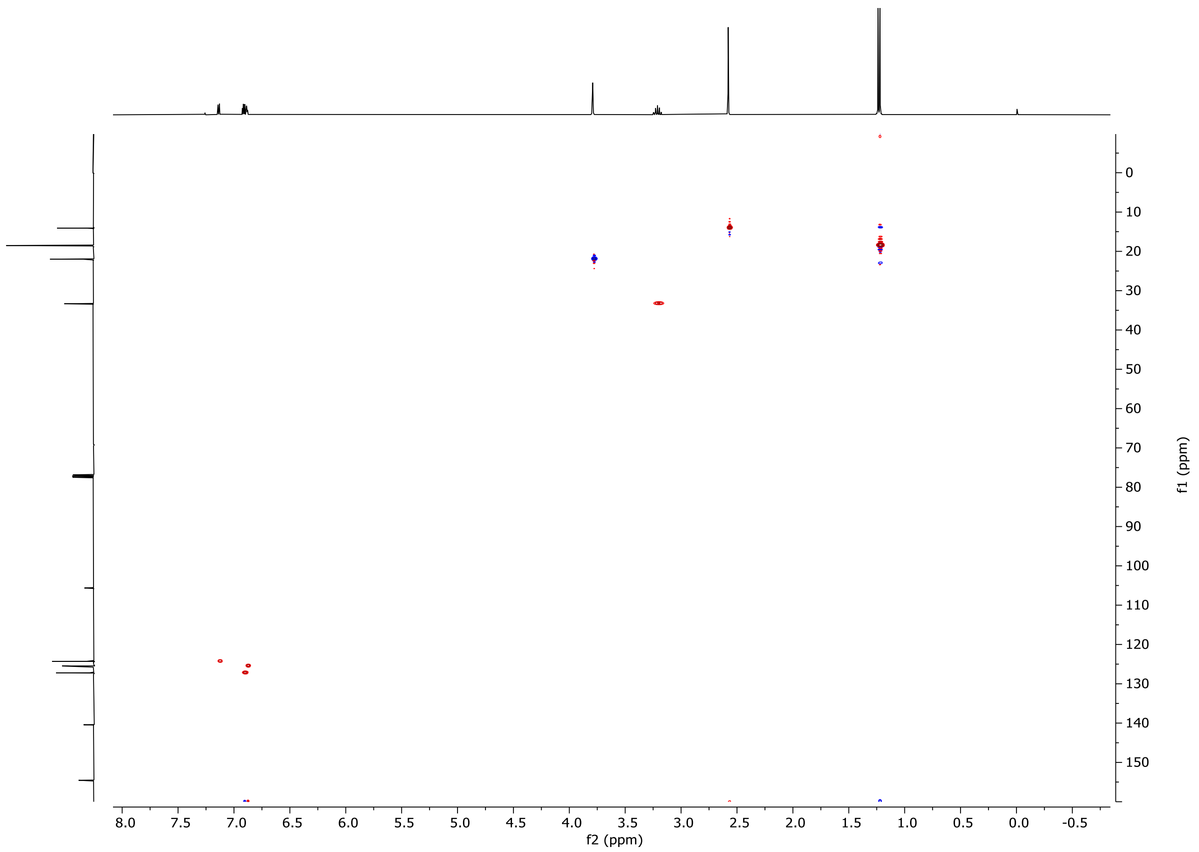

Methyl 4-(4-benzyl-3-methyl-5-oxoisoxazol-2(5H)-yl)-4-oxobutanoate (1t)

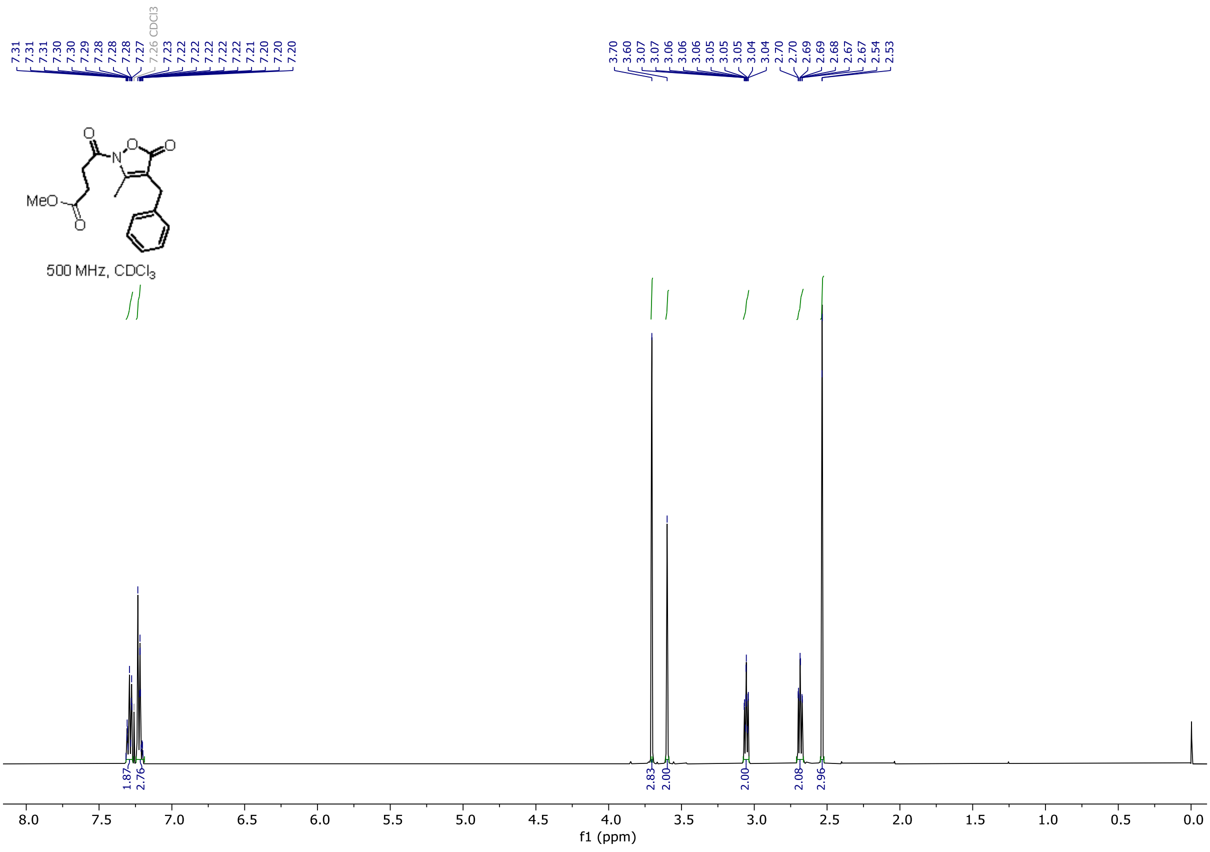

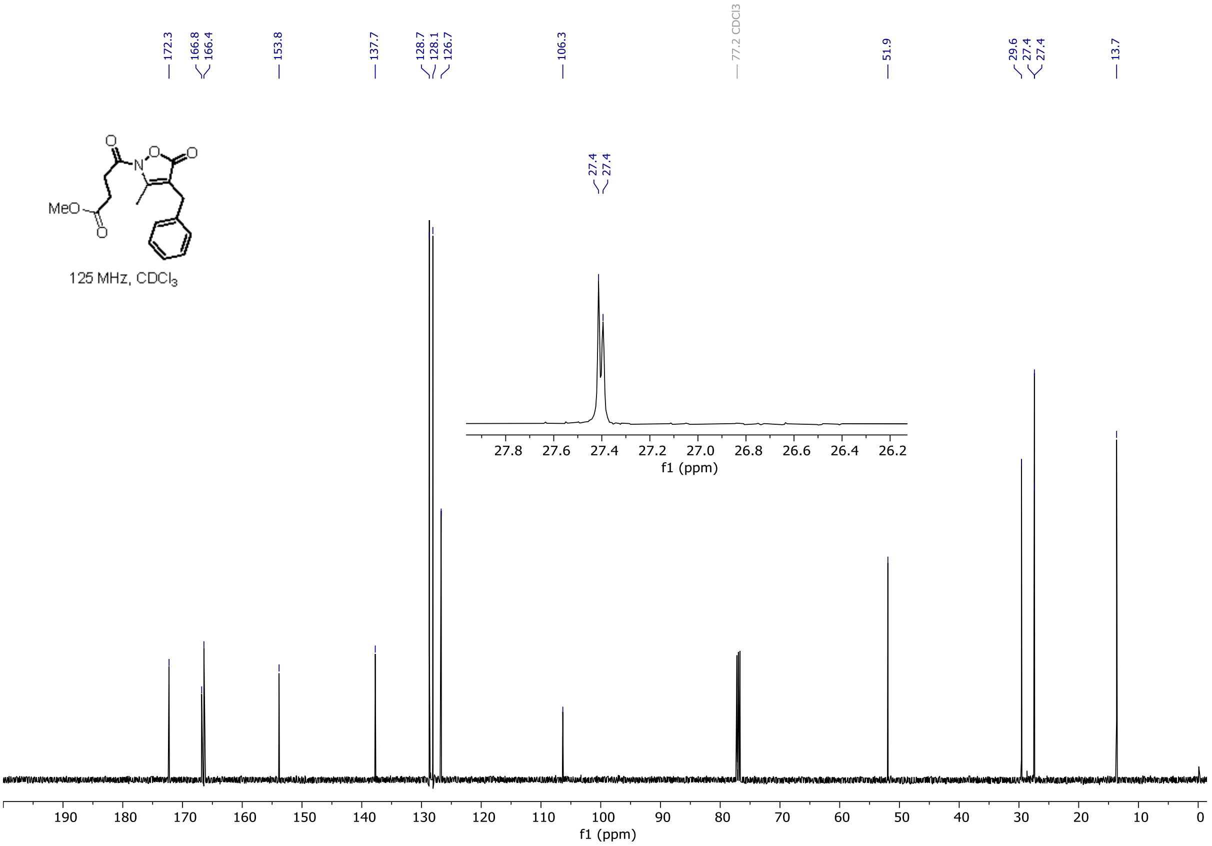
172.3
166.8
166.4
153.8
137.7
128.7
128.1
126.7
106.3
77.2 CDCl3
51.9
29.6
27.4
27.4
13.7
MeO
125 MHz, CDCl$_3$
27.4
27.4
f1 (ppm)
f1 (ppm)

HSQC Data

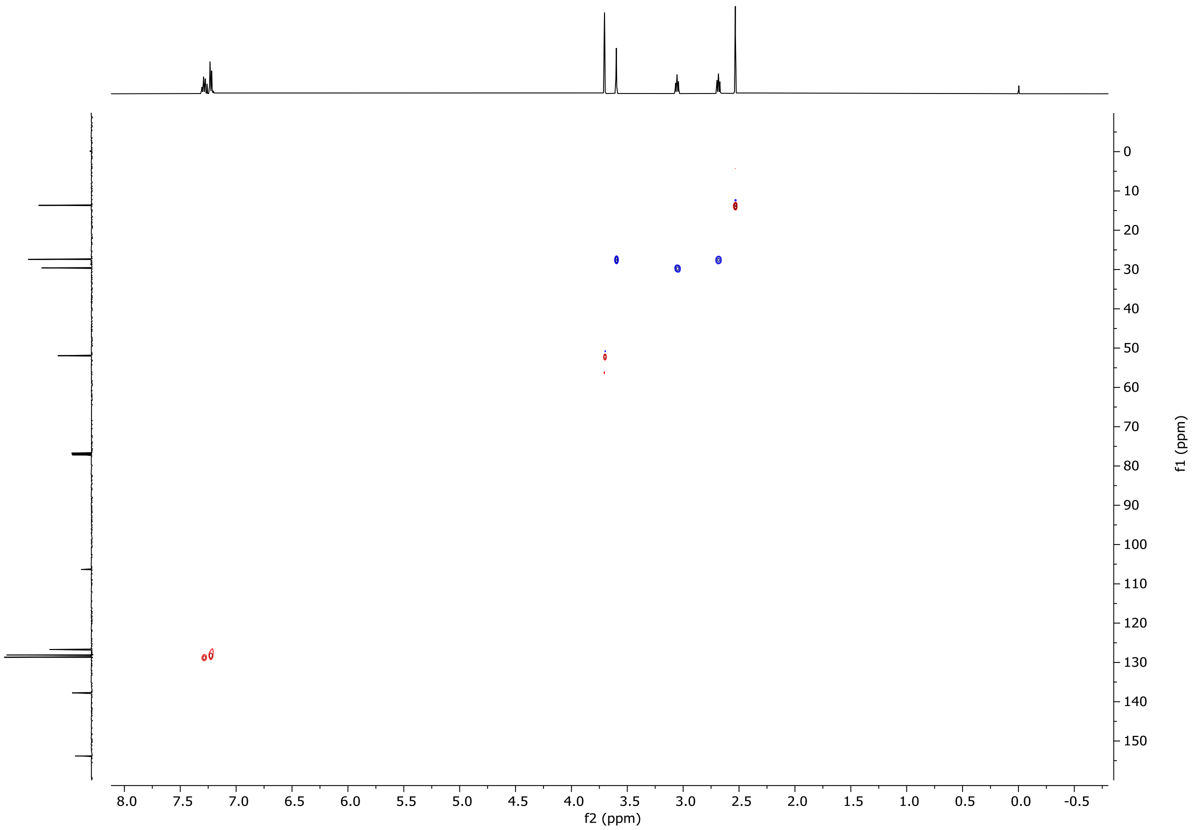
f2 (ppm)
f1 (ppm)

5-Benzyl-2,4-dimethyloxazole (2a)

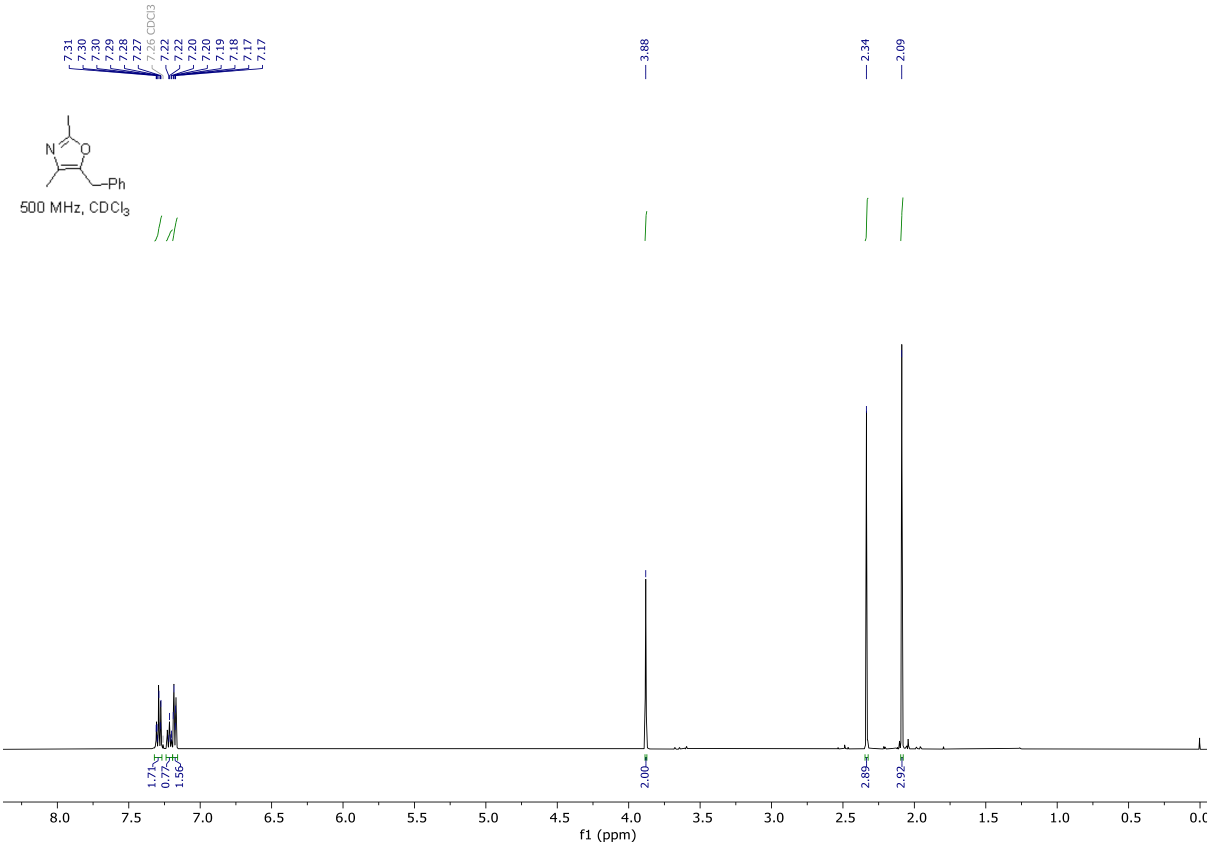

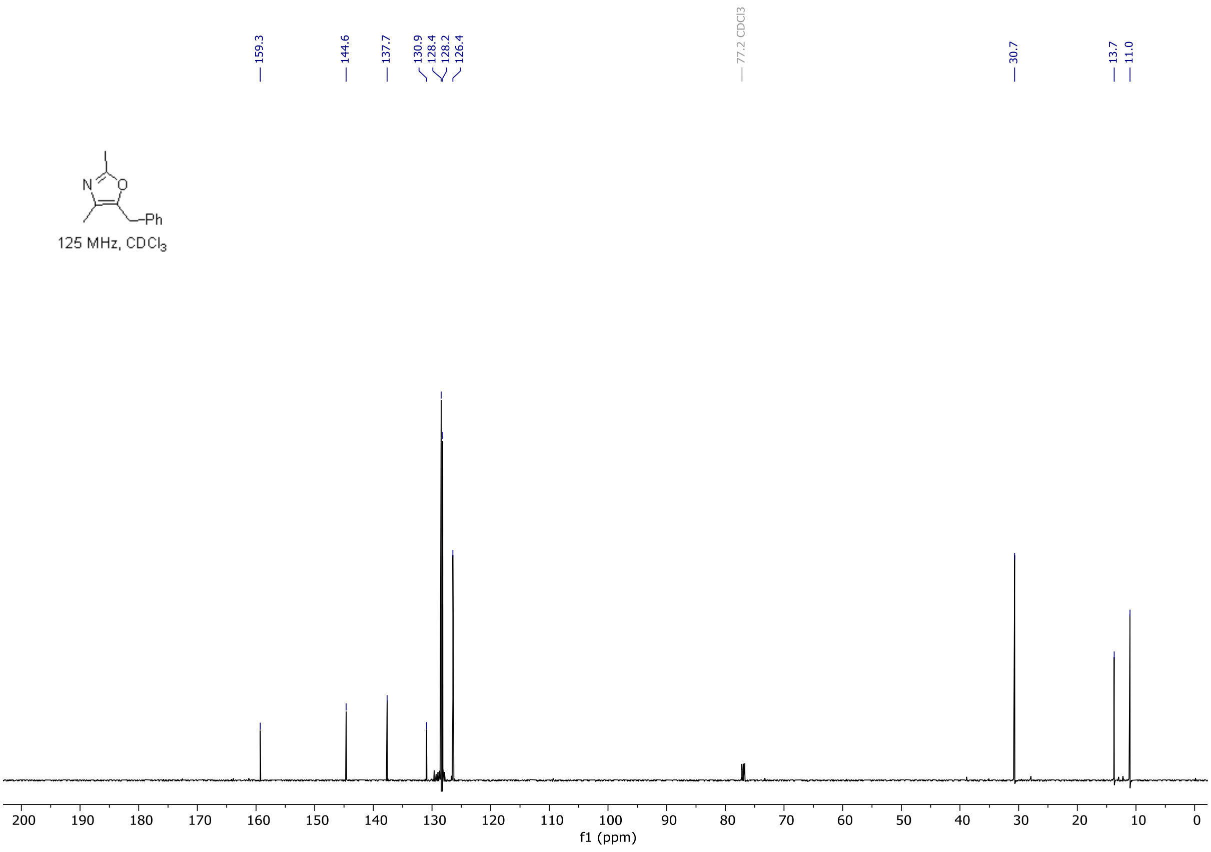

HSQC Data

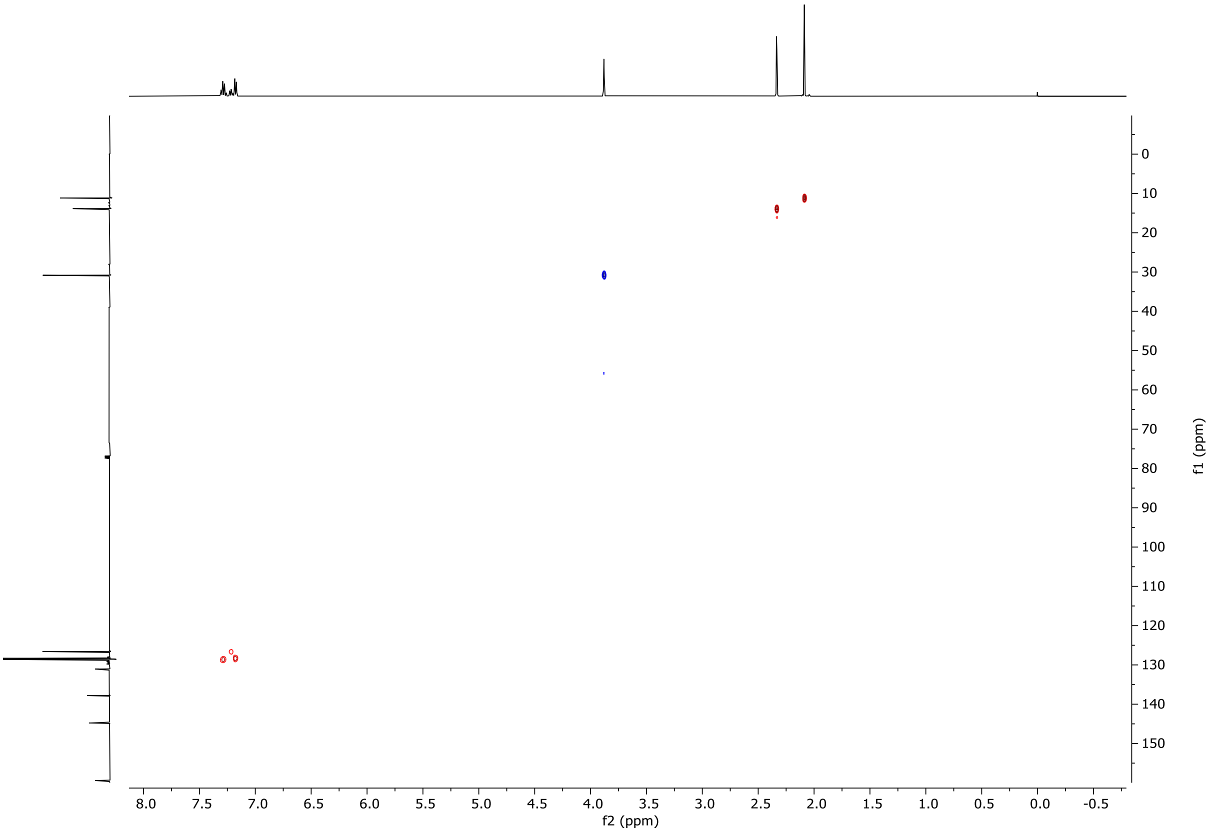

5-Benzyl-2-isopropyl-4-methyloxazole (2b)

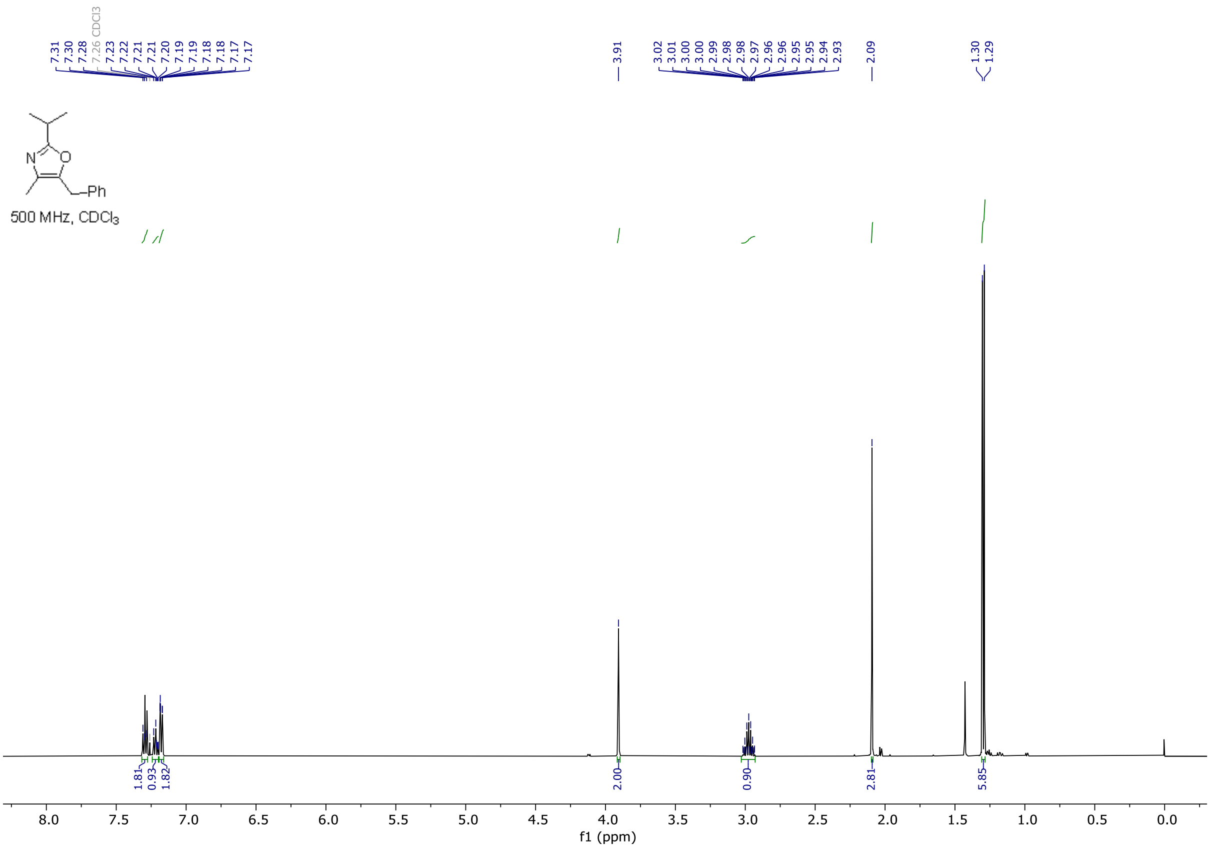

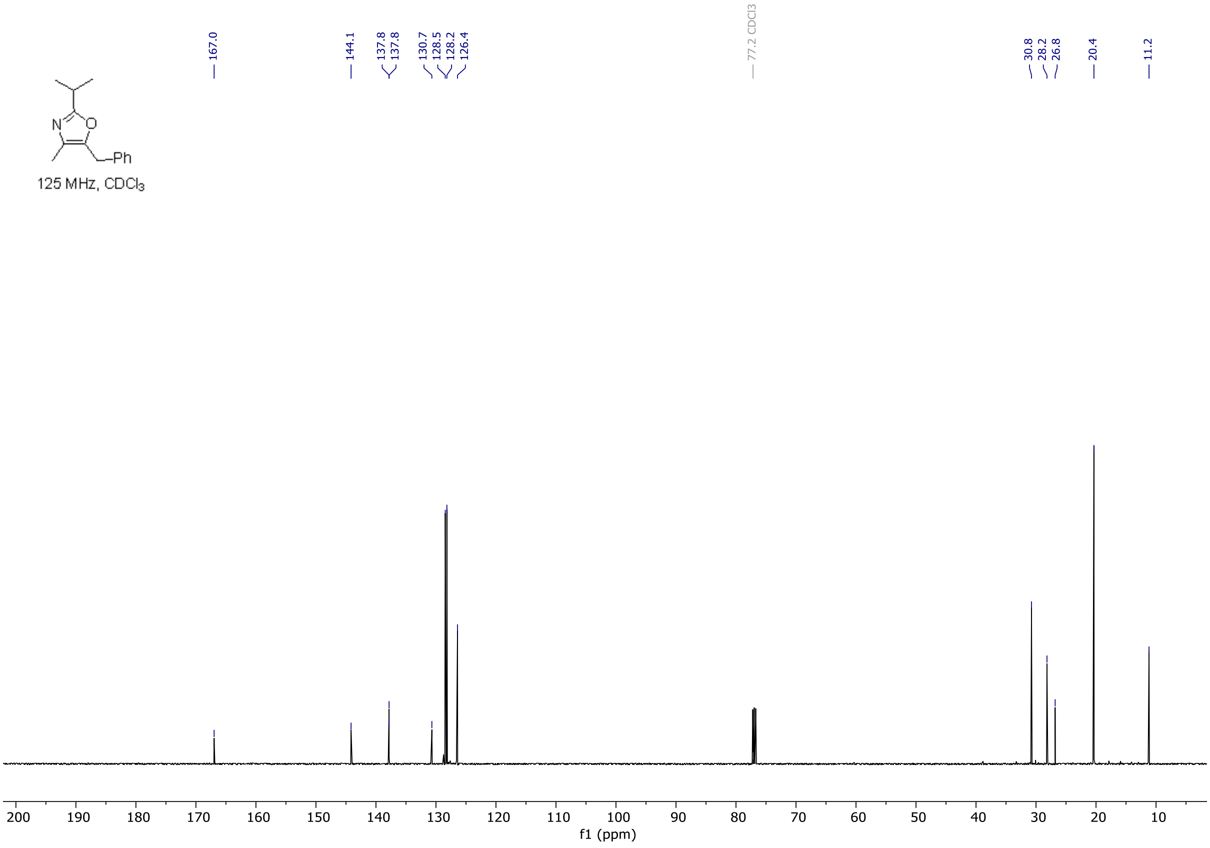
Ph
125 MHz, CDCl3
167.0
144.1
137.8
137.8
130.7
128.5
128.2
126.4
77.2 CDCl3
30.8
28.2
26.8
20.4
11.2
f1 (ppm)

HSQC Data

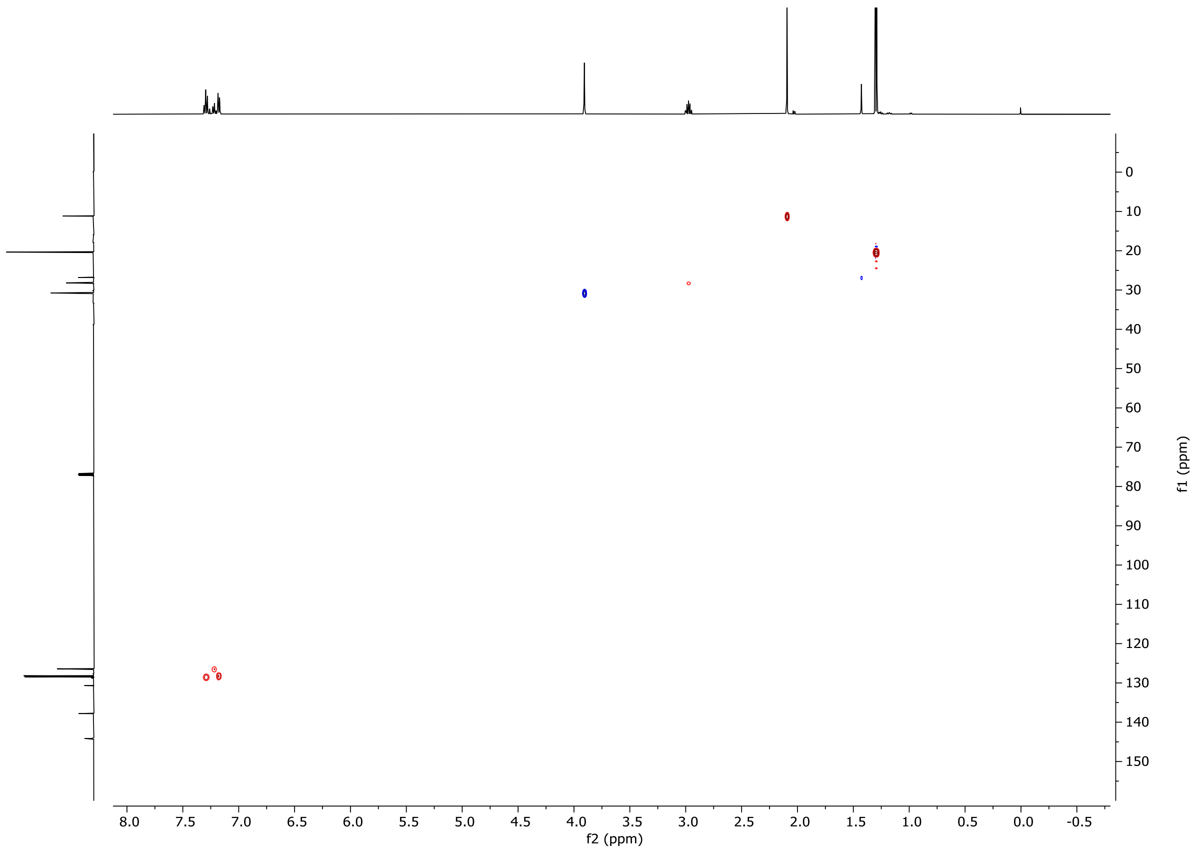
f2 (ppm)
f1 (ppm)

5-Benzyl-2-ethyl-4-methyloxazole (2c)

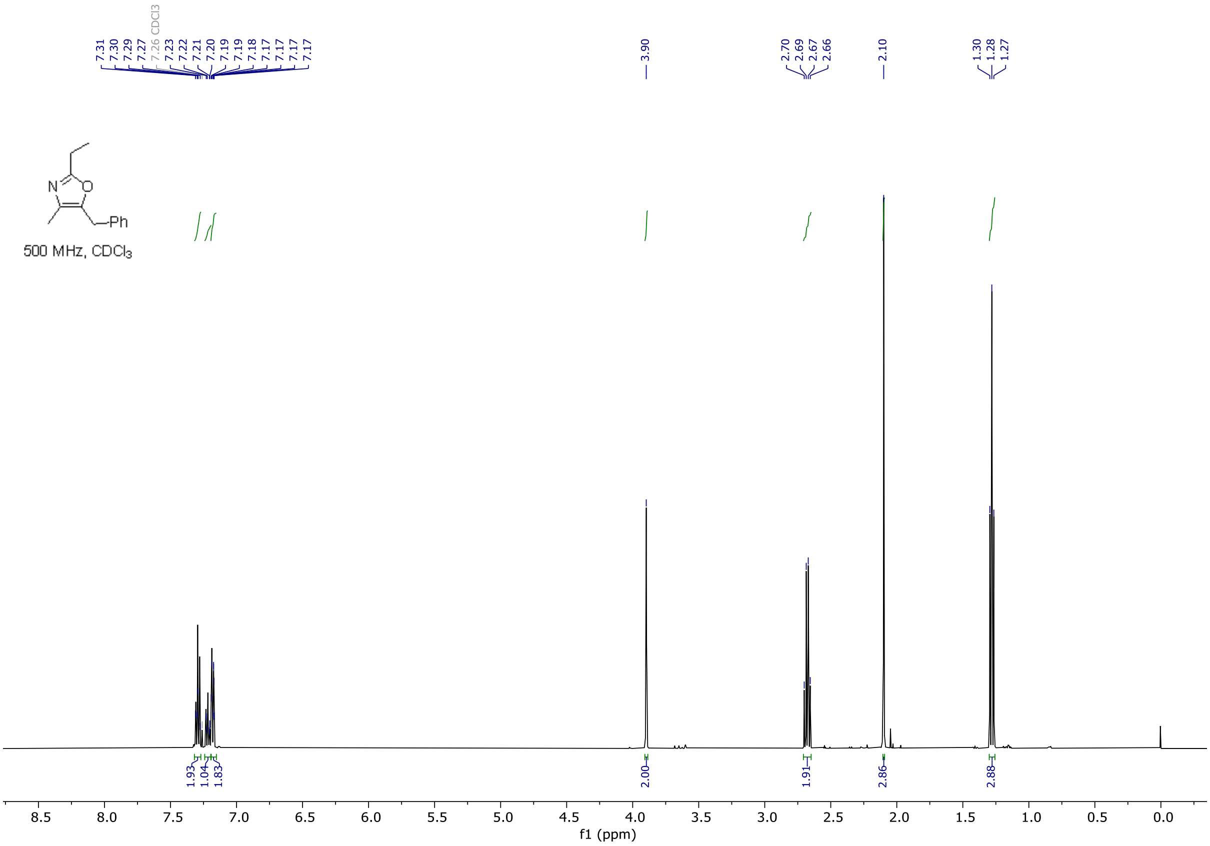

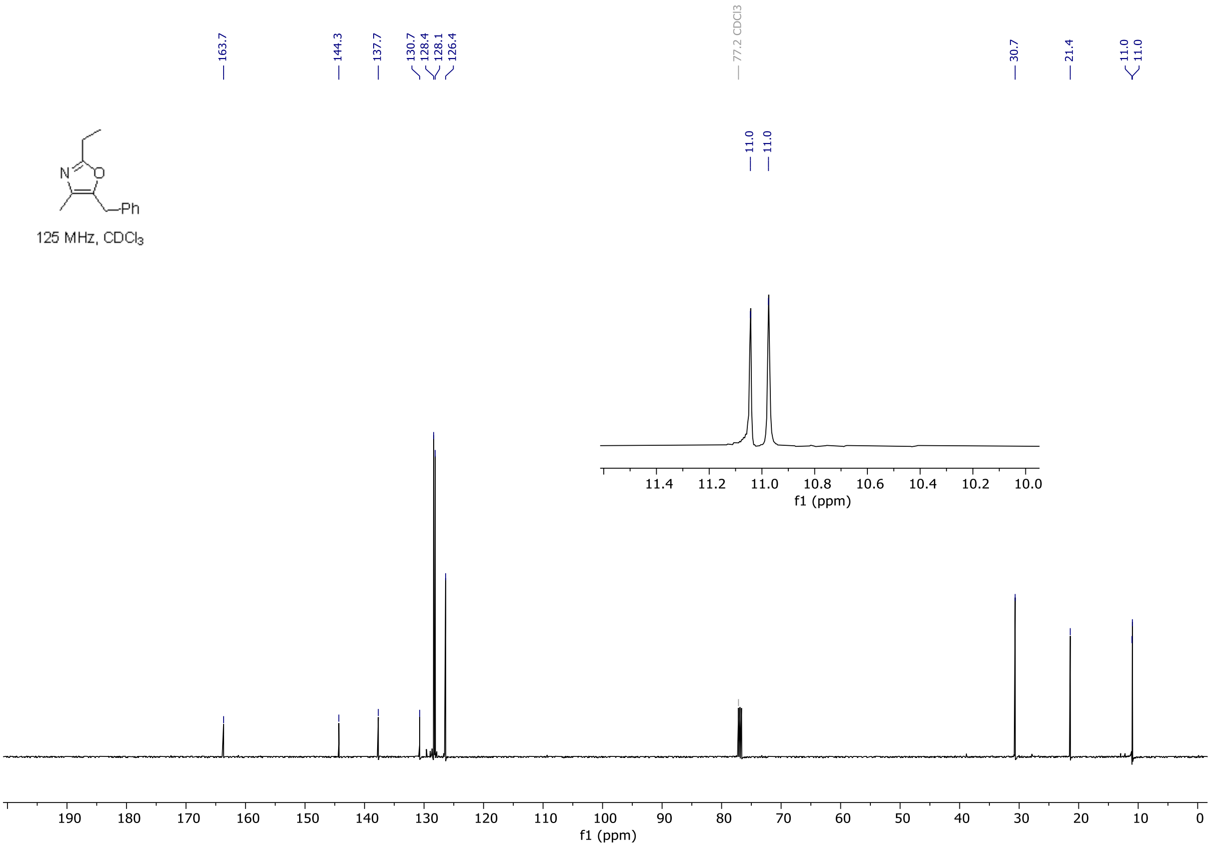

HSQC Data

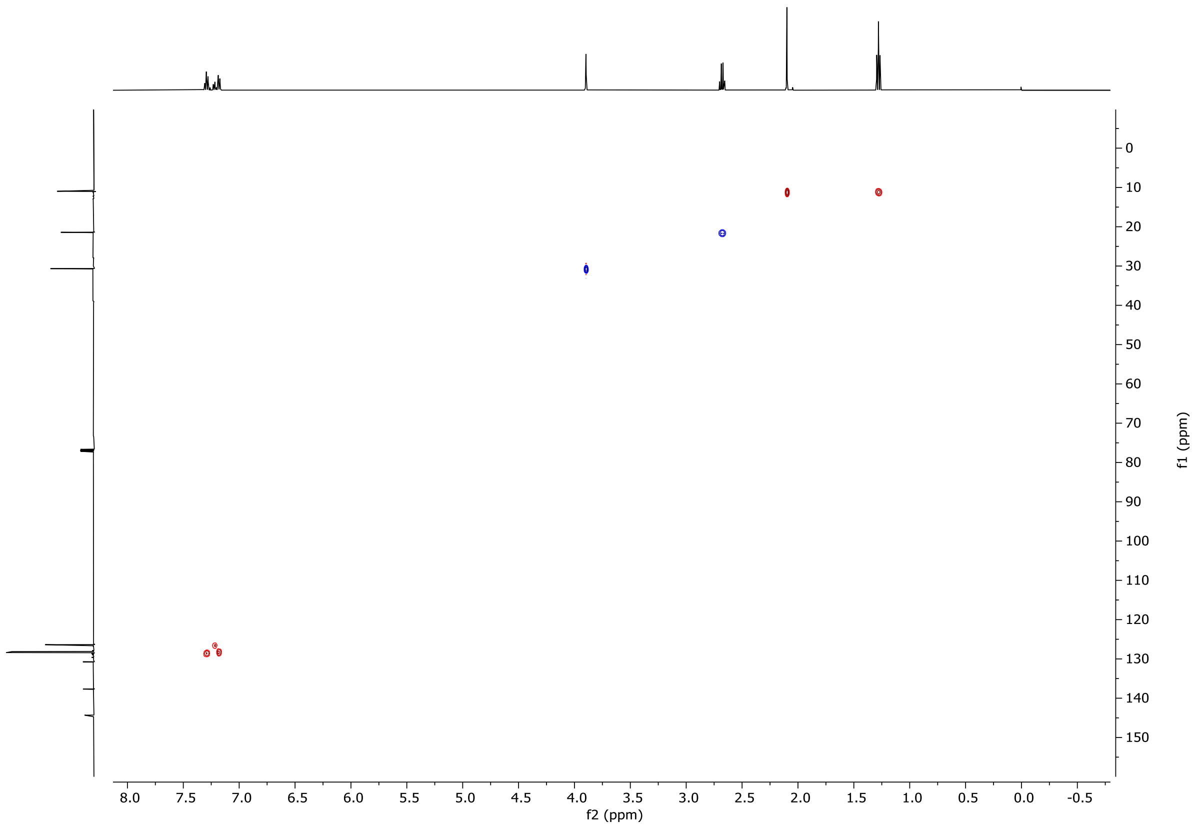

5-Benzyl-2-butyl-4-methyloxazole (2d)

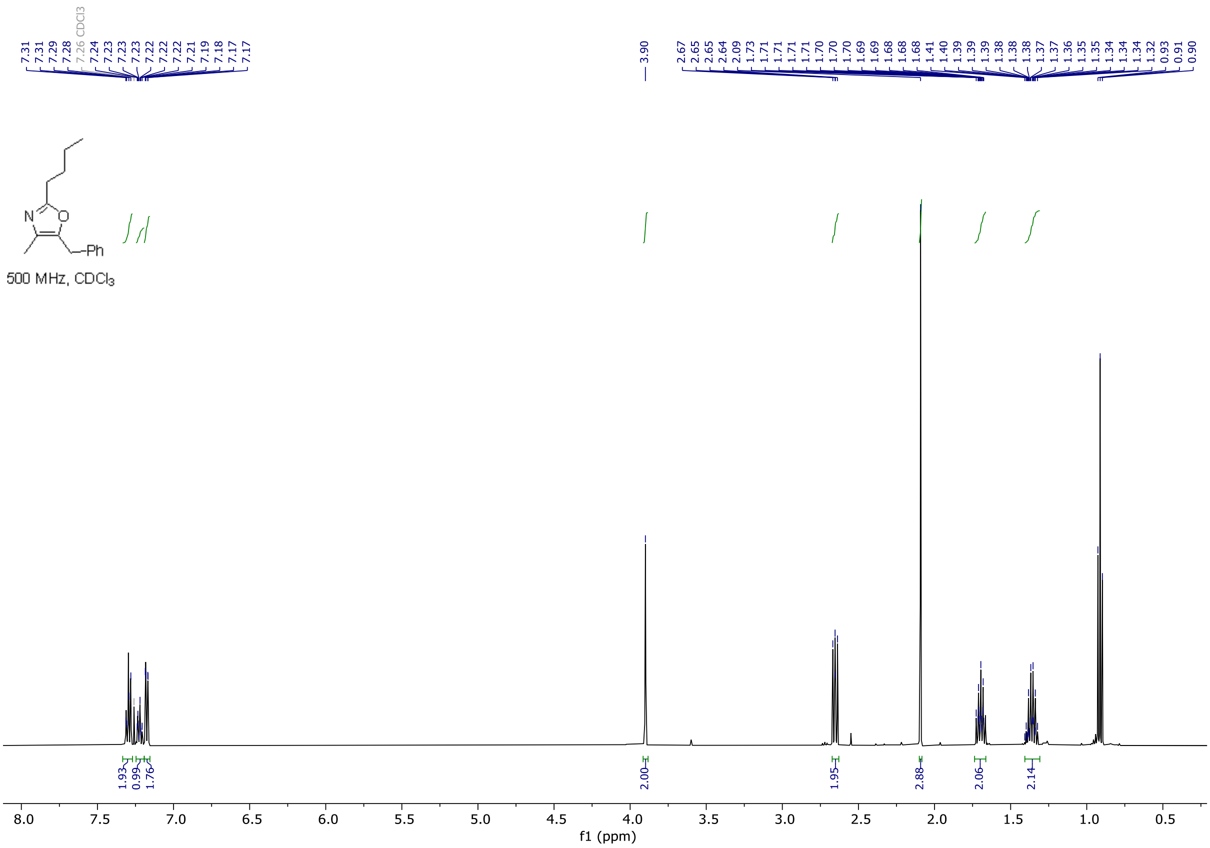

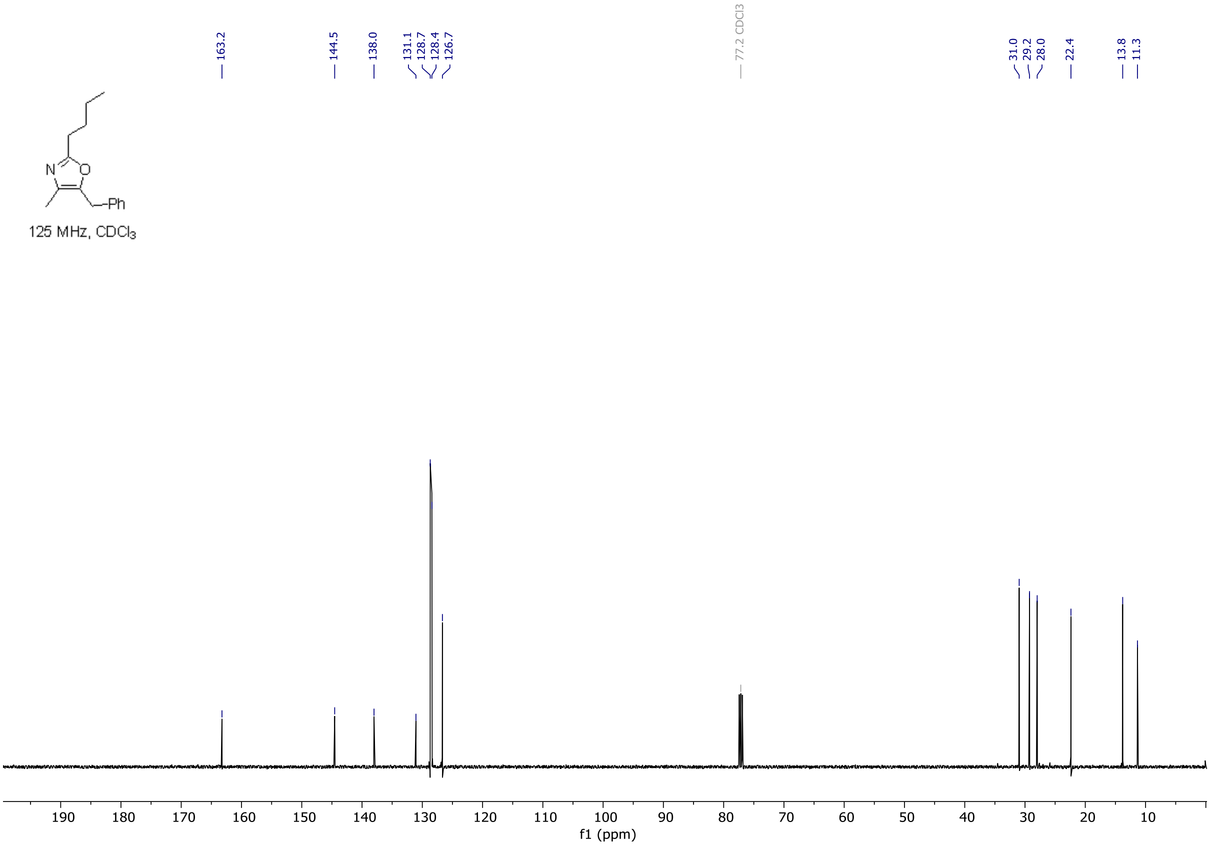

125 MHz, $CDCl_3$

HSQC Data

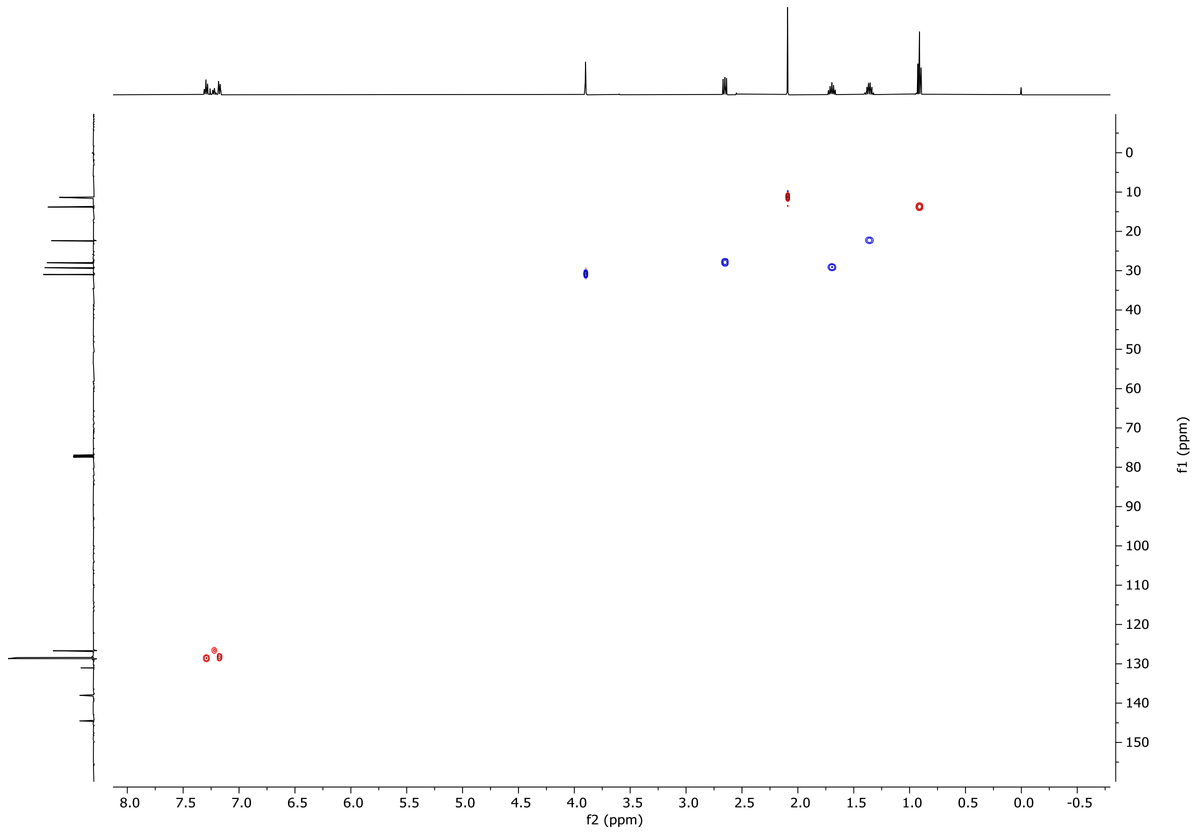

5-Benzyl-2-cyclopropyl-4-methyloxazole (2e)

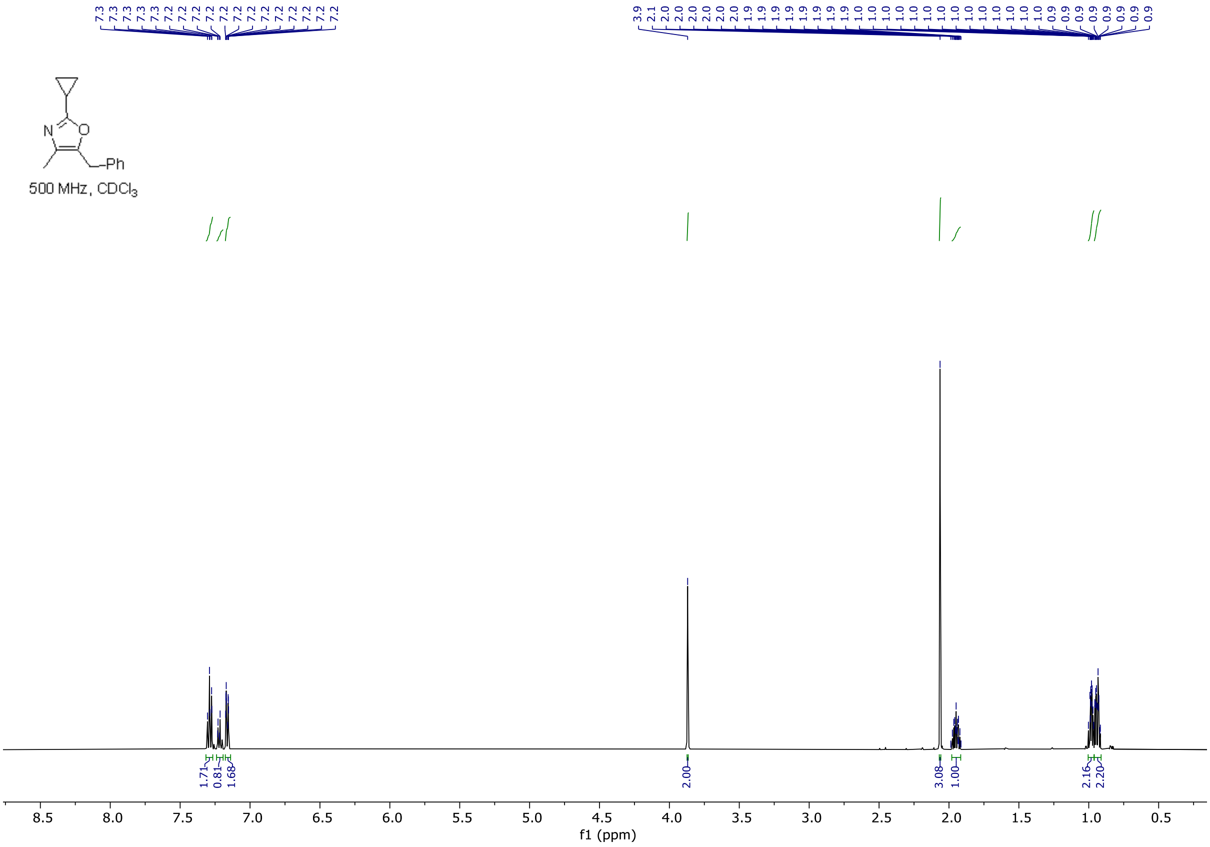

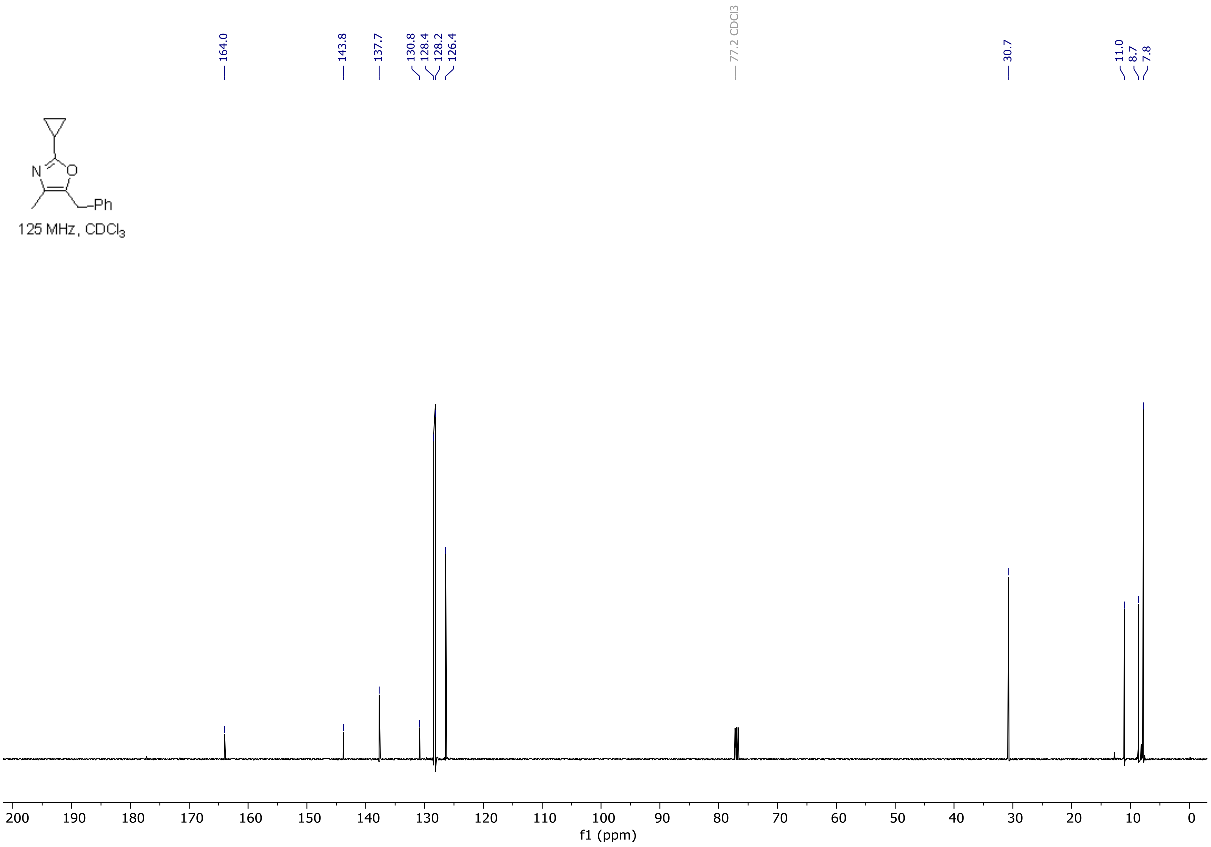

HSQC Data

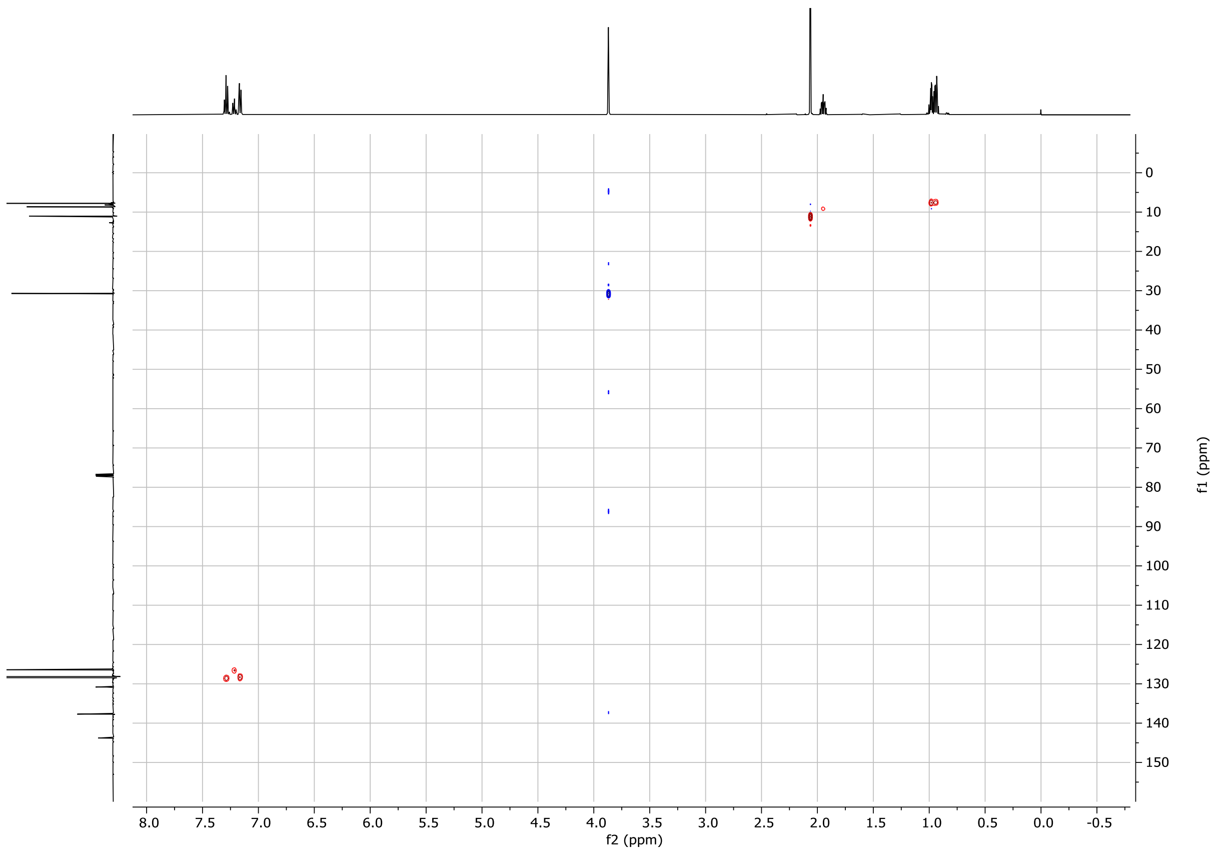

5-Benzyl-2-cyclobutyl-4-methyloxazole (2f)

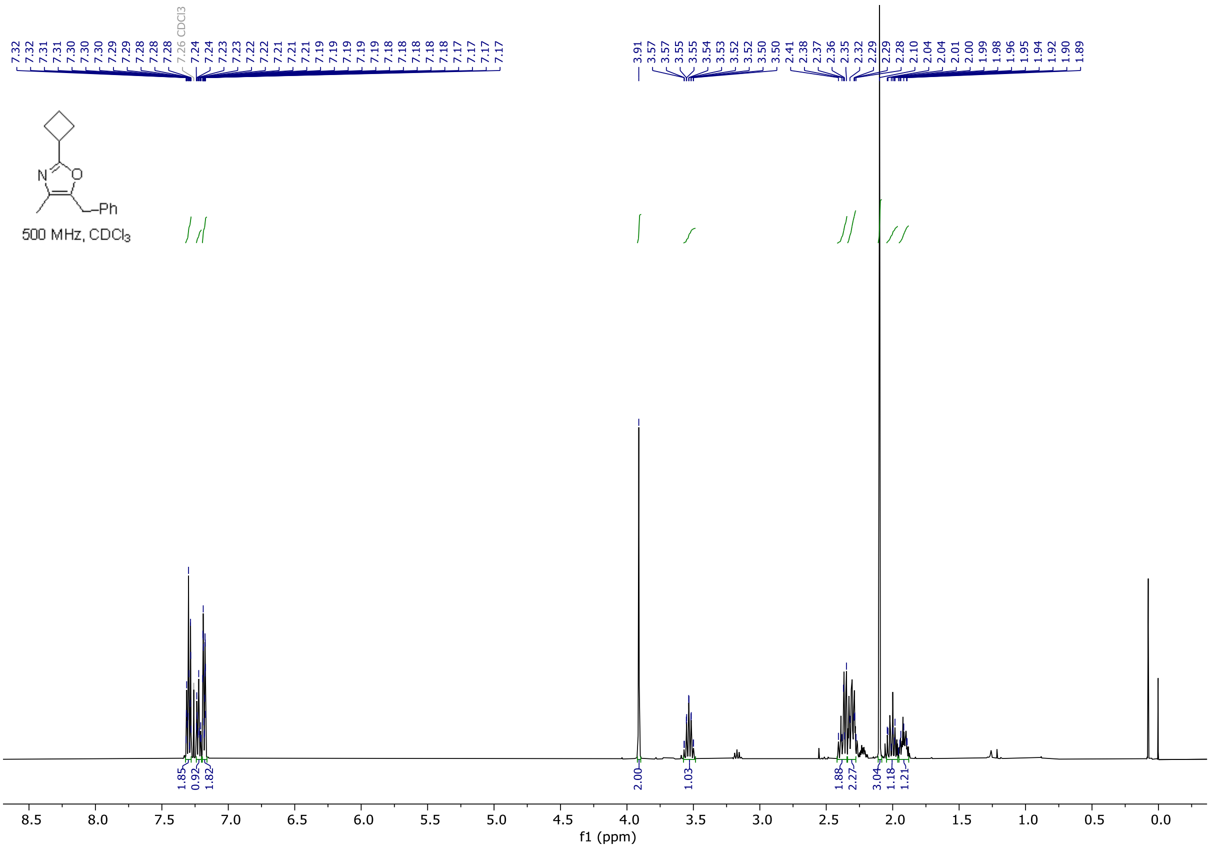

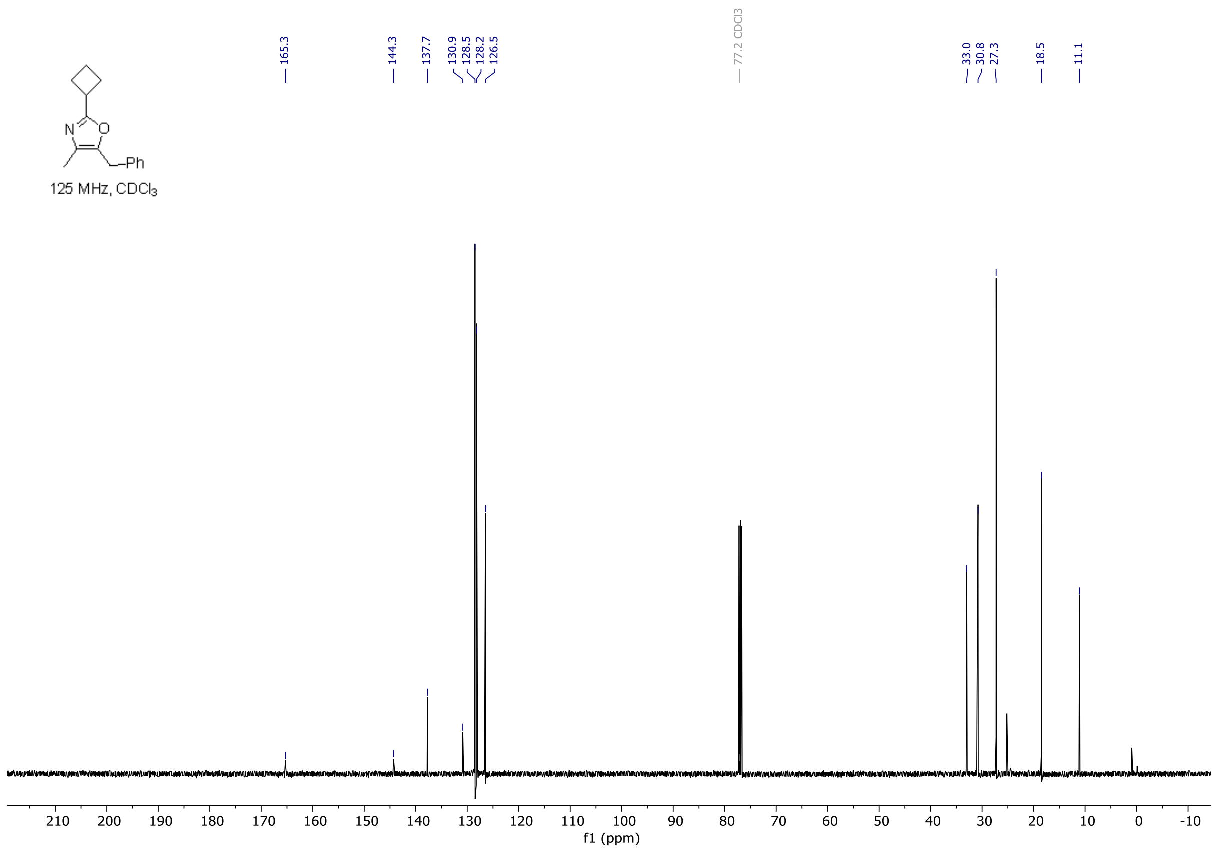
165.3
144.3
137.7
130.9
128.5
128.2
126.5
77.2 CDCl3
33.0
30.8
27.3
18.5
11.1
N
O
Ph
125 MHz, CDCl3
f1 (ppm)

HSQC Data

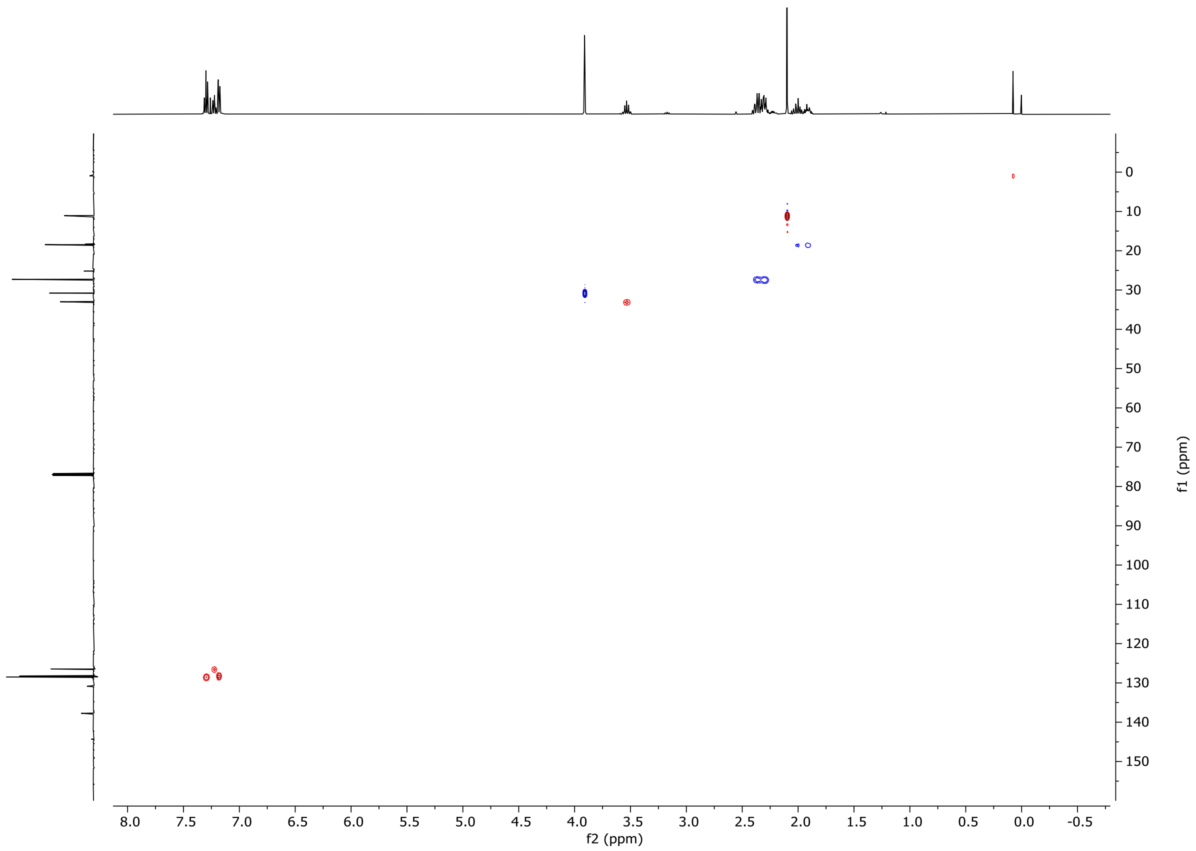
f2 (ppm)
f1 (ppm)

5-Benzyl-2-cyclohexyl-4-methyloxazole (2g)

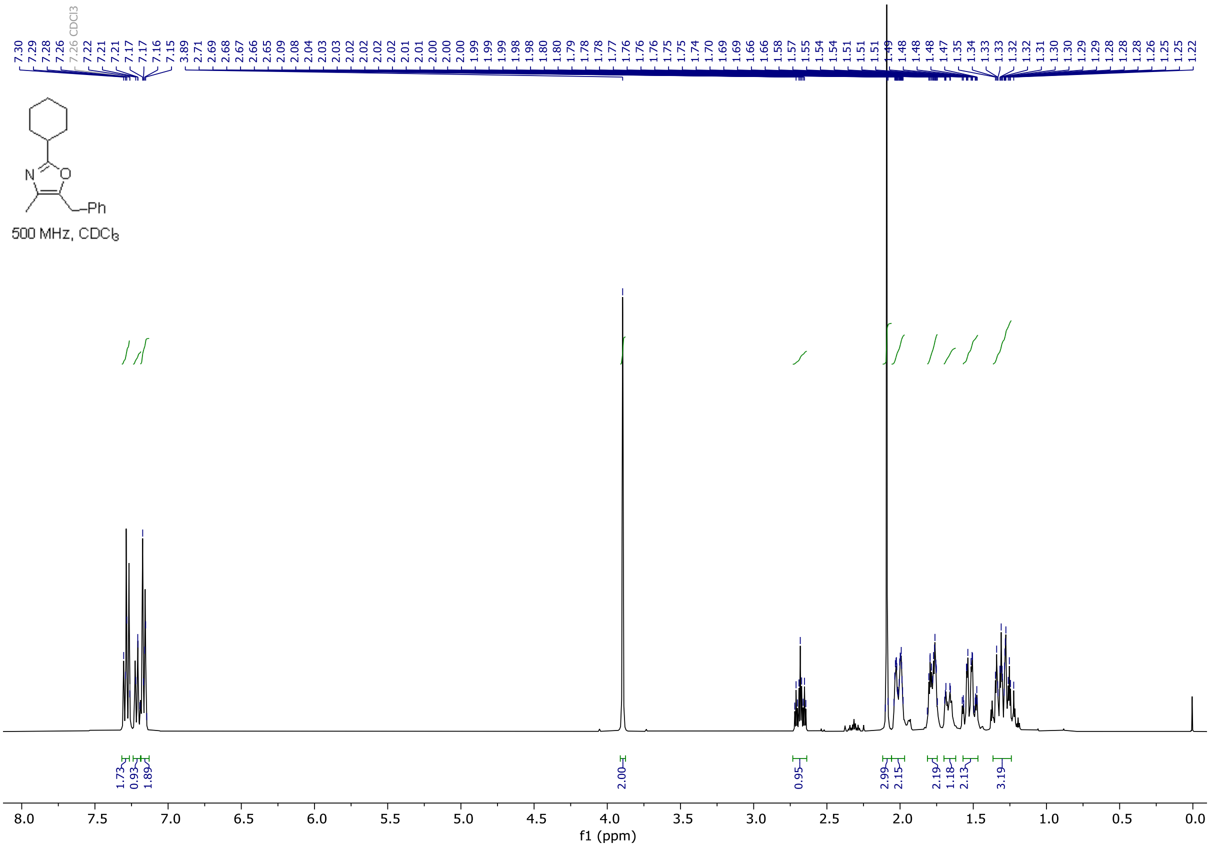

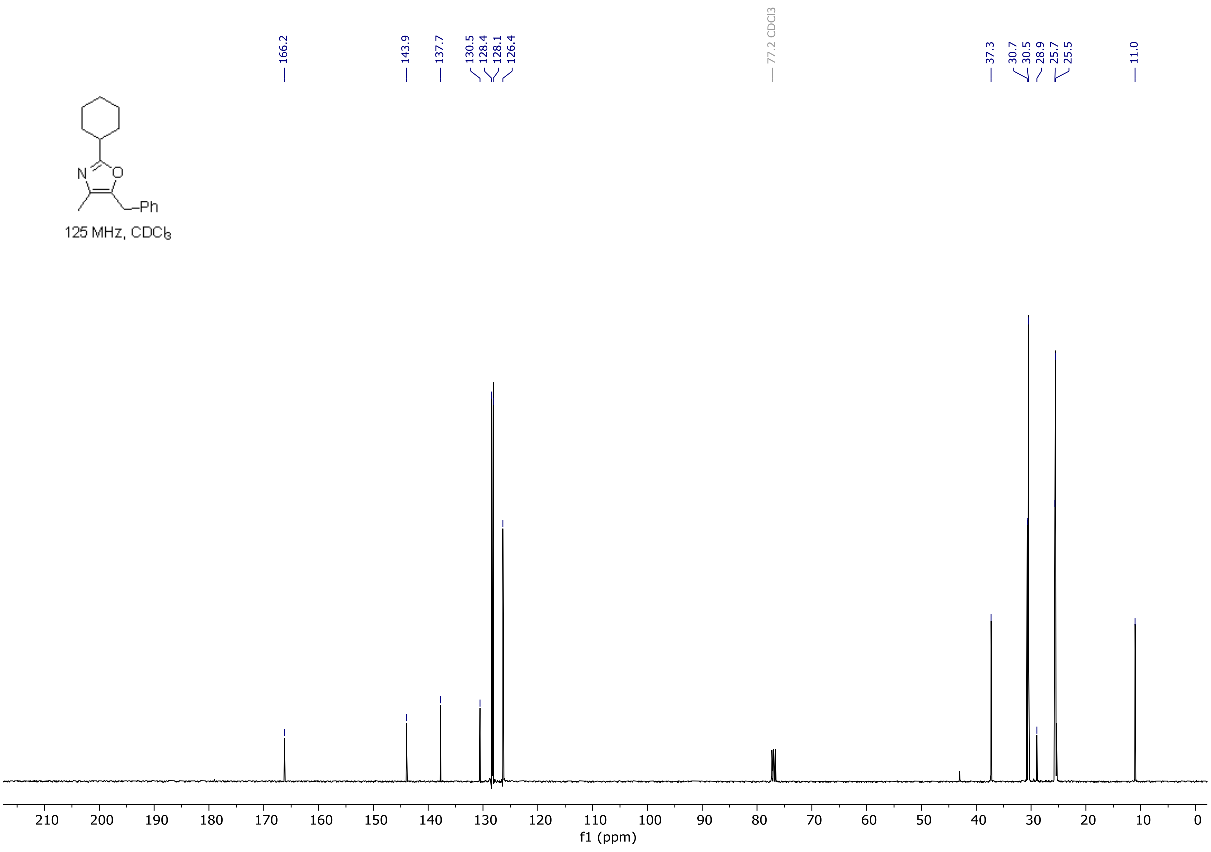

HSQC Data

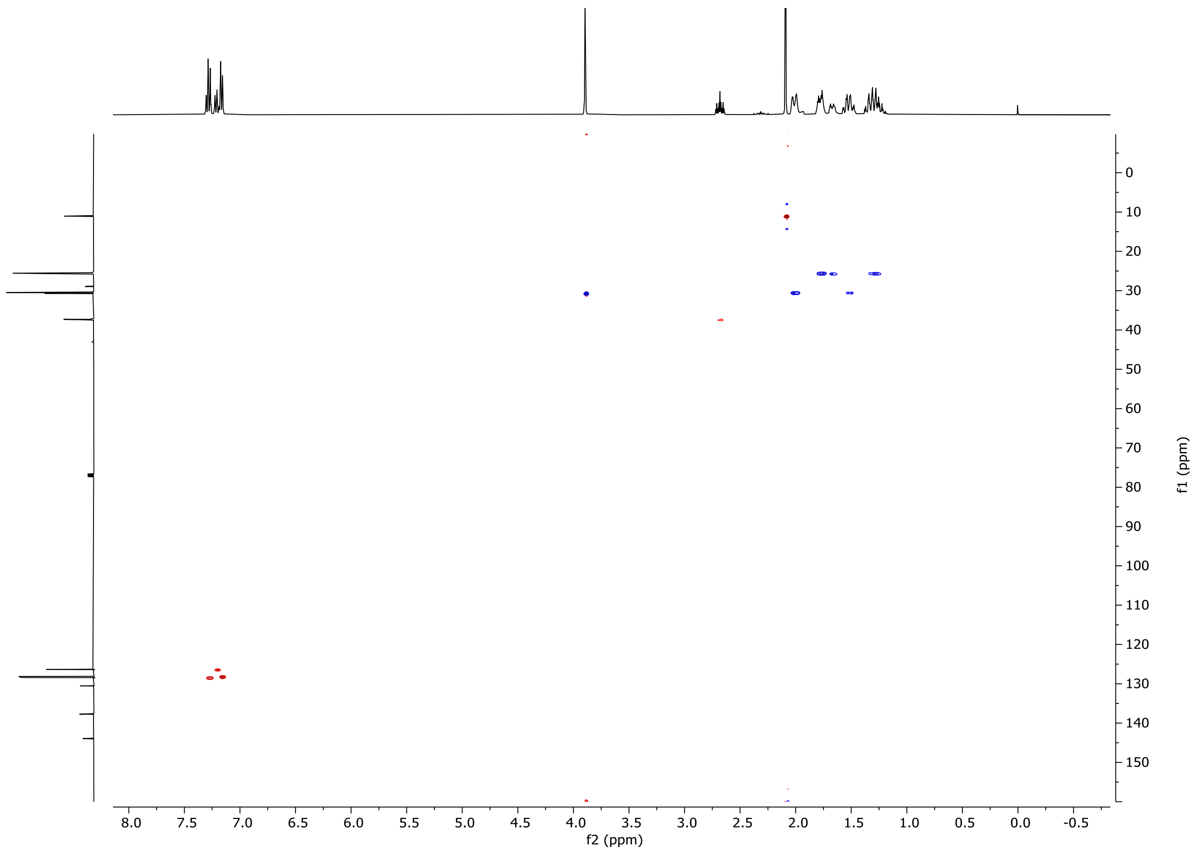

5-Benzyl-4-methyl-2-(tetrahydro-2H-pyran-4-yl)oxazole (2h)

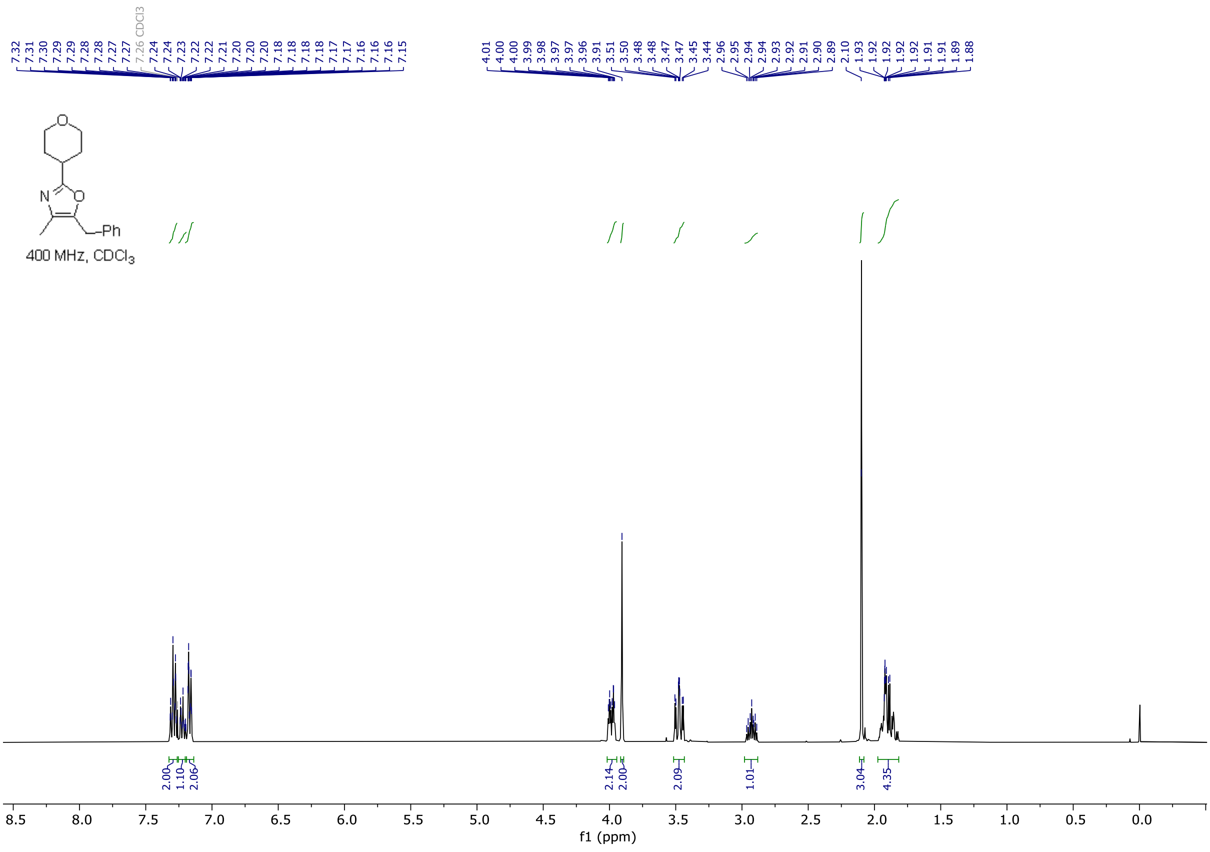

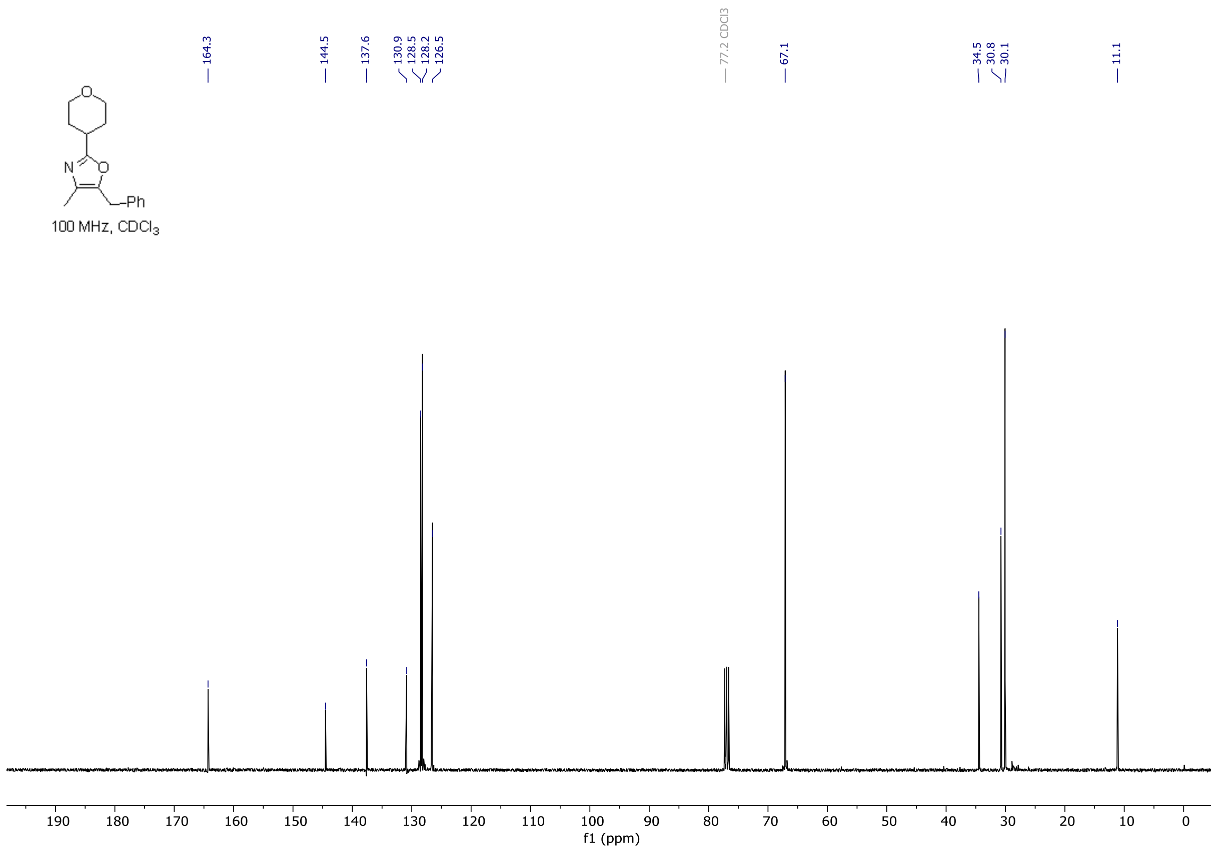

164.3
144.5
137.6
130.9
128.5
128.2
126.5
77.2 CDCl3
67.1
34.5
30.8
30.1
11.1
N
O
O
Ph
100 MHz, CDCl3
f1 (ppm)

HSQC Data

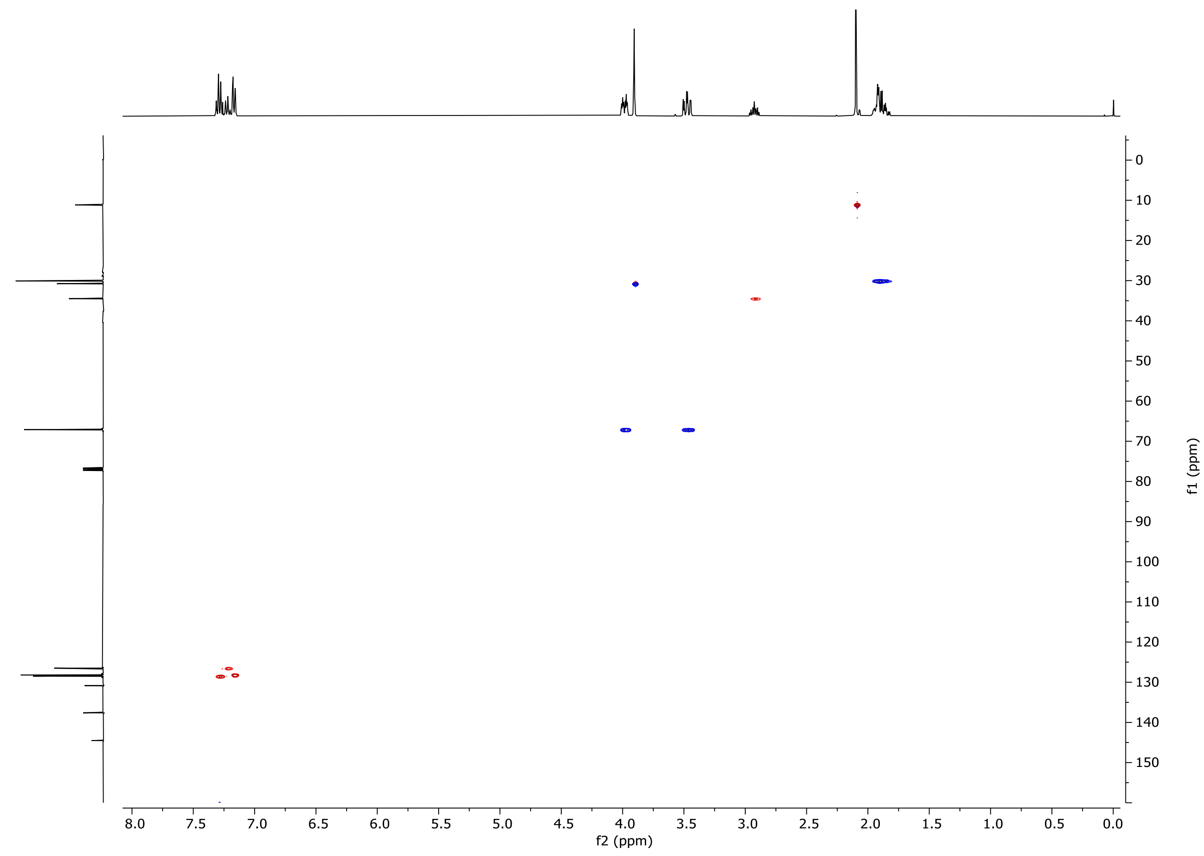

f2 (ppm)
f1 (ppm)

Methyl 4-(5-Benzyl-4-methyloxazol-2-yl)bicyclo[2.2.2]octane-1-carboxylate (2i)

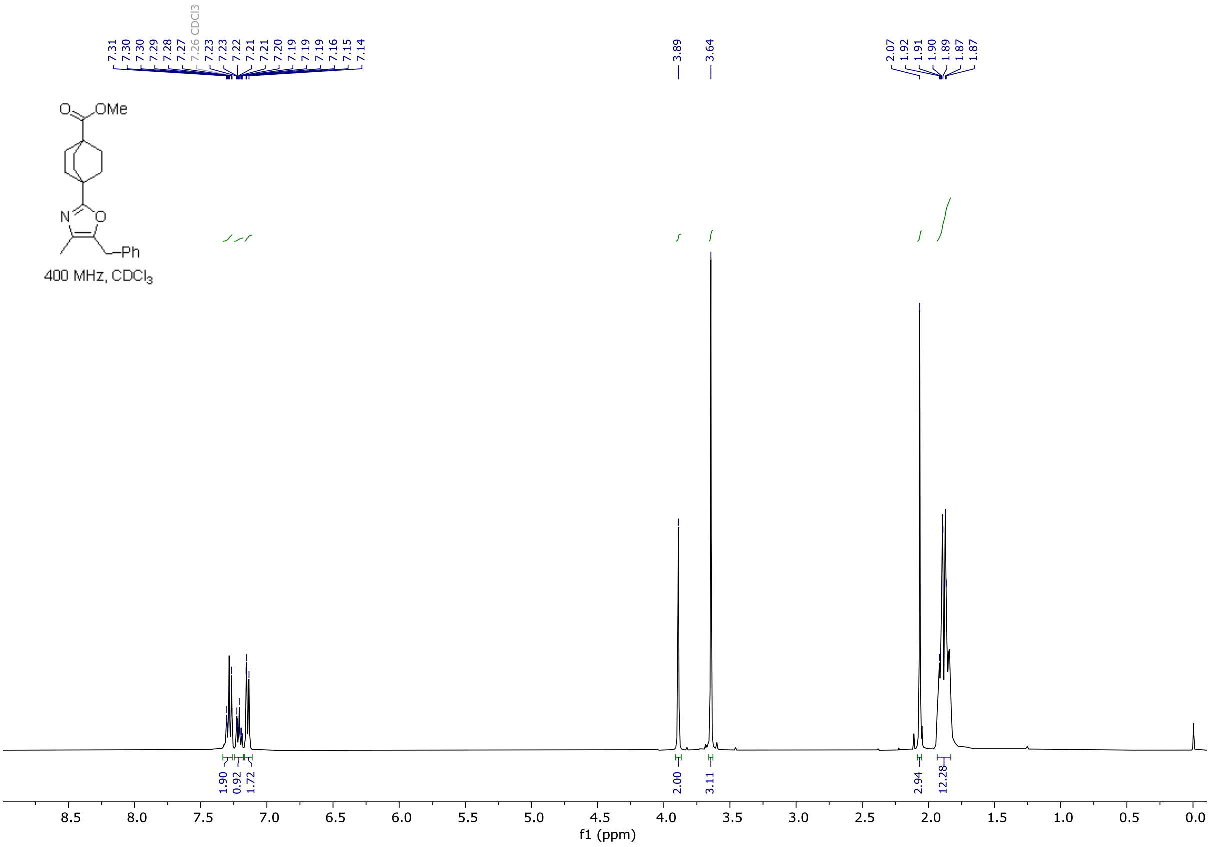

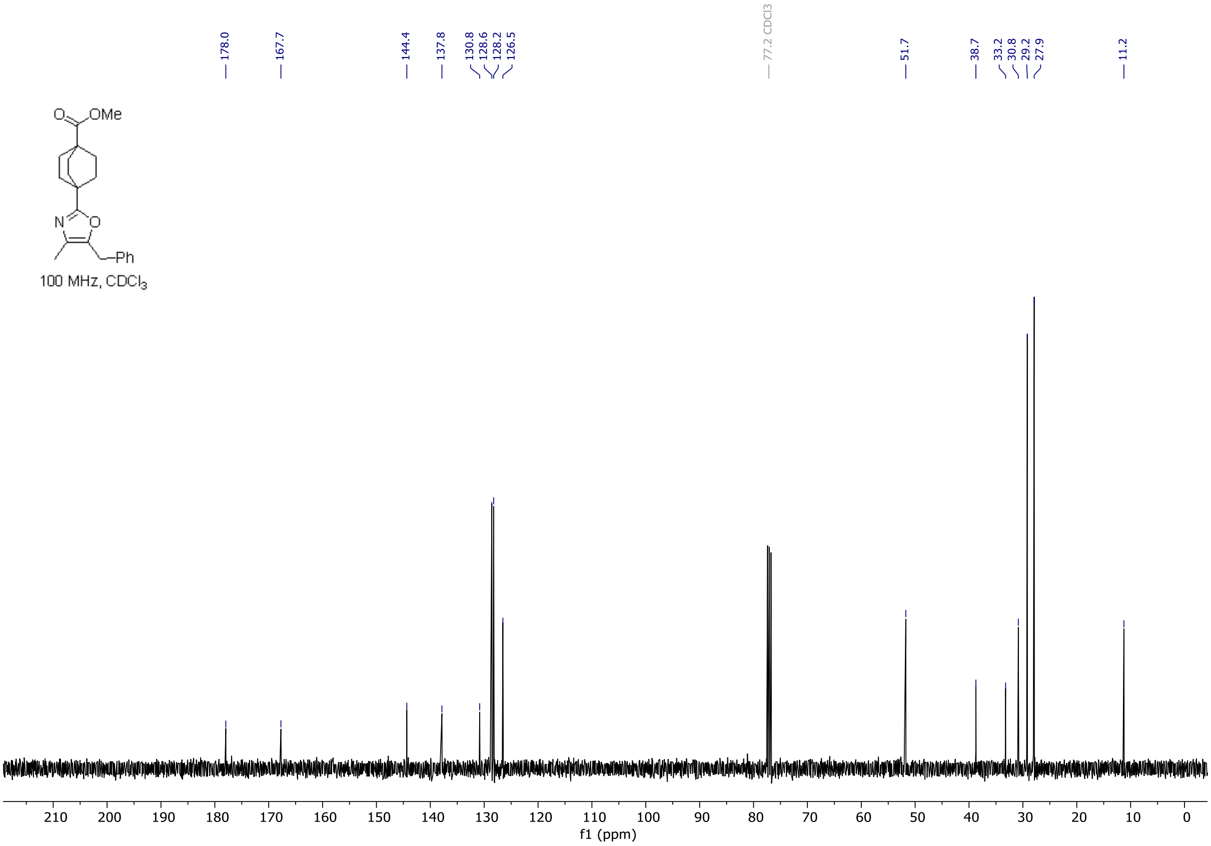

HSQC Data

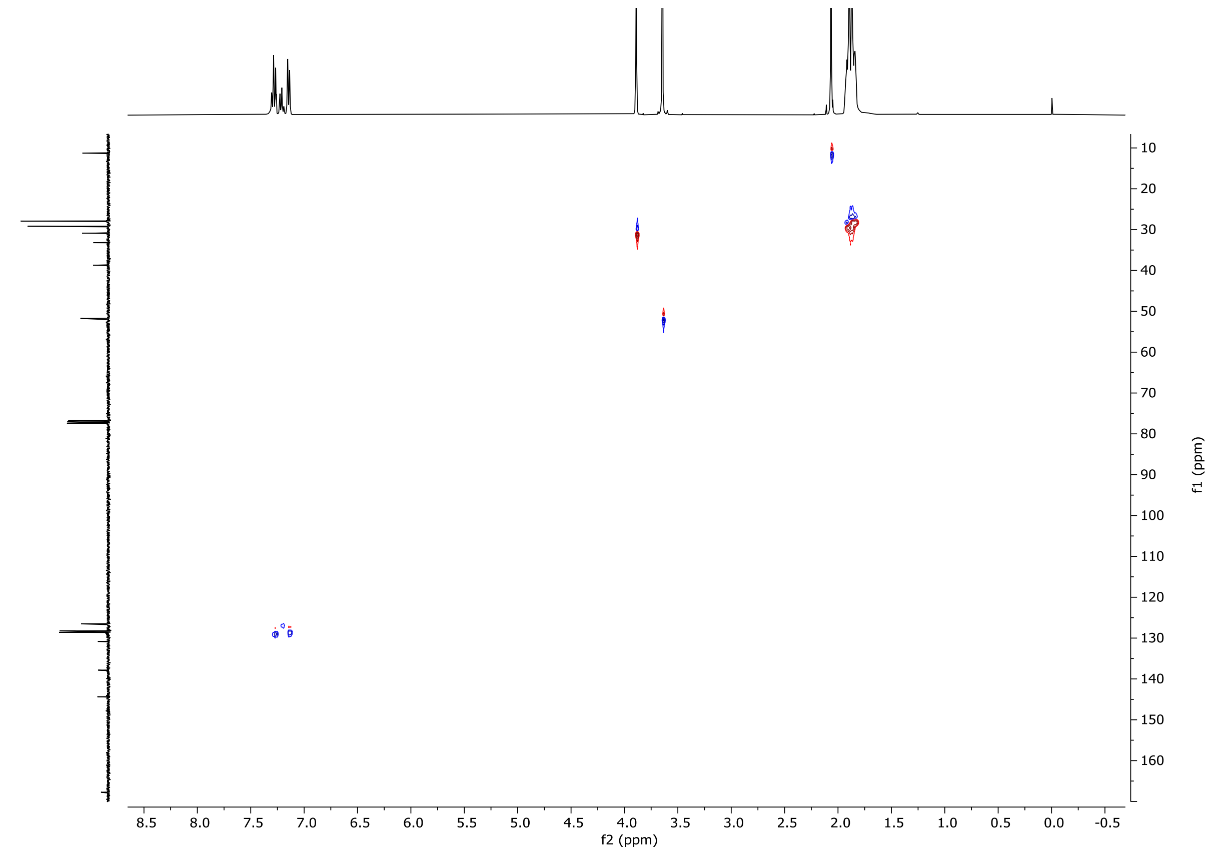

3-(5-Benzyl-4-methyloxazol-2-yl)cyclobutan-1-one (2j)

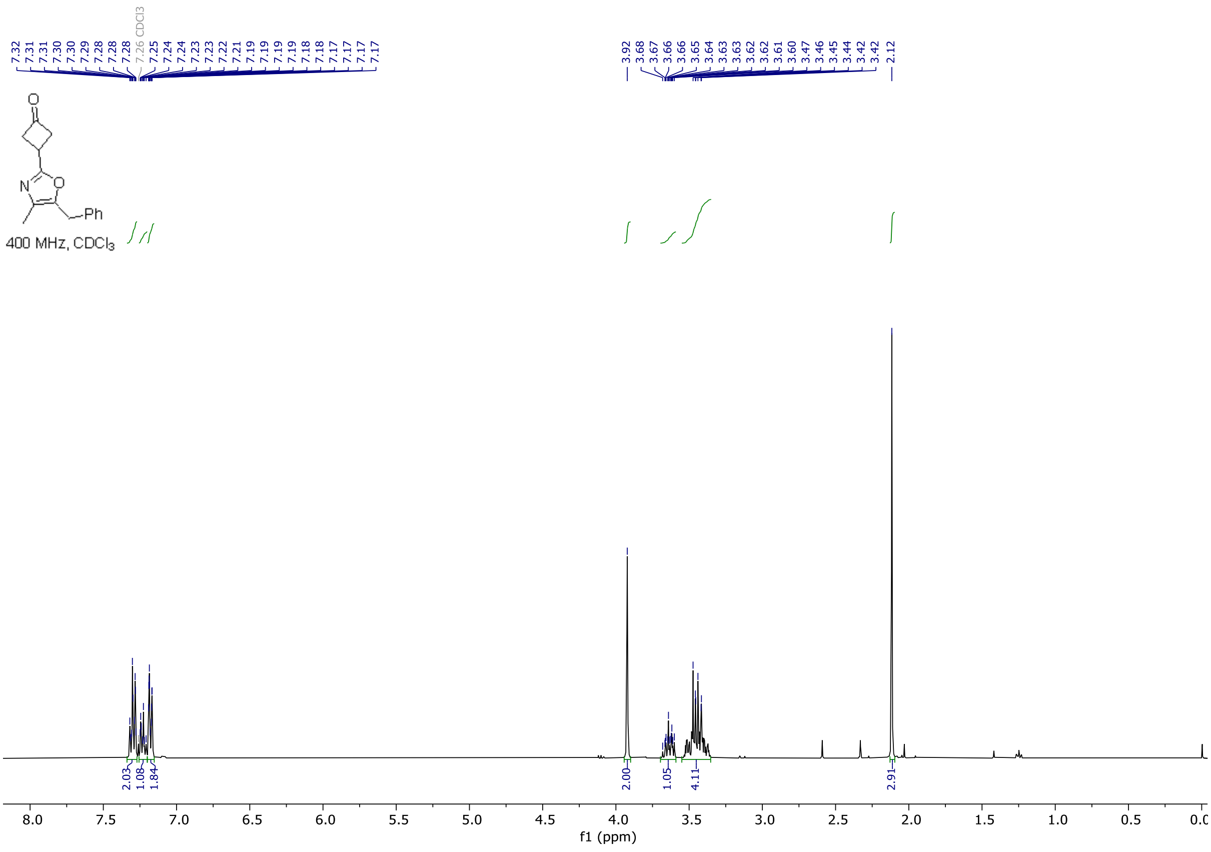

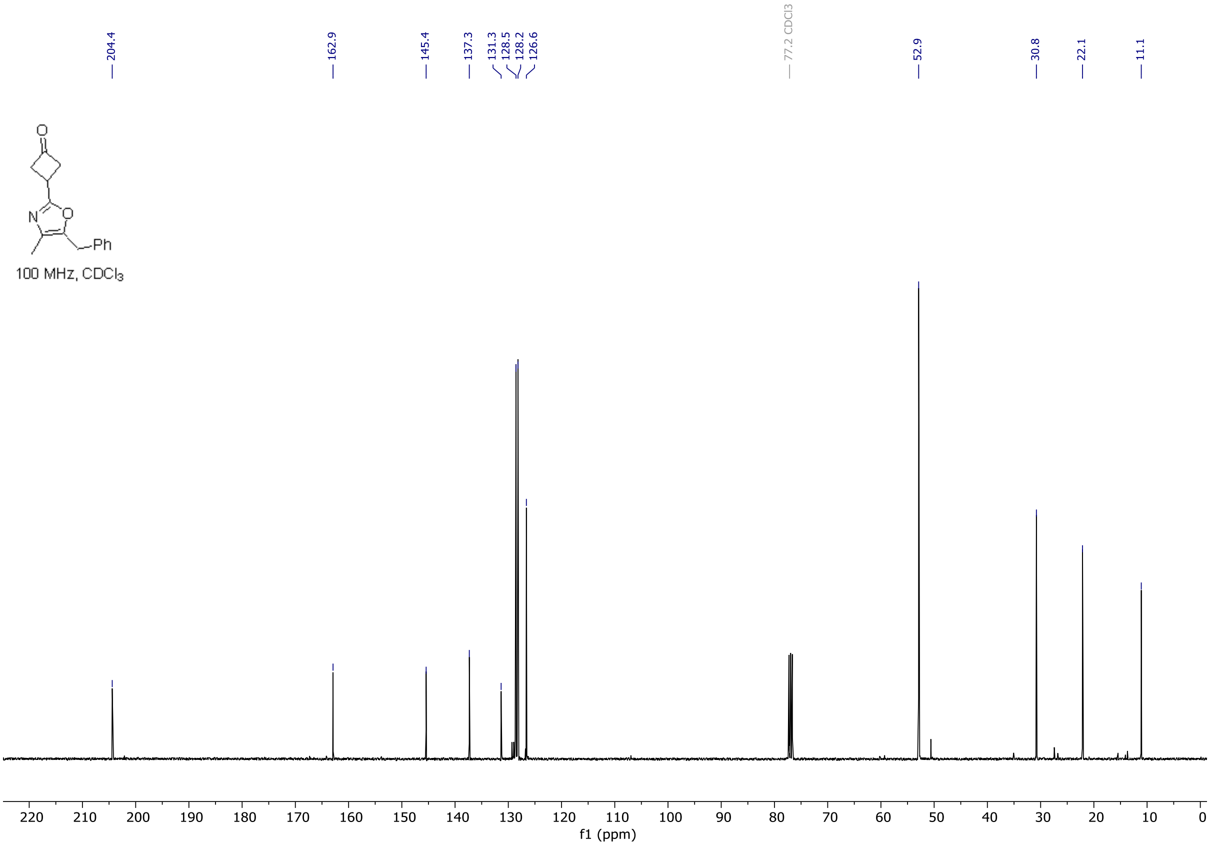
204.4
162.9
145.4
137.3
131.3
128.5
128.2
126.6
77.2 CDCl3
52.9
30.8
22.1
11.1
O
N
O
Ph
100 MHz, CDCl3
f1 (ppm)

HSQC Data

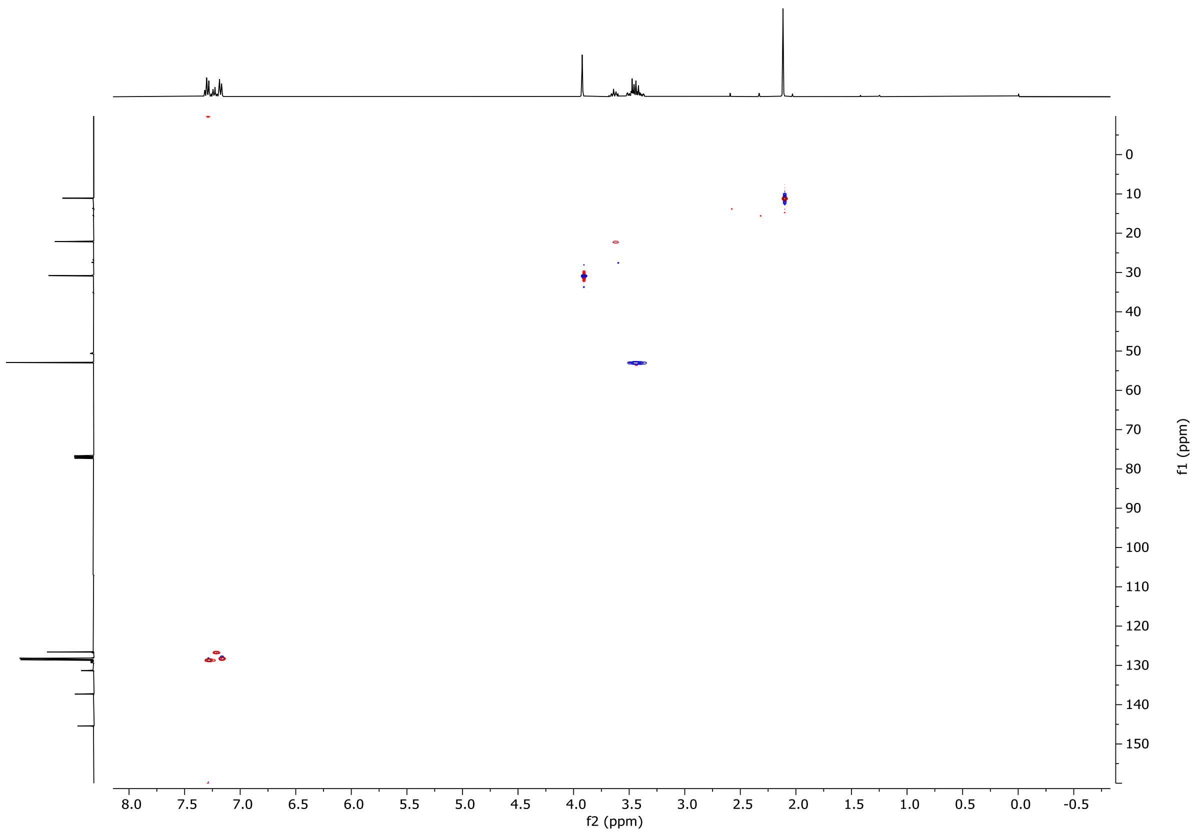
f2 (ppm)
f1 (ppm)

2-((3r,5r,7r)-adamantane-1-carbonyl)-4-benzyl-3-methylisoxazol-5(2*H*)-one (2k)

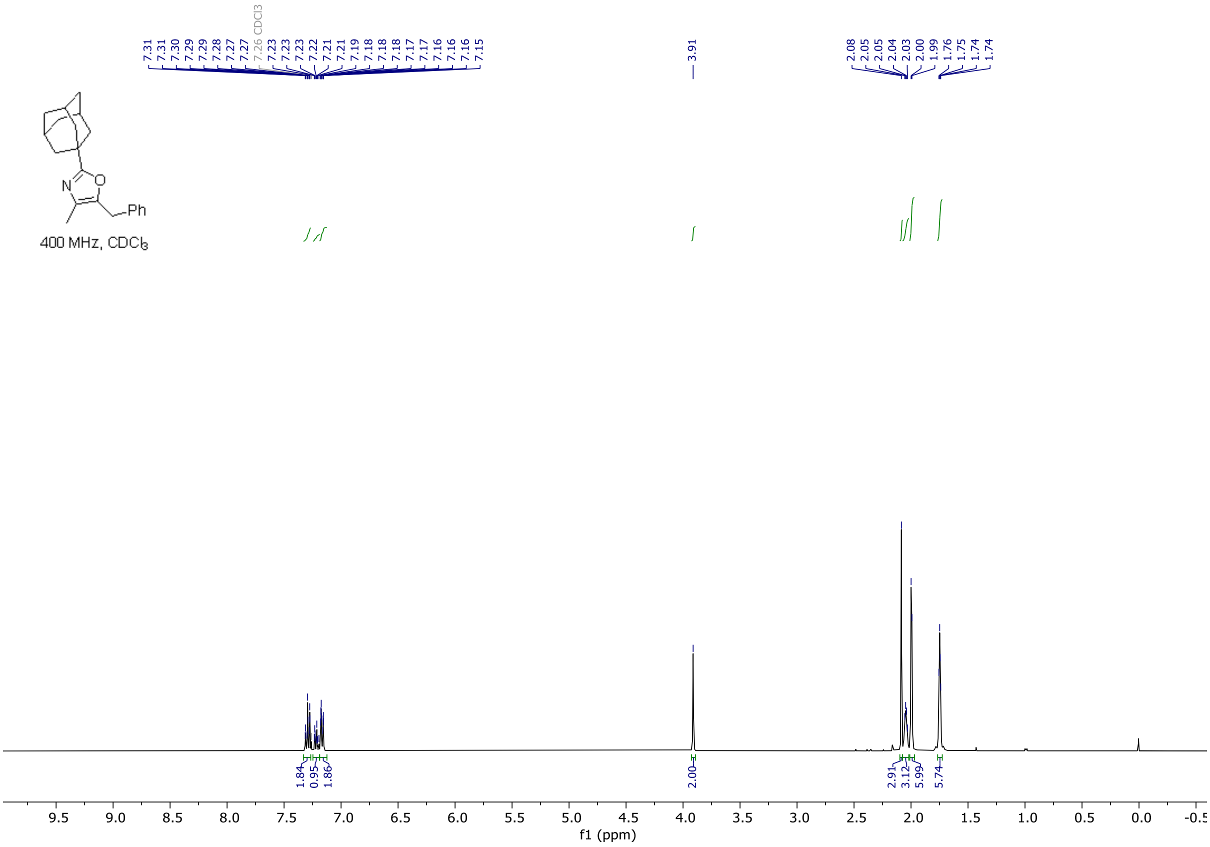

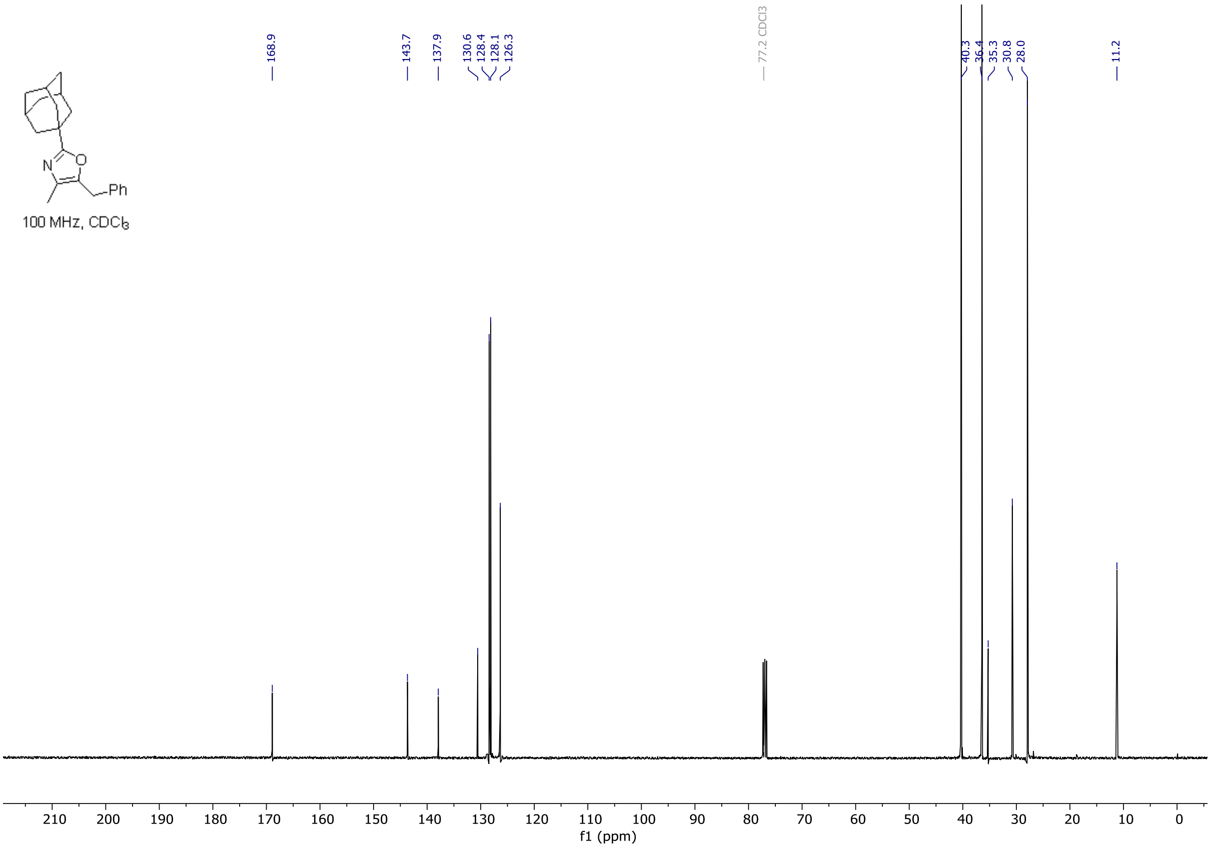

5-Benzyl-2-isobutoxy-4-methyloxazole (2l)

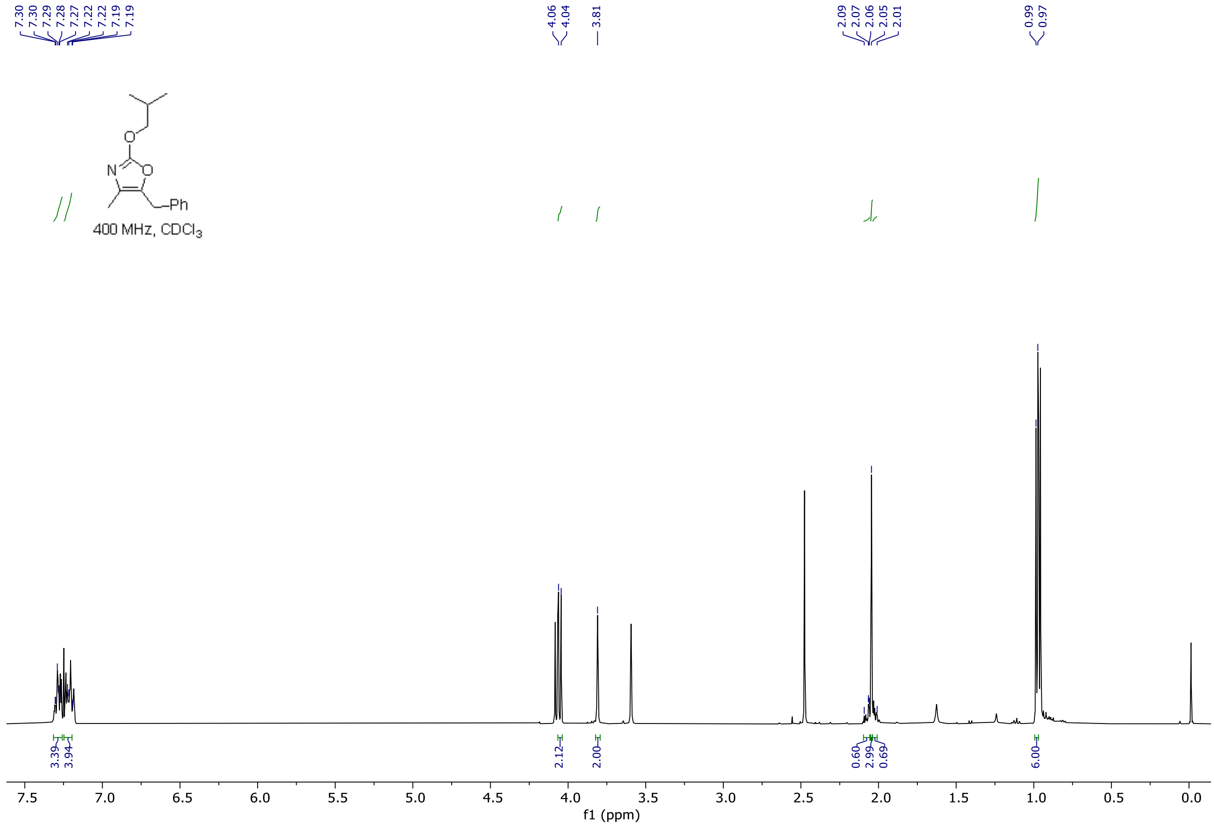

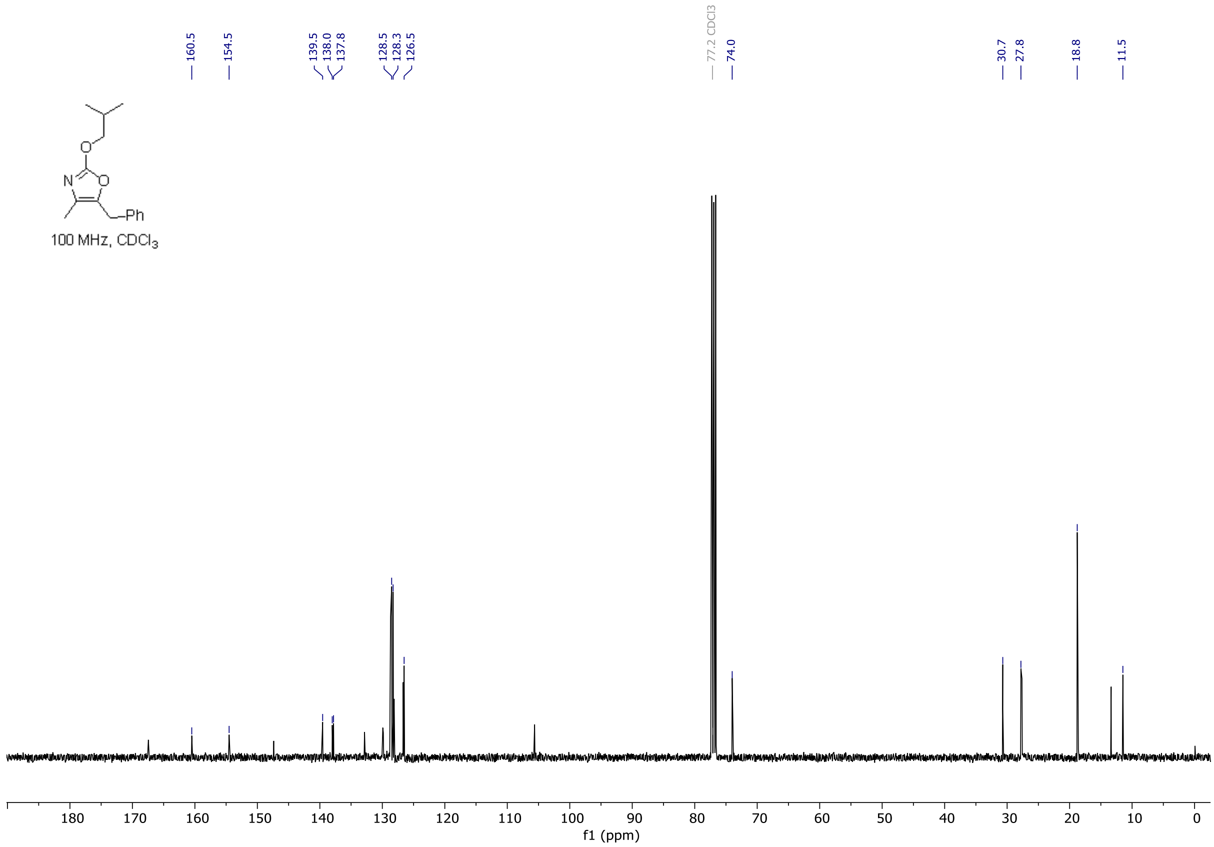

HSQC Data

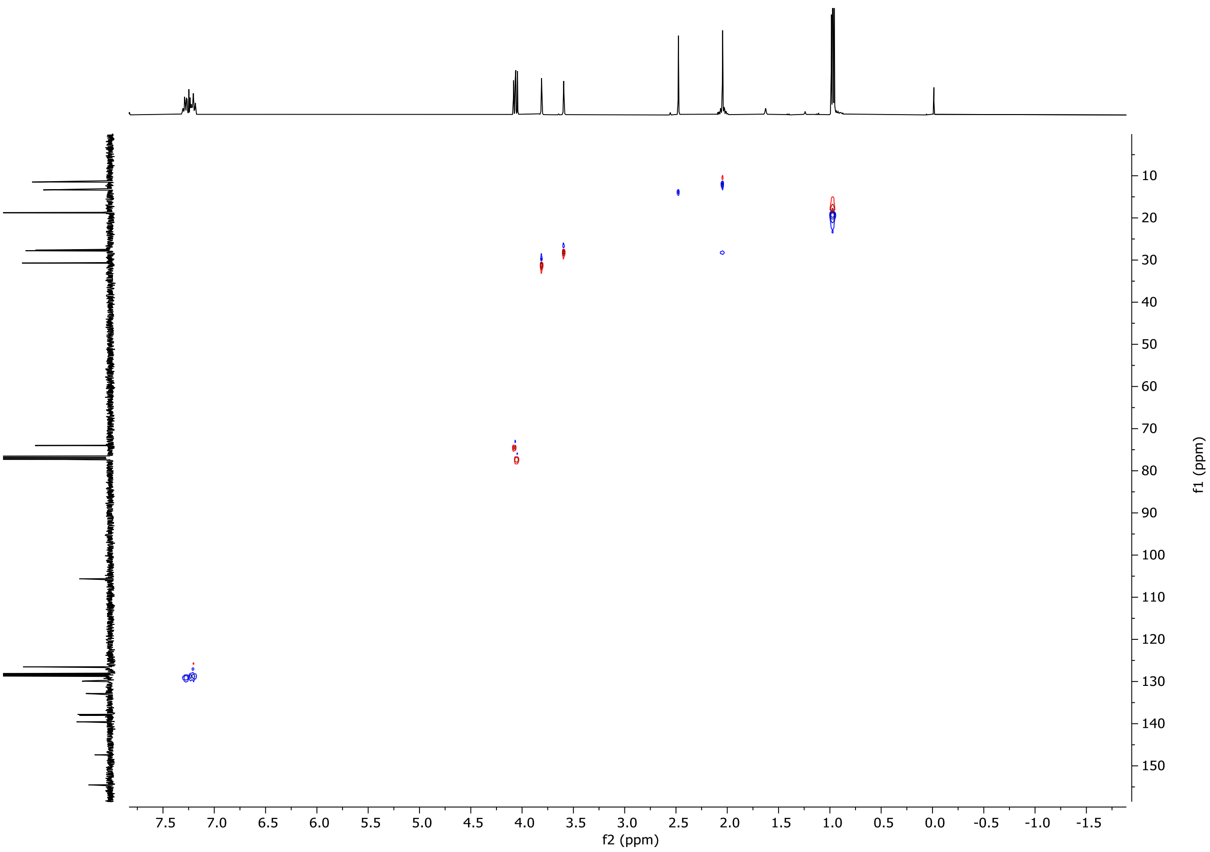

5-Benzyl-4-cyclopropyl-2-methyloxazole (2n)

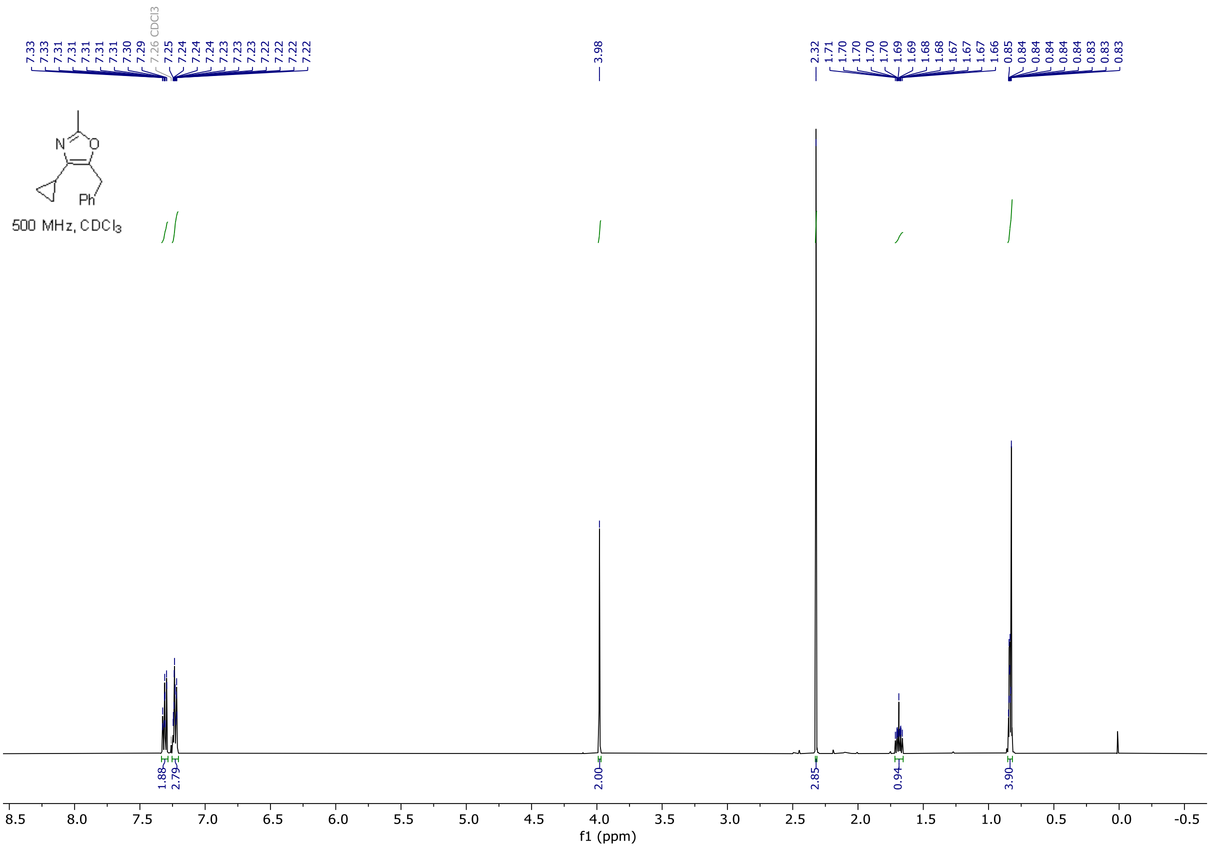

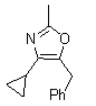

125 MHz, $CDCl_3$

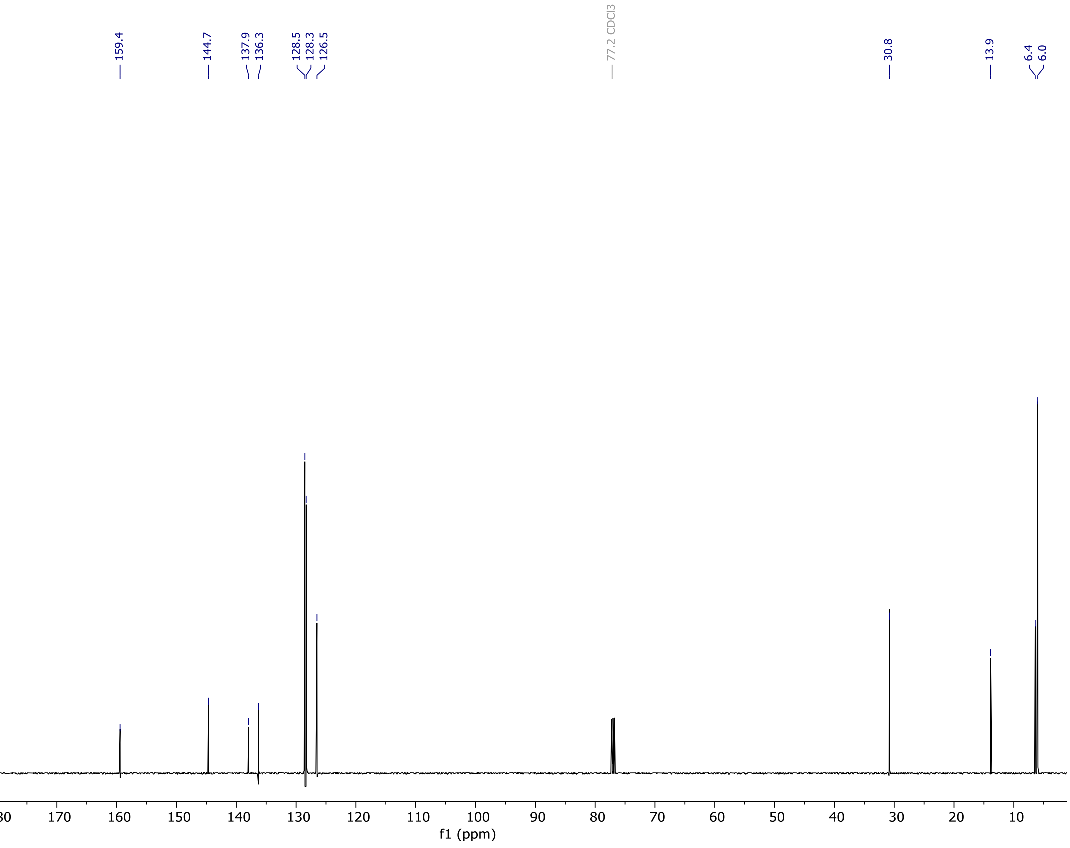

HSQC Data

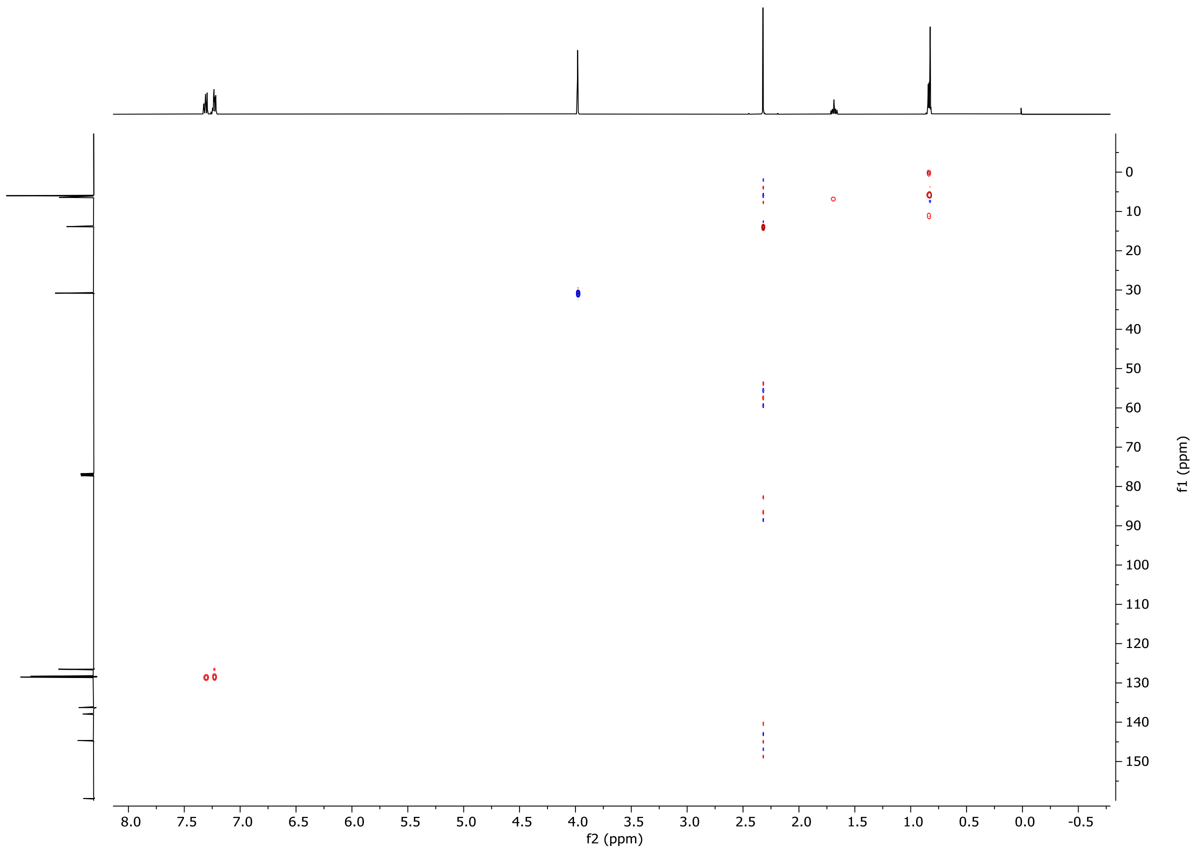

2-((2-Isopropyl-4-methyloxazol-5-yl)methyl)phenol (2o)

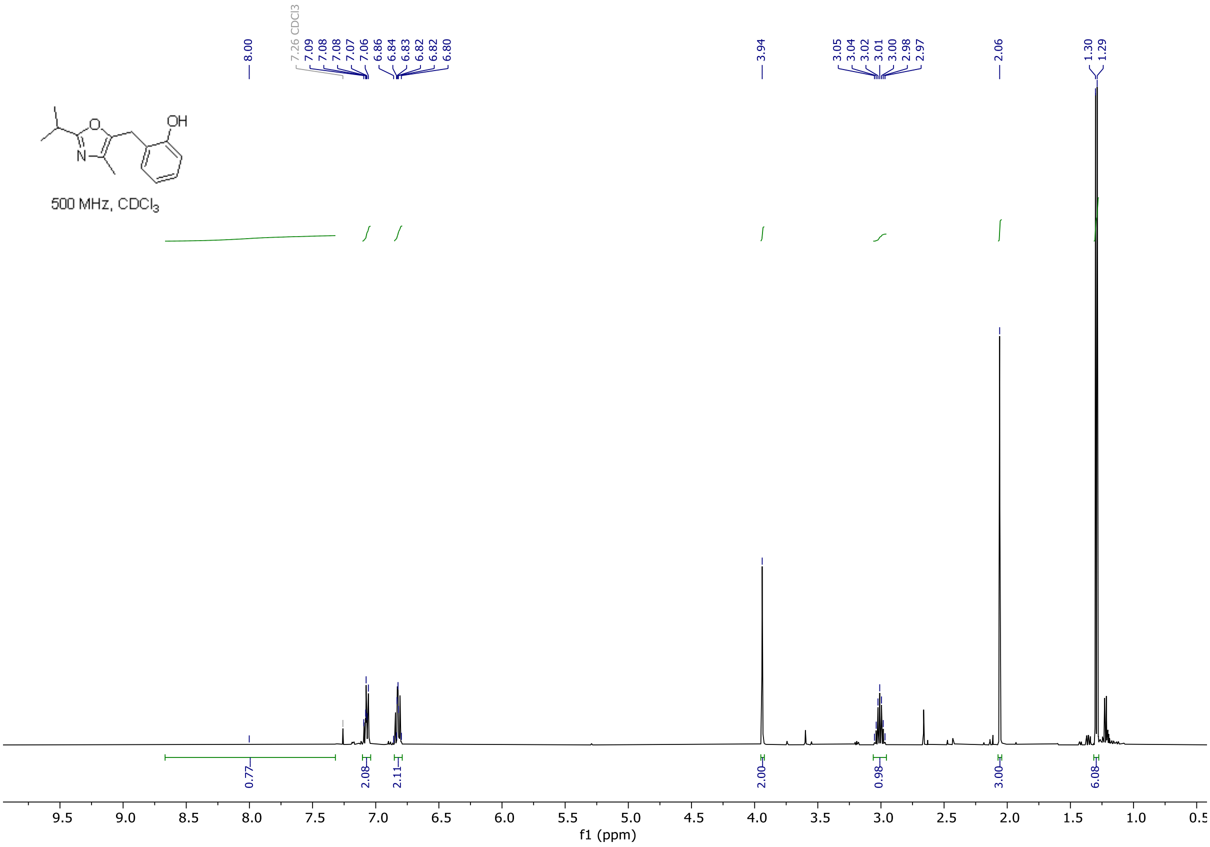

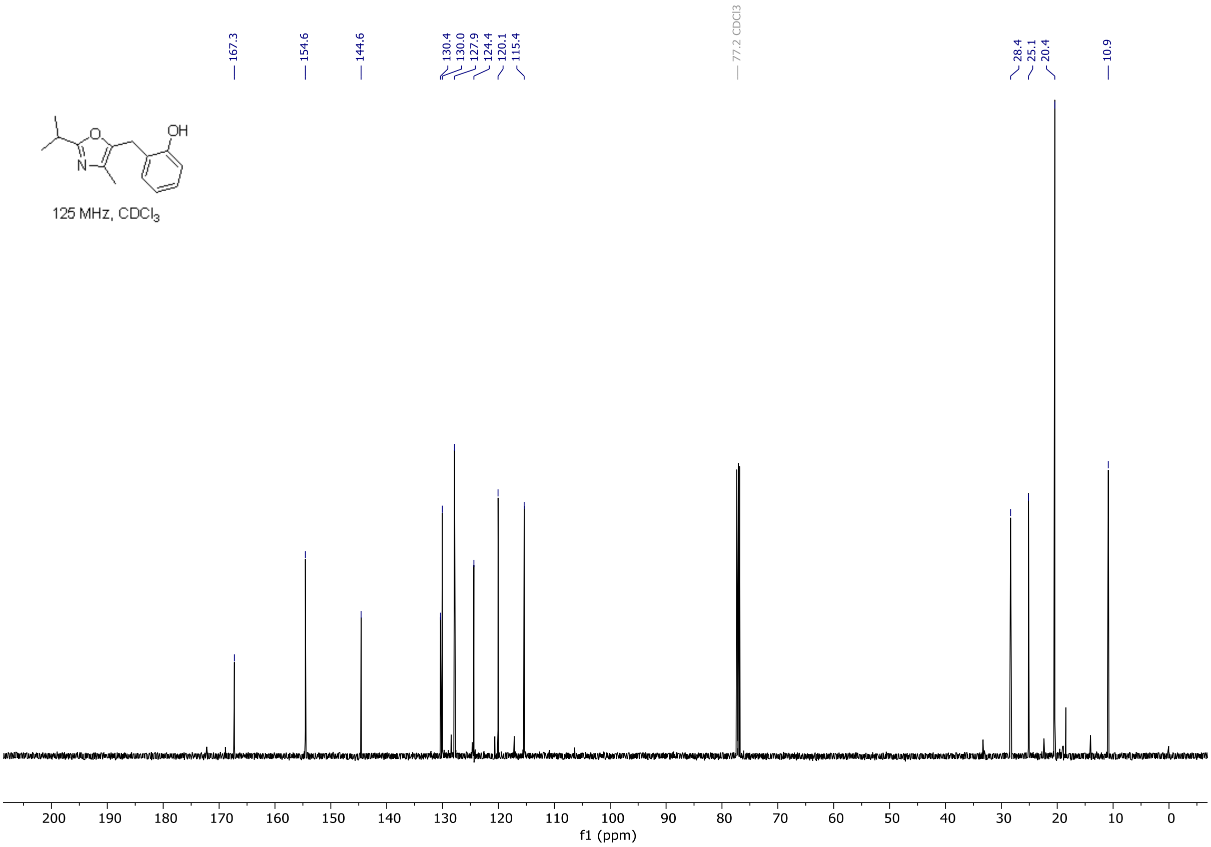

HSQC Data

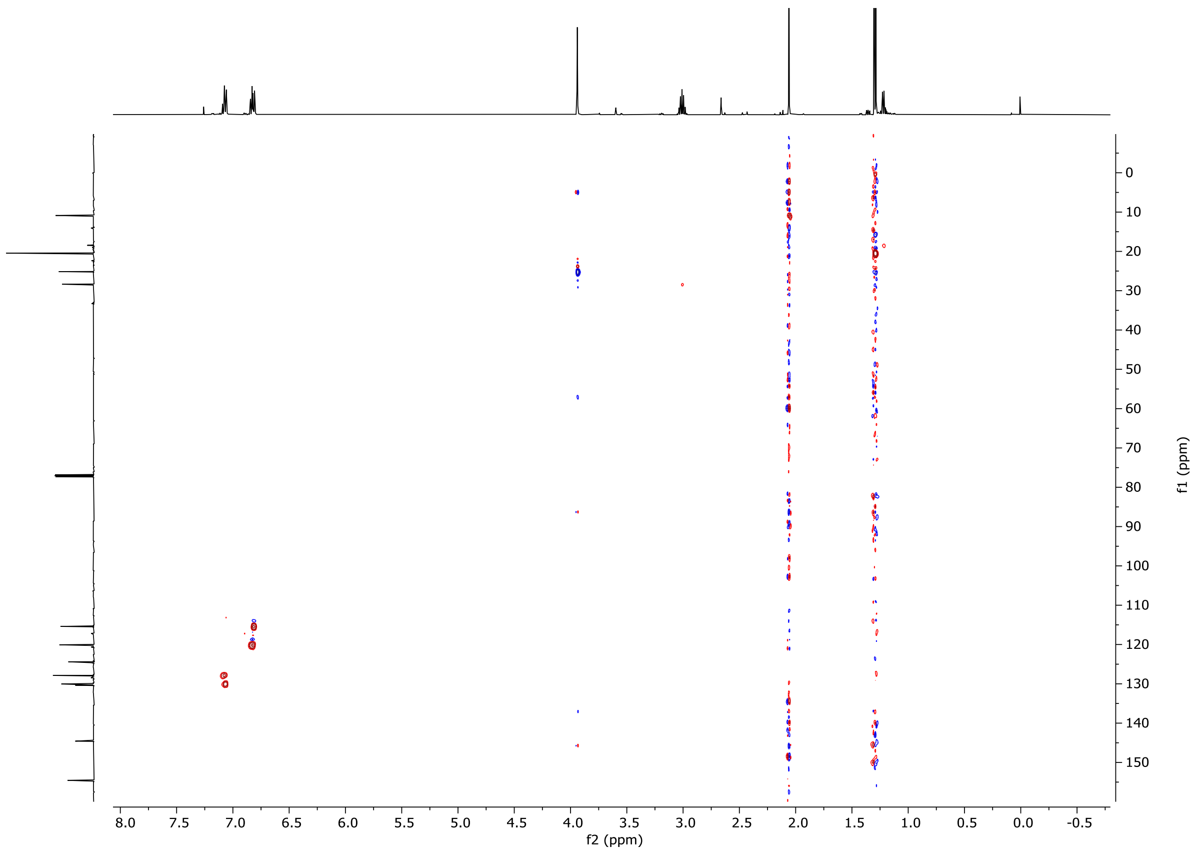

5-(4-Bromobenzyl)-2-isopropyl-4-methyloxazole (2p)

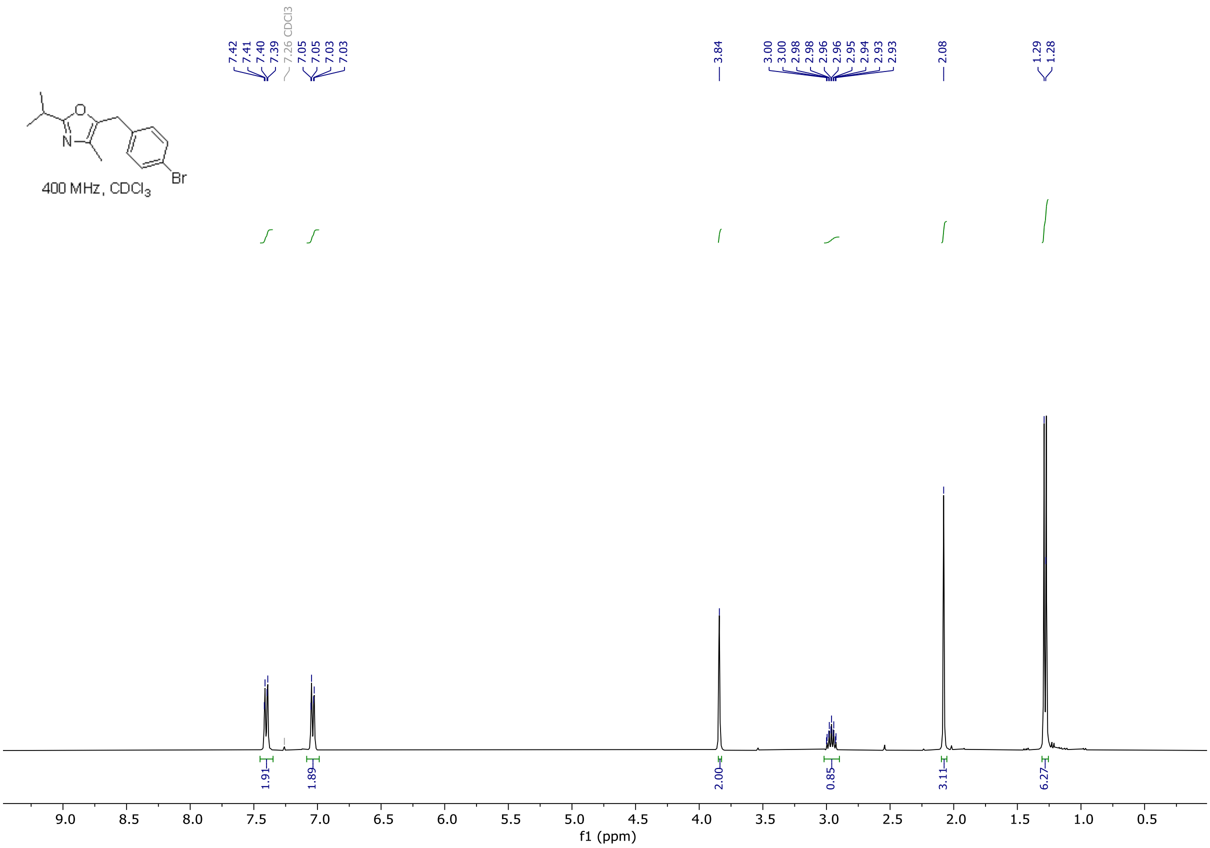

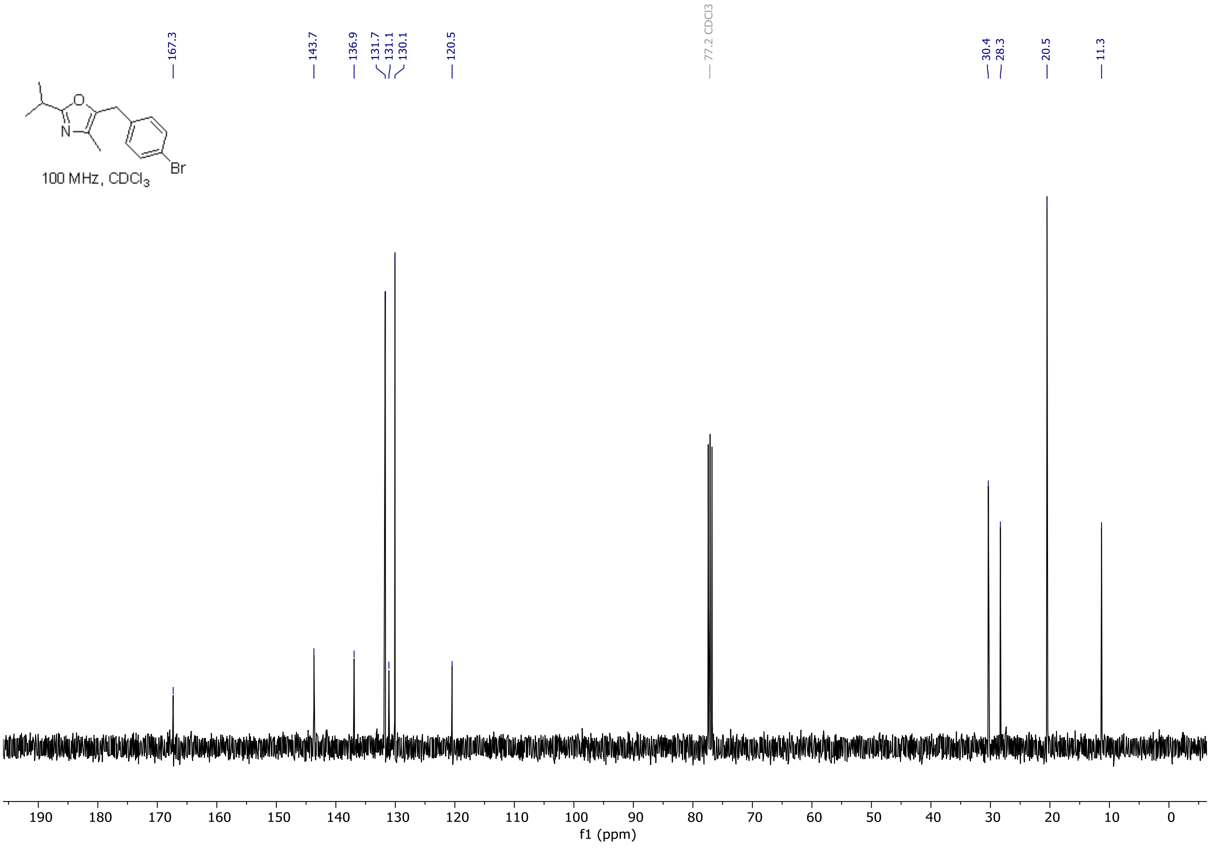
100 MHz, CDCl3
167.3
143.7
136.9
131.7
131.1
130.1
120.5
77.2 CDCl3
30.4
28.3
20.5
11.3
f1 (ppm)

HSQC Data

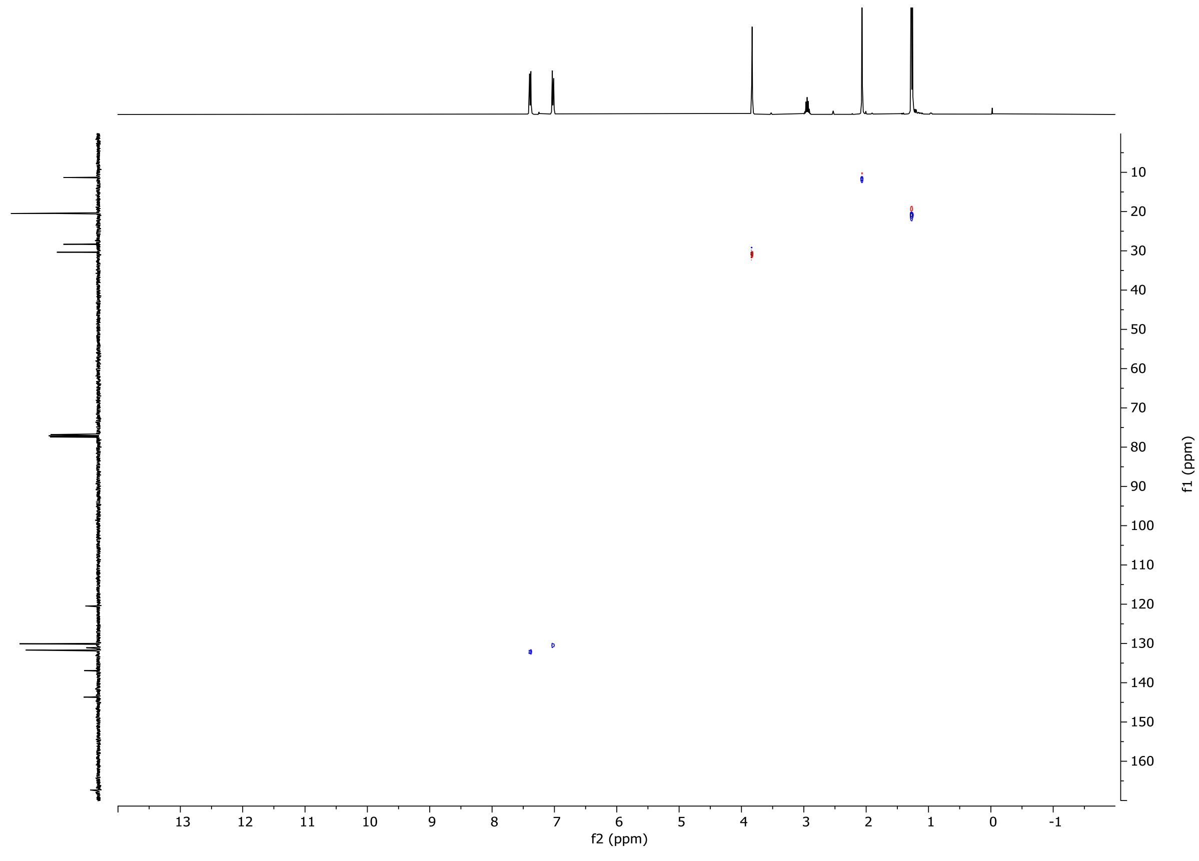
f2 (ppm)
f1 (ppm)

tert-Butyl 3-((2-isopropyl-4-methyloxazol-5-yl)methyl)-1H-indole-1-carboxylate (2r)

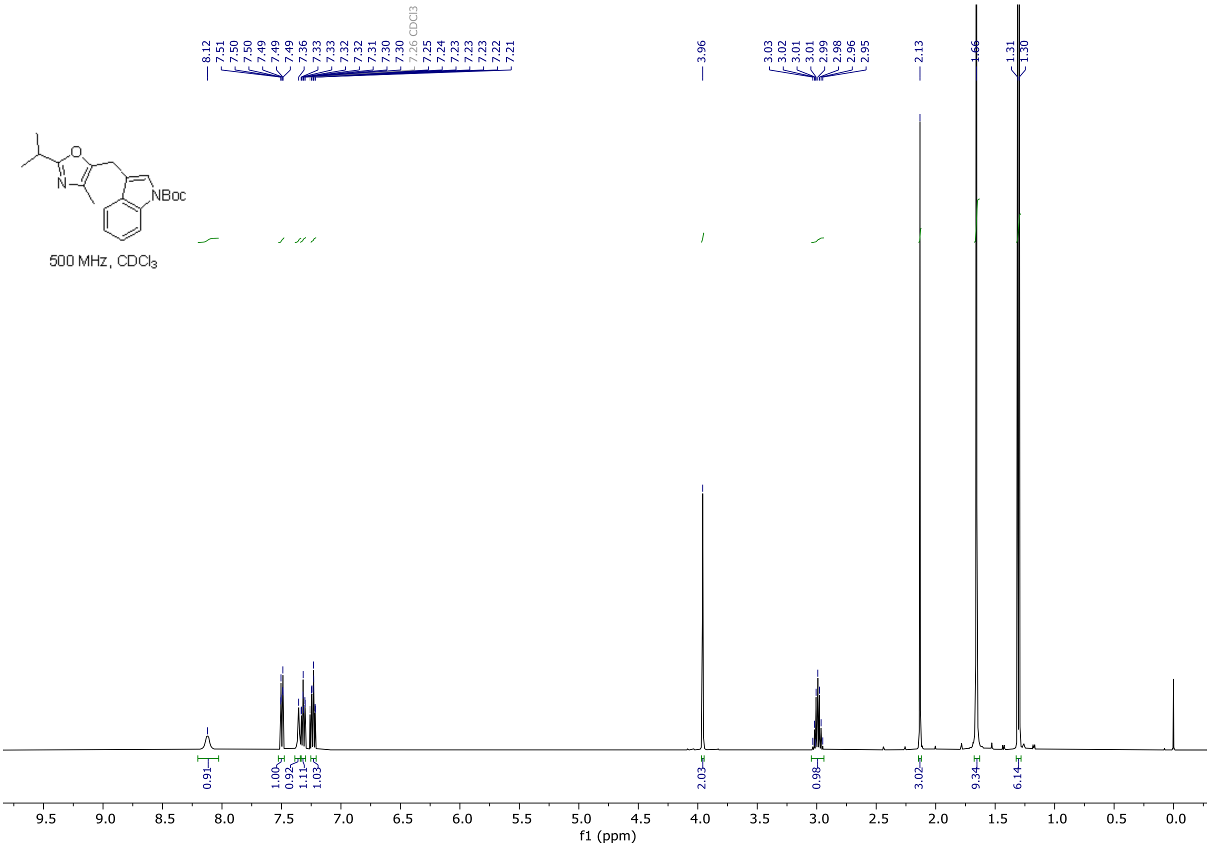

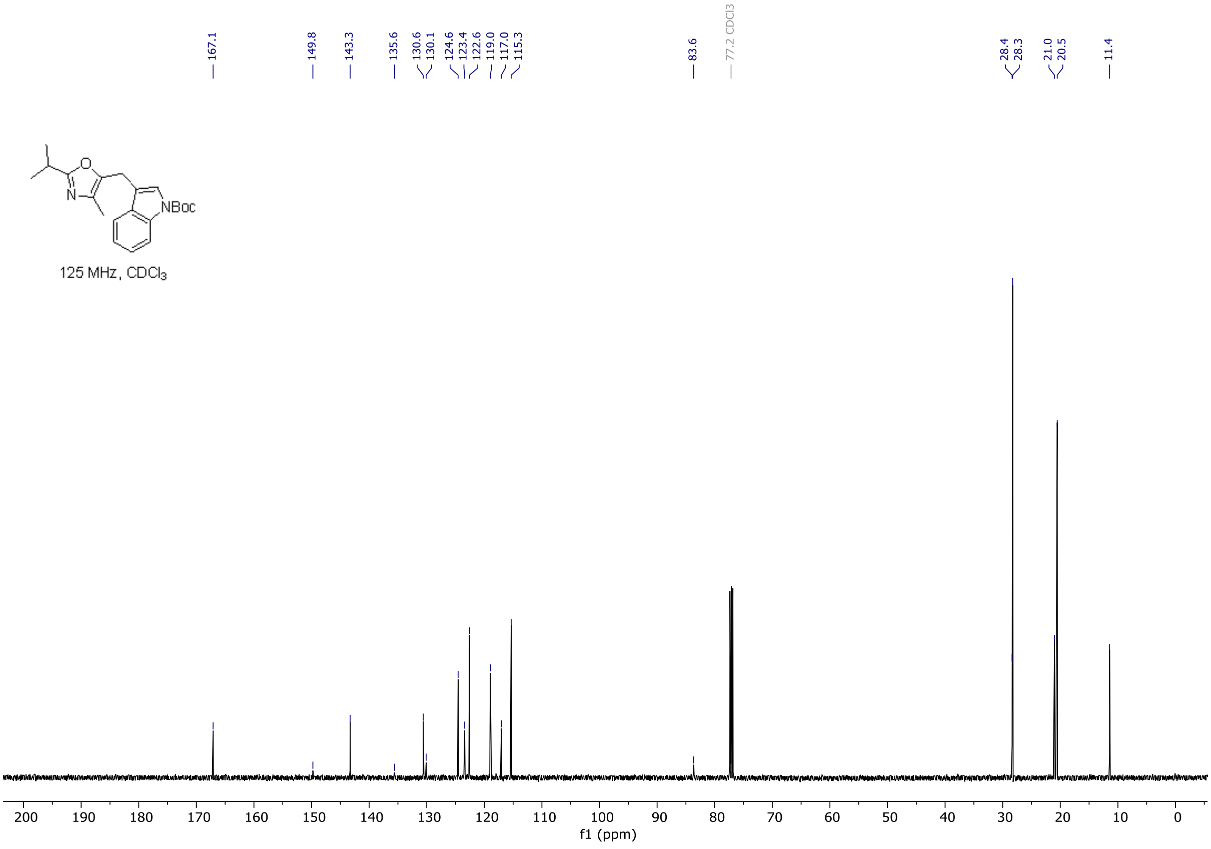

HSQC Data

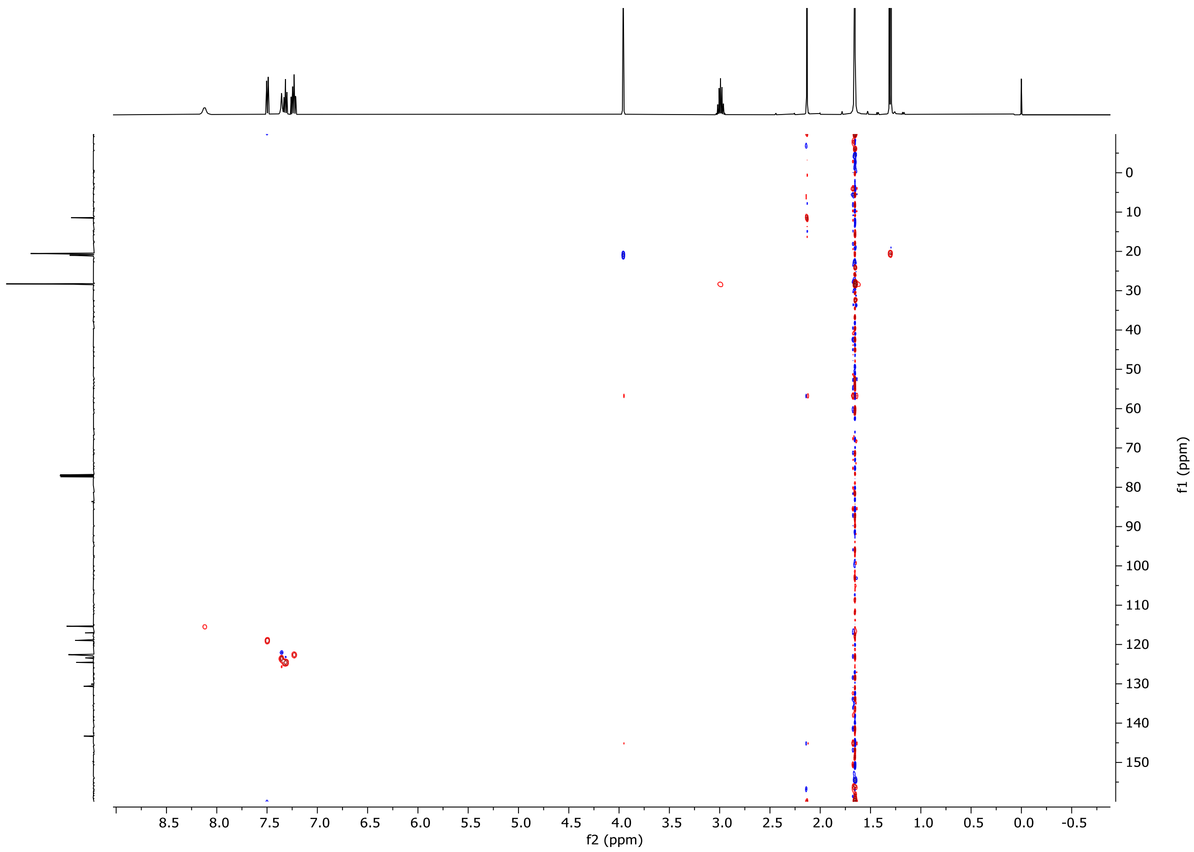

2-Isopropyl-4-methyl-5-(thiophen-2-ylmethyl)oxazole (2s)

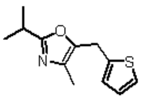

400 MHz, $CDCl_3$

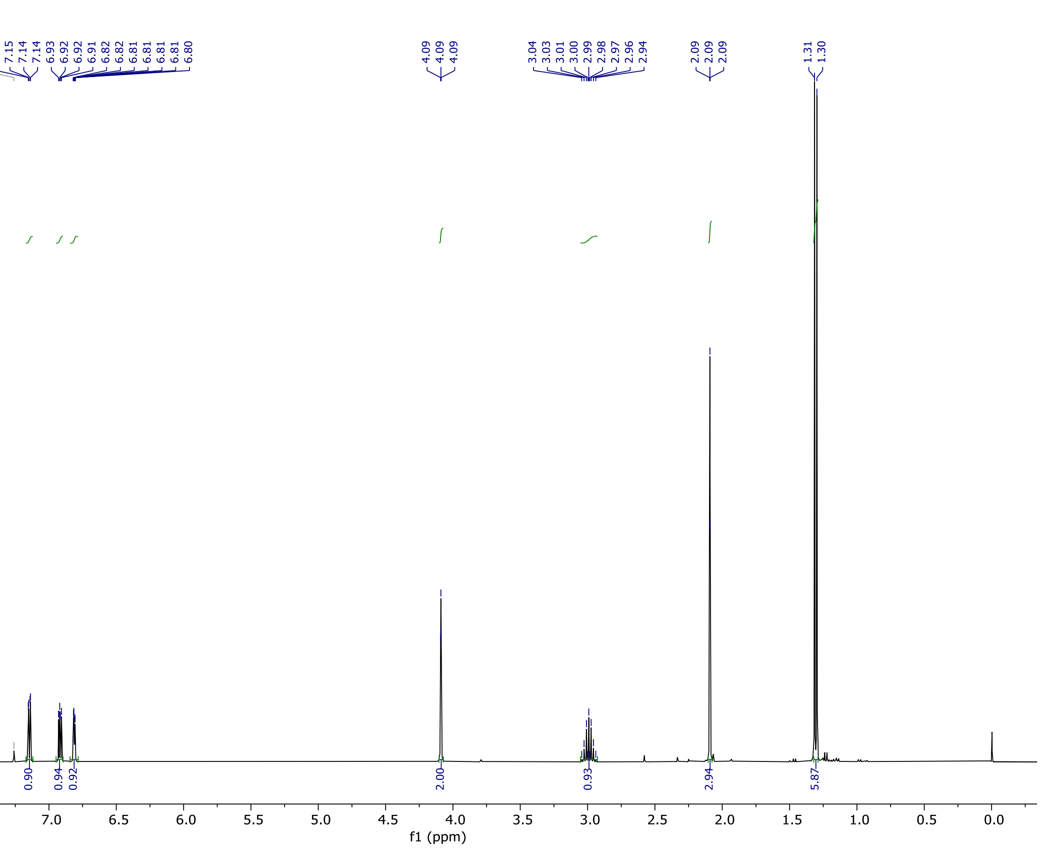

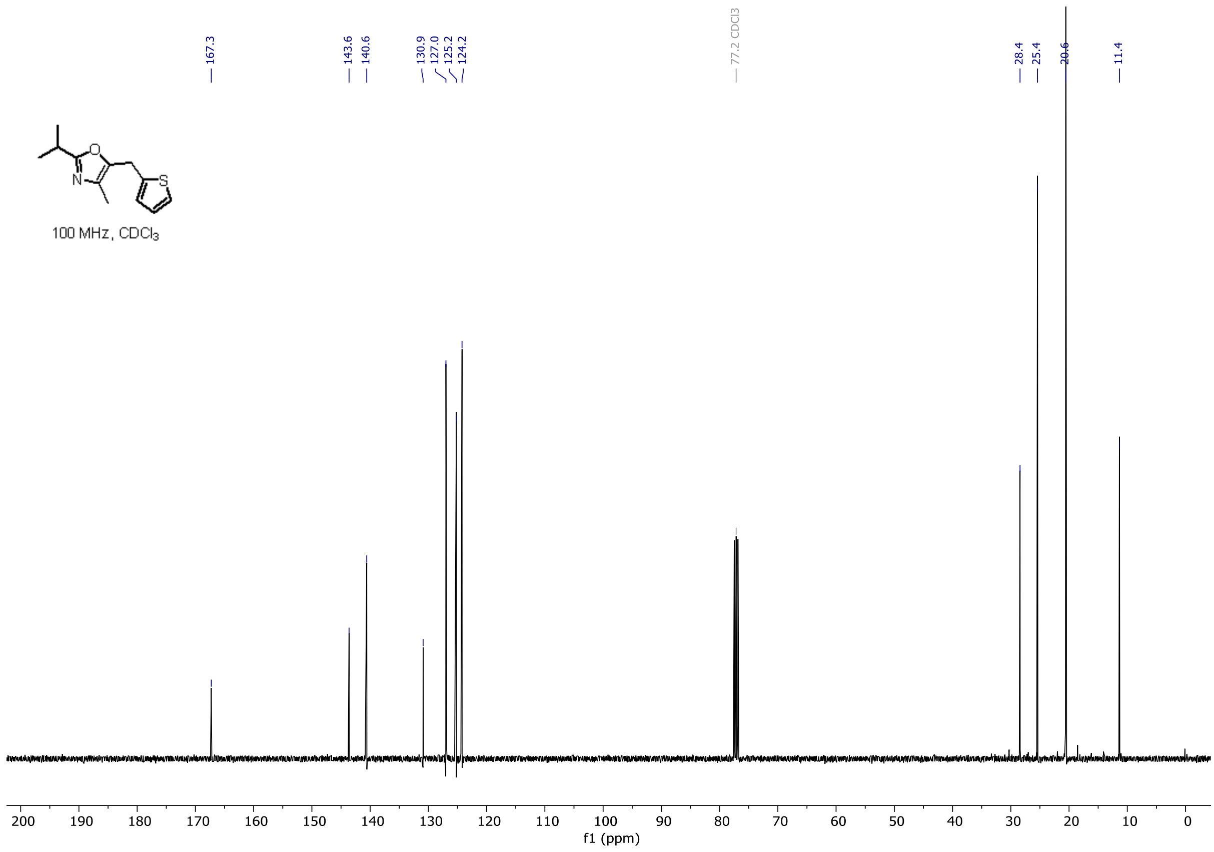
167.3
143.6
140.6
130.9
127.0
125.2
124.2
77.2 CDCl3
28.4
25.4
20.6
11.4
100 MHz, $CDCl_3$
f1 (ppm)

HSQC Data

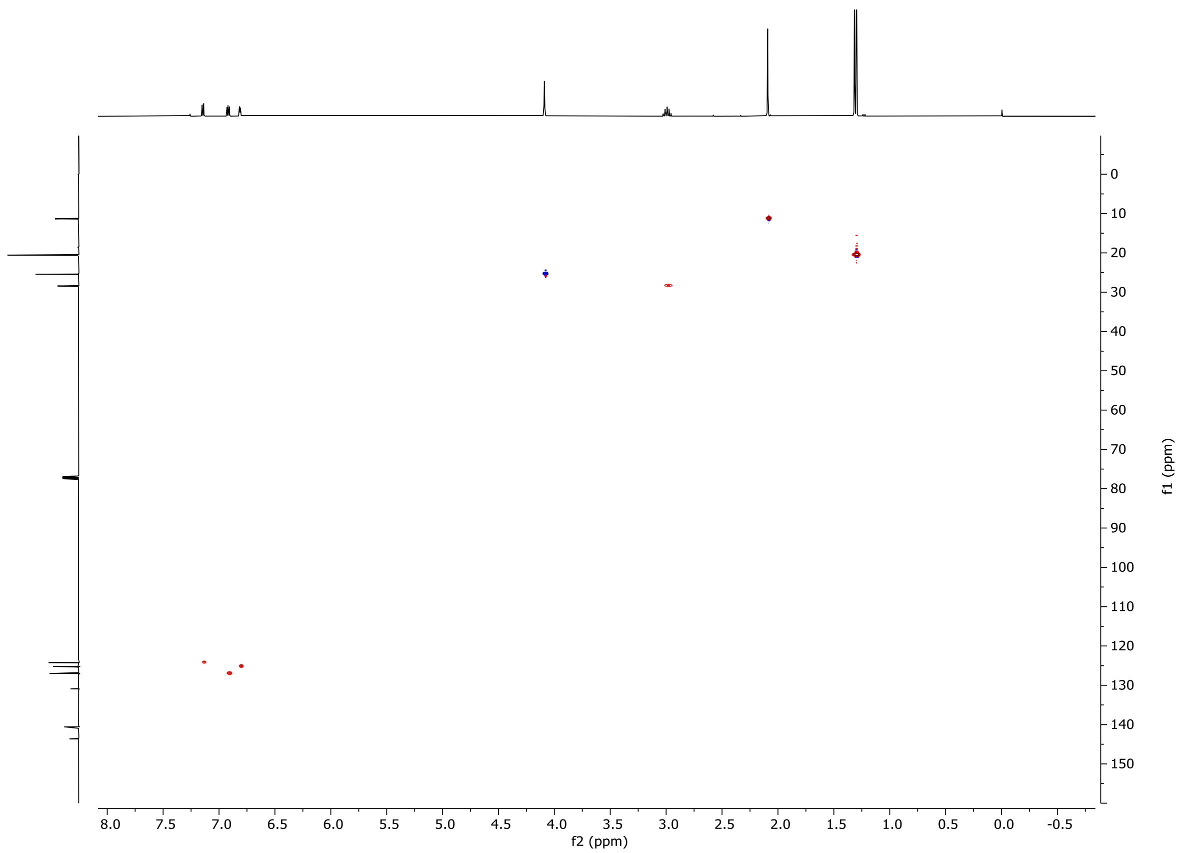
f1 (ppm)
f2 (ppm)

Methyl 3-(5-benzyl-4-methyloxazol-2-yl)propanoate (2t)

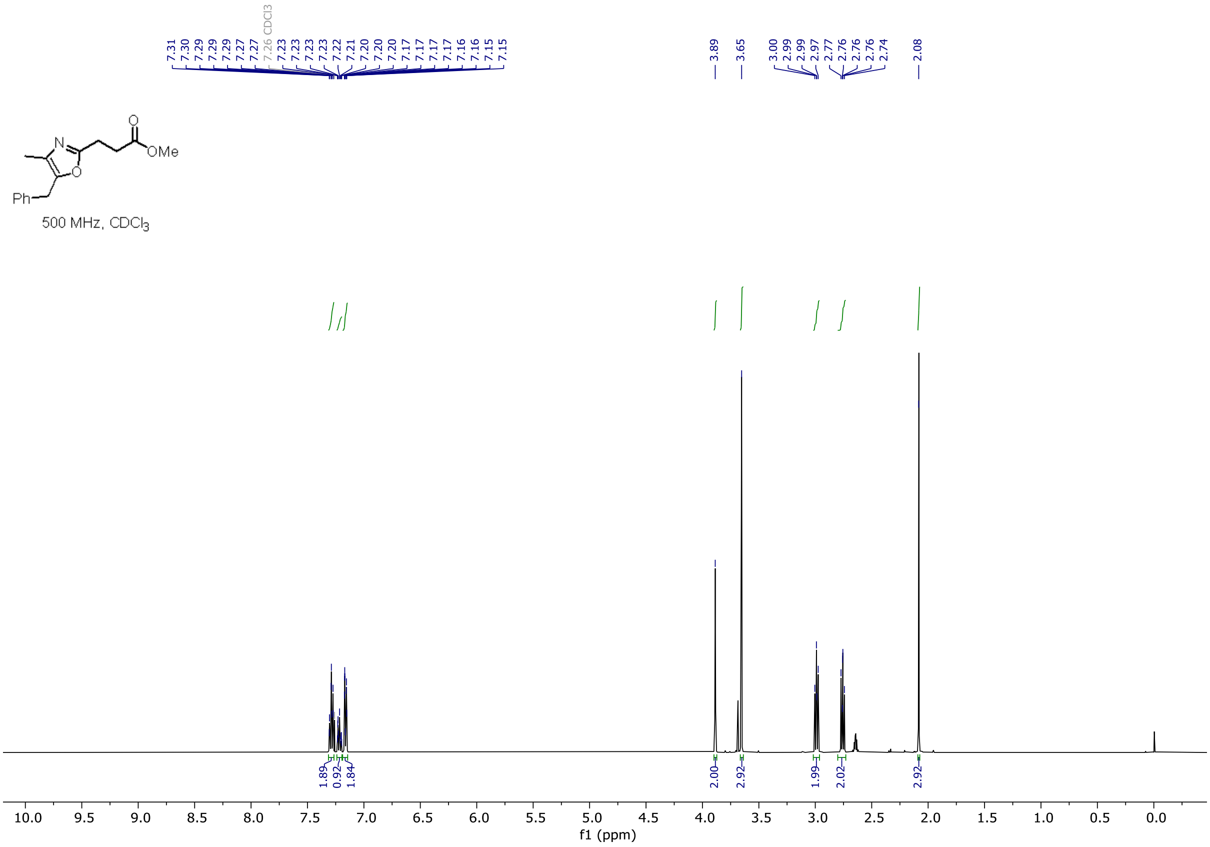

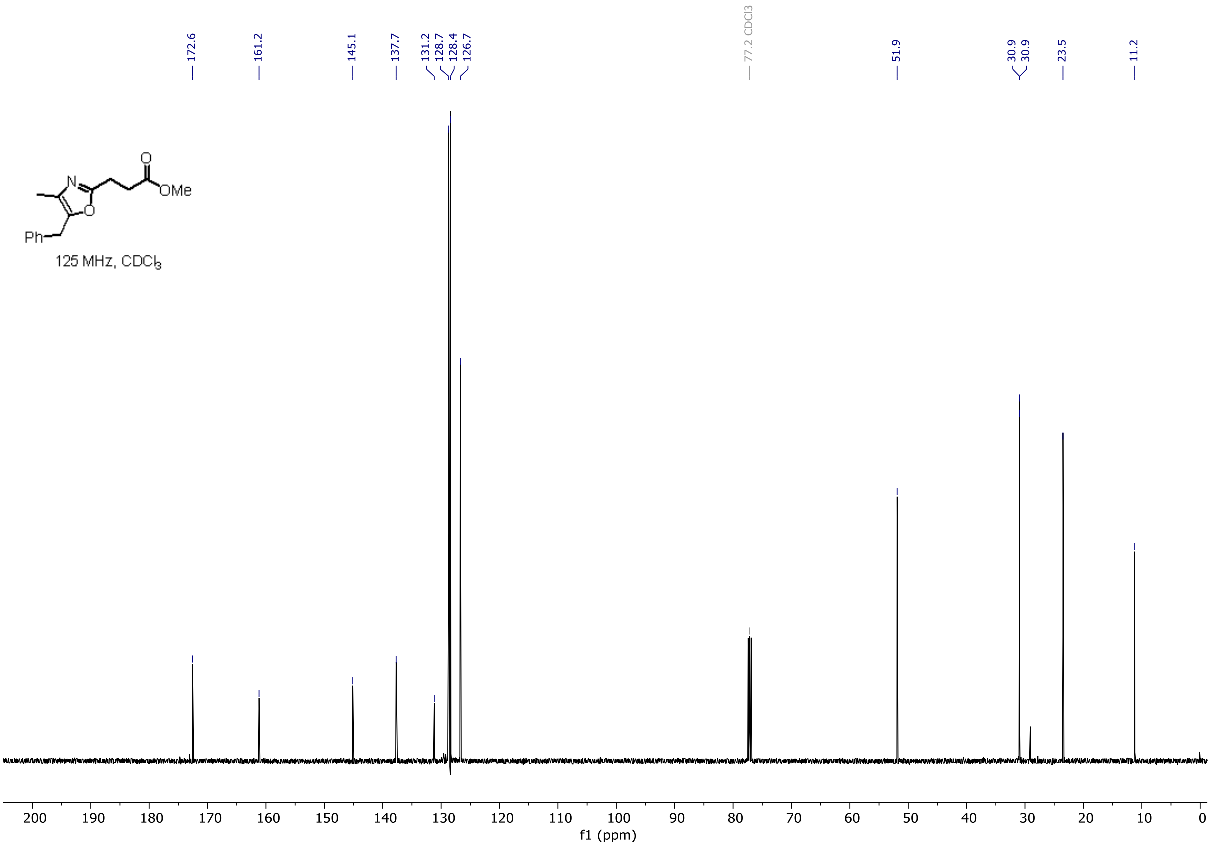

HSQC Data

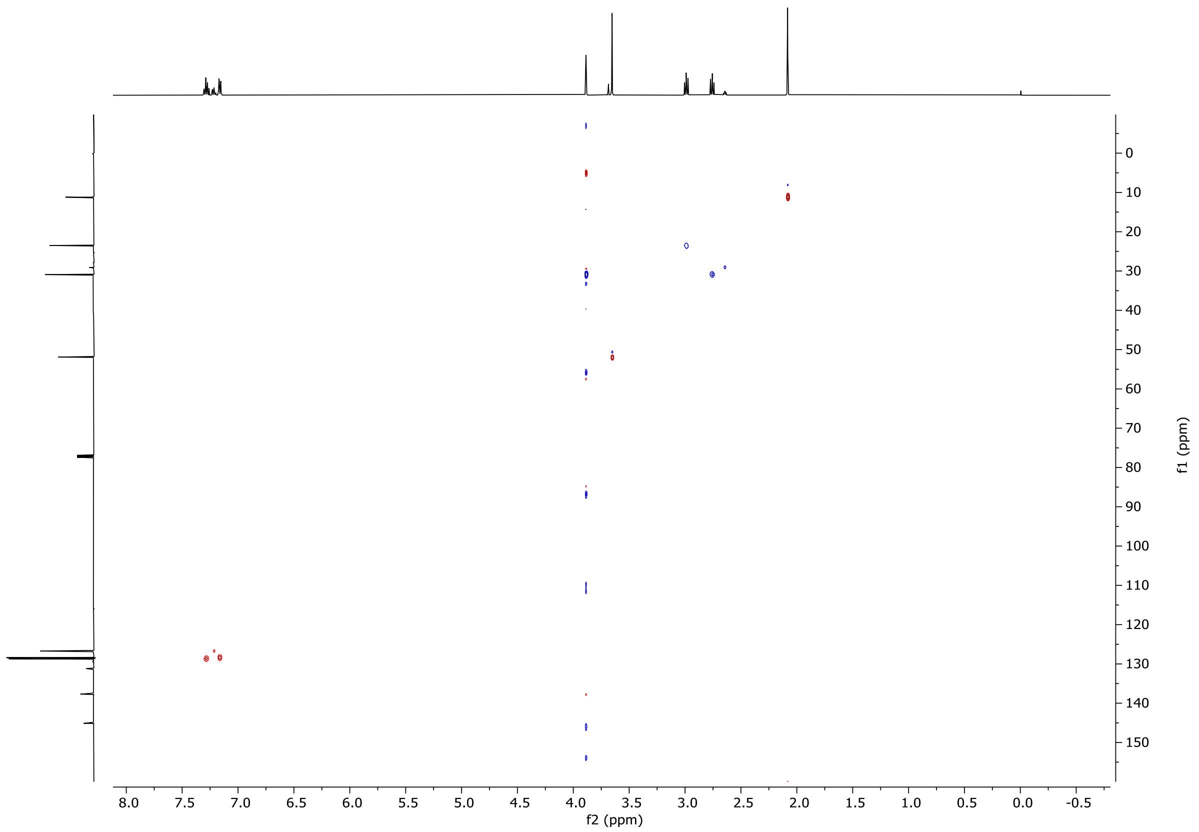

3-(5-Benzyl-4-methyloxazol-2-yl)propanoic acid (3a)

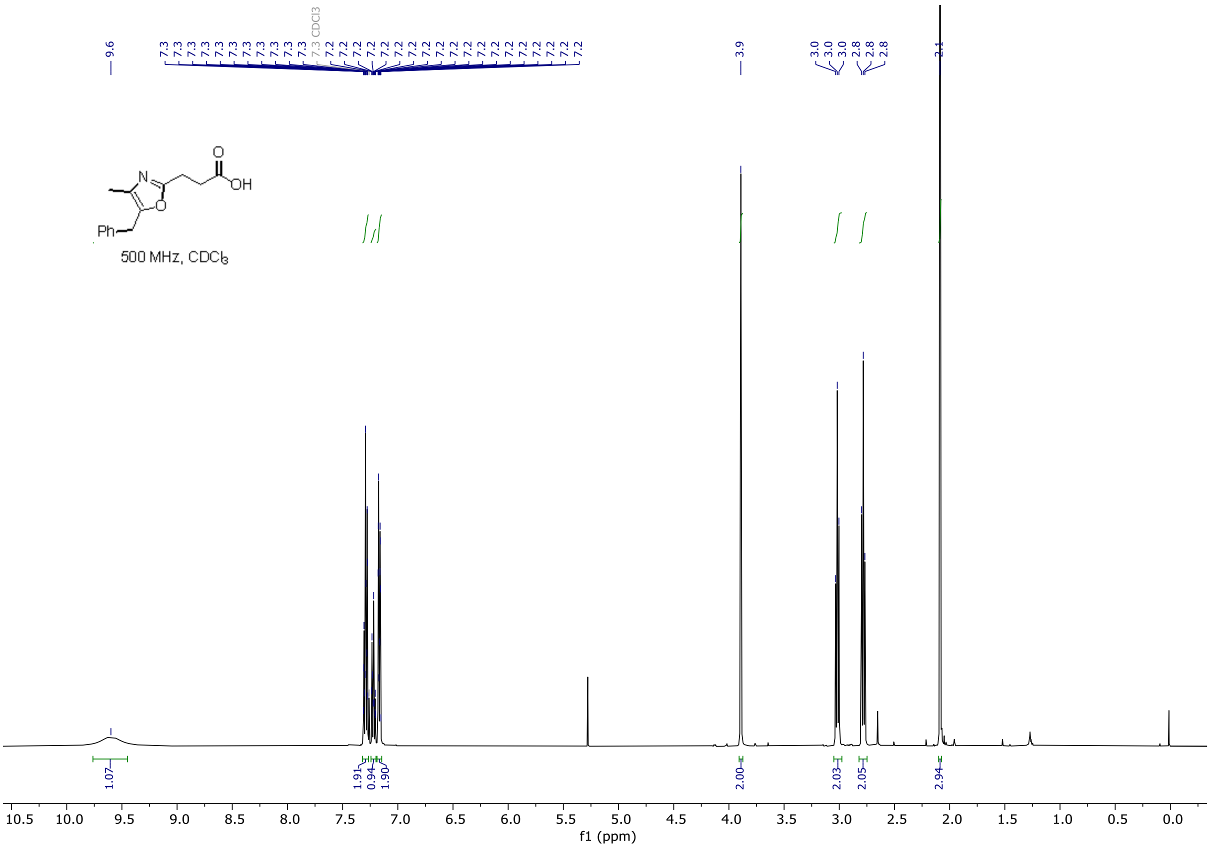

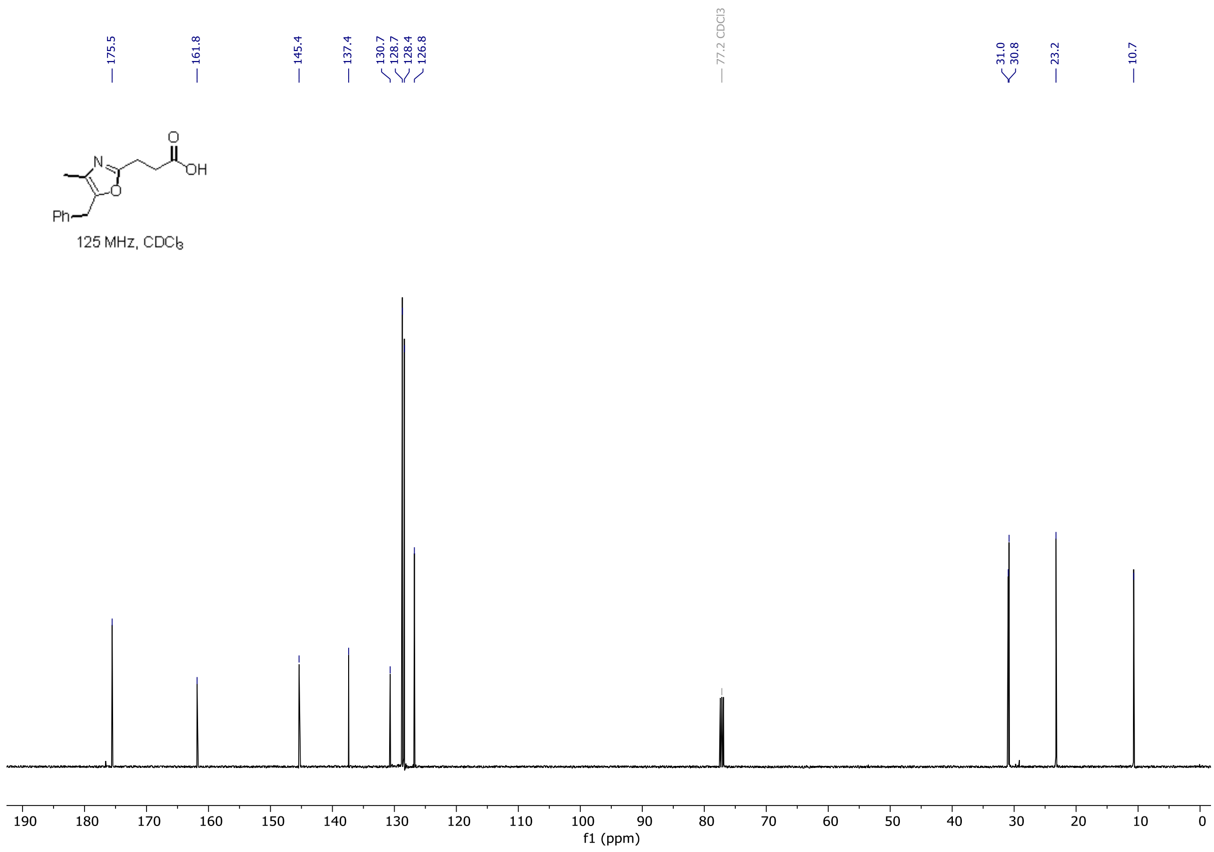
175.5
161.8
145.4
137.4
130.7
128.7
128.4
126.8
77.2 CDCl3
31.0
30.8
23.2
10.7
N
O
OH
O
Ph
125 MHz, CDCl3
f1 (ppm)

HSQC Data

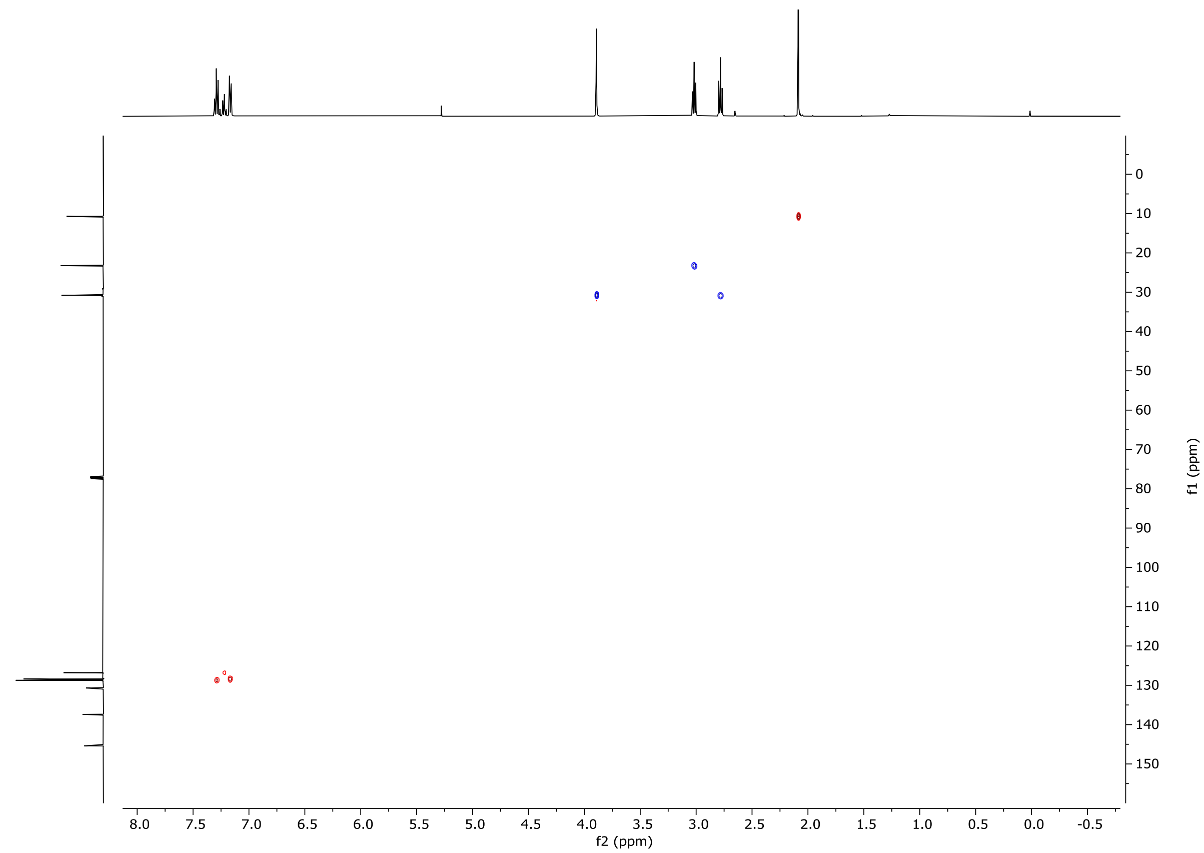
f2 (ppm)
f1 (ppm)
